# Supplementary material for: A Chiron approach towards the stereoselective synthesis of polyfluorinated carbohydrates
Source: Nat Commun. 2018 Nov 9;9:4721. doi: 10.1038/s41467-018-06901-y (PMC6226540; doi:10.1038/s41467-018-06901-y)
Supplement: Supplementary file 1 — Supplementary Information [file 41467_2018_6901_MOESM1_ESM.pdf]

Supplementary Information

A Chiron approach towards the stereoselective synthesis of polyfluorinated carbohydrates

Vincent Denavit, Danny Lainé, Jacob St-Gelais, Paul A. Johnson, and Denis Giguère*

Département de chimie, 1045 av. De la Médecine, Université Laval, Québec City, Qc, Canada G1V 0A6

Supplementary Methods

General Methods

All reactions were carried out under an argon atmosphere with dry solvents under anhydrous conditions, unless otherwise noted. Methylene chloride (CH_2Cl_2) was distilled from CaH_2 and tetrahydrofuran (THF) was distilled from Na/benzophenone immediately before use. Yields refer to chromatographically and spectroscopically (^{1}H NMR) homogeneous materials, unless otherwise stated. Reagents were purchased at the highest commercial quality available and used without further purification, unless otherwise stated. Reactions were monitored by thin-layer chromatography (TLC) carried out on 0.25 mm E. Merck silica gel plates (60F-254) using UV light as visualizing agent and charring with a solution of 3 g of PhOH and 5 mL of H_2SO_4 in 100 mL of EtOH, followed by heating with a heatgun. SiliaFlash® P60 40-63 µm (230-400 mesh) was used for flash column chromatography. NMR spectra were recorded with an Agilent DD2 500 MHz spectrometer and calibrated using residual undeuterated solvent ($CDCl_3$: ^{1}H δ = 7.26 ppm, ^{13}C δ = 77.16 ppm) as an internal reference. Calibration of ^{19}F NMR was performed using hexafluorobenzene, which have been measured at -162.29 ppm compared to the chemical shift of reference compound $CFCl_3$. Coupling constants (*J*) are reported in Hertz (Hz), and the following abbreviations were used to designate multiplicities: s = singlet, d = doublet, t = triplet, q = quartet, p = quintet, m = multiplet, br = broad. Assignments of NMR signals were made by homonuclear (COSY) and heteronuclear (HSQC, HMBC, HOESY, ^{19}F c2HSQC) two-dimensional correlation spectroscopy. Infrared spectra were recorded using a Thermo Scientific Nicolet 380 FT-IR spectrometer. The absorptions are given in wavenumbers (cm^{-1}). High-resolution mass spectra (HRMS) were measured with an Agilent 6210 LC Time of Flight mass spectrometer in electrospray mode. Either protonated molecular ions $[M + nH]^{n+}$, sodium adducts $[M + Na]^+$ or ammonium adducts $[M + NH_4]^+$ were used for empirical formula confirmation. Optical rotations were measured with a JASCO DIP-360 digital polarimeter, and are reported in units of 10^{-1} (deg cm^2 g^{-1}).

Experimental procedures

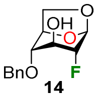
14

1,6-Anhydro-4-*O*-benzyl-2-deoxy-2-fluoro-β-D-glucopyranose (14). To a stirred solution of known compound **13**[1] (5.2 g, 22.20 mmol) in ethylene glycol (65 mL) was added KHF_2 (12.1 g, 155.4 mmol, 7 equiv.). The mixture was heated under reflux (~200 °C) for 2.5 h. After cooling to room temperature, the reaction was quenched with an aqueous 5% K_2CO_3 solution (200 mL) and stirred for 5 min. The mixture was then extracted with $CHCl_3$ (5 × 300 mL), and the combined organic phases were dried over $MgSO_4$, filtered, and concentrated under reduced pressure. The crude oil was purified by flash column chromatography (silica gel, acetone/$CHCl_3$, 1:19 → 1:9) to give **14** as a pale yellow amorphous solid (4.13 g, 16.24 mmol, 73% yield). $R_f = 0.47$ (silica, acetone/$CHCl_3$, 1:19); The spectroscopic data derived from compound **15** match those reported in the literature.

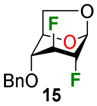
15

1,6-Anhydro-4-*O*-benzyl-2,3-dideoxy-2,3-difluoro-β-D-glucopyranose (15). To a stirred solution of compound **14** (2.94 g, 11.56 mmol) in THF (22 mL) was added a 50% DeoxoFluor solution in THF (9.84 mL, 23.13 mmol, 2 equiv.). The mixture was irradiated in a microwave reactor at 100 °C for 1.5 h. The mixture was cooled down to room temperature and quenched with water (30 mL). The mixture was extracted with CH_2Cl_2 (3 × 20 mL) and the combined organic phases were successively washed with a saturated aqueous $NaHCO_3$ solution (30 mL) and brine (30 mL). The organic solution was dried over $MgSO_4$, filtered, and concentrated under reduced pressure. The resulting crude residue was purified by flash column chromatography (silica gel, Et_2O/CH_2Cl_2, 1:19) to give **15** as a pale yellow oil (1.51 g, 5.76 mmol, 87% yield). The spectroscopic data derived from compound **15** match those reported in the literature.

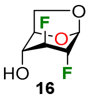

16

1,6-Anhydro-2,3-dideoxy-2,3-difluoro-β-D-glucopyranose (16). To a stirred solution of compound **15** (1.54 g, 6.00 mmol) in CH_2Cl_2 (10 mL) at 0 °C, was added a 1M $TiCl_4$ solution in CH_2Cl_2 (6.60 mL, 6.60 mmol, 1.1 equiv.). The mixture was stirred at 0 °C for 30 min and then quenched with water (20 mL). The mixture was extracted with EtOAc (3 × 20 mL), and the combined organic phases were successively washed with water (50 mL) and brine (50 mL). The organic solution was dried over $MgSO_4$, filtered, and concentrated under reduced pressure. The resulting crude was purified by flash column chromatography (silica gel, EtOAc/ hexanes, 2:3) to give **16** as a white amorphous solid (658 mg, 3.96 mmol, 66% yield). R_f = 0.34 (silica, EtOAc/hexanes, 2:3); $[\alpha]_D^{25}$ = −27.5 (*c* 0.3, MeOH); IR (ATR, ZnSe) ν 3287, 2919, 1342, 1112, 1016, 998, 864 cm^{-1}; ^{1}H NMR (500 MHz, $CDCl_3$) δ 5.57 (q, $^3J_{H1\text{-}H2} = {}^3J_{H1\text{-}F2} = {}^4J_{H1\text{-}F3}$ = 1.9 Hz, 1H, H1), 4.69 (ddp, $^2J_{H3\text{-}F3}$ = 43.0 Hz, $^3J_{H3\text{-}F2}$ = 12.2 Hz, $^3J_{H3\text{-}H2} = {}^3J_{H3\text{-}H4} = {}^4J_{H3\text{-}OH} = {}^4J_{H3\text{-}H5}$ = 1.8 Hz, 1H, H3), 4.63 (m, 1H, H5), 4.43 (ddqd, $^2J_{H2\text{-}F2}$ = 44.1 Hz, $^3J_{H2\text{-}F3}$ = 12.4 Hz, $^3J_{H2\text{-}H3} = {}^3J_{H2\text{-}H1} = {}^5J_{H2\text{-}H6a}$ = 1.6 Hz, $^5J_{H2\text{-}H5}$ = 0.6 Hz, 1H, H2), 4.07 (dt, $^2J_{H6a\text{-}H6b}$ = 7.8 Hz, $^3J_{H6a\text{-}H5} = {}^5J_{H6a\text{-}H2}$ = 1.3 Hz, 1H, H6a), 3.85 (ddt, $^2J_{H6b\text{-}H6a}$ = 7.6 Hz, $^3J_{H6b\text{-}H5}$ = 5.6 Hz, $^5J_{H6b\text{-}F3} = {}^4J_{H6b\text{-}H4}$ = 1.8 Hz, 1H, H6b), 3.78 (ddq, $^3J_{H4\text{-}F3}$ = 13.0 Hz, $^3J_{H4\text{-}OH}$ = 11.2 Hz, $^3J_{H4\text{-}H5} = {}^3J_{H4\text{-}H3} = {}^4J_{H4\text{-}H6b}$ = 1.8 Hz, 1H, H4), 2.60 (dt, $^3J_{OH\text{-}H4}$ = 11.4 Hz, $^4J_{OH\text{-}F3} = {}^4J_{OH\text{-}H3}$ = 0.9 Hz, 1H, OH) ppm; ^{13}C NMR (126 MHz, $CDCl_3$) δ 98.7 (d, $^2J_{C1\text{-}F2}$ = 27.6 Hz, 1C, C1), 88.2 (dd, $^1J_{C3\text{-}F3}$ = 181.3 Hz, $^2J_{C3\text{-}F2}$ = 30.0 Hz, 1C, C3), 84.4 (dd, $^1J_{C2\text{-}F2}$ = 180.4 Hz, $^2J_{C2\text{-}F3}$ = 28.4 Hz, 1C, C2), 75.8 (1C, C5), 67.8 (dd, $^2J_{C4\text{-}F3}$ = 27.3 Hz, $^3J_{C4\text{-}F2}$ = 1.9 Hz, 1C, C4), 64.9 (d, $^4J_{C6\text{-}F3}$ = 4.3 Hz, 1C, C6) ppm; ^{19}F NMR (470 MHz, $CDCl_3$) δ −187.97 (dq, $^2J_{F3\text{-}H3}$ = 42.0 Hz, $^3J_{F3\text{-}H2} = {}^3J_{F3\text{-}F2} = {}^3J_{F3\text{-}H4}$ = 13.1 Hz, 1F, F3), −194.33 (dt, $^2J_{F2\text{-}H2}$ = 44.1 Hz, $^3J_{F2\text{-}H3} = {}^3J_{F2\text{-}F3}$ = 13.4 Hz, 1F, F2) ppm; HRMS calcd for $C_6H_8O_3F_2Na^+$ $[M + Na]^+$ 189.0328, found 189.0334.

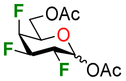

19

1,6-Di-*O*-acetyl-2,3,4-trideoxy-2,3,4-trifluoro-α-D-galactopyranose (19). To a stirred solution of compound **16** (568 mg, 3.42 mmol) in CH_2Cl_2 (30 mL) at 0 °C, were added pyridine (0.83 mL, 10.26 mmol, 3 equiv.) and Tf_2O (1.15 mL, 6.84 mmol, 2 equiv.). The mixture was stirred at 0 °C for 10 min and then quenched with water (50 mL). The mixture was extracted with CH_2Cl_2 (3 × 50 mL), and

the combined organic phases were successively washed with a saturated aqueous $NaHCO_3$ solution (100 mL), aqueous 1M HCl solution (100 mL), and brine (100 mL). The organic solution was dried over $MgSO_4$, filtered, and concentrated under reduced pressure. The crude triflate **17** was used for the next step without further purification. To a stirred solution of the crude triflate **17** in CH_2Cl_2 (30 mL) was added tetrabutylammonium fluoride trihydrate (TBAF·$3H_2O$) (1.62 g, 5.13 mmol, 1.5 equiv.). The mixture was stirred at room temperature for 18 h and formation of intermediate **18** was monitored by TLC (R_f: 0.37, EtOAc/hexanes, 2:8). The mixture cooled to 0 °C and Ac_2O (9.7 mL, 102.57 mmol, 30 equiv.) and H_2SO_4 (1.8 mL, 34.2 mmol, 10 equiv.) were added. The mixture was stirred at room temperature for 18 h, then cooled to 0 °C. Sodium acetate (5.61 g, 68.38 mmol, 20 equiv.) was added and the mixture was stirred for an additional 20 min. Water (50 mL) was added and the mixture was extracted with CH_2Cl_2 (3 × 50 mL). The combined organic phases were successively washed with a saturated aqueous $NaHCO_3$ solution (100 mL) and brine (100 mL). The organic solution was dried over $MgSO_4$, filtered, and concentrated under reduced pressure. The crude residue was purified by flash column chromatography (silica gel, acetone/toluene, 1:19) to give an anomeric mixture (α/β, 4.7:1) of **19** as a colorless thick oil (582 mg, 2.15 mmol, 63%). A second purification by flash column chromatography (silica gel, $Et_2O/CHCl_3$, 3:97 → 6:94) gave pure α anomer, suitable for characterization. R_f = 0.25 (silica, EtOAc/hexanes, 3:7), R_f = 0.29 (silica, acetone/toluene, 1:19); $[\alpha]_D^{25}$ = 18.1 (*c* 0.9, $CHCl_3$); IR (ATR, ZnSe) ν 2921, 1743, 1371, 1216, 1062, 1041, 774 cm^{-1}; ^1H NMR (500 MHz, $CDCl_3$) δ 6.48 (t, $^3J_{H1-H2}$ = $^3J_{H1-F2}$ = 4.3 Hz, 1H, H1), 5.06 (ddtd, $^2J_{H4-F4}$ = 50.1 Hz, $^3J_{H4-F3}$ = 7.5 Hz, $^3J_{H4-H3}$ = 3.5 Hz, $^3J_{H4-H5}$ = 2.7 Hz, $^4J_{H4-H6a}$ = 1.1 Hz, 1H, H4), 5.02 (ddddd, $^2J_{H2-F2}$ = 49.3 Hz, $^3J_{H2-F3}$ = 12.2 Hz, $^3J_{H2-H3}$ = 9.4 Hz, $^3J_{H2-H1}$ = 4.1 Hz, $^4J_{H2-F4}$ = 1.5 Hz, 1H, H2), 4.95 (ddddd, $^2J_{H3-F3}$ = 48.5 Hz, $^3J_{H3-F4}$ = 25.4 Hz, $^3J_{H3-F2}$ = 11.9 Hz, $^3J_{H3-H2}$ = 9.5 Hz, $^3J_{H3-H4}$ = 2.9 Hz, 1H, H3), 4.31 (ddt, $^2J_{H6a-H6b}$ = 11.4 Hz, $^3J_{H6a-H5}$ = 6.5 Hz, $^4J_{H6a-H4}$ = $^4J_{H6a-F4}$ = 1.3 Hz, 1H, H6a), 4.23 (dd, $^2J_{H6b-H6a}$ = 11.3 Hz, $^3J_{H6b-H5}$ = 6.5 Hz, 1H, H6b), 4.14 (dtdt, $^3J_{H5-F4}$ = 27.8 Hz, $^3J_{H5-H6a}$ = $^3J_{H5-H6b}$ = 6.7 Hz, $^3J_{H5-H4}$ = 1.7 Hz, $^4J_{H5-H3}$ = $^4J_{H5-F3}$ = 0.9 Hz, 1H, H5), 2.16 (s, 3H, $COCH_3$), 2.09 (s, 3H, $COCH_3$) ppm; ^{13}C NMR (126 MHz, $CDCl_3$) δ 170.5, 168.6 (2C, 2 × $COCH_3$), 89.1 (dd, $^2J_{C1-F2}$ = 22.5 Hz, $^3J_{C1-F3}$ = 9.4 Hz, 1C, C1), 87.0 (ddd, $^2J_{C4-F4}$ = 186.9 Hz, $^2J_{C4-F3}$ = 17.1 Hz, $^3J_{C4-F2}$ = 8.6 Hz, 1C, C4), 86.5 (ddd, $^1J_{C3-F3}$ = 193.2 Hz, $^2J_{C3-F2}$ = 19.3 Hz, $^2J_{C3-F4}$ = 18.0 Hz, 1C, C3), 84.8 (ddd, $^1J_{C2-F2}$ = 191.6 Hz, $^2J_{C2-F3}$ = 19.3 Hz, $^3J_{C2-F4}$ = 2.7 Hz, 1C, C2), 68.9 (dd, $^2J_{C5-F4}$ = 18.3 Hz, $^3J_{C5-F3}$ = 5.1 Hz, 1C, C5), 61.1 (dd, $^3J_{C6-F4}$ = 6.3 Hz, $^4J_{C6-F3}$ = 2.2 Hz, 1C, C6), 20.9, 20.8 (2C, 2 × $COCH_3$) ppm; ^{19}F NMR (470 MHz, $CDCl_3$) δ −206.51 (m, 1F, F3), −211.27 (dtd, $^2J_{F2-H2}$ = 49.2 Hz, $^3J_{F2-F3}$ = $^3J_{F2-H3}$ = 12.8 Hz, $^3J_{F2-H1}$ = 3.6 Hz, 1F,

F2), –220.55 (dtd, $^2J_{F4\text{-}H4}$ = 50.3 Hz, $^3J_{F4\text{-}H3}$ = $^3J_{F4\text{-}H5}$ = 27.0 Hz, $^3J_{F4\text{-}F3}$ = 14.8 Hz, 1F, F4) ppm; HRMS calcd for $C_{10}H_{17}O_5F_3N^+$ [M + NH_4]$^+$ 288.1059, found 288.1053.

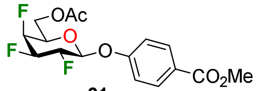

21

4-(Methoxycarbonyl)phenyl 6-*O*-acetyl-2,3,4-trifluoro-2,3,4-trideoxy-β-D-galactopyranoside (21). To a stirred solution of compound **19** (150 mg, 0.56 mmol) in CH_2Cl_2 (3 mL) at 0 °C, was added a 33 wt. % solution of HBr in AcOH (1 mL). The mixture was stirred at room temperature for 66 h and then quenched at 0 °C with a saturated aqueous $NaHCO_3$ solution (5 mL). The mixture was extracted with CH_2Cl_2 (3 × 5 mL), and the combined organic phases were successively washed with a saturated aqueous $NaHCO_3$ solution (10 mL), aqueous 1M HCl solution (10 mL), and brine (10 mL). The organic solution was dried over $MgSO_4$, filtered, and concentrated under reduced pressure. The crude bromide **20** was used for the next step without further purification. To a solution of the crude bromide **20** in EtOAc (4 mL) at room temperature were added tetrabutylammonium hydrogen sulfate (TBAHS) (188 mg, 0.555 mmol, 1 equiv.), methyl *p*-hydroxybenzoate (253 mg, 1.665 mmol, 3 equiv.), and 1M Na_2CO_3 solution (4 mL). The mixture was vigorously stirred at room temperature for 18 h. After this time, water (10 mL) was added, and the mixture was extracted with EtOAc (3 × 10 mL). The combined organic phases were successively washed with a saturated aqueous $NaHCO_3$ solution (20 mL), aqueous 1M HCl solution (20 mL), and brine (20 mL). The organic solution was dried over $MgSO_4$, filtered, and concentrated under reduced pressure. The obtained crude was purified by flash column chromatography (silica gel, acetone/toluene, 1:19) to give **21** as a colorless thick oil (120 mg, 0.331 mmol, 60% yield) and unstable trifluoro glycal **21a** as by-product (23.4 mg, 0.111 mmol, 20% yield). . R_f = 0.32 (silica, acetone/toluene, 1:9); $[\alpha]_D^{25}$ = –41.5 (*c* 0.5, $CHCl_3$); IR (ATR, ZnSe) ν 2877, 1741, 1714, 1605, 1222, 1074, 769 cm^{-1}; ^{1}H NMR (500 MHz, $CDCl_3$) δ 8.06 – 7.98 (m, 2H, Ar), 7.11 – 7.05 (m, 2H, Ar), 5.15 (dd, $^3J_{H1\text{-}H2}$ = 7.4 Hz, $^3J_{H1\text{-}F2}$ = 4.1 Hz, 1H, H1), 5.04 (ddt, $^2J_{H4\text{-}F4}$ = 50.2 Hz, $^3J_{H4\text{-}F3}$ = 6.2 Hz, $^3J_{H4\text{-}H3}$ = $^3J_{H4\text{-}H5}$ = 2.9 Hz, 1H, H4), 4.96 (ddddd, $^2J_{H2\text{-}F2}$ = 51.3 Hz, $^3J_{H2\text{-}F3}$ = 13.0 Hz, $^3J_{H2\text{-}H3}$ = 8.9 Hz, $^3J_{H2\text{-}H1}$ = 7.9 Hz, $^4J_{H2\text{-}F4}$ = 0.9 Hz, 1H, H2), 4.76 (ddddd, $^2J_{H3\text{-}F3}$ = 47.1 Hz, $^3J_{H3\text{-}F4}$ = 26.2 Hz, $^3J_{H3\text{-}F2}$ = 13.9 Hz, $^3J_{H3\text{-}H2}$ = 9.2 Hz, $^3J_{H3\text{-}H4}$ = 3.1 Hz, 1H, H3), 4.42 (dd, $^2J_{H6a\text{-}H6b}$ = 11.5 Hz, $^3J_{H6a\text{-}H5}$ = 7.1 Hz, 1H, H6a), 4.30 (dd, $^2J_{H6b\text{-}H6a}$ = 11.6 Hz, $^3J_{H6b\text{-}H5}$ = 6.0 Hz, 1H, H6b), 3.96 (dddd, $^3J_{H5\text{-}F4}$ = 25.4 Hz, $^3J_{H5\text{-}H6a}$ = 7.3 Hz, $^3J_{H5\text{-}H6b}$ = 5.6 Hz, $^3J_{H5\text{-}H4}$ = 1.8 Hz, 1H, H5), 3.90 (s, 3H, CO_2CH_3), 2.11 (s, 3H, $COCH_3$) ppm; ^{13}C NMR (126 MHz, $CDCl_3$) δ 170.5

(1C, *C*OCH$_3$), 166.6 (1C, *C*O$_2$CH$_3$), 160.1, 131.7, 125.5, 116.6 (6C, Ar), 97.8 (dd, $^2J_{C1\text{-}F2}$ = 23.8 Hz, $^3J_{C1\text{-}F3}$ = 11.0 Hz, 1C, C1), 88.9 (ddd, $^1J_{C3\text{-}F3}$ = 195.6 Hz, $^2J_{C3\text{-}F2}$ = 19.4 Hz, $^2J_{C3\text{-}F4}$ = 18.1 Hz, 1C, C3), 88.2 (dd, $^1J_{C2\text{-}F2}$ = 188.8 Hz, $^2J_{C2\text{-}F3}$ = 20.1 Hz, 1C, C2), 86.1 (ddd, $^2J_{C4\text{-}F4}$ = 188.1 Hz, $^2J_{C4\text{-}F3}$ = 17.0 Hz, $^3J_{C4\text{-}F2}$ = 9.1 Hz, 1C, C4), 70.6 (dd, $^2J_{C5\text{-}F4}$ = 18.4 Hz, $^3J_{C5\text{-}F3}$ = 5.9 Hz, 1C, C5), 61.3 (dd, $^3J_{C6\text{-}F4}$ = 5.8 Hz, $^4J_{C6\text{-}F3}$ = 2.6 Hz, 1C, C6), 52.26 (1C, CO$_2$*C*H$_3$), 20.9 (1C, CO*C*H$_3$) ppm; ^{19}F NMR (470 MHz, CDCl$_3$) δ −202.26 (dqd, $^2J_{F3\text{-}H3}$ = 47.6 Hz, $^3J_{F3\text{-}H2}$ = $^3J_{F3\text{-}F2}$ = $^3J_{F3\text{-}F4}$ = 13.9 Hz, $^3J_{F3\text{-}H4}$ = 6.5 Hz, 1F, F3), −207.94 (dtdd, $^2J_{F2\text{-}H2}$ = 51.4 Hz, $^3J_{F2\text{-}F3}$ = $^3J_{F2\text{-}H3}$ = 14.1 Hz, $^3J_{F2\text{-}H1}$ = 4.1 Hz, $^4J_{F2\text{-}F4}$ = 2.7 Hz, 1F, F2)., −217.78 (dtd, $^2J_{F4\text{-}H4}$ = 50.7 Hz, $^3J_{F4\text{-}H3}$ = $^3J_{F4\text{-}H5}$ = 25.7 Hz, $^3J_{F4\text{-}F3}$ = 15.0 Hz, 1F, F4) ppm; HRMS calcd for $C_{16}H_{18}O_6F_3^+$ $[M + H]^+$ 363.1053, found 363.1050.

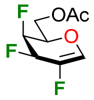

21a

6-*O*-Acetyl-2,3,4-trideoxy-2,3,4-trifluoro-D-galactal (21a). R_f = 0.26 (silica, acetone/toluene, 1:9); $[\alpha]_D^{25}$ = −6.8 (c 0.2, CHCl$_3$); ^{1}H NMR (500 MHz, CDCl$_3$) δ 6.74 (dd, $^3J_{H1\text{-}F2}$ = 4.2 Hz, $^3J_{H1\text{-}F3}$ = 3.0 Hz, 1H, H1), 5.42 – 5.27 (m, $^2J_{H3\text{-}F3}$ = 52.6 Hz, $^3J_{H3\text{-}F2}$ = 12.7 Hz, 1H, H3), 5.00 (ddddd, $^2J_{H4\text{-}F4}$ = 47.3 Hz, $^3J_{H4\text{-}F3}$ = 9.4 Hz, $^3J_{H4\text{-}H3}$ = 4.5 Hz, $^3J_{H4\text{-}H5}$ = 3.4 Hz, $^4J_{H4\text{-}F2}$ = 2.7 Hz, 1H, H4), 4.50 – 4.44 (m, 1H, H6a), 4.32 – 4.23 (m, 2H, H5, H6b), 2.09 (s, 3H, COC*H*$_3$) ppm; ^{13}C NMR (126 MHz, CDCl$_3$) δ 170.6 (1C, *C*OCH$_3$), 142.1 (ddd, $^1J_{C2\text{-}F2}$ = 244.1 Hz, $^2J_{C2\text{-}F3}$ = 16.6 Hz, $^3J_{C2\text{-}F4}$ = 2.8 Hz, 1C, C2), 133.3 (dd, $^2J_{C1\text{-}F2}$ = 39.5 Hz, $^3J_{C1\text{-}F3}$ = 6.2 Hz, 1C, C1), 83.0 (ddd, $^1J_{C4\text{-}F4}$ = 192.0 Hz, $^2J_{C4\text{-}F3}$ = 16.0 Hz, $^3J_{C4\text{-}F2}$ = 7.9 Hz, 1C, C4), 79.9 (ddd, $^1J_{C3\text{-}F3}$ = 187.4 Hz, $^2J_{C3\text{-}F2}$ = 22.9 Hz, $^2J_{C3\text{-}F4}$ = 18.1 Hz, 1C, C3), 73.1 (dd, $^2J_{C5\text{-}F4}$ = 20.5 Hz, $^3J_{C5\text{-}F3}$ = 1.3 Hz, 1C, C5), 60.6 (dd, $^3J_{C6\text{-}F4}$ = 5.3 Hz, $^4J_{C6\text{-}F3}$ = 2.9 Hz, 1C, C6), 20.8 (1C, CO*C*H$_3$) ppm; ^{19}F NMR (470 MHz, CDCl$_3$) δ –170.07 – –170.22 (m, $^3J_{F2\text{-}F3}$ = 23.3 Hz, 1F, F2), –200.00 – –200.26 (m, 1H, F4), –216.07 (ddq, $^2J_{F3\text{-}H3}$ = 50.3 Hz, $^3J_{F3\text{-}F2}$ = 21.1 Hz, $^3J_{F3\text{-}H4}$ = $^3J_{F3\text{-}F4}$ = $^4J_{F3\text{-}H5}$ = 10.5 Hz, 1F, F3) ppm.

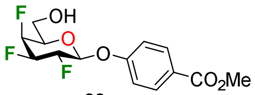

22

4-(Methoxycarbonyl)phenyl 2,3,4-trifluoro-2,3,4-trideoxy-β-D-galactopyranoside (22). To a stirred solution of compound **21** (104 mg, 0.287 mmol) in methanol (5 mL), was added dropwise a methanolic 1M NaOMe solution, until pH ≈ 9. The mixture was stirred at room temperature for 1 h and then neutralized to pH ≈ 7 with acidic resin. The mixture was filtered and concentrated under reduced pressure to afford **22** as a white amorphous solid (91 mg, 0.284 mmol, 99% yield). R_f = 0.18 (silica, EtOAc/hexanes, 1:1); $[\alpha]_D^{25}$ = –53.7 (*c* 0.23, $CHCl_3$); IR (ATR, ZnSe) ν 3309, 2914, 1705, 1605, 1101, 1035, 774 cm^{-1}; ^1H NMR (500 MHz, $CDCl_3$) δ 8.05 – 8.00 (m, 2H, Ar), 7.10 – 7.04 (m, 2H, Ar), 5.20 (dd, $^3J_{H1\text{-}H2}$ = 7.4 Hz, $^3J_{H1\text{-}F2}$ = 4.0 Hz, 1H, H1), 5.06 (ddt, $^2J_{H4\text{-}F4}$ = 50.2 Hz, $^3J_{H4\text{-}F3}$ = 6.2 Hz, $^3J_{H4\text{-}H3}$ = $^3J_{H4\text{-}H5}$ = 2.9 Hz, 1H, H4), 4.96 (ddddd, $^2J_{H2\text{-}F2}$ = 51.4 Hz, $^3J_{H2\text{-}F3}$ = 12.9 Hz, $^3J_{H2\text{-}H3}$ = 8.8 Hz, $^3J_{H2\text{-}H1}$ = 7.6 Hz, $^4J_{H2\text{-}F4}$ = 1.0 Hz, 1H, H2), 4.77 (ddddd, $^2J_{H3\text{-}F3}$ = 47.1 Hz, $^3J_{H3\text{-}F4}$ = 26.3 Hz, $^3J_{H3\text{-}F2}$ = 14.1 Hz, $^3J_{H3\text{-}H2}$ = 9.0 Hz, $^3J_{H3\text{-}H4}$ = 3.1 Hz, 1H, H3), 4.02 – 3.96 (m, 1H, H6a), 3.90 (s, 3H, CO_2CH_3), 3.88 – 3.85 (m, 1H, H6b), 3.82 (dddd, $^3J_{H5\text{-}F4}$ = 25.5 Hz, $^3J_{H5\text{-}H6a}$ = 7.3 Hz, $^3J_{H5\text{-}H6b}$ = 5.8 Hz, $^3J_{H5\text{-}H4}$ = 1.6 Hz, 1H, H5) ppm; ^{13}C NMR (126 MHz, $CDCl_3$) δ 166.6 (1C, CO_2CH_3), 160.0, 131.8, 125.4, 116.3 (6C, Ar), 97.7 (dd, $^2J_{C1\text{-}F2}$ = 23.7 Hz, $^3J_{C1\text{-}F3}$ = 10.9 Hz, 1C, C1), 89.2 (dt, $^1J_{C3\text{-}F3}$ = 195.1 Hz, $^2J_{C3\text{-}F2}$ = 19.3 Hz, $^2J_{C3\text{-}F4}$ = 18.3 Hz, 1C, C3), 88.4 (ddd, $^1J_{C2\text{-}F2}$ = 188.7 Hz, $^2J_{C2\text{-}F3}$ = 20.1 Hz, $^3J_{C2\text{-}F4}$ = 0.8 Hz, 1C, C2), 86.2 (ddd, $^2J_{C4\text{-}F4}$ = 186.9 Hz, $^2J_{C4\text{-}F3}$ = 16.7 Hz, $^3J_{C4\text{-}F2}$ = 9.1 Hz, 1C, C4), 73.4 (dd, $^2J_{C5\text{-}F4}$ = 18.3 Hz, $^3J_{C5\text{-}F3}$ = 5.1 Hz, 1C, C5), 60.6 (dd, $^3J_{C6\text{-}F4}$ = 5.5 Hz, $^4J_{C6\text{-}F3}$ = 2.5 Hz, 1C, C6), 52.3 (1C, CO_2CH_3) ppm; ^{19}F NMR (470 MHz, $CDCl_3$) δ –202.14 (dqd, $^2J_{F3\text{-}H3}$ = 47.7 Hz, $^3J_{F3\text{-}H2}$ = $^3J_{F3\text{-}F2}$ = $^3J_{F3\text{-}F4}$ = 14.0 Hz, $^3J_{F3\text{-}H4}$ = 6.5 Hz, 1F, F3), –207.95 (dtt, $^2J_{F2\text{-}H2}$ = 51.6 Hz, $^3J_{F2\text{-}F3}$ = $^3J_{F2\text{-}H3}$ = 14.1 Hz, $^3J_{F2\text{-}H1}$ = $^4J_{F2\text{-}F4}$ = 3.3 Hz, 1F, F2), –217.49 (dtd, $^2J_{F4\text{-}H4}$ = 50.9 Hz, $^3J_{F4\text{-}H3}$ = $^3J_{F4\text{-}H5}$ = 25.8 Hz, $^3J_{F4\text{-}F3}$ = 14.7 Hz, 1F, F4) ppm; HRMS calcd for $C_{14}H_{16}O_5F_3^+$ $[M + H]^+$ 321.0943, found 321.0944.

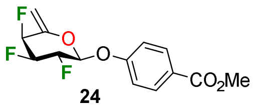

24

4-(Methoxycarbonyl)phenyl 2,3,4-trideoxy-2,3,4-trifluoro-β-*arabino*-hex-5-enopyranoside (24). To a stirred solution of compound **22** (8.9 mg, 0.0271 mmol) in CH_2Cl_2 (0.5 mL) at 0 °C, were added pyridine (11 µL, 0.136 mmol, 5 equiv.) and a 1M Tf_2O solution in CH_2Cl_2 (54 µL, 0.054 mmol, 2 equiv.).

The mixture was stirred at 0 °C for 15 min and then quenched with water (5 mL). The mixture was extracted with CH_2Cl_2 (3 × 5 mL), and the combined organic phases were successively washed with a saturated aqueous $NaHCO_3$ solution (10 mL), aqueous 1M HCl solution (10 mL), and brine (10 mL). The organic solution was dried over $MgSO_4$, filtered, and concentrated under reduced pressure. The crude triflate **23** was used for the next step without further purification and was dissolved in a dry 1M TBAF solution in THF (0.5 mL, 0.5 mmol, 18 equiv.). The mixture was stirred at room temperature for 1 h and then quenched with water (5 mL). The mixture was extracted with CH_2Cl_2 (3 × 5 mL), and the combined organic phases were successively washed with a saturated aqueous $NaHCO_3$ solution (10 mL), and brine (10 mL). The organic solution was dried over $MgSO_4$, filtered, and concentrated under reduced pressure. The obtained crude was purified by flash column chromatography (silica gel, toluene/CH_2Cl_2, 1:1) to give **24** as a colorless thick oil (6.0 mg, 0.020 mmol, 73% yield) and **25** as a white amorphous solid (0.5 mg, 0.002 mmol, 7% yield). Compound **24**: R_f = 0.33 (silica, toluene/CH_2Cl_2, 1:1); $[\alpha]_D^{25}$ = −14.5 (*c* 1.0, $CHCl_3$); IR (ATR, ZnSe) ν 2955, 1715, 1606, 1227, 1103, 1026, 769 cm^{-1}; ^{1}H NMR (500 MHz, $CDCl_3$) δ 8.05 – 8.01 (m, 2H, Ar), 7.15 – 7.10 (m, 2H, Ar), 5.55 (dd, $^3J_{H1-F2}$ = 10.1 Hz, $^3J_{H1-H2}$ = 4.0 Hz, 1H, H1), 5.26 (ddt, $^2J_{H4-F4}$ = 49.1 Hz, $^3J_{H4-F3}$ = 14.2 Hz, $^3J_{H4-H3}$ = $^3J_{H4-H5}$ = 3.2 Hz, 1H, H4), 5.12 (ddddd, $^2J_{H2-F2}$ = 48.5 Hz, $^3J_{H2-F3}$ = 11.1 Hz, $^3J_{H2-H3}$ = 6.8 Hz, $^3J_{H2-H1}$ = 3.8 Hz, $^4J_{H2-F4}$ = 2.1 Hz, 1H, H2), 4.98 (d, $^2J_{H6a-H6b}$ = 1.9 Hz, 1H, H6a), 4.94 (ddddd, $^2J_{H3-F3}$ = 48.4 Hz, $^3J_{H3-F4}$ = 19.0 Hz, $^3J_{H3-F2}$ = 11.2 Hz, $^3J_{H3-H2}$ = 7.0 Hz, $^3J_{H3-H4}$ = 3.0 Hz, 1H, H3), 4.93 (t, $^2J_{H6b-H6a}$ = $^4J_{H6b-F4}$ = 2.2 Hz, 1H, H6b), 3.90 (s, 3H, CO_2CH_3) ppm; ^{13}C NMR (126 MHz, $CDCl_3$) δ 166.6 (1C, CO_2CH_3), 159.5 (1C, Ar), 148.2 (dd, $^2J_{C5-F4}$ = 18.5 Hz, $^3J_{C5-F3}$ = 5.6 Hz, 1C, C5), 131.8, 125.3, 116.4 (5C, Ar), 103.5 (d, $^3J_{C6-F4}$ = 5.9 Hz, 1C, C6), 97.4 (dd, $^2J_{C1-F2}$ = 30.9 Hz, $^3J_{C1-F3}$ = 6.0 Hz, 1C, C1), 88.0 (ddd, $^1J_{C2-F2}$ = 182.2 Hz, $^2J_{C2-F3}$ = 23.5 Hz, $^3J_{C2-F4}$ = 4.1 Hz, 1C, C2), 86.9 (ddd, $^1J_{C3-F3}$ = 193.4 Hz, $^2J_{C3-F2}$ = 24.0 Hz, $^2J_{C3-F4}$ = 18.6 Hz, 1C, C3), 85.0 (dddd, $^1J_{C4-F4}$ = 185.8 Hz, $^2J_{C4-F3}$ = 18.9 Hz, $^3J_{C4-F2}$ = 6.0, 1C, C4), 52.2 (1C, CO_2CH_3) ppm; ^{19}F NMR (470 MHz, $CDCl_3$) δ −196.33 (dt, $^2J_{F4-H4}$ = 48.9 Hz, $^3J_{F4-H3}$ = $^3J_{F4-F3}$ = 16.8 Hz, 1F, F4), −203.32 (ddt, $^2J_{F2-H2}$ = 50.9 Hz, $^3J_{F2-F3}$ = 16.0 Hz, $^3J_{F2-H1}$ = $^3J_{F2-H3}$ = 10.2 Hz, 1F, F2), −205.77 (dqd, $^2J_{F3-H3}$ = 48.5 Hz, $^3J_{F3-F2}$ = $^3J_{F3-H4}$ = $^3J_{F3-F4}$ = 14.5 Hz, $^3J_{F3-H2}$ = 10.8 Hz, 1F, F3) ppm; HRMS calcd for $C_{14}H_{13}O_4F_3Na^+$ $[M + Na]^+$ 325.0659, found 325.0658.

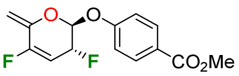

25

(2R,3R)-3,5-difluoro-2-[4-(Methoxycarbonyl)phenyl]-6-methylene-3,6-dihydro-2H-pyran (25). Compound **24** (4.6 mg, 0.015 mmol) was dissolved in a dry 1M TBAF solution in THF (0.5 mL, 0.5 mmol, 33 equiv.). The mixture was stirred at room temperature for 3 days and then quenched with water (5 mL). The mixture was extracted with CH_2Cl_2 (3 × 5 mL), and the combined organic phases were successively washed with a saturated aqueous $NaHCO_3$ solution (10 mL), and brine (10 mL). The organic solution was dried over $MgSO_4$, filtered, and concentrated under reduced pressure. The obtained crude was purified by flash column chromatography (silica gel, toluene/CH_2Cl_2, 1:1) to give **25** as a white amorphous solid (3.7 mg, 0.013 mmol, 86% yield). R_f = 0.38 (silica, toluene/CH_2Cl_2, 1:1); $[\alpha]_D^{25} = -176.5$ (*c* 0.17, $CHCl_3$); IR (ATR, ZnSe) ν 2924, 2854, 1713, 1625, 1279, 1014, 769 cm^{-1}; ^{1}H NMR (500 MHz, $CDCl_3$) δ 8.04 – 8.00 (m, 2H, Ar), 7.13 – 7.10 (m, 2H, Ar), 5.86 (dtt, $^{3}J_{H1\text{-}F2}$ = 8.4 Hz, $^{3}J_{H1\text{-}H2}$ = $^{5}J_{H1\text{-}H6b}$ = 1.6 Hz, $^{4}J_{H1\text{-}H3}$ = $^{5}J_{H1\text{-}H6a}$ = 0.8 Hz, 1H, H1), 5.71 (ddddt, $^{3}J_{H3\text{-}F4}$ = 12.3 Hz, $^{3}J_{H3\text{-}H2}$ = 6.3Hz, $^{3}J_{H3\text{-}F2}$ = 2.7 Hz, $^{5}J_{H3\text{-}H6b}$ = 1.8 Hz, $^{4}J_{H3\text{-}H1}$ = $^{5}J_{H3\text{-}H6a}$ = 0.9 Hz, 1H, H3), 5.24 (dddd, $^{2}J_{H2\text{-}F2}$ = 48.3 Hz, $^{3}J_{H2\text{-}H3}$ = 6.2 Hz, $^{4}J_{H2\text{-}F4}$ = 4.0 Hz, $^{3}J_{H2\text{-}H1}$ = 1.4 Hz, 1H, H2), 4.98 (tdt, $^{2}J_{H6a\text{-}H6b}$ = $^{4}J_{H6a\text{-}F4}$ = 3.1 Hz, $^{6}J_{H6a\text{-}F2}$ = 2.3 Hz, $^{5}J_{H6a\text{-}H3}$ = $^{5}J_{H6a\text{-}H1}$ = 0.8 Hz, 1H, H6a), 4.84 (tt, $^{2}J_{H6b\text{-}H6a}$ = $^{4}J_{H6b\text{-}F4}$ = 3.6 Hz, $^{5}J_{H6b\text{-}H3}$ = $^{5}J_{H6b\text{-}H1}$ = 2.0 Hz, 1H, H6b), 3.90 (s, 3H, CO_2CH_3) ppm; ^{13}C NMR (126 MHz, $CDCl_3$) δ 166.6 (1C, CO_2CH_3), 159.6 (1C, Ar), 156.6 (dd, $^{1}J_{C4\text{-}F4}$ = 263.4 Hz, $^{3}J_{C4\text{-}F2}$ = 13.2 Hz, 1C, C4), 143.6 (dd, $^{2}J_{C5\text{-}F4}$ = 31.7 Hz, $^{4}J_{C5\text{-}F2}$ = 6.7 Hz, 1C, C5), 131.8, 125.1, 116.1 (5C, Ar), 97.8 (t, $^{2}J_{C3\text{-}F2}$ = $^{2}J_{C3\text{-}F4}$ = 16.8 Hz, 1C, C3), 97.6 (d, $^{3}J_{C6\text{-}F4}$ = 5.0 Hz, 1C, C6), 94.8 (dd, $^{2}J_{C1\text{-}F2}$ = 33.8 Hz, $^{4}J_{C1\text{-}F4}$ = 1.4 Hz, 1C, C1), 83.8 (dd, $^{1}J_{C2\text{-}F2}$ = 173.8 Hz, $^{3}J_{C2\text{-}F4}$ = 13.1 Hz, 1C, C2), 52.2 (1C, CO_2CH_3) ppm; ^{19}F NMR (470 MHz, $CDCl_3$) δ –119.54 (dh, $^{3}J_{F4\text{-}H3}$ = 11.5 Hz, $^{4}J_{F4\text{-}F2}$ = $^{4}J_{F4\text{-}H2}$ = $^{4}J_{F4\text{-}H6a}$ = $^{4}J_{F4\text{-}H6b}$ = $^{5}J_{F4\text{-}H1}$ = 3.5 Hz, 1F, F4), –170.68 (dddt, $^{2}J_{F2\text{-}H2}$ = 48.8 Hz, $^{3}J_{F2\text{-}H1}$ = 9.6 Hz, $^{3}J_{F2\text{-}H3}$ = 6.3 Hz, $^{4}J_{F2\text{-}F4}$ = $^{6}J_{F2\text{-}H6a}$ = 3.2 Hz, 1F, F2) ppm; HRMS calcd for $C_{14}H_{13}O_4F_2^+$ $[M + H]^+$ 283.0782, found 283.0776.

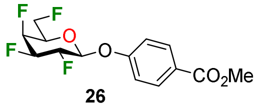

26

4-(Methoxycarbonyl)phenyl 2,3,4,6-tetrafluoro-2,3,4,6-tetradeoxy-β-D-galactopyranoside (26). To a stirred solution of compound **22** (51 mg, 0.159 mmol) in CH_2Cl_2 (2.5 mL) were added 2,4,6-collidine

(0.126 mL, 0.956 mmol, 6 equiv.) and diethylaminosulfur trifluoride (DAST) (0.059 mL, 0.478 mmol, 3 equiv.). The mixture was irradiated in a microwave reactor at 100 °C for 1 h. After cooling, the reaction was quenched with water (5 mL). The mixture was extracted with CH_2Cl_2 (3 × 5 mL). The combined organic phases were successively washed with a saturated aqueous $NaHCO_3$ solution (10 mL) and brine (10 mL). The organic solution was dried over $MgSO_4$, filtered, and concentrated under reduced pressure. The resulting crude was purified by flash column chromatography (silica gel, toluene/CH_2Cl_2, 1:1) to give **26** as a white amorphous solid (25 mg, 0.0766 mmol, 48% yield), along with compound **24** (23.2 mg, 0.077 mmol, 48%). R_f = 0.13 (silica, toluene/CH_2Cl_2, 1:1); $[\alpha]_D^{25}$ = −1.4 (*c* 0.1, MeOH); IR (ATR, ZnSe) ν 2957, 2885, 1699, 1609, 1462, 1038, 772 cm^{-1}; ^{1}H NMR (500 MHz, $CDCl_3$) δ 8.05 – 7.99 (m, 2H, Ar), 7.11 – 7.06 (m, 1H, Ar), 5.20 (dd, $^3J_{H1\text{-}H2}$ = 7.3 Hz, $^3J_{H1\text{-}F2}$ = 4.2 Hz, 1H, H1), 5.08 (ddt, $^2J_{H4\text{-}F4}$ = 49.8 Hz, $^3J_{H4\text{-}F3}$ = 6.2 Hz, $^3J_{H4\text{-}H5}$ = $^3J_{H4\text{-}H3}$ = 2.9 Hz, 1H, H4), 4.97 (ddddd, $^2J_{H2\text{-}F2}$ = 51.0 Hz, $^3J_{H2\text{-}F3}$ = 12.9 Hz, $^3J_{H2\text{-}H3}$ = 8.9 Hz, $^3J_{H2\text{-}H1}$ = 7.7 Hz, $^3J_{H2\text{-}F4}$ = 1.2 Hz, 1H, H2), 4.79 (ddddd, $^2J_{H3\text{-}F3}$ = 47.0 Hz, $^3J_{H3\text{-}F4}$ = 26.3 Hz, $^3J_{H3\text{-}F2}$ = 13.9 Hz, $^3J_{H3\text{-}H2}$ = 9.2 Hz, $^3J_{H3\text{-}H4}$ = 3.1 Hz, 1H, H3), 4.67 (dd, $^2J_{H6\text{-}F6}$ = 46.1 Hz, $^3J_{H6\text{-}H5}$ = 6.4 Hz, 2H, 2 × H6), 4.02 (ddtd, $^3J_{H5\text{-}F4}$ = 25.3 Hz, $^3J_{H5\text{-}F6}$ = 10.1 Hz, $^3J_{H5\text{-}H6a}$ = $^3J_{H5\text{-}H6b}$ = 6.4 Hz, $^3J_{H5\text{-}H4}$ = 1.9 Hz, 1H, H5), 3.90 (s, 3H, CO_2CH_3) ppm; ^{13}C NMR (126 MHz, $CDCl_3$) δ 166.6 (1C, CO_2CH_3), 160.0, 131.8, 125.5, 116.5 (6C, Ar), 97.9 (dd, $^2J_{C1\text{-}F2}$ = 24.1 Hz, $^3J_{C1\text{-}F3}$ = 11.0 Hz, 1C, C1), 88.8 (dt, $^1J_{C3\text{-}F3}$ = 195.3 Hz, $^2J_{C3\text{-}F2}$ = $^2J_{C3\text{-}F4}$ = 18.9 Hz, 1C, C3), 88.2 (ddd, $^1J_{C2\text{-}F2}$ = 188.8 Hz, $^2J_{C2\text{-}F3}$ = 20.1 Hz, $^3J_{C2\text{-}F4}$ = 1.1 Hz, 1C, C2), 85.7 (dddd, $^2J_{C4\text{-}F4}$ = 187.7 Hz, $^2J_{C4\text{-}F3}$ = 17.0 Hz, $^3J_{C4\text{-}F2}$ = 9.1 Hz, $^3J_{C4\text{-}F6}$ = 5.1 Hz, 1C, C4), 79.7 (ddd, $^1J_{C6\text{-}F6}$ = 171.4 Hz, $^3J_{C6\text{-}F4}$ = 5.8 Hz, $^4J_{C6\text{-}F3}$ = 2.5 Hz, 1C, C6), 71.1 (ddd, $^2J_{C5\text{-}F6}$ = 24.6 Hz, $^2J_{C5\text{-}F4}$ = 18.3 Hz, $^3J_{C5\text{-}F3}$ = 5.8 Hz, 1C, C5), 52.2 (1C, CO_2CH_3) ppm; ^{19}F NMR (470 MHz, $CDCl_3$) δ –202.32 (dqd, $^2J_{F3\text{-}H3}$ = 47.4 Hz, $^3J_{F3\text{-}H2}$ = $^3J_{F3\text{-}F2}$ = $^3J_{F3\text{-}F4}$ = 13.8 Hz, $^3J_{F3\text{-}H4}$ = 6.3 Hz, 1F, F3), –207.85 (dtdd, $^2J_{F2\text{-}H2}$ = 51.3 Hz, $^3J_{F2\text{-}F3}$ = $^3J_{F2\text{-}H3}$ = 14.0 Hz, $^3J_{F2\text{-}H1}$ = 4.2 Hz, $^4J_{F2\text{-}F4}$ = 2.7 Hz, 1F, F2), –218.08 (dtd, $^2J_{F4\text{-}H4}$ = 51.1 Hz, $^3J_{F4\text{-}H3}$ = $^3J_{F4\text{-}H5}$ = 26.0 Hz, $^3J_{F4\text{-}F3}$ = 14.9 Hz, 1F, F4), –231.70 (td, $^2J_{F6\text{-}H6a}$ = $^2J_{F6\text{-}H6b}$ = 45.7 Hz, $^3J_{F6\text{-}H5}$ = 9.8 Hz, 1F, F6) ppm; HRMS calcd for $C_{14}H_{15}O_4F_4^+$ $[M + H]^+$ 323.0904, found 323.0901.

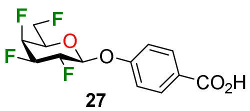

27

4-Carboxyphenyl 2,3,4,6-tetrafluoro-2,3,4,6-tetradeoxy-β-D-galactopyranoside (27). To a stirred solution of compound **26** (19 mg, 0.0590 mmol) in H_2O/MeOH/THF (2:3:5) (1.3 mL) was added an

aqueous 1M LiOH solution (0.207 mL, 0.207 mmol, 3.5 equiv.). The mixture was stirred at room temperature for 3 h and then neutralized to pH ≈ 7 with acidic resin, filtered and concentrated under reduced pressure. The resulting crude was recrystallized from acetone/heptane to give **27** as colorless crystals (18 mg, 0.0572 mmol, 97% yield). R_f = 0.61 (silica, MeOH/CH_2Cl_2, 1:9); m.p. = 224 – 225 °C; $[\alpha]_D^{25}$ = −30.1 (*c* 0.1, acetone); IR (ATR, ZnSe) ν 3087, 2957, 1679, 1606, 1456, 1044, 785 cm^{-1}; ^1H NMR (500 MHz, Acetone-d_6) δ 8.05 – 8.01 (m, 2H, Ar), 7.25 – 7.20 (m, 2H, Ar), 5.70 (dd, $^3J_{H1-H2}$ = 7.5 Hz, $^3J_{H1-F2}$ = 3.8 Hz, 1H, H1), 5.35 (ddt, $^2J_{H4-F4}$ = 51.0 Hz, $^3J_{H4-F3}$ = 6.3 Hz, $^3J_{H4-H5}$ = $^3J_{H4-H3}$ = 2.9 Hz, 1H, H4), 5.24 (ddddd, $^2J_{H3-F3}$ = 47.1 Hz, $^3J_{H3-F4}$ = 27.0 Hz, $^3J_{H3-F2}$ = 14.1 Hz, $^3J_{H3-H2}$ = 9.2 Hz, $^3J_{H3-H4}$ = 3.1 Hz, 1H, H3), 4.90 (ddddd, $^2J_{H2-F2}$ = 52.3 Hz, $^3J_{H2-F3}$ = 13.1 Hz, $^3J_{H2-H3}$ = 8.9 Hz, $^3J_{H2-H1}$ = 7.9 Hz, $^3J_{H2-F4}$ = 1.0 Hz, 1H, H2), 4.77 (ddd, $^2J_{H6a-F6}$ = 45.7 Hz, $^2J_{H6a-H6b}$ = 9.7 Hz, $^3J_{H6a-H5}$ = 5.0 Hz, 1H, H6a), 4.67 (ddd, $^2J_{H6b-F6}$ = 47.2 Hz, $^2J_{H6b-H6a}$ = 9.7 Hz, $^3J_{H6a-H5}$ = 7.0 Hz, 1H, H6b), 4.52 (ddddd, $^3J_{H5-F4}$ = 27.1 Hz, $^3J_{H5-F6}$ = 12.9 Hz, $^3J_{H5-H6b}$ = 6.8 Hz, $^3J_{H5-H6a}$ = 5.0 Hz, $^3J_{H5-H4}$ = 1.8 Hz, 1H, H5) ppm; ^{13}C NMR (126 MHz, Acetone-d_6) δ 166.8 (1C, *C*O_2CH_3), 160.6, 132.2, 125.8, 116.5 (6C, Ar), 97.3 (dd, $^2J_{C1-F2}$ = 23.4 Hz, $^3J_{C1-F3}$ = 11.3 Hz, 1C, C1), 90.3 – 88.3 (m, 2C, C2, C3), 87.2 (dddd, $^2J_{C4-F4}$ = 184.0 Hz, $^2J_{C4-F3}$ = 16.5 Hz, $^3J_{C4-F2}$ = 9.5 Hz, $^3J_{C4-F6}$ = 6.9 Hz, 1C, C4), 80.9 (ddd, $^1J_{C6-F6}$ = 170.1 Hz, $^3J_{C6-F4}$ = 5.2 Hz, $^4J_{C6-F3}$ = 2.6 Hz, 1C, C6), 71.5 (ddd, $^2J_{C5-F6}$ = 23.8 Hz, $^2J_{C5-F4}$ = 17.8 Hz, $^3J_{C5-F3}$ = 6.2 Hz, 1C, C5) ppm; ^{19}F NMR (470 MHz, Acetone-d_6) δ –201.83 (dqd, $^2J_{F3-H3}$ = 47.4 Hz, $^3J_{F3-H2}$ = $^3J_{F3-F2}$ = $^3J_{F3-F4}$ = 13.7 Hz, $^3J_{F3-H4}$ = 6.7 Hz, 1F, F3), –207.14 (dtt, $^2J_{F2-H2}$ = 52.3 Hz, $^3J_{F2-F3}$ = $^3J_{F2-H3}$ = 14.1 Hz, $^3J_{F2-H1}$ = $^4J_{F2-F4}$ = 3.3 Hz, 1F, F2), –217.20 (dtd, $^2J_{F4-H4}$ = 51.1 Hz, $^3J_{F4-H3}$ = $^3J_{F4-H5}$ = 27.0 Hz, $^3J_{F4-F3}$ = 14.1 Hz, 1F, F4), –230.43 (td, $^2J_{F6-H6a}$ = $^2J_{F6-H6b}$ = 46.4 Hz, $^3J_{F6-H5}$ = 12.8 Hz, 1F, F6) ppm; HRMS calcd for $C_{13}H_{12}O_4F_4Na^+$ $[M + Na]^+$ 331.0566, found 331.0564.

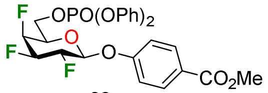

28

4-(Methoxycarbonyl)phenyl 2,3,4-trideoxy-2,3,4-trifluoro-6-((diphenoxyphosphoryl)oxy)-β-D-galactopyranoside (28). To a stirred solution of compound **22** (17 mg, 0.053 mmol) in CH_2Cl_2 (1 mL) were added $ClPO(OPh)_2$ (16.5 µL, 0.0787 mmol, 1.5 equiv.) and DMAP (9.6 mg, 0.0787 mmol, 1.5 equiv.). The mixture was stirred at room temperature for 16 h and then quenched with water (3 mL), and the mixture was extracted with CH_2Cl_2 (3 × 2 mL). The combined organic phases were washed with a saturated aqueous $NaHCO_3$ solution (10 mL), dried over $MgSO_4$, filtered, and concentrated under

reduced pressure. The obtained crude was purified by flash column chromatography (silica gel, EtOAc/hexanes, 2:3) to give **28** as a white amorphous solid (25 mg, 0.044 mmol, 84% yield). R_f = 0.32 (silica, EtOAc/hexanes, 2:3); $[\alpha]_D^{25}$ = –39.9 (*c* 0.9, $CHCl_3$); IR (ATR, ZnSe) ν 2922, 2851, 1717, 1489, 1279, 1047, 947 cm^{-1}; ^1H NMR (500 MHz, $CDCl_3$) δ 7.98 – 7.95 (m, 2H, Ar), 7.37 – 7.31 (m, 4H, Ar), 7.24 – 7.18 (m, 6H, Ar), 7.07 – 7.04 (m, 2H, Ar), 5.14 (dd, $^3J_{H1-H2}$ = 7.4 Hz, $^3J_{H1-F2}$ = 4.2 Hz, 1H, H1), 5.02 – 4.85 (m, 2H, H2, H4), 4.69 (ddddd, $^2J_{H3-F3}$ = 47.0 Hz, $^3J_{H3-F4}$ = 26.2 Hz, $^3J_{H3-F2}$ = 14.0 Hz, $^3J_{H3-H2}$ = 9.1 Hz, $^3J_{H3-H4}$ = 3.1 Hz, 1H, H3), 4.52 – 4.41 (m, 2H, H6a, H6b), 3.98 (dtd, $^3J_{H5-F4}$ = 24.9 Hz, $^3J_{H5-H6a}$ = $^3J_{H5-H6b}$ = 6.7 Hz, $^3J_{H5-H4}$ = 1.8 Hz, 1H, H5), 3.90 (s, 3H, CO_2CH_3) ppm; ^{13}C NMR (126 MHz, $CDCl_3$) δ 166.5 (1C, CO_2CH_3), 159.9 (1C, Ar), 150.33 (d, $^2J_{C-P}$ = 5.4 Hz, 1C, Ar), 150.27 (d, $^2J_{C-P}$ = 5.1 Hz, 1C, Ar), 131.8, 130.1 (4C, Ar), 126.0 (d, $^4J_{C-P}$ = 1.2 Hz, 1C, Ar), 125.9 (d, $^4J_{C-P}$ = 1.1 Hz, 1C, Ar), 125.53 (2C, Ar), 120.12 (d, $^3J_{C-P}$ = 5.0 Hz, 1C, Ar), 120.07 (d, $^3J_{C-P}$ = 5.1 Hz, 1C, Ar), 116.5 (2C, Ar), 97.8 (dd, $^2J_{C1-F2}$ = 23.7 Hz, $^3J_{C1-F3}$ = 11.2 Hz, 1C, C1), 88.8 (ddd, $^1J_{C3-F3}$ = 195.3 Hz, $^2J_{C3-F2}$ = 19.6 Hz, $^2J_{C3-F4}$ = 17.2 Hz, 1C, C3), 88.1 (dd, $^1J_{C2-F2}$ = 189.1 Hz, $^2J_{C2-F3}$ = 19.4 Hz, 1C, C2), 85.6 (ddd, $^1J_{C4-F4}$ = 187.6 Hz, $^2J_{C4-F3}$ = 17.2 Hz, $^3J_{C4-F2}$ = 8.9 Hz, 1C, C4), 71.20 (dd, $^2J_{C5-F4}$ = 11.7 Hz, $^3J_{C5-F3}$ = 5.7 Hz, 1C, C5), 65.01 (td, $^3J_{C6-F4}$ = $^2J_{C6-P}$ = 6.0 Hz, $^4J_{C6-F3}$ = 2.8 Hz, 1C, C6), 52.23 (1C, CO_2CH_3) ppm; ^{19}F NMR (470 MHz, $CDCl_3$) δ –201.35 (dqd, $^2J_{F3-H3}$ = 47.7 Hz, $^3J_{F3-H2}$ = $^3J_{F3-F2}$ = $^3J_{F3-F4}$ = 14.0 Hz, $^3J_{F4-H3}$ = 6.5 Hz, 1F, F3), –208.13 (dtt, $^2J_{F2-H2}$ = 51.3 Hz, $^3J_{F2-F3}$ = $^3J_{F2-H3}$ = 13.6 Hz, $^3J_{F2-H1}$ = $^4J_{F2-F4}$ = 3.2 Hz, 1F, F2), –217.59 (dtd, $^2J_{F4-H4}$ = 51.0 Hz, $^3J_{F4-H3}$ = $^3J_{F4-H5}$ = 25.7 Hz, $^3J_{F4-F3}$ = 15.1 Hz, 1F, F4) ppm; ^{31}P NMR (202 MHz, Chloroform-*d*) δ –12.05 ppm; HRMS calcd for $C_{26}H_{25}O_8F_3P^+$ $[M + H]^+$ 553.1234, found 553.1249.

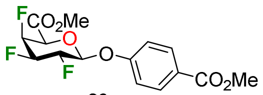

29

4-(Methoxycarbonyl)phenyl 2,3,4-trifluoro-2,3,4-trideoxy-β-D-galacturonic acid methyl ester (29). To a stirred solution of compound **22** (12 mg, 0.0359 mmol) in CH_2Cl_2/H_2O (3:1) (0.6 mL) were added TEMPO (1 mg, 0.0072 mmol, 0.2 equiv.) and BAIB (29 mg, 0.0898 mmol, 2.5 equiv.). The mixture was stirred at room temperature for 1 h and then quenched with an aqueous 1M Na_2SO_3 solution (1.5 mL) and an aqueous 1M HCl solution (~2 mL) until pH ≈ 2. The mixture was extracted with CH_2Cl_2 (5 × 5 mL) and EtOAc (5 × 5 mL). The combined organic phases were dried over $MgSO_4$, filtered and concentrated under reduced pressure. The crude carboxylic acid was used for the next step without further purification. To a stirred solution of the carboxylic acid in CH_3CN (0.6 mL) were added K_2CO_3 (6 mg,

0.0395 mmol, 1.1 equiv.) and MeI (88.6 µL, 1.436 mmol, 40 equiv.). The mixture was stirred at room temperature for 18 h and then concentrated under reduced pressure. The obtained crude was purified by flash column chromatography (silica gel, EtOAc/hexanes, 1:4 → 1:1) to give **29** as a white amorphous solid (11 mg, 0.032 mmol, 88% yield over 2 steps). R_f = 0.24 (silica, EtOAc/hexanes, 2:3); $[\alpha]_D^{25}$ = –83.9 (*c* 0.3, $CHCl_3$); IR (ATR, ZnSe) ν 2955, 1755, 1718, 1609, 1229, 1074, 768 cm^{-1}; ^{1}H NMR (500 MHz, $CDCl_3$) δ 8.05 – 7.98 (m, 2H, Ar), 7.15 – 7.09 (m, 2H, Ar), 5.39 (ddtd, $^2J_{H4-F4}$ = 49.0 Hz, $^3J_{H4-F3}$ = 6.0 Hz, $^3J_{H4-H5}$ = $^3J_{H4-H3}$ = 2.9 Hz, $^4J_{H4-F2}$ = 0.9 Hz, 1H, H4), 5.18 (ddd, $^3J_{H1-H2}$ = 7.6 Hz, $^3J_{H1-F2}$ = 4.2 Hz, $^4J_{H1-F3}$ = 1.0 Hz, 1H, H1), 4.99 (ddddd, $^2J_{H2-F2}$ = 51.2 Hz, $^3J_{H2-F3}$ = 13.0 Hz, $^3J_{H2-H3}$ = 8.9 Hz, $^3J_{H2-H1}$ = 7.8 Hz, $^4J_{H2-F4}$ = 1.2 Hz, 1H, H2), 4.82 (ddddd, $^2J_{H3-F3}$ = 47.0 Hz, $^3J_{H3-F4}$ = 26.1 Hz, $^3J_{H3-F2}$ = 14.0 Hz, $^3J_{H3-H2}$ = 9.1 Hz, $^3J_{H3-H4}$ = 3.0 Hz, 1H, H3), 4.36 (ddd, $^3J_{H5-F4}$ = 28.0 Hz, $^3J_{H3-H4}$ = 2.0 Hz, $^4J_{H3-F3}$ = 0.9 Hz, 1H, H5), 3.90 (s, 3H, CO_2CH_3), 3.87 (s, 3H, CO_2CH_3) ppm; ^{13}C NMR (126 MHz, $CDCl_3$) δ 166.6 (1C, CO_2CH_3), 165.3 (d, $^3J_{C6-F4}$ = 3.2 Hz, 1C, C6), 160.1, 131.8, 125.7, 116.9 (6C, Ar), 97.9 (dd, $^2J_{C1-F2}$ = 24.2 Hz, $^3J_{C1-F3}$ = 11.0 Hz, 1C, C1), 88.5 (ddd, $^1J_{C3-F3}$ = 196.4 Hz, $^2J_{C3-F2}$ = 19.6 Hz, $^2J_{C3-F4}$ = 18.1 Hz, 1C, C3), 87.8 (dd, $^1J_{C2-F2}$ = 189.2 Hz, $^2J_{C2-F3}$ = 20.1 Hz, 1C, C2), 87.1 (ddd, $^1J_{C4-F4}$ = 189.9 Hz, $^2J_{C4-F3}$ = 17.8 Hz, $^3J_{C4-F2}$ = 9.2 Hz, 1C, C4), 71.9 (dd, $^2J_{C5-F4}$ = 20.1 Hz, $^3J_{C5-F3}$ = 6.1 Hz, 1C, C5), 53.5, 52.3 (2C, 2 × CO_2CH_3) ppm; ^{19}F NMR (470 MHz, $CDCl_3$) δ –201.32 (dqd, $^2J_{F3-H3}$ = 48.0 Hz, $^3J_{F3-H2}$ = $^3J_{F3-F2}$ = $^3J_{F3-F4}$ = 14.2 Hz, $^3J_{F4-H3}$ = 6.1 Hz, 1F, F3), –207.57 (dtt, $^2J_{F2-H2}$ = 51.2 Hz, $^3J_{F2-F3}$ = $^3J_{F2-H3}$ = 14.1 Hz, $^3J_{F2-H1}$ = $^4J_{F2-F4}$ = 3.5 Hz, 1F, F2), –211.97 (dtd, $^2J_{F4-H4}$ = 49.1 Hz, $^3J_{F4-H3}$ = $^3J_{F4-H5}$ = 27.1 Hz, $^3J_{F4-F3}$ = 15.8 Hz, 1F, F4) ppm; HRMS calcd for $C_{15}H_{15}O_6F_3Na^+$ $[M + Na]^+$ 371.0713, found 371.0721.

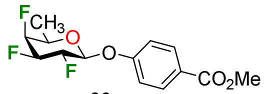

30

4-(Methoxycarbonyl)phenyl 2,3,4-trideoxy-2,3,4-trifluoro-β-D-fucopyranoside (30). To a stirred solution of compound **22** (6 mg, 0.018 mmol) in THF (0.2 mL) were added PPh_3 (6.9 mg, 0.0264 mmol, 1.5 equiv.) and Imidazole (2 mg, 0.035 mmol, 2 equiv.). The mixture was heated under reflux (~68 °C) for 30 min, and then I_2 (6.7 mg, 0.0264, 1.5 equiv.) was added. The mixture was heated under reflux (~68 °C) for another 2 h. After cooling to room temperature, a saturated aqueous $NaHCO_3$ solution (2 mL) was added. The mixture was extracted with CH_2Cl_2 (3 × 2 mL), and the combined organic phases were washed with brine (5 mL). The organic solution was dried over $MgSO_4$, filtered, and concentrated under reduced pressure. The crude iodo intermediate was used for the next step without further purification. To

a stirred solution of the crude iodo intermediate in toluene (0.5 mL) were added tris(trimethylsilyl)silane (11 µL, 0.035 mmol, 2 equiv.) and AIBN (1 mg, 1.7 µmol, 0.1 equiv.). The mixture was heated under reflux (~110 °C) for 18 h and then concentrated under reduced pressure. The resulting crude was purified by flash column chromatography (silica gel, EtOAc/ hexanes, 1:4 → 3:2) to give **30** as a white amorphous solid (3 mg, 9.86 µmol, 56% yield). $R_f = 0.32$ (silica, EtOAc/hexanes, 2:3); $[\alpha]_D^{25} = -46.5$ (*c* 0.1, $CHCl_3$); IR (ATR, ZnSe) ν 3009, 2920, 1720, 1236, 1167, 1057, 768 cm^{-1}; ^{1}H NMR (500 MHz, $CDCl_3$) δ 8.05 – 7.98 (m, 2H, Ar), 7.11 – 7.04 (m, 2H, Ar), 5.14 (dd, $^3J_{H1\text{-}H2}$ = 7.3 Hz, $^3J_{H1\text{-}F2}$ = 4.1 Hz, 1H, H1), 4.93 (ddddd, $^2J_{H2\text{-}F2}$ = 51.8 Hz, $^3J_{H2\text{-}F3}$ = 13.1 Hz, $^3J_{H2\text{-}H3}$ = 8.9 Hz, $^3J_{H2\text{-}H1}$ = 7.6 Hz, $^4J_{H2\text{-}F4}$ = 1.3 Hz, 1H, H2), 4.93 – 4.72 (m, 1H, H4), 4.74 (ddddd, $^2J_{H3\text{-}F3}$ = 47.3 Hz, $^3J_{H3\text{-}F4}$ = 26.3 Hz, $^3J_{H3\text{-}F2}$ = 14.2 Hz, $^3J_{H3\text{-}H2}$ = 9.1 Hz, $^3J_{H3\text{-}H4}$ = 3.1 Hz, 1H, H3), 3.90 (s, 3H, CO_2CH_3), 3.86 (dqd, $^3J_{H5\text{-}F4}$ = 25.1 Hz, $^3J_{H5\text{-}H6}$ = 6.6 Hz, $^3J_{H5\text{-}H4}$ = 1.9 Hz, 1H, H5), 1.47 (dt, $^3J_{H6\text{-}H5}$ = 6.6 Hz, $^4J_{H6\text{-}F4} = {}^4J_{H6\text{-}H4}$ = 0.7 Hz, 3H, 3 × H6) ppm; ^{13}C NMR (126 MHz, $CDCl_3$) δ 166.6 (1C, CO_2CH_3), 160.3, 131.7, 125.2, 116.5 (6C, Ar), 97.7 (dd, $^2J_{C1\text{-}F2}$ = 23.1 Hz, $^3J_{C1\text{-}F3}$ = 11.1 Hz, 1C, C1), 89.4 (dt, $^1J_{C3\text{-}F3}$ = 194.2 Hz, $^2J_{C3\text{-}F2} = {}^2J_{C3\text{-}F4}$ = 19.1 Hz, 1C, C3), 88.9 (ddd, $^1J_{C4\text{-}F4}$ = 188.0 Hz, $^2J_{C4\text{-}F3}$ = 15.8 Hz, $^3J_{C4\text{-}F2}$ = 9.0 Hz, 1C, C4), 88.2 (dd, $^1J_{C2\text{-}F2}$ = 188.4 Hz, $^2J_{C2\text{-}F3}$ = 19.6 Hz, 1C, C2), 69.0 (dd, $^2J_{C5\text{-}F4}$ = 19.2 Hz, $^3J_{C5\text{-}F3}$ = 5.7 Hz, 1C, C5), 52.2 (1C, CO_2CH_3), 15.8 (dd, $^3J_{C6\text{-}F4}$ = 5.2 Hz, $^4J_{C6\text{-}F3}$ = 1.9 Hz, 1C, C6) ppm; ^{19}F NMR (470 MHz, $CDCl_3$) δ –201.35 (dqd, $^2J_{F3\text{-}H3}$ = 48.2 Hz, $^3J_{F3\text{-}H2} = {}^3J_{F3\text{-}F2} = {}^3J_{F3\text{-}F4}$ = 14.1 Hz, $^3J_{F4\text{-}H3}$ = 6.6 Hz, 1F, F3), –208.13 (dtt, $^2J_{F2\text{-}H2}$ = 51.7 Hz, $^3J_{F2\text{-}F3} = {}^3J_{F2\text{-}H3}$ = 13.5 Hz, $^3J_{F2\text{-}H1} = {}^4J_{F2\text{-}F4}$ = 3.6 Hz, 1F, F2), –217.59 (dtd, $^2J_{F4\text{-}H4}$ = 50.8 Hz, $^3J_{F4\text{-}H3} = {}^3J_{F4\text{-}H5}$ = 25.4 Hz, $^3J_{F4\text{-}F3}$ = 15.8 Hz, 1F, F4) ppm; HRMS calcd for $C_{14}H_{16}O_4F_3^+$ $[M + H]^+$ 305.0995, found 305.1000.

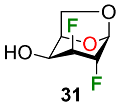

31

1,6-Anhydro-2,3-dideoxy-2,3-difluoro-β-D-galactopyranose (31). To a stirred solution of compound **16** (190 mg, 1.142 mmol) in CH_2Cl_2 (5 mL) at 0 °C were added pyridine (0.277 mL, 3.427 mmol, 3 equiv.) and 1M Tf_2O solution in CH_2Cl_2 (2.29 mL, 2.285 mmol, 2 equiv.). The mixture was stirred at room temperature for 25 min and then quenched with water (10 mL). The mixture was extracted with CH_2Cl_2 (3 × 5 mL), and the combined organic phases were successively washed with a saturated aqueous $NaHCO_3$ solution (15 mL), aqueous 1M HCl solution (15 mL) and brine (15 mL). The organic solution was dried over $MgSO_4$, filtered, and concentrated under reduced pressure. The crude triflate **17** was used

for the next step without further purification. To the crude triflate in CH_3CN (10 mL) was added $TBANO_2$ (989 mg, 3.427 mmol, 3.0 equiv.). The mixture was irradiated in a microwave reactor at 100 °C for 3 h. After cooling to room temperature, the reaction was quenched with water (60 mL) and brine (1 mL). The mixture was extracted with EtOAc (4 × 30 mL). The combined organic phases were dried over $MgSO_4$, filtered, and concentrated under a gentle stream of air (avoiding reduced pressure is important because of volatility issues). The resulting crude was purified through a short silica gel pad (Et_2O/CH_2Cl_2, 9:1) to give **31** as a white amorphous solid (180 mg, 1.08 mmol, 95%). R_f = 0.31 (silica, EtOAc/hexanes, 2:3); $[\alpha]_D^{25}$ = –30.6 (*c* 0.9, $CHCl_3$); IR (ATR, ZnSe) ν 3441, 2964, 2914, 1406, 1134, 1028, 932 cm^{-1}; ^1H NMR (500 MHz, $CDCl_3$) δ 5.51 (q, $^3J_{H1\text{-}H2} = {}^3J_{H1\text{-}F2} = {}^4J_{H1\text{-}H3}$ = 1.6 Hz, 1H, H1), 4.88 (dddq, $^2J_{H3\text{-}F3}$ = 47.2 Hz, $^3J_{H3\text{-}F2}$ = 9.4 Hz, $^3J_{H3\text{-}H2}$ = 4.7 Hz, $^4J_{H3\text{-}H1} = {}^3J_{H3\text{-}H4} = {}^3J_{H3\text{-}OH}$ = 1.6 Hz, 1H, H3), 4.62 (ddtt, $^2J_{H2\text{-}F2}$ = 44.1 Hz, $^3J_{H2\text{-}F3}$ = 11.2 Hz, $^3J_{H2\text{-}H1} = {}^3J_{H2\text{-}H3}$ = 1.8 Hz, $^4J_{H2\text{-}H4} = {}^5J_{H2\text{-}H6a}$ = 0.5 Hz, 1H, H2), 4.52 (dddd, $^3J_{H5\text{-}H6b}$ = 5.0 Hz, $^3J_{H5\text{-}H4}$ = 4.4 Hz, $^3J_{H5\text{-}H6a}$ = 1.2 Hz, $^4J_{H5\text{-}F3}$ = 0.8 Hz 1H, H5), 4.14 (ddq, $^2J_{H6a\text{-}H6b}$ = 7.9 Hz, $^3J_{H6a\text{-}H5}$ = 1.3 Hz, $^5J_{H6a\text{-}H2} = {}^4J_{H6a\text{-}H4} = {}^5J_{H6a\text{-}F3}$ = 0.6 Hz, 1H, H6a), 4.13 (br dt, $^3J_{H4\text{-}F3}$ = 26.4 Hz, $^3J_{H4\text{-}H5} = {}^3J_{H4\text{-}OH}$ = 4.6 Hz, 1H, H4), 3.73 (ddddt, $^2J_{H6b\text{-}H6a}$ = 7.9 Hz, $^3J_{H6b\text{-}H5}$ = 5.2 Hz, $^4J_{H6b\text{-}H4}$ = 1.7 Hz, $^5J_{H6b\text{-}F2}$ = 1.2 Hz, $^5J_{H6b\text{-}F3} = {}^4J_{H6b\text{-}H1}$ = 0.6 Hz, 1H, H6b), 2.33 (br s, 1H, OH) ppm; ^{13}C NMR (126 MHz, $CDCl_3$) δ 98.1 (d, $^2J_{C1\text{-}F2}$ = 25.8 Hz, 1C, C1), 87.4 (dd, $^1J_{C3\text{-}F3}$ = 177.8 Hz, $^2J_{C3\text{-}F2}$ = 33.0 Hz, 1C, C3), 86.1 (dd, $^1J_{C2\text{-}F2}$ = 181.9 Hz, $^2J_{C2\text{-}F3}$ = 27.9 Hz, 1C, C2), 74.0 (1C, C5), 64.8 (d, $^2J_{C4\text{-}F3}$ = 17.6 Hz, 1C, C4), 63.62 (d, $^3J_{C6\text{-}F3}$ = 3.3 Hz, 1C, C5) ppm; ^{19}F NMR (470 MHz, $CDCl_3$) δ –195.57 (ddd, $^2J_{F2\text{-}H2}$ = 44.2 Hz, $^3J_{F2\text{-}F3}$ = 13.9 Hz, $^3J_{F2\text{-}H3}$ = 9.5 Hz, 1F, F2), –208.96 (ddt, $^2J_{F3\text{-}H3}$ = 47.4 Hz, $^3J_{F3\text{-}H4}$ = 26.0 Hz, $^3J_{F3\text{-}H2} = {}^3J_{F3\text{-}F2}$ = 12.6 Hz, 1F, F3) ppm; HRMS calcd for $C_6H_{12}O_3F_2N^+$ $[M + NH_4]^+$ 184.0780, found 184.0783.

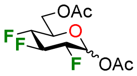

8

1,6-Di-*O*-acetyl-2,3,4-trideoxy-2,3,4-trifluoro-α/β-D-glucopyranose (8). To a stirred solution of compound **31** (94 mg, 0.563 mmol) in CH_2Cl_2 (4 mL) at room temperature, were added pyridine (0.137 mL, 1.690 mmol, 3 equiv.) and Tf_2O (0.190 mL, 1.127 mmol, 2 equiv.). The mixture was stirred at room temperature for 10 min and then quenched with water (10 mL). The mixture was extracted with CH_2Cl_2 (3 × 5 mL), and the combined organic phases were successively washed with a saturated aqueous $NaHCO_3$ solution (20 mL), aqueous 1M HCl solution (20 mL), and brine (20 mL). The organic solution was dried over $MgSO_4$, filtered, and concentrated under reduced pressure. The crude triflate was used for

the next step without further purification. To a solution of the crude triflate in CH_2Cl_2 (4 mL) was added TBAF·$3H_2O$ (267 mg, 0.845 mmol, 1.5 equiv.). The mixture was stirred at room temperature for 18 h and formation of the trifluoro intermediate **32** was monitored by TLC (R_f: 0.21, EtOAc/hexanes, 1:4). The mixture was cooled down to 0 °C and Ac_2O (1.60 mL, 16.90 mmol, 30 equiv.) and H_2SO_4 (0.30 mL, 5.634 mmol, 10 equiv.) were added. The mixture was stirred at room temperature for 18 h. After cooling to 0 °C, NaOAc (924 mg, 11.27 mmol, 20 equiv.) was added and the mixture was stirred for an additional 20 min. Water (20 mL) was added and the mixture was extracted with CH_2Cl_2 (3 × 15 mL). The combined organic phases were successively washed with a saturated aqueous $NaHCO_3$ solution (40 mL) and brine (40 mL). The organic solution was dried over $MgSO_4$, filtered, and concentrated under reduced pressure. The obtained crude was purified by flash column chromatography (silica gel, EtOAc/hexanes, 2:8 → 3:7) to give an anomeric mixture (α/β, 4.5:1) of **8** as a colorless thick oil (95.7 mg, 0.354 mmol, 63% yield, over 3 steps). A second purification using flash column chromatography (silica gel, acetone/toluene, 1:19 → 1:9) gave a pure fraction of the α anomer, that was used for characterization. R_f = 0.37 (silica, EtOAc/hexanes, 3:7); R_f = 0.52 (silica, acetone/toluene, 1:9); $[\alpha]_D^{25}$ = 87.1 (*c* 0.6, $CHCl_3$); IR (ATR, ZnSe) ν 2961, 1744, 1375, 1213, 1084, 1024, 933 cm^{-1}; ^1H NMR (500 MHz, $CDCl_3$) δ 6.4 (br q, $^3J_{H1-H2}$ = $^3J_{H1-F2}$ = $^4J_{H1-F3}$ = 3.3 Hz, 1H, H1), 5.03 (dddt, $^2J_{H3-F3}$ = 53.7 Hz, $^3J_{H3-F4}$ = 16.1 Hz, $^3J_{H3-F2}$ = 13.7 Hz, $^3J_{H3-H2}$ = $^3J_{H3-H4}$ = 8.6 Hz, 1H, H3), 4.68 (dddd, $^2J_{H2-F2}$ = 48.9 Hz, $^3J_{H2-F3}$ = 13.1 Hz, $^3J_{H2-H3}$ = 8.9 Hz, $^3J_{H2-H1}$ = 4.1 Hz, 1H, H2), 4.62 (dddd, $^2J_{H4-F4}$ = 50.4 Hz, $^3J_{H4-F3}$ = 14.8 Hz, $^3J_{H4-H5}$ = 10.1 Hz, $^3J_{H4-H3}$ = 8.4 Hz, 1H, H4), 4.39 (dq, $^2J_{H6a-H6b}$ = 12.5 Hz, $^3J_{H6a-H5}$ = $^4J_{H6a-H4}$ = $^4J_{H6a-F4}$ = 1.8 Hz, 1H, H6a), 4.27 (ddd, $^2J_{H6b-H6a}$ = 12.5 Hz, $^3J_{H6b-H5}$ = 4.4 Hz, $^4J_{H6b-F4}$ = 1.6 Hz, 1H, H6b), 4.07 (dtdt, $^3J_{H5-H4}$ = 10.1 Hz, $^3J_{H5-F4}$ = $^3J_{H5-H6b}$ = 4.3 Hz, $^3J_{H5-H6a}$ = 2.4 Hz, $^4J_{H5-H3}$ = $^4J_{H5-F3}$ = 0.6 Hz, 1H, H5), 2.19 (s, 3H, $COCH_3$), 2.10 (s, 3H, $COCH_3$) ppm; ^{13}C NMR (126 MHz, $CDCl_3$) δ 170.5, 168.4 (2C, 2 × $COCH_3$), 91.2 – 89.1 (m, 1C, C3), 88.4 (ddd, $^2J_{C1-F2}$ = 22.0 Hz, $^3J_{C1-F3}$ = 9.6 Hz, $^4J_{C1-F4}$ = 1.1 Hz, 1C, C1), 86.3 (ddd, $^1J_{C2-F2}$ = 195.4 Hz, $^2J_{C2-F3}$ = 18.4 Hz, $^3J_{C2-F4}$ = 8.4 Hz, 1C, C2), 86.3 (ddd, $^1J_{C4-F4}$ = 188.6 Hz, $^2J_{C4-F3}$ = 19.6 Hz, $^3J_{C4-F2}$ = 7.7 Hz, 1C, C4), 68.7 (dd, $^2J_{C5-F4}$ = 23.4 Hz, $^3J_{C5-F3}$ = 7.0 Hz, 1C, C5), 61.5 (1C, C6), 20.9, 20.8 (2C, 2 × $COCH_3$) ppm; ^{19}F NMR (470 MHz, $CDCl_3$) δ –200.09 (ddddp, $^2J_{F4-H4}$ = 52.1 Hz, $^3J_{F4-H3}$ = 16.1 Hz, $^3J_{F4-F3}$ = 12.8 Hz, $^3J_{F4-H5}$ = 4.2 Hz, $^4J_{F4-H2}$ = $^4J_{F4-F2}$ = $^4J_{F4-H6a}$ = $^4J_{F4-H6b}$ = 2.1 Hz, 1F, F4), –200.24 (br dp, $^3J_{F3-H3}$ = 53.9 Hz, $^3J_{F3-H2}$ = $^3J_{F3-F2}$ = $^3J_{F3-H4}$ = $^3J_{F3-F4}$ = 13.4 Hz, 1F, F3), –203.95 (dtd, $^2J_{F2-H2}$ = 48.9 Hz, $^3J_{F2-H3}$ = $^3J_{F2-F3}$ = 13.0 Hz, $^3J_{F2-H1}$ = 1.7 Hz, 1F, F2) ppm; HRMS calcd for $C_{10}H_{13}O_5F_3Na^+$ $[M + Na]^+$ 293.0607, found 293.0601.

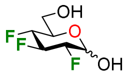

33

2,3,4-trideoxy-2,3,4-trifluoro-α/β-D-glucopyranose (33). To a stirred solution of compound **8** (50 mg, 0.186 mmol) in methanol (2 mL), was added dropwise a methanolic 1M NaOMe solution, until pH ≈ 9. The mixture was stirred at room temperature for 1 h and then neutralized to pH ≈ 7 with acidic resin. The mixture was filtered and concentrated under reduced pressure to afford **33** as a colorless oil (34 mg, 0.183 mmol, 98% yield). The spectroscopic data derived from compound **33** match those reported in the literature.

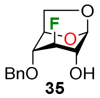

35

1,6-Anhydro-4-*O*-benzyl-3-deoxy-3-fluoro-β-D-glucopyranose (35). To a stirred solution of known compound **34** (3.9 g, 16.65 mmol) in ethylene glycol (120 mL) was added KHF_2 (7.93 g, 101.6 mmol, 6.1 equiv.). The mixture was heated under reflux (~200 °C) for 5 h. After cooling to room temperature, the mixture was quenched with an aqueous 5% K_2CO_3 solution (300 mL) and stirred for 5 min. The mixture was then extracted with CH_2Cl_2 (5 × 200 mL), and the combined organic phases were successively washed with water (2 × 200 mL), and brine (2 × 200 mL). The organic solution was dried over $MgSO_4$, filtered, and concentrated under reduced pressure. The crude oil was purified by flash column chromatography (silica gel, EtOAc/hexanes, 1:2 → 2:3) to give **35** as a white amorphous solid (2.75 g, 10.82 mmol, 65% yield). R_f = 0.38 (silica, EtOAc/hexanes, 2:3); $[\alpha]_D^{25}$ = –47.1 (*c* 0.5, MeOH); IR (ATR, ZnSe) ν 3434, 2962, 2870, 1415, 1321, 1078, 720 cm^{-1}; ^{1}H NMR (500 MHz, $CDCl_3$) δ 7.40 – 7.32 (m, 5H, Ar), 5.47 (t, $^2J_{H1-H2} = {}^3J_{H1-F3}$ = 2.0 Hz, 1H, H1), 4.73 – 4.61 (m, 4H, CH_2Ph, H3, H5), 4.01 (dt, $^2J_{H6a-H6b}$ = 7.7 Hz, $^3J_{H6a-H5} = {}^5J_{H6a-H2}$ = 1.2 Hz, 1H, H6a), 3.80 (ddd, $^2J_{H6b-H6a}$ = 7.8 Hz, $^3J_{H6b-H5}$ = 5.7 Hz, $^4J_{H6b-H4}$ = 2.1 Hz, 1H, H6b), 3.67 (tqd, $^3J_{H2-F3} = {}^3J_{H2-OH}$ = 12.2 Hz, $^3J_{H2-H1} = {}^3J_{H2-H3} = {}^5J_{H2-H6a}$ = 1.7 Hz, $^4J_{H2-H4}$ = 0.6 Hz, 1H, H2), 3.53 (dqd, $^3J_{H4-F3}$ = 12.9 Hz, $^3J_{H4-H3} = {}^3J_{H4-H5} = {}^4J_{H4-H6b}$ = 1.7 Hz, $^4J_{H4-H2}$ = 0.7 Hz, 1H), 2.50 (ddd, $^3J_{OH-H2}$ = 12.4 Hz, $^4J_{OH-F3}$ = 2.1 Hz, $^4J_{OH-H1}$ = 1.3 Hz, 1H, OH) ppm; ^{13}C NMR (126 MHz, $CDCl_3$) δ 136.9, 128.8, 128.5, 128.0 (6C, Ar), 101.4 (1C, C1), 88.2 (d, $^1J_{C3-F3}$ = 184.1 Hz, 1C, C3), 74.2 (d, $^2J_{C4-F3}$ = 26.5 Hz, 1C, C4), 73.7 (1C, C5), 71.8 (1C, *C*H_2Ph), 67.4 (d, $^2J_{C2-F3}$ = 23.7 Hz, 1C, C2), 65.1 (d, $^4J_{C6-F3}$ = 4.7 Hz, 1C, C6) ppm; ^{19}F NMR (470 MHz, $CDCl_3$) δ –184.73 (dt, $^2J_{F3-H3}$ = 44.2 Hz, $^3J_{F3-H2} = {}^3J_{F3-H4}$ = 12.5 Hz, 1F, F3) ppm; HRMS calcd for $C_{13}H_{19}O_4NF^+$ $[M + NH_4]^+$ 272.1293, found 272.1300.

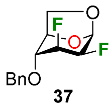

37

1,6-Anhydro-4-*O*-benzyl-2,3-dideoxy-2,3-difluoro-β-D-mannopyranose (37). To a stirred solution of compound **35** (1.90 g, 7.48 mmol) in CH_2Cl_2 (30 mL) at 0 °C, were added pyridine (1.21 mL, 10.26 mmol, 3 equiv.) and Tf_2O (1.39 mL, 8.233 mmol, 1.1 equiv.). The mixture was stirred at 0 °C for 30 min allowing formation of compound **36** and then a 1M TBAF solution in THF (75 mL, 75 mmol, 10 equiv.) was added. The mixture was stirred at room temperature for 22 h and then quenched with water (50 mL) and extracted with CH_2Cl_2 (3 × 100 mL). The combined organic phases were washed with a saturated aqueous $NaHCO_3$ solution (200 mL), dried over $MgSO_4$, filtered, and concentrated under reduced pressure. The obtained crude was purified by flash column chromatography (silica gel, EtOAc/hexanes, 3:7) to give **37** as a white amorphous solid (1.63 g, 6.361 mmol, 85% yield). R_f = 0.41 (silica, EtOAc/hexanes, 3:7); $[\alpha]_D^{25}$ = –85.3 (*c* 0.9, $CHCl_3$); IR (ATR, ZnSe) ν 3032, 2908, 1454, 1157, 1067, 897, 810 cm^{-1}; ^{1}H NMR (500 MHz, $CDCl_3$) δ 7.41 – 7.31 (m, 5H, Ar), 5.58 (br s, 1H, H1), 4.94 (dtt, $^2J_{H3\text{-}F3}$ = 49.1 Hz, $^3J_{H3\text{-}F2}$ = $^3J_{H3\text{-}H2}$ = 3.9 Hz, $^3J_{H3\text{-}H4}$ = $^4J_{H3\text{-}H5}$ = 1.9 Hz, 1H, H3), 4.69 (s, 2H, C*H*₂Ph), 4.63 – 4.57 (m, 1H, H5), 4.56 (dddd, $^2J_{H2\text{-}F2}$ = 44.6 Hz, $^3J_{H2\text{-}F3}$ = 23.6 Hz, $^3J_{H2\text{-}H3}$ = 4.4, $^3J_{H2\text{-}H1}$ = 1.9 Hz, 1H, H2), 4.07 (dt, $^2J_{H6a\text{-}H6b}$ = 7.8 Hz, $^3J_{H6a\text{-}H5}$ = $^4J_{H6a\text{-}H4}$ = 1.3 Hz, 1H, H6a), 3.84 (ddd, $^2J_{H6b\text{-}H6a}$ = 7.7 Hz, $^3J_{H6b\text{-}H5}$ = 5.9 Hz, $^4J_{H6b\text{-}H4}$ = 3.7 Hz, 1H, H6b), 3.78 (ddt, $^3J_{H4\text{-}F3}$ = 12.4 Hz, $^4J_{H4\text{-}H6b}$ = 4.2 Hz, $^3J_{H4\text{-}H3}$ = $^3J_{H4\text{-}H5}$ = 2.0 Hz, 1H, H4) ppm; ^{13}C NMR (126 MHz, $CDCl_3$) δ 136.9, 128.9, 128.6, 128.0 (6C, Ar), 99.0 (d, $^2J_{C1\text{-}F2}$ = 26.7 Hz, 1C, C1), 86.3 (dd, $^1J_{C3\text{-}F3}$ = 186.3 Hz, $^2J_{C3\text{-}F2}$ = 14.8 Hz, 1C, C3), 84.7 (dd, $^1J_{C2\text{-}F2}$ = 193.9 Hz, $^2J_{C2\text{-}F3}$ = 14.8 Hz, 1C, C2), 77.0 – 76.8 (m, 1C, C4), 73.6 (1C, C5), 72.2 (1C, *C*H₂Ph), 65.2 (d, $^4J_{C6\text{-}F3}$ = 5.8 Hz, 1C, C6) ppm; ^{19}F NMR (470 MHz, $CDCl_3$) δ –204.48 (dddt, $^2J_{F3\text{-}H3}$ = 49.1 Hz, $^3J_{F3\text{-}H2}$ = 22.8 Hz, $^3J_{F3\text{-}H4}$ = 12.7 Hz, $^3J_{F3\text{-}F2}$ = $^4J_{F3\text{-}H5}$ = 4.6 Hz, 1F, F3), –209.72 (br d, $^2J_{F2\text{-}H2}$ = 44.4 Hz, 1F, F2) ppm; HRMS calcd for $C_{13}H_{18}O_3F_2N^+$ $[M + NH_4]^+$ 274.1249, found 274.1245.

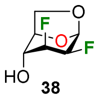

38

1,6-Anhydro-2,3-dideoxy-2,3-difluoro-β-D-mannopyranose (38). To a stirred solution of compound **37** (0.54 g, 2.107 mmol) in CH_2Cl_2 (20 mL) at 0 °C, was added a 1M $TiCl_4$ solution in CH_2Cl_2 (4.4 mL, 4.4 mmol, 2 equiv.). The mixture was stirred at 0 °C for 45 min and then quenched with water (20 mL).

The mixture was extracted with EtOAc (8 × 20 mL), and the combined organic phases were washed with brine (50 mL). The resulting aqueous phases were extracted again with EtOAc (3 × 20 mL). The combined organic solution was dried over $MgSO_4$, filtered, and concentrated under reduced pressure. The resulting crude was purified by flash column chromatography (silica gel, EtOAc/hexanes, 2:3 → 9:1) to give **38** as a white amorphous solid (308 mg, 1.854 mmol, 88% yield). R_f = 0.29 (silica, EtOAc/hexanes, 1:1); $[\alpha]_D^{25}$ = −132.2 (*c* 1.0, $CHCl_3$); IR (ATR, ZnSe) ν 3389, 2950, 1318, 1237, 1118, 1080, 773 cm^{-1}; ^{1}H NMR (500 MHz, $CDCl_3$) δ 5.59 (br s, 1H, H1), 4.91 (dtt, $^2J_{H3\text{-}F3}$ = 49.1 Hz, $^3J_{H3\text{-}F2}$ = $^3J_{H3\text{-}H2}$ = 4.0 Hz, $^3J_{H3\text{-}H4}$ = $^4J_{H3\text{-}H5}$ = 1.9 Hz, 1H, H3), 4.58 – 4.55 (m, 1H, H5), 4.54 (dddd, $^2J_{H2\text{-}F2}$ = 44.2 Hz, $^3J_{H2\text{-}F3}$ = 23.1 Hz, $^3J_{H2\text{-}H3}$ = 4.4, $^3J_{H2\text{-}H1}$ = 2.0 Hz, 1H, H2), 4.19 (ddd, $^2J_{H6a\text{-}H6b}$ = 7.8 Hz, $^3J_{H6a\text{-}H5}$ = 1.6 Hz, $^4J_{H6a\text{-}H4}$ = 1.2 Hz, 1H, H6a), 4.09 (ddt, $^3J_{H4\text{-}F3}$ = 10.0 Hz, $^4J_{H4\text{-}H6b}$ = 4.8 Hz, $^3J_{H4\text{-}H3}$ = $^3J_{H4\text{-}H5}$ = 2.3 Hz, 1H, H4), 3.90 (ddd, $^2J_{H6b\text{-}H6a}$ = 7.8 Hz, $^3J_{H6b\text{-}H5}$ = 5.9 Hz, $^4J_{H6b\text{-}H4}$ = 3.8 Hz, 1H, H6b), 2.24 (br s, 1H, OH) ppm; ^{13}C NMR (126 MHz, $CDCl_3$) δ 99.2 (d, $^2J_{C1\text{-}F2}$ = 26.6 Hz, 1C, C1), 87.9 (dd, $^1J_{C3\text{-}F3}$ = 187.2 Hz, $^2J_{C3\text{-}F2}$ = 14.4 Hz, 1C, C3), 84.1 (dd, $^1J_{C2\text{-}F2}$ = 195.1 Hz, $^2J_{C2\text{-}F3}$ = 15.2 Hz, 1C, C2), 75.7 (1C, C5), 70.8 (dd, $^2J_{C4\text{-}F3}$ = 25.3 Hz, $^3J_{C4\text{-}F2}$ = 3.9 Hz, 1C, C4), 65.2 (d, $^3J_{C6\text{-}F3}$ = 6.6 Hz, 1C, C6) ppm; ^{19}F NMR (470 MHz, $CDCl_3$) δ −204.00 (dddt, $^2J_{F3\text{-}H3}$ = 49.0 Hz, $^3J_{F3\text{-}H2}$ = 22.8 Hz, $^3J_{F3\text{-}H4}$ = 9.6 Hz, $^3J_{F3\text{-}F2}$ = $^4J_{F3\text{-}H5}$ = 4.1 Hz, 1F, F3), −210.39 (dq, $^2J_{F2\text{-}H2}$ = 44.1 Hz, $^3J_{F2\text{-}H3}$ = $^3J_{F2\text{-}H1}$ = $^3J_{F2\text{-}F3}$ = 5.1 Hz, 1F, F2) ppm; HRMS calcd for $C_6H_8O_3F_2Na^+$ [M + Na]$^+$ 189.0334, found 189.0334.

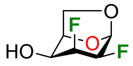

40

1,6-Anhydro-2,3-dideoxy-2,3-difluoro-β-D-talopyranose (40). To a stirred solution of compound **38** (174 mg, 1.047 mmol) in CH_2Cl_2 (5 mL) at 0 °C were added pyridine (0.81 mL, 10.01 mmol, 9.6 equiv.) and Tf_2O (0.41 mL, 2.499 mmol, 2.4 equiv.). The mixture was stirred at 0 °C for 30 min and then quenched with water (10 mL). The mixture was extracted with CH_2Cl_2 (3 × 10 mL), and the combined organic phases were successively washed with a saturated aqueous $NaHCO_3$ solution (20 mL), aqueous 1M HCl solution (20 mL) and brine (20 mL). The organic solution was dried over $MgSO_4$, filtered, and concentrated under reduced pressure. The crude triflate **39** was used for the next step without further purification. To the crude triflate in CH_3CN (9 mL) was added $TBANO_2$ (906 mg, 3.141 mmol, 3 equiv.). The mixture was irradiated in a microwave reactor at 100 °C for 3 h. After cooling to room temperature, the reaction was quenched with water (20 mL). The mixture was extracted with Et_2O (3 × 20 mL). The

combined organic phases were dried over $MgSO_4$, filtered, and concentrated under a gentle stream of air (avoiding reduced pressure is important because of volatility issues). The resulting crude was purified through a short silica gel pad (Et_2O/*n*-pentane, 4:1) to give **40** as a white amorphous solid (158.3 mg, 0.953 mmol, 91% yield). R_f = 0.25 (silica, EtOAc/hexanes, 1:1); $[\alpha]_D^{25}$ = –63.2 (*c* 0.5, $CHCl_3$); IR (ATR, ZnSe) ν 3431, 2986, 2920, 1128, 1022, 951, 790 cm^{-1}; ^1H NMR (500 MHz, $CDCl_3$) δ 5.54 (t, $^3J_{H1-H2}$ = $^3J_{H1-F2}$ = 1.8 Hz, 1H, H1), 5.10 (dtdt, $^2J_{H3-F3}$ = 54.1 Hz, $^3J_{H3-H2}$ = $^3J_{H3-H4}$ = 4.0 Hz, $^3J_{H3-F2}$ = 3.0 Hz, $^4J_{H3-H1}$ = $^3J_{H3-OH}$ = 1.4 Hz, 1H, H3), 4.50 – 4.47 (m, 1H, H5), 4.46 (ddddd, $^2J_{H2-F2}$ = 44.5 Hz, $^3J_{H2-F3}$ = 22.9 Hz, $^3J_{H2-H3}$ = 4.0, $^3J_{H2-H1}$ = 2.1 Hz, $^3J_{H3-OH}$ = 0.6 Hz, 1H, H2), 4.29 (dt, $^2J_{H6a-H6b}$ = 7.9 Hz, $^3J_{H6a-H5}$ = $^4J_{H6a-H4}$ = 1.1 Hz, 1H, H6a), 4.02 (dt, $^3J_{H4-F3}$ = 24.4 Hz, $^3J_{H4-H3}$ = $^3J_{H4-H5}$ = 4.1 Hz, 1H, H4), 3.82 (ddt, $^2J_{H6b-H6a}$ = 8.2 Hz, $^3J_{H6b-H5}$ = 5.2 Hz, $^4J_{H6b-H4}$ = $^5J_{H6b-F3}$ = 1.5 Hz, 1H, H6b), 2.56 (br s, 1H, OH) ppm; ^{13}C NMR (126 MHz, $CDCl_3$) δ 98.1 (d, $^2J_{C1-F2}$ = 25.9 Hz, 1C, C1), 88.1 (dd, $^1J_{C3-F3}$ = 183.6 Hz, $^2J_{C3-F2}$ = 14.8 Hz, 1C, C3), 85.8 (dd, $^1J_{C2-F2}$ = 197.7 Hz, $^2J_{C2-F3}$ = 14.8 Hz, 1C, C2), 74.1 (1C, C5), 67.1 (dd, $^2J_{C4-F3}$ = 17.2 Hz, $^3J_{C4-F2}$ = 1.7 Hz, 1C, C4), 65.1 (d, $^3J_{C6-F3}$ = 4.3 Hz, 1C, C6) ppm; ^{19}F NMR (470 MHz, $CDCl_3$) δ –205.61 (ddt, $^2J_{F2-H2}$ = 45.1 Hz, $^3J_{F2-F3}$ = 7.8 Hz, $^3J_{F2-H1}$ = $^3J_{F2-H3}$ = 3.4 Hz, 1F, F2), –220.98 (dtd, $^2J_{F3-H3}$ = 55.0 Hz, $^3J_{F3-H2}$ = $^3J_{F3-H4}$ = 23.6 Hz, $^3J_{F3-F2}$ = 8.2 Hz, 1F, F3) ppm; HRMS calcd for $C_6H_8O_3F_2Na^+$ $[M + Na]^+$ 189.0334, found 189.0338.

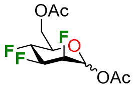

9

1,6-Di-*O*-acetyl-2,3,4-trideoxy-2,3,4-trifluoro-α/β-D-mannopyranose (9). To a stirred solution of compound **40** (110 mg, 0.662 mmol) in CH_2Cl_2 (3.5 mL) at room temperature, were added pyridine (0.52 mL, 6.43 mmol, 9.7 equiv.) and Tf_2O (0.26 mL, 1.523 mmol, 2.3 equiv.). The mixture was stirred at 0 °C for 30 min and then quenched with water (10 mL). The mixture was extracted with CH_2Cl_2 (4 × 10 mL), and the combined organic phases were successively washed with an aqueous 1M HCl solution (2 × 20 mL), and brine (20 mL). The organic solution was dried over $MgSO_4$, filtered, and concentrated under reduced pressure. The crude triflate **41** was used for the next step without further purification. To a solution of the crude triflate **41** in Et_3N (5 mL) was added $Et_3N\cdot 3HF$ (1.6 mL, 9.93 mmol, 15 equiv.). The mixture was stirred at 80 °C for 48 h allowing formation of intermediate **42**. After this time, the mixture was cooled down to 0 °C and Ac_2O (12.5 mL, 132.4 mmol, 200 equiv.) and H_2SO_4 (2.8 mL, 52.96 mmol, 80 equiv.) were added. The mixture was stirred at room temperature for 16 h. After cooling to 0 °C,

NaOAc (5.43 g, 66.2 mmol, 100 equiv.) was added. The mixture was stirred for an additional 20 min and then quenched with water (50 mL). The mixture was extracted with CH_2Cl_2 (4 × 30 mL). The combined organic phases were successively washed with a saturated aqueous $NaHCO_3$ solution (100 mL), aqueous 1M HCl solution (100 mL) and brine (100 mL). The organic solution was dried over $MgSO_4$, filtered, and concentrated under reduced pressure. The obtained crude was purified by flash column chromatography (silica gel, EtOAc/hexanes, 3:7) to give the desired product **9** (contaminated with an unidentified elimination product) and compound **10** as a white amorphous solid (52 mg, 0.1925 mmol, 29% yield). The mixture containing **9** and the elimination product were dissolved in EtOH/H_2O (2:1) (5 mL) at 0 °C and $KMnO_4$ (76 mg) and K_2CO_3 (54 mg) were added. The mixture was stirred at 0 °C for 2 h and then quenched with water (5 mL). The mixture was extracted with CH_2Cl_2 (3 × 5 mL). The combined organic phases were successively washed with aqueous 1M HCl solution (10 mL) and brine (10 mL). The organic solution was dried over $MgSO_4$, filtered, and concentrated under reduced pressure. The obtained crude was purified by flash column chromatography (silica gel, EtOAc/hexanes, 3:7) to give pure product **9** as an anomeric mixture (α/β, 10.2:1) as a colorless thick oil (75 mg, 0.278 mmol, 42% yield). R_f = 0.30 (silica, EtOAc/hexanes, 3:7); $[\alpha]_D^{25}$ = 44.9 (*c* 0.2, $CHCl_3$); IR (ATR, ZnSe) ν 2924, 2853, 1744, 1375, 1217, 1016, 976 cm^{-1}; ^{1}H NMR (500 MHz, $CDCl_3$) δ 6.33 (ddd, $^3J_{H1\text{-}F2}$ = 6.3 Hz, $^3J_{H1\text{-}H2}$ = 5.6 Hz, $^4J_{H1\text{-}F3}$ = 2.2 Hz, 1H, H1β), 6.29 (ddt, $^3J_{H1\text{-}F2}$ = 6.6 Hz, $^3J_{H1\text{-}F2}$ = 4.5 Hz, $^4J_{H1\text{-}H3}$ = $^4J_{H1\text{-}F3}$ = 2.4 Hz, 1H, H1α), 5.03 – 4.69 (m, 6H, H2α, H2β, H3α, H3β, H4α, H4β), 4.46 (dt, $^2J_{H6a\text{-}H6b}$ = 12.3 Hz, $^3J_{H6a\text{-}H5}$ = $^4J_{H6a\text{-}H4}$ = 2.1 Hz, 1H, H6aβ), 4.45 (dq, $^2J_{H6a\text{-}H6b}$ = 12.3 Hz, $^3J_{H6a\text{-}H5}$ = $^4J_{H6a\text{-}H4}$ = $^4J_{H6a\text{-}F4}$ = 2.0 Hz, 1H, H6aα), 4.38 (dd, $^2J_{H6b\text{-}H6a}$ = 12.4 Hz, $^3J_{H6b\text{-}H5}$ = 4.1 Hz, 1H, H6bβ), 4.28 (ddd, $^2J_{H6b\text{-}H6a}$ = 12.4 Hz, $^3J_{H6b\text{-}H5}$ = 4.6, $^4J_{H6b\text{-}F4}$ = 1.6 Hz, 1H, H6bα), 4.06 – 4.00 (m, 2H, H5α, H5β), 2.16 (s, 3H, COC*H*$_3$β), 2.16 (s, 3H, COC*H*$_3$α), 2.12 (s, 3H, COC*H*$_3$β), 2.12 (s, 3H, COC*H*$_3$α) ppm; ^{13}C NMR (126 MHz, $CDCl_3$) δ 170.70, 170.66, 167.90, 167.89 (4C, 2 × *C*OCH$_3$α, 2 × *C*OCH$_3$β), 90.5 (dd, $^2J_{C1\text{-}F2}$ = 30.2 Hz, $^3J_{C1\text{-}F3}$ = 6.7 Hz, 1C, C1β), 90.3 (ddd, $^2J_{C1\text{-}F2}$ = 30.4 Hz, $^3J_{C1\text{-}F3}$ = 6.7 Hz, $^4J_{C1\text{-}F4}$ = 0.8 Hz, 1C, C1α), 88.5 – 86.5 (m, 1C, C3α), 89.1 – 84.7 (m, 3C, C2β, C3β, C4β), 86.4 (ddd, $^1J_{C2\text{-}F2}$ = 183.0 Hz, $^2J_{C2\text{-}F3}$ = 16.5 Hz, $^3J_{C2\text{-}F4}$ = 9.3 Hz, 1C, C2α), 85.2 (ddd, $^2J_{C4\text{-}F4}$ = 184.5 Hz, $^2J_{C4\text{-}F3}$ = 20.6 Hz, $^3J_{C4\text{-}F2}$ = 1.5 Hz, 1C, C4α), 72.8 (d, $^3J_{C5\text{-}F3}$ = 5.2 Hz, 1C, C5β), 69.92 (dd, $^2J_{C5\text{-}F4}$ = 23.8 Hz, $^3J_{C5\text{-}F3}$ = 6.7 Hz, 1C, C5α), 62.25 (d, $^3J_{C6\text{-}F4}$ = 1.8 Hz, 1C, C6β), 61.70 (d, $^3J_{C6\text{-}F4}$ = 2.0 Hz, 1C, C6α), 20.92, 20.90, 20.88, 20.86 (4C, 2 × CO*C*H$_3$α, 2 × CO*C*H$_3$β) ppm; ^{19}F NMR (470 MHz, $CDCl_3$) δ –198.77 (ddq, $^2J_{F3\text{-}H3}$ = 46.0 Hz, $^3J_{F3\text{-}F2}$ = 15.7 Hz, $^3J_{F3\text{-}H2}$ = $^3J_{F3\text{-}H4}$ = $^3J_{F3\text{-}F4}$ = 7.3 Hz, 1F, F3β), –205.63 (dddd, $^2J_{F2\text{-}H2}$ = 49.0 Hz, $^3J_{F2\text{-}H3}$ = 27.4 Hz, $^3J_{F2\text{-}F3}$ = 16.4 Hz, $^3J_{F2\text{-}H1}$ = 6.4 Hz, 1F, F2α), –206.34

– –206.71 (m, 4F, F2β, F3α, F4α, F4β) ppm; HRMS calcd for $C_{10}H_{14}O_5F_3^+$ $[M + H]^+$ 271.0788, found 271.0788.

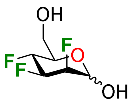

43

2,3,4-trideoxy-2,3,4-trifluoro-α/β-D-mannopyranose (43). To a stirred solution of compound **9** (39.5 mg, 0.146 mmol) in methanol (1 mL) was added dropwise a methanolic 1M NaOMe solution, until pH ≈ 9. The mixture was stirred at room temperature for 1 h and then neutralized to pH ≈ 7 with acidic resin. The mixture was filtered and concentrated under reduced pressure. The obtained crude was purified by flash column chromatography (silica gel, methanol/CH_2Cl_2, 1:19) to give pure product **33** as a colorless thick oil (25.5 mg, 0.137 mmol, 94% yield). R_f = 0.38 (silica, EtOAc/hexanes, 4:1); $[\alpha]_D^{25}$ = 22.2 (*c* 0.9, MeOH); IR (ATR, diamond crystal) ν 3348, 2947, 1396, 1119, 1057, 802, 671 cm^{-1}; ^1H NMR (500 MHz, Acetone-d_6) δ 6.39 (br s, 1H, OH), 5.36 (ddt, $^3J_{H1-F2}$ = 6.7 Hz, $^3J_{H1-H2}$ = 4.4 Hz, $^4J_{H1-F3}$ = 2.4 Hz, 1H, H1), 4.99 (dddddd, $^2J_{H3-F3}$ = 49.0 Hz, $^3J_{H3-F2}$ = 27.7 Hz, $^3J_{H3-F4}$ = 14.8 Hz, $^3J_{H3-H4}$ = 9.1 Hz, $^3J_{H3-H2}$ = 2.9 Hz, $^4J_{H3-H5}$ = 0.4 Hz, 1H, H3), 5.02 – 4.77 (m, 2H, H2, H4), 4.00 – 3.91 (m, 2H, H5, OH), 3.85 – 3.70 (m, 2H, H6a, H6b) ppm; ^{13}C NMR (126 MHz, Acetone-d_6) δ 92.6 (ddd, $^2J_{C1-F2}$ = 27.7 Hz, $^3J_{C1-F3}$ = 7.3 Hz, $^4J_{C1-F4}$ = 1.4 Hz, 1C, C1), 89.6 (ddd, $^1J_{C3-F3}$ = 188.0 Hz, $^2J_{C3-F4}$ = 19.1 Hz, $^2J_{C3-F2}$ = 16.4 Hz, 1C, C3), 89.4 (ddd, $^1J_{C2-F2}$ = 176.9 Hz, $^2J_{C2-F3}$ = 15.1 Hz, $^3J_{C1-F4}$ = 9.0 Hz, 1C, C2), 86.9 (ddd, $^1J_{C4-F4}$ = 178.8 Hz, $^2J_{C4-F3}$ = 18.6 Hz, $^3J_{C4-F2}$ = 1.0 Hz, 1C, C4), 70.7 (dd, $^2J_{C5-F4}$ = 23.5 Hz, $^3J_{C5-F3}$ = 6.0 Hz, 1C, C5), 60.9 (d, $^3J_{C6-F4}$ = 1.8 Hz, 1C, C6) ppm; ^{19}F NMR (470 MHz, Acetone-d_6) δ –205.60 (dddd, $^2J_{F2-H2}$ = 50.6 Hz, $^3J_{F2-H3}$ = 27.7 Hz, $^3J_{F2-F3}$ = 16.5 Hz, $^3J_{F2-H1}$ = 6.9 Hz, 1F, F2), –206.30 (dtp, $^2J_{F4-H4}$ = 52.2 Hz, $^3J_{F4-F3}$ = 14.9 Hz, $^3J_{F4-H3}$ = 13.0 Hz, $^2J_{F4-H5}$ = 4.4 Hz, $^4J_{F4-F2}$ = 2.2 Hz, 1F, F4), –206.65 – –206.91 (m, 1F, F3) ppm; HRMS calcd for $C_6H_{13}O_3NF_3^+$ $[M + NH_4]^+$ 204.0842, found 204.0850.

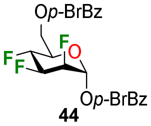

44

1,6-Di-*O*-(4-bromobenzoyl)-2,3,4-trideoxy-2,3,4-trifluoro-α-D-mannopyranose (44). To a stirred solution of compound **43** (6.5 mg, 0.035 mmol) in CH_2Cl_2 (1 mL) were added Et_3N (40 μL, 0.287 mmol,

8 equiv.), *p*-bromobenzoylchloride (30.7 mg, 0.140 mmol, 4 equiv.) and DMAP (4.3 mg, 0.035 mmol, 1 equiv.). The mixture was stirred at room temperature for 18 h and then quenched with water (5 mL). The mixture was extracted with CH_2Cl_2 (4 × 5 mL). The combined organic phases were successively washed with aqueous 1M HCl solution (2 × 10 mL) and brine (10 mL). The organic solution was dried over $MgSO_4$, filtered, and concentrated under reduced pressure. The obtained crude was purified by flash column chromatography (silica gel, EtOAc/hexanes, 1:9 → 1:4). The resulting product was recrystallized from acetone/heptane to give **44** as colorless crystals (15.9 mg, 0.029 mmol, 83% yield). R_f = 0.23 (silica, EtOAc/hexanes, 1:9); m.p. = 147 – 148 °C; $[\alpha]_D^{25}$ = –38.3 (*c* 0.4, $CHCl_3$); IR (ATR, ZnSe) ν 2918, 2851, 1724, 1589, 1259, 1055, 976 cm^{-1}; ^{1}H NMR (500 MHz, $CDCl_3$) δ 7.92 – 7.82 (m, 4H, Ar), 7.69 – 7.62 (m, 2H, Ar), 7.63 – 7.56 (m, 2H, Ar), 6.55 (ddt, $^3J_{H1-F2}$ = 6.6 Hz, $^3J_{H1-F2}$ = 4.3 Hz, $^4J_{H1-H3}$ = $^4J_{H1-F3}$ = 2.4 Hz, 1H, H1), 5.20 – 4.96 (m, 3H, H2, H3, H4), 4.70 (dq, $^2J_{H6a-H6b}$ = 12.5 Hz, $^3J_{H6a-H5}$ = $^4J_{H6a-H4}$ = $^4J_{H6a-F4}$ = 2.2 Hz, 1H, H6a), 4.55 (ddt, $^2J_{H6b-H6a}$ = 12.3 Hz, $^3J_{H6b-H5}$ = 4.2, $^4J_{H6b-H4}$ = $^4J_{H6b-F4}$ = 1.1 Hz, 1H, H6b), 4.26 – 4.18 (m, 1H, H5) ppm; ^{13}C NMR (126 MHz, $CDCl_3$) δ 165.4, 163.0 (2C, 2 × *C*OCH_3), 132.5, 132.0, 131.5, 131.4, 130.0, 128.7, 128.4, 127.1 (12C, Ar), 91.1 (dd, $^2J_{C1-F2}$ = 30.5 Hz, $^3J_{C1-F3}$ = 6.7 Hz, 1C, C1), 88.8 – 86.8 (m, 1C, C3), 86.4 (ddd, $^1J_{C2-F2}$ = 182.7 Hz, $^2J_{C2-F3}$ = 16.3 Hz, $^3J_{C2-F4}$ = 9.3 Hz, 1C, C2), 85.3 (dd, $^1J_{C4-F4}$ = 185.2 Hz, $^2J_{C4-F3}$ = 21.7 Hz, 1C, C4), 70.3 (dd, $^2J_{C5-F4}$ = 23.8 Hz, $^3J_{C5-F3}$ = 6.8 Hz, 1C, C5), 62.2 (d, $^3J_{C6-F4}$ = 1.4 Hz, 1C, C6) ppm; ^{19}F NMR (470 MHz, $CDCl_3$) δ –205.54 (dddd, $^2J_{F2-H2}$ = 48.3 Hz, $^3J_{F2-H3}$ = 28.5 Hz, $^3J_{F2-F3}$ = 16.6 Hz, $^3J_{F2-H1}$ = 6.0 Hz, 1F, F2), –205.98 (br dt, $^2J_{F3-H3}$ = 50.4 Hz, $^3J_{F3-F2}$ = $^3J_{F3-F4}$ = 16.2 Hz, 1F, F3), –206.00 – –206.23 (m, 1F, F4) ppm; HRMS calcd for $C_{20}H_{15}O_5F_3Br_2Na^+$ $[M + Na]^+$ 574.9111, found 574.9115.

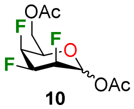

10

1,6-Di-*O*-acetyl-2,3,4-trideoxy-2,3,4-trifluoro-α/β-D-talopyranose (10). To a stirred solution of compound **40** (66 mg, 0.396 mmol) in CH_2Cl_2 (5 mL) was added DAST (105 µL, 0.791 mmol, 2 equiv.). The mixture was irradiated in a microwave reactor at 100 °C for 1 h. After this time, the mixture was cooled to 0 °C and Ac_2O (1.12 mL, 11.85 mmol, 30 equiv.) and H_2SO_4 (211 µL, 3.955 mmol, 10 equiv.) were added. The mixture was stirred at room temperature for 16 h. After cooling to 0 °C, NaOAc (649 mg, 7.91 mmol, 20 equiv.) was added and the mixture was stirred for an additional 20 min. Water (10 mL) was added and the mixture was extracted with CH_2Cl_2 (3 × 10 mL). The combined organic phases were

successively washed with water (20 mL) and brine (20 mL). The organic solution was dried over $MgSO_4$, filtered, and concentrated under reduced pressure. The obtained crude was purified by flash column chromatography (silica gel, EtOAc/hexanes, 2:3) to give an anomeric mixture (α/β, 23:1) of **10** as a colorless thick oil (83 mg, 0.303 mmol, 77% yield). R_f = 0.22 (silica, EtOAc/hexanes, 2:3); $[\alpha]_D^{25}$ = 79.3 (*c* 0.6, $CHCl_3$); IR (ATR, ZnSe) ν 2924, 1744, 1371, 1221, 1140, 1022, 795 cm^{-1}; ^1H NMR (500 MHz, $CDCl_3$) δ 6.39 (ddd, $^3J_{H1-F2}$ = 7.5 Hz, $^4J_{H1-F3}$ = 5.9 Hz, $^3J_{H1-H2}$ = 1.9 Hz, 1H, H1), 4.99 (dddt, $^2J_{H4-F4}$ = 50.6 Hz, $^3J_{H4-F3}$ = 6.7 Hz, $^3J_{H4-H3}$ = 2.8 Hz, $^3J_{H4-H5}$ = $^4J_{H4-H6a}$ = 1.0 Hz, 1H, H4), 4.79 (dddt, $^2J_{H2-F2}$ = 49.6 Hz, $^3J_{H2-F3}$ = 5.8 Hz, $^3J_{H2-H3}$ = 3.0 Hz, $^3J_{H2-H1}$ = $^4J_{H2-F4}$ = 1.5 Hz, 1H, H2), 4.77 (dtt, $^2J_{H3-F3}$ = 42.7 Hz, $^3J_{H3-F2}$ = $^3J_{H2-F4}$ = 29.0 Hz, $^3J_{H3-H2}$ = $^3J_{H3-H4}$ = 3.0 Hz, 1H, H3), 4.37 (ddt, $^2J_{H6a-H6b}$ = 11.4 Hz, $^3J_{H6a-H5}$ = 6.7 Hz, $^4J_{H6a-H4}$ = $^4J_{H6a-F4}$ = 1.3 Hz, 1H, H6a), 4.31 (dd, $^2J_{H6b-H6a}$ = 11.5 Hz, $^3J_{H6b-H5}$ = 6.4 Hz, 1H, H6b), 4.13 (dtdd, $^3J_{H5-F4}$ = 27.6 Hz, $^3J_{H5-H6a}$ = $^3J_{H5-H6b}$ = 6.5 Hz, $^4J_{H5-F3}$ = 1.9 Hz, $^3J_{H5-H4}$ = 1.0 Hz, 1H, H5), 2.13 (s, 3H, $COCH_3$), 2.09 (s, 3H, $COCH_3$) ppm; ^{13}C NMR (126 MHz, $CDCl_3$) δ 170.5, 167.8 (2C, 2 × $COCH_3$), 91.0 (dd, $^2J_{C1-F2}$ = 31.4 Hz, $^3J_{C1-F3}$ = 7.1 Hz, 1C, C1), 84.6 (dd, $^1J_{C4-F4}$ = 193.3 Hz, $^2J_{C4-F3}$ = 17.9 Hz, 1C, C4), 83.9 (dd, $^1J_{C2-F2}$ = 188.9 Hz, $^2J_{C2-F3}$ = 17.5 Hz, 1C, C2), 83.5 (dt, $^1J_{C3-F3}$ = 196.1 Hz, $^2J_{C3-F2}$ = $^2J_{C3-F4}$ = 16.5 Hz, 1C, C3), 69.2 (dd, $^2J_{C5-F4}$ = 18.6 Hz, $^3J_{C5-F3}$ = 5.5 Hz, 1C, C5), 61.4 (dd, $^3J_{C6-F4}$ = 6.7 Hz, $^4J_{C6-F3}$ = 2.5 Hz, 1C, C6), 20.8, 20.7 (2C, 2 × $COCH_3$) ppm; ^{19}F NMR (470 MHz, $CDCl_3$) δ –205.46 (dtdd, $^3J_{F2-H2}$ = 49.0 Hz, $^3J_{F2-H3}$ = $^4J_{F2-F4}$ = 29.2 Hz, $^3J_{F2-F3}$ = 13.3 Hz, $^3J_{F2-H1}$ = 7.8 Hz, 1F, F2), –208.33 (dtq, $^3J_{F3-H3}$ = 42.7 Hz, $^3J_{F3-F2}$ = $^3J_{F3-F4}$ = 12.2 Hz, $^4J_{F3-H1}$ = $^3J_{F3-H2}$ = $^3J_{F3-H4}$ = 6.1 Hz, 1F, F3), –219.90 (dqd, $^2J_{F4-H4}$ = 50.4 Hz, $^3J_{F4-H3}$ = $^3J_{F4-H5}$ = $^4J_{F4-F2}$ = 28.1 Hz, $^3J_{F4-F3}$ = 12.2 Hz, 1F, F4) ppm; HRMS calcd for $C_{10}H_{13}O_5F_3Na^+$ $[M + Na]^+$ 293.0607, found 293.0608.

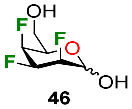

46

2,3,4-trideoxy-2,3,4-trifluoro-α/β-D-talopyranose (46). To a stirred solution of compound **10** (34.9 mg, 0.129 mmol) in water (1.3 mL) at room temperature, was added an aqueous hydrochloric acid solution (37%) (2.9 mL). The mixture was stirred room temperature for 1 h and then evaporated with a gentle air flow. The obtained yellow crude was purified by flash column chromatography (silica gel, EtOAc/hexanes, 4:1) to give pure product **46** (α/β, 11:1) as a colorless thick oil (23.8 mg, 0.128 mmol, 99% yield). R_f = 0.31 (silica, EtOAc/hexanes, 4:1); $[\alpha]_D^{25}$ = 38.7 (c 0.8, MeOH); IR (ATR, diamond crystal) ν 3337, 2959, 1396, 1117, 1051, 793, 677 cm^{-1}; ^1H NMR (500 MHz, Acetone-d_6) δ 6.21 (dd, $^3J_{OH-}$

$_{H1}$ = 4.5 Hz, $^{4}J_{OH-F2}$ = 2.3 Hz, 1H, OH), 5.41 (dtd, $^{3}J_{H1-F2}$ = 8.0 Hz, $^{3}J_{H1-OH}$ = $^{4}J_{H1-F3}$ = 5.5 Hz, $^{3}J_{H1-H2}$ = 1.2 Hz, 1H, H1), 5.04 (dddt, $^{2}J_{H4-F4}$ = 51.7 Hz, $^{3}J_{H4-F3}$ = 7.1 Hz, $^{3}J_{H4-H3}$ = 2.7 Hz, $^{3}J_{H4-H5}$ = $^{4}J_{H4-H6b}$ = 1.2 Hz, 1H, H4), 4.94 (dtt, $^{2}J_{H3-F3}$ = 42.0 Hz, $^{3}J_{H3-F2}$ = $^{3}J_{H3-F4}$ = 30.4 Hz, $^{3}J_{H3-H2}$ = $^{3}J_{H3-H4}$ = 2.9 Hz, 1H, H3), 4.79 (dddtd, $^{2}J_{H2-F2}$ = 50.6 Hz, $^{3}J_{H2-F3}$ = 6.4 Hz, $^{3}J_{H2-H3}$ = 3.0 Hz, $^{3}J_{H2-H1}$ = $^{4}J_{H2-F4}$ = 1.9 Hz, $^{4}J_{H2-H4}$ = 0.3 Hz, 1H, H2), 4.13 (dtddd, $^{3}J_{H5-F4}$ = 29.2 Hz, $^{3}J_{H5-H6a}$ = $^{4}J_{H5-H4}$ = 7.4 Hz, $^{3}J_{H5-H6b}$ = 6.4 Hz, $^{3}J_{H5-H4}$ = 2.0 Hz, $^{4}J_{H5-F3}$ = 0.9 Hz, 1H, H5), 4.02 (dd, $^{3}J_{OH-H6a}$ = 6.6 Hz, $^{3}J_{OH-H6b}$ = 5.0 Hz, 1H, OH), 3.76 (dt, $^{2}J_{H6a-H6b}$ = 10.7 Hz, $^{3}J_{H6a-H5}$ = $^{3}J_{H6a-OH}$ = 6.9 Hz, 1H, H6a), 3.68 (dddt, $^{2}J_{H6b-H6a}$ = 10.7 Hz, $^{3}J_{H6b-H5}$ = 6.5 Hz, $^{3}J_{H6b-OH}$ = 4.8 Hz, $^{4}J_{H6b-H4}$ = $^{4}J_{H6b-F4}$ = 1.8 Hz, 1H, H6b) ppm; ^{13}C NMR (126 MHz, Acetone-d_6) δ 93.4 (dd, $^{2}J_{C1-F2}$ = 29.4 Hz, $^{3}J_{C1-F3}$ = 7.5 Hz, 1C, C1), 87.2 (dd, $^{1}J_{C2-F2}$ = 183.2 Hz, $^{2}J_{C2-F3}$ = 16.1 Hz, 1C, C2), 86.4 (dd, $^{1}J_{C4-F4}$ = 188.5 Hz, $^{2}J_{C4-F3}$ = 16.9 Hz, 1C, C4), 85.7 (dt, $^{1}J_{C3-F3}$ = 190.3 Hz, $^{2}J_{C3-F2}$ = $^{2}J_{C3-F4}$ = 16.0 Hz, 1C, C3), 70.2 (dd, $^{2}J_{C5-F4}$ = 18.2 Hz, $^{3}J_{C5-F3}$ = 5.1 Hz, 1C, C5), 60.4 (dd, $^{3}J_{C6-F4}$ = 6.5 Hz, $^{4}J_{C6-F3}$ = 2.7 Hz, 1C, C6) ppm ; ^{19}F NMR (470 MHz, Acetone-d_6) δ –204.50 – –204.67 (m, 1F, F3β), –204.83 (dddddd, $^{2}J_{F2-H2}$ = 50.5 Hz, $^{3}J_{F2-H3}$ = 29.9 Hz, $^{4}J_{F2-F4}$ = 27.8 Hz, $^{3}J_{F2-F3}$ = 13.9 Hz, $^{3}J_{F2-H1}$ = 8.5 Hz, $^{3}J_{F2-OH}$ = 2.1 Hz, 1F, F2α), –208.64 (dtq, $^{2}J_{F3-H3}$ = 40.5 Hz, $^{3}J_{F3-F2}$ = $^{3}J_{F3-F4}$ = 13.4 Hz, $^{4}J_{F3-H1}$ = $^{3}J_{F3-H2}$ = $^{3}J_{F3-H4}$ = 6.7 Hz, 1F, F3α), –219.98 (dtdd, $^{2}J_{F4-H4}$ = 51.5 Hz, $^{3}J_{F4-H3}$ = $^{3}J_{F4-H5}$ = 28.5 Hz, $^{4}J_{F4-F2}$ = 24.7 Hz, $^{3}J_{F4-F3}$ = 12.5 Hz, 1F, F4β), –220.84 (dqd, $^{2}J_{F4-H4}$ = 51.3 Hz, $^{3}J_{F4-H3}$ = $^{3}J_{F4-H5}$ = $^{4}J_{F4-F2}$ = 28.8 Hz, $^{3}J_{F4-H3}$ = 12.5 Hz, 1F, F4α), –224.04 (ddddd, $^{2}J_{F2-H2}$ = 52.0 Hz, $^{3}J_{F2-H3}$ = 30.8 Hz, $^{4}J_{F2-F4}$ = 24.4 Hz, $^{3}J_{F2-H1}$ = 19.5 Hz, $^{3}J_{F2-F3}$ = 12.9 Hz, 1F, F2β) ppm; HRMS calcd for $C_6H_{13}O_3NF_3^+$ [M + NH_4]$^+$ 204.0842, found 204.0847.

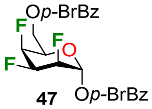

47

1,6-Di-*O*-(4-bromobenzoyl)-2,3,4-trideoxy-2,3,4-trifluoro-α-D-talopyranose (47). To a stirred solution of compound **46** (8.3 mg, 0.045 mmol) in CH_2Cl_2 (1 mL) were added Et_3N (50 µL, 0.357 mmol, 8 equiv.), *p*-bromobenzoylchloride (39 mg, 0.178 mmol, 4 equiv.) and DMAP (5.5 mg, 0.045 mmol, 1 equiv.). The mixture was stirred at room temperature for 18 h and then quenched with water (5 mL). The mixture was extracted with CH_2Cl_2 (4 × 5 mL) and the combined organic phases were successively washed with aqueous 1M HCl solution (2 × 10 mL) and brine (10 mL). The organic solution was dried over $MgSO_4$, filtered, and concentrated under reduced pressure. The obtained crude was purified by flash column chromatography (silica gel, EtOAc/hexanes, 1:4). The resulting product was recrystallized from acetone/heptane to give **47** as colorless crystals (19.6 mg, 0.036 mmol, 80% yield). R_f = 0.31 (silica,

EtOAc/hexanes, 1:4); m.p. = 183 – 190 °C; $[\alpha]_D^{25}$ = 8.9 (*c* 0.1, $CHCl_3$); IR (ATR, ZnSe) ν 2957, 2833, 1734, 1398, 1244, 1074, 993 cm^{-1}; ^{1}H NMR (500 MHz, $CDCl_3$) δ 7.88 – 7.78 (m, 4H, Ar), 7.65 – 7.54 (m, 4H, Ar), 6.66 (t, $^3J_{H1-H2} = {}^3J_{H1-F2}$ = 6.8 Hz, 1H, H1), 5.20 – 4.85 (m, 3H, H2, H3, H4), 4.65 – 4.58 (m, 2H, H6a, H6b), 4.33 (dt, $^3J_{H5-F4}$ = 27.5 Hz, $^3J_{H5-H6a} = {}^3J_{H5-H6b}$ = 6.6 Hz, 1H, H5) ppm; ^{13}C NMR (126 MHz, $CDCl_3$) δ 164.5, 163.0 (2C, 2 × *C*OAr), 132.4, 132.0, 131.4, 131.3, 129.8, 129.2, 128.9, 128.2 (12C, Ar), 103.7 – 83.1 (m, 4C, C1, C2, C3, C4), 69.8 (m, 1C, C5), 62.2 (m, 1C, C6) ppm; ^{19}F NMR (470 MHz, $CDCl_3$) δ –205.25 (dtdd, $^2J_{F2-H2}$ = 49.4 Hz, $^3J_{F2-H3} = {}^3J_{F2-F4}$ = 28.7 Hz, $^3J_{F2-F3}$ = 13.6 Hz, $^3J_{F2-H1}$ = 7.4 Hz, 1F, F2), –207.96 (dtq, $^2J_{F3-H3}$ = 42.6 Hz, $^3J_{F3-F2} = {}^3J_{F3-F4}$ = 11.9, $^3J_{F3-H2} = {}^3J_{F3-H4} = {}^4J_{F3-H1}$ = 5.8 Hz, 1F, F3), –219.53 (dqd, $^2J_{F4-H4}$ = 50.3 Hz, $^3J_{F4-H3} = {}^3J_{F4-H5} = {}^4J_{F4-F2}$ = 28.3 Hz, $^3J_{F4-F3}$ = 12.4 Hz, 1F, F4) ppm; HRMS calcd for $C_{20}H_{15}O_5F_3Br_2Na^+$ $[M + Na]^+$ 574.9111, found 574.9101.

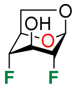

49

1,6-Anhydro-2,4-dideoxy-2,4-difluoro-α-D-glucopyranose (49). To a flask containing compound **48** (23.5 g, 50.0 mmol) was added KHF_2 (15.6 g, 200 mmol, 4 equiv.) and TBAF·$3H_2O$ (126.2 g, 400 mmol, 8 equiv.). The mixture was heated at 180 °C for 24 h. After cooling to room temperature, the reaction was dissolved in water (500 mL) and extracted with EtOAc (3 × 500 mL). The combined organic phases were washed with brine (1 L), dried over MgSO4, filtered and concentrated under reduced pressure. The crude mixture was then dissolved in CH_3CN (800 mL) and washed with hexane (3 × 300 mL). The organic solution was concentrated under reduced pressure and the obtained crude was purified by flash column chromatography (silica gel, EtOAc/$CHCl_3$, 1:4 → 1:1) to give **49** as a white crystal forming needles from $CHCl_3$ (4.96 g, 29.9 mmol, 60% yield). R_f = 0.21 (silica, EtOAc/hexanes, 2:3); m.p. = 95 – 98 °C; $[\alpha]_D^{25}$ = –45.6 (*c* 0.2, $CHCl_3$); IR (ATR, ZnSe) ν 3450, 2962, 2924, 1333, 1148, 1016, 876 cm^{-1}; ^{1}H NMR (500 MHz, $CDCl_3$) δ 5.60 (dt, $^3J_{H1-F2}$ = 3.4 Hz, $^3J_{H1-H2} = {}^5J_{H1-F4}$ = 1.4 Hz, 1H, H1), 4.77 (ddq, $^3J_{H5-F4}$ = 12.8 Hz, $^3J_{H5-H6b}$ = 5.7 Hz, $^3J_{H5-H6a} = {}^3J_{H5-H4} = {}^5J_{H5-F2}$ = 1.4 Hz, 1H, H5), 4.44 (dddd, $^2J_{H4-F4}$ = 46.4 Hz, $^3J_{H4-H3}$ = 2.6 Hz, $^3J_{H4-H5}$ = 1.6 Hz, $^4J_{H4-F2}$ = 1.0 Hz, 1H, H4), 4.29 (ddt, $^2J_{H2-F2}$ = 46.4 Hz, $^3J_{H2-H3}$ = 2.6 Hz, $^3J_{H2-H1} = {}^4J_{H2-F4}$ = 1.3 Hz, 1H, H2), 4.11 (br t, $^3J_{H3-F2} = {}^3J_{H3-F4}$ = 18.0 Hz, 1H, H3), 4.03 (dt, $^2J_{H6a-H6b}$ = 7.9 Hz, $^3J_{H6a-H5} = {}^4J_{H6a-F4}$ = 1.1 Hz, 1H, H6a), 3.79 (dddt, $^2J_{H6b-H6a}$ = 7.9 Hz, $^3J_{H6b-H5}$ = 5.3 Hz, $^4J_{H6b-F4}$ = 4.7 Hz, $^4J_{H6b-H4} = {}^4J_{H6b-H1}$ = 0.6 Hz, 1H, H6b), 2.27 (br s, 1H, OH) ppm; ^{13}C NMR (126 MHz, $CDCl_3$) δ 99.5 (d, $^2J_{C1-F2}$ = 29.0, 1C, C1), 90.0 (dd, $^1J_{C4-F4}$ = 182.4 Hz, $^3J_{C4-F2}$ = 5.5 Hz, 1C, C4), 88.1 (dd, $^1J_{C2-F2}$ =

184.6 Hz, $^{3}J_{C2\text{-}F4}$ = 4.3 Hz, 1C, C2), 74.5 (d, $^{2}J_{C5\text{-}F4}$ = 22.4 Hz, 1C, C5), 69.6 (dd, $^{2}J_{C3\text{-}F2}$ = 29.3 Hz, $^{2}J_{C3\text{-}F4}$ = 28.2 Hz, 1C, C3), 64.9 (d, $^{3}J_{C6\text{-}F4}$ = 9.5 Hz, 1C, C6) ppm; ^{19}F NMR (470 MHz, $CDCl_3$) δ −183.96 (dddd, $^{2}J_{F4\text{-}H4}$ = 46.1 Hz, $^{3}J_{F4\text{-}H3}$ = 17.0 Hz, $^{3}J_{F4\text{-}H5}$ = 12.9 Hz, $^{4}J_{F4\text{-}H6b}$ = 4.4 Hz, 1F, F4), −189.00 (ddd, $^{2}J_{F2\text{-}H2}$ = 46.4 Hz, $^{3}J_{F2\text{-}H3}$ = 18.5 Hz, $^{3}J_{F2\text{-}H1}$ = 4.4 Hz, 1F, F2) ppm; HRMS calcd for $C_6H_9O_3F_2^+$ $[M + H]^+$ 167.0514, found 167.0504.

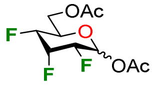

11

1,6-Di-*O*-acetyl-2,3,4-trideoxy-2,3,4-trifluoro-α/β-D-allopyranose (11). To a solution of **49** (45.7 mg, 0.275 mmol) in CH_2Cl_2 (2.8 mL) was added pyridine (66.7 µL, 0.825 mmol, 3 equiv.) and a 1M Tf_2O solution in CH_2Cl_2 (0.413 mL, 0.413 mmol, 1.5 equiv.). The mixture was stirred at room temperature for 30 min and then quenched with a saturated aqueous $NaHCO_3$ solution (15 mL). The mixture was extracted with CH_2Cl_2 (3 × 10 mL) and the combined organic phases were successively washed with aqueous 1M HCl solution (15 mL) and brine (15 mL). The organic solution was dried over $MgSO_4$, filtered, and concentrated under reduced pressure. The crude triflate was used for the next step without further purification and dissolved in $Et_3N{\cdot}3HF$ (4.5 mL, 27.5 mmol, 100 equiv.). The mixture was heated at 120 °C for 20 h and then cooled to room temperature. The reaction was then quenched with water (20 mL). The mixture was extracted with CH_2Cl_2 (3 × 15 mL) and the combined organic phases were successively washed with a saturated aqueous $NaHCO_3$ solution (1 × 20 mL), aqueous 1M HCl solution (1 × 20 mL) and brine (1 × 20 mL). The organic solution was dried over $MgSO_4$, filtered, and concentrated carefully with air flow because of volatility issues. The crude trifluoro intermediate **53** was used in the next step without further purification. Intermediate **53** was dissolved in Ac_2O (0.78 mL, 8.25 mmol, 30 equiv.) at 0 °C and H_2SO_4 (0.15 mL, 2.75 mmol, 10 equiv.) was added. The mixture was stirred at room temperature for 16 h. After cooling to 0 °C, NaOAc (451 mg, 5.5 mmol, 20 equiv.) was added. The mixture was stirred for 20 min and then quenched with water (20 mL), and the mixture was extracted with CH_2Cl_2 (4 × 15 mL). The combined organic phases were successively washed with a saturated aqueous $NaHCO_3$ solution (1 × 30 mL), aqueous 1M HCl solution (1 × 30 mL) and brine (1 × 30 mL). The organic solution was dried over $MgSO_4$, filtered, and concentrated under reduced pressure. The obtained crude was purified by flash column chromatography (silica gel, EtOAc/hexanes, 2:8 → 3:7) to give an anomeric mixture (α/β, 1:1.7) of **11** as a white solid (25.2 mg, 0.0933 mmol, 34 % yield over 3 steps). The anomeric

mixture was purified again by flash column chromatography (silica gel, $Et_2O/CHCl_3$, 0:1 → 1:9) to give a pure fraction of the β anomer that was used for characterization. $R_{f\alpha}$ = 0.24 (silica, EtOAc/hexanes, 3:7); $R_{f\alpha}$ = 0.27 (silica, $Et_2O/CHCl_3$, 1:9); $R_{f\beta}$ = 0.26 (silica, EtOAc/hexanes, 3:7); $R_{f\beta}$ = 0.35 (silica, $Et_2O/CHCl_3$, 1:9); m.p. = 110 – 111 °C (acetone/heptane); $[\alpha]_D^{25}$ = –44.6 (*c* 0.6, $CHCl_3$); IR (ATR, ZnSe) ν 2920, 1774, 1732, 1378, 1251, 1202, 1056, 877 cm^{-1}; ^{1}H NMR (500 MHz, $CDCl_3$) δ 6.05 (ddd, $^{3}J_{H1-H2}$ = 8.2 Hz, $^{3}J_{H1-F2}$ = 1.9 Hz, $^{4}J_{H1-F3}$ = 1.2 Hz, 1H, H1), 5.36 (dtt, $^{2}J_{H3-F3}$ = 54.3 Hz, $^{3}J_{H3-F2}$ = $^{3}J_{H3-F4}$ = 8.9 Hz, $^{3}J_{H3-H2}$ = $^{3}J_{H3-H4}$ = 2.2 Hz, 1H, H3), 4.54 (dddt, $^{2}J_{H4-F4}$ = 45.3 Hz, $^{3}J_{H4-F3}$ = 25.3 Hz, $^{3}J_{H4-H5}$ = 9.3 Hz, $^{3}J_{H4-H3}$ = $^{4}J_{H4-F2}$ = 1.9 Hz, 1H, H4), 4.43 – 4.39 (m, 1H, H6a), 4.41 (dddddd, $^{2}J_{H2-F2}$ = 46.0 Hz, $^{3}J_{H2-F3}$ = 25.9 Hz, $^{3}J_{H2-H1}$ = 8.2 Hz, $^{3}J_{H2-H3}$ = 2.3 Hz, $^{4}J_{H2-F4}$ = 1.6 Hz, $^{4}J_{H2-H4}$ = 0.6 Hz, 1H, H2), 4.27 – 4.22 (m, 2H, H5, H6b), 2.17 (s, 3H, $COCH_3$), 2.10 (s, 3H, $COCH_3$) ppm; ^{13}C NMR (126 MHz, $CDCl_3$) δ 170.5, 168.9 (2C, 2 × $COCH_3$), 89.5 (dd, $^{2}J_{C1-F2}$ = 25.6 Hz, $^{3}J_{C1-F3}$ = 3.9 Hz, 1C, C1), 87.3 (dt, $^{1}J_{C3-F3}$ = 186.3Hz, $^{2}J_{C3-F2}$ = $^{2}J_{C3-F4}$ = 17.6 Hz, 1C, C3), 85.5 (ddd, $^{1}J_{C2-F2}$ = 197.9 Hz, $^{2}J_{C2-F3}$ = 16.9 Hz, $^{3}J_{C2-F4}$ = 5.6 Hz, 1C, C2), 83.6 (ddd, $^{1}J_{C4-F4}$ = 194.9 Hz, $^{2}J_{C4-F3}$ = 17.6 Hz, $^{3}J_{C4-F2}$ = 5.6 Hz, 1C, C4), 69.9 (dd, $^{2}J_{C5-F4}$ = 25.1 Hz, $^{3}J_{C5-F3}$ = 3.4 Hz, 1C, C5), 61.9 (1C, C6), 20.94, 20.87 (2C, 2 × $COCH_3$) ppm; ^{19}F NMR (470 MHz, $CDCl_3$) δ –204.75 (ddddp, $^{2}J_{F4-H4}$ = 45.3 Hz, $^{3}J_{F4-F3}$ = 12.8 Hz, $^{3}J_{F4-H3}$ = 9.2 Hz, $^{4}J_{F4-F2}$ = 3.4 Hz, $^{3}J_{F4-H5}$ = $^{4}J_{F4-F2}$ = $^{4}J_{F4-H6a}$ = $^{4}J_{F4-H6b}$ = 1.9 Hz, 1F, F4), –205.03 (ddddt, $^{2}J_{F2-H2}$ = 46.0 Hz, $^{3}J_{F2-F3}$ = 14.2 Hz, $^{3}J_{F2-H3}$ = 8.9 Hz, $^{4}J_{F2-F4}$ = 3.8 Hz, $^{3}J_{F2-H1}$ = $^{4}J_{F2-H4}$ = 1.9 Hz, 1F, F2), –217.87 (dtt, $^{2}J_{F3-H3}$ = 54.3 Hz, $^{3}J_{F3-H2}$ = $^{3}J_{F3-H4}$ = 25.6 Hz, $^{3}J_{F3-F2}$ = $^{3}J_{F3-F4}$ = 13.9 Hz, 1F, F3) ppm; HRMS calcd for $C_{10}H_{17}O_5F_3N^+$ $[M + NH_4]^+$ 288.1053, found 288.1047.

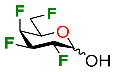

54

2,3,4,6-tetradeoxy-2,3,4,6-tetrafluoro-α/β-D-galactopyranose (54). This compound prove to be highly difficult to isolate due to its high volatility. Compound **54** was isolated as a colorless oil. ^{1}H NMR (500 MHz, Chloroform-*d*) δ 5.56 (t, $^{3}J_{H1-H2}$ = $^{3}J_{H1-F2}$ = 4.2 Hz, 1H, H1α), 5.13 – 4.68 (m, 7H, H1β, H2α, H2β, H3α, H3β, H4α, H4β), 4.62 (ddd, $^{2}J_{H6a-F6}$ = 46.0 Hz, $^{2}J_{H6a-H6b}$ = 9.4 Hz, $^{3}J_{H6a-H5}$ = 6.4 Hz, 1H, H6aα), 4.69 – 4.56 (m, 2H, H6aβ, H6bβ), 4.57 (ddd, $^{2}J_{H6b-F6}$ = 46.3 Hz, $^{2}J_{H6b-H6a}$ = 9.3 Hz, $^{3}J_{H6b-H5}$ = 6.4 Hz, 1H, H6bα), 4.38 (ddt, $^{3}J_{H5-F4}$ = 28.9 Hz, $^{3}J_{H5-F6}$ = 11.6 Hz, $^{3}J_{H5-H6a}$ = $^{3}J_{H5-H6b}$ = 6.3 Hz, 1H, H5α), 3.89 – 3.80 (m, 1H, H5β), 3.50 (br s, 1H, OHα) ppm; ^{19}F NMR (470 MHz, Chloroform-*d*) δ –202.44 (ddtd, $^{2}J_{F3-H3}$ = 45.6 Hz, $^{3}J_{F3-F4}$ = 15.5 Hz, $^{3}J_{F3-H2}$ = $^{3}J_{F3-F2}$ = 13.2 Hz, $^{3}J_{F3-H4}$ = 6.7 Hz, 1F, F3β), –207.69 (dtt, $^{2}J_{F2-H2}$ = 51.3 Hz,

$^3J_{F2-H3}$ = $^3J_{F2-F3}$ = 13.9 Hz, $^3J_{F2-H1}$ = $^4J_{F2-F4}$ = 2.6 Hz, 1F, F2β), –208.01 – –208.27 (m, 1F, F3α), –209.00 (dtd, $^2J_{F2-H2}$ = 50.8 Hz, $^3J_{F2-H3}$ = $^3J_{F2-F3}$ = 13.3 Hz, $^3J_{F2-H1}$ = 3.8 Hz, 1F, F2α), –217.69 (dtd, $^2J_{F4-H4}$ = 50.9 Hz, $^3J_{F4-H3}$ = $^3J_{F4-H5}$ = 25.8 Hz, $^3J_{F4-F3}$ = 14.9 Hz, 1F, F4β), –220.21 (dtd, $^2J_{F4-H4}$ = 50.4 Hz, $^3J_{F4-H3}$ = $^3J_{F4-H5}$ = 27.8 Hz, $^3J_{F4-F3}$ = 14.1 Hz, 1F, F4α), –231.42 (td, $^2J_{F6-H6a}$ = $^2J_{F6-H6b}$ = 45.4 Hz, $^3J_{F6-H5}$ = 9.5 Hz, 1F, F6β), –231.42 (td, $^2J_{F6-H6a}$ = $^2J_{F6-H6b}$ = 46.4 Hz, $^3J_{F6-H5}$ = 11.2 Hz, 1F, F6α), –231.51 (td, $^2J_{F6-H6a}$ = $^2J_{F6-H6b}$ = 46.4 Hz, $^3J_{F6-H5}$ = 11.2 Hz, 1F, F6α) ppm; HRMS calcd for $C_6H_7O_2F_4^-$ [M - H]$^-$ 187.0388, found 187.0404.

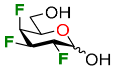

55

2,3,4-trideoxy-2,3,4-trifluoro-α/β-D-galactopyranose (55). To a stirred solution of compound **19** (22.4 mg, 0.083 mmol) in water (0.83 mL) at room temperature, was added an aqueous hydrochloric acid solution (37%) (1.8 mL). The mixture was stirred room temperature for 1 h and then evaporated with a gentle air flow. The obtained yellow crude was purified by flash column chromatography (silica gel, EtOAc/hexanes, 4:1) to give pure product **55** (α/β, 1:1.9) as a pale yellow thick oil (15.1 mg, 0.081 mmol, 98% yield). This compound prove to be highly difficult to isolate due to its high volatility. R_f = 0.41 (silica, EtOAc/hexanes, 4:1); $[\alpha]_D^{25}$ = 58.9 (c 0.6, MeOH); IR (ATR, diamond crystal) ν 3337, 2957, 1367, 1148, 1036, 824, 796 cm^{-1}; ^{1}H NMR (500 MHz, Acetone-d_6) δ 5.47 (t, $^3J_{H1-H2}$ = $^3J_{H1-F2}$ = 4.4 Hz, 1H, H1α), 5.18 (ddtd, $^2J_{H4-F4}$ = 51.1 Hz, $^3J_{H4-F3}$ = 7.9 Hz, $^3J_{H4-H3}$ = 3.6 Hz, $^3J_{H4-H5}$ = 3.1 Hz, $^4J_{H4-H2}$ = 0.7 Hz, 1H, H4α), 5.19 – 4.90 (m, 4H, H4β), 4.89 (ddd, $^3J_{H1-H2}$ = 7.6 Hz, $^3J_{H1-F2}$ = 3.5 Hz, $^3J_{H1-OH}$ = 1.1 Hz, 1H, H1β), 4.79 (ddddd, $^2J_{H2-F2}$ = 51.0 Hz, $^3J_{H2-F3}$ = 11.5 Hz, $^3J_{H2-H3}$ = 9.7 Hz, $^3J_{H2-H1}$ = 3.9 Hz, $^4J_{H2-H4}$ = 1.2 Hz, 1H, H2α), 4.47 (ddddd, $^2J_{H2-F2}$ = 52.5 Hz, $^3J_{H2-F3}$ = 13.3 Hz, $^3J_{H2-H3}$ = 8.9 Hz, $^3J_{H2-H1}$ = 7.8 Hz, $^4J_{H2-H4}$ = 1.1 Hz, 1H, H2β), 4.16 (dddd, $^3J_{H5-F4}$ = 29.7 Hz, $^3J_{H5-H6a}$ = 7.6 Hz, $^3J_{H5-H6b}$ = 6.7 Hz, $^3J_{H5-H4}$ = 1.7 Hz, 1H, H5α), 3.79 (dddd, $^3J_{H5-F4}$ = 26.7 Hz, $^3J_{H5-H6a}$ = 7.7 Hz, $^3J_{H5-H6b}$ = 5.9 Hz, $^3J_{H5-H4}$ = 1.9 Hz, 1H, H5β), 3.74 – 3.68 (m, 3H, H6aα, H6bα, H6aβ, H6bβ), 3.65 (ddt, $^2J_{H6b-H6a}$ = 10.7 Hz, $^3J_{H6b-H5}$ = 6.3 Hz, $^3J_{H6b-OH}$ = 1.6 Hz, 1H, H6bα) ppm; ^{13}C NMR (126 MHz, Acetone-d_6) δ 95.0 (dd, $^2J_{C1-F2}$ = 22.5 Hz, $^3J_{C1-F3}$ = 10.5 Hz, 1C, C1β), 91.8 (dd, $^1J_{C2-F2}$ = 184.0 Hz, $^2J_{C2-F3}$ = 18.0 Hz, 1C, C2β), 91.4 (dd, $^2J_{C1-F2}$ = 20.9 Hz, $^3J_{C1-F3}$ = 9.5 Hz, 1C, C1α), 90.6 (ddd, $^1J_{C3-F3}$ = 188.9 Hz, $^2J_{C3-F2}$ = 19.1 Hz, $^2J_{C3-F4}$ = 17.5 Hz, 1C, C3β), 88.8 (ddd, $^1J_{C4-F4}$ = 181.7 Hz, $^2J_{C4-F3}$ = 16.0 Hz, $^3J_{C4-F2}$ = 8.3 Hz, 1C, C4α), 88.2 (ddd, $^1J_{C3-F3}$ = 187.3 Hz, $^2J_{C3-F2}$ = 18.7 Hz, $^2J_{C3-F4}$ = 17.6 Hz, 1C, C3α), 88.0 (ddd, $^1J_{C4-F4}$ = 181.9 Hz, $^2J_{C4-F3}$ = 15.9 Hz, $^3J_{C4-F2}$ = 9.2 Hz, C4β), 87.8

(ddd, $^{1}J_{C2\text{-}F2}$ = 187.5 Hz, $^{2}J_{C2\text{-}F3}$ = 17.3 Hz, $^{3}J_{C2\text{-}F4}$ = 2.2 Hz, 1C, C2α), 73.4 (dd, $^{2}J_{C5\text{-}F4}$ = 18.0 Hz, $^{3}J_{C5\text{-}F3}$ = 5.3 Hz, 1C, C5β), 69.6 (ddd, $^{2}J_{C5\text{-}F4}$ = 18.1 Hz, $^{3}J_{C5\text{-}F3}$ = 4.7 Hz, $^{4}J_{C5\text{-}F2}$ = 0.9 Hz, 1C, C5α), 60.04 (dd, $^{3}J_{C6\text{-}F4}$ = 5.9 Hz, $^{4}J_{C6\text{-}F3}$ = 2.1 Hz, 1C, C6α), 59.98 (dd, $^{3}J_{C6\text{-}F4}$ = 5.9 Hz, $^{4}J_{C6\text{-}F3}$ = 2.6 Hz, 1C, C6β) ppm; ^{19}F NMR (470 MHz, Acetone-d_6) δ –202.71 (dqd, $^{2}J_{F3\text{-}H3}$ = 48.6 Hz, $^{3}J_{F3\text{-}H2}$ = $^{3}J_{F3\text{-}F2}$ = $^{3}J_{F3\text{-}F4}$ = 14.1 Hz, $^{3}J_{F3\text{-}H4}$ = 6.8 Hz, 1F, F3β), –207.69 (dtt, $^{2}J_{F2\text{-}H2}$ = 52.5 Hz, $^{3}J_{F2\text{-}H3}$ = $^{3}J_{F2\text{-}F3}$ = 14.1 Hz, $^{3}J_{F2\text{-}H1}$ = $^{4}J_{F2\text{-}F4}$ = 3.4 Hz, 1F, F2β), –207.90 – –208.14 (m, 1F, F3α), –208.74 (ddddd, $^{2}J_{F2\text{-}H2}$ = 51.3 Hz, $^{3}J_{F2\text{-}F3}$ = 13.1 Hz, $^{3}J_{F2\text{-}H3}$ = 11.8 Hz, $^{3}J_{F2\text{-}H1}$ = 3.5 Hz, $^{4}J_{F2\text{-}F4}$ = 1.4 Hz, 1F, F2α), –219.71 (dtdd, $^{2}J_{F4\text{-}H4}$ = 50.8 Hz, $^{3}J_{F4\text{-}H3}$ = $^{3}J_{F4\text{-}H5}$ = 27.1 Hz, $^{3}J_{F4\text{-}F3}$ = 15.2 Hz, $^{4}J_{F4\text{-}F2}$ = 2.6 Hz, 1F, F4β), –222.51 (dtdd, $^{2}J_{F4\text{-}H4}$ = 51.1 Hz, $^{3}J_{F4\text{-}H3}$ = $^{3}J_{F4\text{-}H5}$ = 28.0 Hz, $^{3}J_{F4\text{-}F3}$ = 15.0 Hz, $^{4}J_{F4\text{-}F2}$ = 1.9 Hz, 1F, F4α) ppm; HRMS calcd for $C_6H_8O_3F_3^-$ [M - H]$^-$ 185.0431, found 185.0426.

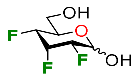

56

2,3,4-trideoxy-2,3,4-trifluoro-α/β-D-allopyranose (56). To a stirred solution of compound **11** (12.1 mg, 0.045 mmol) in water (0.45 mL) at room temperature, was added an aqueous hydrochloric acid solution (37%) (1.0 mL). The mixture was stirred at room temperature for 4 h and then evaporated with a gentle air flow. The obtained yellow crude was purified by flash column chromatography (silica gel, EtOAc/hexanes, 4:1) to give pure product **56** as a white amorphous solid (7.9 mg, 0.042 mmol, 95% yield). This compound prove to be highly difficult to isolate due to its high volatility. R_f = 0.36 (silica, EtOAc/hexanes, 4:1); $[\alpha]_D^{25}$ = 4.9 (c 0.4, MeOH); IR (ATR, diamond crystal) ν 3323, 2947, 1366, 1144, 1016, 914, 723 cm^{-1}; ^{1}H NMR (500 MHz, Acetone-d_6) δ 6.46 (br s, 1H, OH), 5.40 (dtt, $^{2}J_{H3\text{-}F3}$ = 56.0 Hz, $^{3}J_{H3\text{-}F2}$ = $^{3}J_{H3\text{-}F4}$ = 9.5 Hz, $^{3}J_{H3\text{-}H2}$ = $^{3}J_{H3\text{-}H4}$ = 2.3 Hz, 1H, H3), 5.07 (dt, $^{3}J_{H1\text{-}H2}$ = 8.0 Hz, $^{3}J_{H1\text{-}F2}$ = $^{4}J_{H1\text{-}F3}$ = 1.4 Hz, 1H, H1), 4.75 (dddddd, $^{2}J_{H4\text{-}F4}$ = 45.1 Hz, $^{3}J_{H4\text{-}F3}$ = 26.9 Hz, $^{3}J_{H4\text{-}H5}$ = 9.7 Hz, $^{3}J_{H4\text{-}H3}$ = 2.1 Hz, $^{4}J_{H4\text{-}F2}$ = 1.6 Hz, $^{4}J_{H4\text{-}H2}$ = 0.5 Hz, 1H, H4), 4.32 (dddddd, $^{2}J_{H2\text{-}F2}$ = 45.9 Hz, $^{3}J_{H2\text{-}F3}$ = 27.1 Hz, $^{3}J_{H2\text{-}H1}$ = 7.9 Hz, $^{3}J_{H2\text{-}H3}$ = 2.3 Hz, $^{4}J_{H2\text{-}F4}$ = 1.6 Hz, $^{4}J_{H2\text{-}H4}$ = 0.6 Hz, 1H, H2), 3.98 (br s, 1H, OH), 3.86 (ddq, $^{3}J_{H5\text{-}H4}$ = 9.7 Hz, $^{3}J_{H5\text{-}F4}$ = 3.8 Hz, $^{3}J_{H5\text{-}H6a}$ = $^{3}J_{H5\text{-}H6b}$ = $^{4}J_{H5\text{-}F3}$ = 1.9 Hz, 1H, H5), 3.83 (br d, $^{2}J_{H6a\text{-}H6b}$ = 11.8 Hz, 1H, H6a), 3.66 (br d, $^{2}J_{H6b\text{-}H6a}$ = 12.4 Hz, 1H, H6b) ppm; ^{13}C NMR (126 MHz, Acetone-d_6) δ 93.0 (dd, $^{2}J_{C1\text{-}F2}$ = 23.9 Hz, $^{3}J_{C1\text{-}F3}$ = 4.1 Hz, 1C, C1), 89.4 (dt, $^{1}J_{C3\text{-}F3}$ = 180.3 Hz, $^{2}J_{C3\text{-}F2}$ = $^{2}J_{C3\text{-}F4}$ = 17.2 Hz, 1C, C3), 88.9 (ddd, $^{1}J_{C4\text{-}F4}$ = 192.4 Hz, $^{2}J_{C4\text{-}F3}$ = 16.4 Hz, $^{3}J_{C4\text{-}F2}$ = 5.5 Hz, 1C, C4), 84.8 (ddd, $^{1}J_{C2\text{-}F2}$ = 188.8 Hz, $^{2}J_{C2\text{-}F3}$ = 16.9 Hz, $^{3}J_{C2\text{-}F4}$ = 5.2 Hz, 1C, C2), 72.6 (dd, $^{2}J_{C5\text{-}F4}$ = 24.3 Hz, $^{3}J_{C5\text{-}F3}$ = 2.5 Hz, 1C,

C5), 61.1 (1C, C6) ppm; ^{19}F NMR (470 MHz, Acetone-d_6) δ –202.34 (dddtt, $^2J_{F4\text{-}H4}$ = 44.8Hz, $^3J_{F4\text{-}F3}$ = 11.9 Hz, $^3J_{F4\text{-}H3}$ = 10.2 Hz, $^3J_{F4\text{-}H5}$ = $^4J_{F4\text{-}F2}$ = 3.7 Hz, $^4J_{F4\text{-}H2}$ = $^4J_{F4\text{-}H6}$ = 1.9 Hz, 1F, F4α), –202.69 (br d, $^2J_{F2\text{-}H2}$ = 44.5 Hz, 1F, F2α), –203.26 (ddddt, $^2J_{F2\text{-}H2}$ = 45.7 Hz, $^3J_{F2\text{-}F3}$ = 14.5 Hz, $^3J_{F2\text{-}H3}$ = 9.5 Hz, $^4J_{F2\text{-}F4}$ = 4.3 Hz, $^3J_{F2\text{-}H1}$ = $^4J_{F2\text{-}H4}$ = 1.9 Hz, 1F, F2β), –205.39 (dttt, $^2J_{F4\text{-}H4}$ = 45.2 Hz, $^3J_{F4\text{-}H3}$ = $^3J_{F4\text{-}F3}$ = 11.4 Hz, $^3J_{F4\text{-}H5}$ = $^4J_{F4\text{-}F2}$ = 3.5 Hz, $^4J_{F4\text{-}H2}$ = $^4J_{F4\text{-}H6}$ = 1.7 Hz, 1F, F4β), –215.77 (dddt, $^2J_{F3\text{-}H3}$ = 55.1 Hz, $^3J_{F3\text{-}H2}$ = 29.5 Hz, $^3J_{F3\text{-}H4}$ = 25.1 Hz, $^3J_{F3\text{-}F2}$ = $^3J_{F3\text{-}F4}$ = 12.5 Hz, 1F, F3α), –217.90 (dttt, $^2J_{F3\text{-}H3}$ = 56.0 Hz, $^3J_{F3\text{-}H2}$ = $^3J_{F3\text{-}H4}$ = 26.7 Hz, $^3J_{F3\text{-}F2}$ = 14.7 Hz, $^3J_{F3\text{-}F4}$ = 13.5 Hz, $^4J_{F3\text{-}H5}$ = $^4J_{F3\text{-}H1}$ = 1.4 Hz, 1F, F3β) ppm; HRMS calcd for $C_6H_{13}O_3NF_3^+$ $[M + NH_4]^+$ 204.0842, found 204.0847.

Supplementary Discussion

Density functional theory (DFT) calculations were performed with the CAM-B3LYP functional using Grimme's D3 correction and the 6-31+G(d,p) basis set. Gaussian 09 rev E.01 was used was used for all calculations. To study possible solvent effects, we employed the polarizable continuum model (PCM) specifically for acetone. While all calculations in the main body employed PCM, those that do so in the supporting information will be clearly labelled.

A scan of the H_5-C_5-C_6-F_6 dihedral was performed for molecule **27**. Three stable structures were found, the least stable of which is that observed in the crystal structure (GG conformer). Their relative stabilities are reported in **Supplementary Table 1**. In vacuum, the most stable conformer is TG while in PCM the most stable is GT. Thermochemistry and dipole moments for the optimized structures of each conformer in vacuum and in PCM are summarized in **Supplementary Table 2** and **Supplementary Table 3** respectively. Optimized structures for all three conformers in vacuum and in PCM are in the Supplementary Table section (**Supplementary Table 4–Supplementary Table 9**).

The implicit solvation model predicts that the GT conformer is the most stable, though the TG conformer is very close in terms of free energy. This difference is small enough that both conformers might be observed at room temperature. The dipole moments of the molecules in PCM are larger, though the trend does not change.

To understand the arrangement in the solid state we studied monomers and small clusters of repeat units of the three staggered conformers (**Supplementary Figure 1**) and data are summarized in **Supplementary Table 10**. In the calculations that follow, the molecules are in the geometry of the crystal structure with the fluoromethyl group rotated to adopt the three conformers.

The TG conformer and small clusters of the TG conformer are the most stable, but the gap shrinks by more than a kcal/mol already in the structure with 3 repeated units. The GG conformer benefits most with repeating units. This becomes clearer if we look at a stabilization energy, which we define as the benefit of each structure from being in the cluster. Numerically, it is the difference between the energy of the cluster minus the energy of the isolated individual units. The stabilization energy of the Dimer of the GG conformer (entry 4) is substantially larger than that of the dimer of the GT conformer (entry 5) and the dimer of the GG conformer (6). Similarly, the stabilization energy of the trimer of the GG conformer (entry 7) is also much larger than the other 2 trimers (entry 8 and 9). Further, the stabilization of trimer of

the GG conformer (entry 7) is more than twice that the dimer of the same conformer (entry 4), while the others are about twice.

The orientation of the fluorine atom at C6 has a large effect on the molecular dipole. We performed analysis of the three staggered conformers of molecule **27**, along with small clusters of the GG conformer (**Supplementary Table 11**).

In the crystal structure, a number of hydrogen fluorine distances are potential hydrogen bonds. We performed a Natural Bonding Orbital (NBO) analysis which effectively ruled out this possibility. Specifically, in the GG conformer of molecule **27**, the dimer of the GG conformer, and the trimer of the GG conformer we looked at the NBO populations of the lone pairs on the fluorine centres (donors), as well as the C-H antibonding orbitals (acceptors). The results are presented in **Supplementary Table 12**. Off-diagonal Fock matrix energies in the NBO basis are reported in **Supplementary Table 13**. If hydrogen bonding were present, an appreciable portion of lone pair NBO population would be donated to antibonding pairs, and the off-diagonal Fock matrix elements would be reasonably large. Looking at the results, it is clear that this is not the case. The NBO populations are essentially constant and the off-diagonal Fock energies are small. The population of σ^{*}_{C3H3} does increase, though not appreciably. Other hydrogen bonds have shown increases in population of around 0.03.[2]

Supplementary Tables

Supplementary Table 1. Relative energy of the dihedral scan of the H_5-C_5-C_6-F_6 dihedral angle for molecule **27**.

Entry	HCCF dihedral (°)	Relative E (kcal/mol)	Relative E in PCM (kcal/mol)	Conformer
**1**	**-179,34**	**4,43**	**1.52**	**GG**
2	-164,34	5,17	2.14	
3	-149,34	6,70	3.72	
4	-134,34	8,20	5.46	
5	-119,34	8,52	6.18	
6	-104,34	7,19	5.27	
7	-89,34	4,83	3.29	
8	-74,34	2,51	1.29	
9	-59,34	1,03	0.09	
**10**	**-44,34**	**0,68**	**0.00**	**GT**
11	-29,34	1,27	0.82	
12	-14,35	2,13	1.90	
13	0,66	2,53	2.50	
14	15,65	2,10	2.25	
15	30,66	1,10	1.40	
16	45,65	0,19	0.50	
**17**	**60,66**	**0,00**	**0.19**	**TG**
18	75,66	0,78	0.70	
19	90,66	2,34	1.90	
20	105,65	4,17	3.30	
21	120,66	5,53	4.21	
22	135,66	5,88	4.08	
23	150,66	5,33	3.10	
24	165,66	4,61	1.98	
25	180,66	4,43	1.52	

Supplementary Table 2. Thermochemistry for conformers of molecule **27** in vacuum (CAM-B3LYP-D3/6-31+G(d,p)).

Entry	Conformer	Energy (kcal/mol)	Enthalpy (kcal/mol)	Gibbs' Free Energy (kcal/mol)	Dipole Moment (D)
1	GG	4.44	4.33	4.42	3.6439
2	GT	0.70	0.64	0.70	2.3735
3	TG	0.00	0.00	0.00	1.2847

Supplementary Table 3. Thermochemistry for conformers of molecule **27** in PCM (CAM-B3LYP-D3/6-31+G(d,p)).

Entry	Conformer	Energy (kcal/mol)	Enthalpy (kcal/mol)	Gibbs' Free Energy (kcal/mol)	Dipole Moment (D)
1	GG	1.62	1.54	1.41	5.2859
2	GT	0.00	0.00	0.00	3.4897
3	TG	0.28	0.36	0.20	2.0293

Supplementary Table 4. Optimized cartesian coordinates of GG conformer in vacuum (CAM-B3LYP-D3/6-31+G(d,p)).

F	-1.831895	-2.750148	-0.543797
F	-4.499676	-1.804418	-0.428842
F	-4.036288	0.444465	1.015979
F	-2.936065	2.941619	1.092258
O	-1.359100	0.750628	0.161162
O	0.012628	-0.998028	0.633493
O	5.735596	1.349027	-0.957480
O	6.268222	-0.369796	0.376762
H	7.152379	-0.039532	0.153947
C	-1.007277	-0.568221	-0.197503
H	-0.684034	-0.599994	-1.252896
C	-2.183409	-1.522327	-0.000092
H	-2.383372	-1.655106	1.065510
C	-3.409060	-1.006197	-0.724568
H	-3.243459	-1.067836	-1.807253
C	-3.701760	0.430042	-0.326828
H	-4.557469	0.816748	-0.890472
C	-2.451818	1.274650	-0.569077
H	-2.239074	1.235101	-1.653843
C	-2.590652	2.743851	-0.231066
H	-1.634215	3.243984	-0.408169
H	-3.367768	3.194667	-0.857718
C	1.299560	-0.600911	0.381891
C	1.630253	0.491130	-0.418995
H	0.857154	1.112896	-0.850499
C	2.291595	-1.363519	0.996565
H	1.995609	-2.203665	1.613950
C	2.969319	0.799262	-0.617029
H	3.255146	1.643772	-1.234203
C	3.623030	-1.040808	0.800698
H	4.400314	-1.630105	1.272096
C	3.971428	0.041293	-0.014330
C	5.379679	0.423548	-0.260053

Supplementary Table 5. Optimized cartesian coordinates of GT conformer in vacuum (CAM-B3LYP-D3/6-31+G(d,p)).

F	-1.980494	-2.693733	-0.731309
F	-4.615560	-1.680539	-0.461730
F	-4.122617	0.441511	1.162159
F	-1.533536	3.486318	-0.064902
O	-1.418396	0.675668	0.393254
O	-0.059559	-1.135832	0.597172
O	5.650699	1.397436	-0.740154
O	6.194959	-0.567602	0.187003
H	7.076577	-0.212256	-0.005103
C	-1.085164	-0.583626	-0.148271
H	-0.779694	-0.474369	-1.203420
C	-2.282929	-1.524929	-0.047404
H	-2.473662	-1.772098	0.999655
C	-3.500103	-0.895544	-0.694168
H	-3.345688	-0.857730	-1.779750
C	-3.750684	0.509519	-0.177342
H	-4.579253	0.971598	-0.723535
C	-2.472125	1.332190	-0.287150
H	-2.224042	1.456334	-1.354366
C	-2.616611	2.705309	0.326992
H	-3.529474	3.195071	-0.024667
H	-2.616491	2.636538	1.416283
C	1.228564	-0.717444	0.371257
C	1.549190	0.514136	-0.197601
H	0.774012	1.226802	-0.448225
C	2.225107	-1.607513	0.767401
H	1.934647	-2.554347	1.207687
C	2.886541	0.835306	-0.388660
H	3.165644	1.786964	-0.826998
C	3.555034	-1.271230	0.582088
H	4.336769	-1.958201	0.883134
C	3.894299	-0.047890	-0.004845
C	5.300362	0.354436	-0.231774

Supplementary Table 6. Optimized cartesian coordinates of TG conformer in vacuum (CAM-B3LYP-D3/6-31+G(d,p)).

F	-1.752264	-2.715170	-0.802249
F	-4.433852	-1.854675	-0.487068
F	-3.976304	0.204180	1.242700
F	-3.666291	3.273848	-0.192138
O	-1.332045	0.653773	0.397407
O	0.089702	-1.109679	0.570040
O	5.723064	1.575932	-0.790376
O	6.323653	-0.310581	0.257855
H	7.195395	0.068555	0.065945
C	-0.959945	-0.587670	-0.164704
H	-0.665641	-0.451990	-1.219500
C	-2.112964	-1.585781	-0.081022
H	-2.282517	-1.874715	0.958744
C	-3.366770	-0.998940	-0.696050
H	-3.227876	-0.906272	-1.780313
C	-3.675001	0.363743	-0.107284
H	-4.545085	0.815980	-0.590578
C	-2.448415	1.260029	-0.228485
H	-2.250703	1.439185	-1.297774
C	-2.639647	2.596087	0.463860
H	-2.926751	2.454470	1.507217
H	-1.728204	3.193377	0.395876
C	1.362401	-0.651568	0.347162
C	1.650583	0.553801	-0.290839
H	0.855298	1.214293	-0.610258
C	2.384473	-1.474615	0.817309
H	2.121069	-2.403920	1.308913
C	2.977987	0.917958	-0.472580
H	3.231021	1.850324	-0.965029
C	3.703971	-1.096590	0.639882
H	4.504450	-1.731980	0.999370
C	4.009914	0.102089	-0.012782
C	5.403904	0.548704	-0.231674

Supplementary Table 7. Optimized cartesian coordinates of GG conformer in PCM (acetone) (CAM-B3LYP-D3/6-31+G(d,p)).

F	-1.882400	-2.759617	-0.539950
F	-4.529183	-1.766695	-0.387551
F	-4.041459	0.484636	1.015237
F	-2.813564	2.941571	1.130530
O	-1.355656	0.735046	0.165404
O	0.007054	-1.026632	0.606651
O	5.735774	1.320089	-0.979745
O	6.255884	-0.349392	0.419366
H	7.150724	-0.035094	0.210380
C	-1.016972	-0.578882	-0.218580
H	-0.702899	-0.604474	-1.273474
C	-2.205395	-1.512798	-0.007240
H	-2.396052	-1.638653	1.060821
C	-3.429001	-0.981468	-0.722597
H	-3.292707	-1.058039	-1.805837
C	-3.699199	0.461883	-0.338022
H	-4.546559	0.860619	-0.901154
C	-2.437662	1.286914	-0.573926
H	-2.221404	1.251348	-1.653817
C	-2.545977	2.753737	-0.227354
H	-1.598627	3.250428	-0.445966
H	-3.354780	3.216227	-0.798064
C	1.293446	-0.624141	0.358902
C	1.625390	0.440351	-0.478534
H	0.858723	1.034471	-0.957928
C	2.283430	-1.353003	1.018092
H	1.990622	-2.172512	1.664258
C	2.964793	0.753865	-0.665568
H	3.242519	1.577323	-1.313448
C	3.614700	-1.025090	0.830614
H	4.385770	-1.591620	1.338341
C	3.966541	0.030082	-0.019180
C	5.375850	0.411554	-0.253591

Supplementary Table 8. Optimized cartesian coordinates of GT conformer in PCM (acetone) (CAM-B3LYP-D3/6-31+G(d,p)).

F	-2.006534	-2.694518	-0.739264
F	-4.628424	-1.668664	-0.437857
F	-4.102387	0.430736	1.183306
F	-1.500016	3.478215	0.027069
O	-1.409883	0.677843	0.371991
O	-0.060848	-1.139961	0.556191
O	5.665882	1.337483	-0.825387
O	6.191972	-0.522208	0.305940
H	7.086594	-0.192299	0.121369
C	-1.092339	-0.577478	-0.185160
H	-0.797348	-0.471143	-1.240451
C	-2.294189	-1.509693	-0.064765
H	-2.473703	-1.751294	0.985309
C	-3.513710	-0.876814	-0.700046
H	-3.388722	-0.838974	-1.787088
C	-3.754222	0.524029	-0.169606
H	-4.590570	0.994681	-0.691661
C	-2.477511	1.347954	-0.287836
H	-2.245791	1.485216	-1.353900
C	-2.620894	2.706646	0.355145
H	-3.505288	3.223529	-0.023478
H	-2.661826	2.621258	1.441953
C	1.226464	-0.723412	0.335773
C	1.554435	0.453731	-0.335802
H	0.784787	1.125864	-0.691618
C	2.220018	-1.559134	0.845557
H	1.929883	-2.465063	1.365184
C	2.893777	0.774068	-0.511584
H	3.168697	1.684516	-1.031686
C	3.551510	-1.223827	0.672733
H	4.325621	-1.872523	1.064208
C	3.899230	-0.054839	-0.014236
C	5.308587	0.338610	-0.228060

Supplementary Table 9. Optimized cartesian coordinates of TG conformer in PCM (acetone) (CAM-B3LYP-D3/6-31+G(d,p)).

F	-1.804508	-2.736374	-0.791286
F	-4.461947	-1.822106	-0.442652
F	-3.977683	0.244172	1.238994
F	-3.626618	3.295878	-0.146012
O	-1.319522	0.630682	0.394101
O	0.089409	-1.144929	0.532124
O	5.721280	1.564820	-0.803232
O	6.314769	-0.285669	0.309889
H	7.196828	0.080721	0.133823
C	-0.965176	-0.604036	-0.190813
H	-0.682949	-0.463672	-1.245338
C	-2.132870	-1.581237	-0.084601
H	-2.294222	-1.856341	0.960092
C	-3.381910	-0.979156	-0.692725
H	-3.270219	-0.907339	-1.779353
C	-3.664638	0.394929	-0.117235
H	-4.525378	0.857271	-0.604807
C	-2.421991	1.267830	-0.236424
H	-2.216762	1.442743	-1.302118
C	-2.575709	2.603334	0.466726
H	-2.825760	2.466673	1.519750
H	-1.665544	3.195111	0.361320
C	1.360460	-0.678174	0.317243
C	1.646062	0.513191	-0.348417
H	0.853448	1.155613	-0.708450
C	2.383346	-1.477798	0.826794
H	2.126153	-2.396799	1.340763
C	2.972738	0.886424	-0.515580
H	3.214706	1.809091	-1.030494
C	3.701645	-1.090510	0.661812
H	4.498370	-1.710964	1.053689
C	4.007123	0.095118	-0.016626
C	5.401029	0.545841	-0.219042

Supplementary Table 10. Relative energies, enthalpies and Gibbs' Free energies of small clusters of conformers of molecule **27** in the crystal structure geometry.

Entry	Structure	Relative E (kcal/mol)	Relative H (kcal/mol)	Relative G (kcal/mol)	Stabilization E (kcal/mol)	Stabilization H (kcal/mol)	Stabilization G (kcal/mol)
1	Monomer of GG	4.09	3.90	4.17	na	na	na
2	Monomer of GT	0.94	0.84	1.01	na	na	na
3	Monomer of TG	0.00	0.00	0.00	na	na	na
4	Dimer of GG	4.03	3.77	4.86	-18.41	-18.56	-0.94
5	Dimer of GT	2.49	2.23	2.44	-13.65	-13.98	2.95
6	Dimer of TG	0.00	0.00	0.00	-14.26	-14.53	2.53
7	Trimer of GG	2.80	2.52	4.15	-38.65	-38.38	-4.63
8	Trimer of GT	3.72	3.40	3.73	-28.28	-28.33	4.42
9	Trimer of TG	0.00	0.00	0.00	-29.17	-29.21	3.73

Supplementary Table 11. Dipole moments of the three staggered conformers of molecule **27** and small cluster of the GG conformer obtained from CAM-B3LYP-D3/6-31+G(d,p).

Entry	Structure	X	Y	Z	Total
1	GG conformer	0.3090	1.3827	-3.2450	3.5408
2	GT conformer	-1.5921	0.7841	-0.6064	1.8754
3	TG conformer	1.3770	0.8778	-0.3476	1.6696
4	Dimer of the GG conformer	0.9527	-6.0767	4.9558	7.8990
5	Trimer of the GG conformer	-7.5315	7.6675	6.5430	12.5827

Supplementary Table 12. NBO populations of lone pairs of F3 and F4 (n_F) and antibonds σ^*_{CH}. NBO for conformer GG of molecule **27** and small clusters of the GG conformer.[a]

		Energy (kcal/mol)			
Entry	NBO populations	GG conformer	Dimer of the GG conformer	Trimer of the GG conformer	
1	n_{1F4}	1.99352	1.99296	1.99292	1.99295
2	n_{2F4}	1.97279	1.97256	1.97264	1.97339
3	n_{3F4}	1.96979	1.96964	1.96969	1.97005
4	n_{1F6}	1.99533	1.99363	1.99357	1.99359
5	n_{2F6}	1.97761	1.97762	1.97762	1.97762
6	n_{3F6}	1.97214	1.97322	1.97333	1.97366
7	σ^*_{C3H3}	0.02245	0.02951	0.02959	0.02951
8	σ^*_{C4H4}	0.02138	0.02175	0.02159	0.02165
9	σ^*_{C5H5}	0.02101	0.02163	0.02126	0.02149
10	σ^*_{C6H6}	0.01609	0.01628	0.0158	0.01625

[a]Carried out at CAM-B3LYP-D3/6-31+G(d,p).

Supplementary Table 13. Off-diagonal Fock matrix elements in NBO basis evaluated with second-order perturbation theory: $E^{(2)}$ (donor → acceptor).[a]

		Energy (kcal/mol)		
**Entry**	**$E^{(2)}$: n → σ^***	**Dimer of the GG conformer**	**Trimer of the GG conformer**	
1	$n_{1F4} \rightarrow \sigma^*_{C3H3}$	0.26	0.26	0.25
2	$n_{1F4} \rightarrow \sigma^*_{C4H4}$	0.21	0.21	0.21
3	$n_{2F4} \rightarrow \sigma^*_{C3H3}$	0.22	0.22	0.25
4	$n_{3F4} \rightarrow \sigma^*_{C3H3}$	0.13	0.14	0.11
5	$n_{3F4} \rightarrow \sigma^*_{C4H4}$	0.08	0.08	0.08
6	$n_{1F6} \rightarrow \sigma^*_{C4H4}$	0.73	0.75	0.72
7	$n_{1F6} \rightarrow \sigma^*_{C5H5}$	0.11	0.11	0.11
8	$n_{1F6} \rightarrow \sigma^*_{C6H6}$	0.22	0.23	0.23
9	$n_{2F6} \rightarrow \sigma^*_{C5H5}$	0.24	0.25	0.24
10	$n_{2F6} \rightarrow \sigma^*_{C6H6}$	0.08	0.08	0.08
11	$n_{3F6} \rightarrow \sigma^*_{C4H4}$	0.14	0.14	0.14
12	$n_{3F6} \rightarrow \sigma^*_{C6H6}$	--	0.05	0.05

[a]NBO carried out at CAM-B3LYP-D3/6-31+G(d,p).

Supplementary Table 14. Crystal data and structure refinement for **27**

Empirical formula	$C_{13}H_{12}O_4F_4$
Formula weight	308.23
Temperature/K	100
Crystal system	monoclinic
Space group	$P2_1$
a/Å	13.1007(10)
b/Å	4.5410(4)
c/Å	21.0852(16)
α/°	90
β/°	91.791(3)
γ/°	90
Volume/Å^3	1253.75(17)
Z	4
ρ_{calc}g/cm^3	1.633
μ/mm^{-1}	0.895
F(000)	632.0
Crystal size/mm^3	0.4 × 0.12 × 0.08
Radiation	GaKα (λ = 1.34139)
2Θ range for data collection/°	5.872 to 121.288
Index ranges	$-17 \leq h \leq 17$, $-5 \leq k \leq 5$, $-27 \leq l \leq 27$
Reflections collected	42584
Independent reflections	5658 [R_{int} = 0.0577, R_{sigma} = 0.0309]
Data/restraints/parameters	5658/33/428
Goodness-of-fit on F^2	1.151
Final R indexes [I>=2σ (I)]	R_1 = 0.0514, wR_2 = 0.1252
Final R indexes [all data]	R_1 = 0.0538, wR_2 = 0.1266
Largest diff. peak/hole / e Å^{-3}	0.34/-0.34
Flack parameter	0.2(2)

Supplementary Table 15. Selected bond distances for compound **27** and for the corresponding β-D-galactopyranose[3]

Entry	Bond	Distance (Å)		Δ Distance (Å)
		Compound 27	β-D-galactopyranose[a]	
1	C1-C2	1.528(4)	1.524(5)	0.004
2	C2-C3	1.514(5)	1.524(1)	0.010
3	C3-C4	1.508(5)	1.528(3)	0.020
4	C4-C5	1.536(4)	1.527(2)	0.009
5	C5-C6	1.506(4)	1.512(4)	0.006
6	C5-O5	1.439(4)	1.440(1)	0.001
7	C1-O5	1.423(4)	1.422(3)	0.001
8	C1-O1	1.396(4)	1.396(2)	0.000
9	C6-F6	1.395(4)	1.432(2)	0.037
10	C2-F2	1.388(4)	1.432(3)	0.044
11	C3-F3	1.403(3)	1.427(2)	0.024
12	C4-F4	1.403(4)	1.435(2)	0.032

[a] For β-D-galactopyranose F2 is O2, F3 is O3, F4 is O4, and F6 is O6.

Supplementary Table 16. Key interatomic distances (intramolecular) for compound **27** and β-D-galactopyranose[3]

Entry	Bond	Distance (Å)		Δ Distance (Å)
		Compound 27	β-D-galactopyranose[a]	
1	F2-F3	2.812(3)	2.877(3)	0.065
2	F3-F4	2.753(3)	2.848(1)	0.095
3	F4-F2	4.182(3)	4.286(2)	0.104
4	F4-F6	2.739(3)	4.235(2)	1.496
5	F3-H2	2.548	2.650(3)	0.102
6	F3-H4	2.636	2.656(1)	0.020

[a] For β-D-galactopyranose F2 is O2, F3 is O3, F4 is O4, and F6 is O6.

Supplementary Table 17. Selected H···F bond distances and angles for compound **27**

Entry	D–H···A	*d*(D–H) (Å)	*d*(H···A) (Å)	*d*(D···A) (Å)	*a*(D–H–A) (°)
1	C_6–H···F_6	0.99	2.792	3.413	121.37
2	C_5–H···F_6	1.00	2.626	3.140	111.92
3	C_4–H···F_6	1.00	2.502	3.163	123.22
4	C_4–H···F_4	1.00	2.870	3.424	118.28
5	C_3–H···F_4	1.00	2.567	3.106	113.65

Supplementary Table 18. Selected bond angles for compound **27**, and for the corresponding β-D-galactopyranose[3]

Entry	Bond	Angles (°)		Δ Angles (°)
		Compound 27	β-D-galactopyranose[a]	
1	C1-C2-C3	109.8(2)	109.1(1)	0.7
2	C2-C3-C4	111.5(3)	110.9(3)	0.6
3	C3-C4-C5	107.8(2)	108.3(1)	0.5
4	C4-C5-O5	108.9(2)	108.2(4)	0.7
5	C5-O5-C1	112.4(2)	111.5(1)	0.9
6	O5-C1-C2	110.3(2)	110.6(2)	0.3
7	O1-C1-O5	107.1(2)	106.7(5)	0.4
8	O1-C1-C2	107.1(2)	109.4(1)	2.3
9	F2-C2-C1	108.1(2)	109.1(4)	1.0
10	F2-C2-C3	108.7(2)	107.9(2)	0.8
11	F2-C2-H2	110	110.8(2)	0.8
12	F3-C3-C2	108.6(2)	111.9(5)	3.3
13	F3-C3-C4	110.2(2)	110.8(4)	0.6
14	F3-C3-H3	108.8	107.5(3)	1.3
15	F4-C4-C3	108.5(2)	109.5(2)	1.0
16	F4-C4-C5	110.0(2)	110.6(3)	0.6
17	F4-C4-H4	110.2	109.4(3)	0.8
18	C5-C6-F6	111.4(2)	110.1(4)	1.3

[a] For β-D-galactopyranose F2 is O2, F3 is O3, F4 is O4, and F6 is O6.

Supplementary Table 19. Selected torsion angles for compound **27** and for the corresponding β-D-galactopyranose[3]

Entry	Bond	Torsion angles (°)		Δ Torsion angles (°)
		Compound 27	β-D-galactopyranose[a]	
1	O5-C5-C6-F6	-62.4(3)	57.6(1)	120.0
2	C1-C2-C3-C4	-53.6(3)	-53.0(1)	0.6
3	C2-C3-C4-C5	56.3(3)	55.9(5)	0.4
4	C3-C4-C5-O5	-60.0(3)	-60.4(4)	0.4
5	C4-C5-O5-C1	64.3(3)	65.7(1)	1.4
6	C5-O5-C1-C2	-61.1(3)	-63.6(2)	2.5
7	O5-C1-C2-C3	54.2(3)	55.8(2)	1.6
8	O5-C1-C2-F2	172.6(2)	173.4(2)	0.8
9	C4-C3-C2-F2	-171.7(2)	-171.5(5)	0.2
10	O1-C1-C2-F2	-71.1(3)	-69.4(2)	1.7
11	C1-C2-C3-F3	-175.2(2)	-177.2(3)	2.0
12	C5-C4-C3-F3	177.0(2)	-179.4(3)	2.4
13	C2-C3-C4-F4	-62.8(3)	-64.9(5)	2.1
14	O5-C5-C4-F4	58.1(3)	59.5(4)	1.4
15	C6-C5-C4-F4	-63.7(3)	-58.0(1)	5.7
16	F2-C2-C3-F3	66.6(3)	64.4(1)	2.2
17	F3-C3-C4-F4	57.9(3)	59.9(4)	2.0
18	H1-C1-C2-F2	49.7	52.0(1)	2.3
19	F6-C6-C5-C4	59.7(3)	176.3(1)	116.6

[a] For β-D-galactopyranose F2 is O2, F3 is O3, F4 is O4, and F6 is O6.

Supplementary Table 20. Crystal data and structure refinement for compound **44**

Empirical formula	$C_{20}H_{15}Br_2F_3O_5$
Formula weight	552.14
Temperature/K	150
Crystal system	orthorhombic
Space group	$P2_12_12_1$
a/Å	7.5271(3)
b/Å	10.4562(4)
c/Å	25.1511(8)
α/°	90
β/°	90
γ/°	90
Volume/Å^3	1979.51(13)
Z	4
ρ_{calc}g/cm^3	1.853
μ/mm^{-1}	3.790
F(000)	1088.0
Crystal size/mm^3	0.23 × 0.11 × 0.07
Radiation	GaKα (λ = 1.34139)
2Θ range for data collection/°	6.114 to 121.324
Index ranges	$-9 \leq h \leq 9$, $-13 \leq k \leq 13$, $-32 \leq l \leq 32$
Reflections collected	27401
Independent reflections	4539 [R_{int} = 0.0413, R_{sigma} = 0.0251]
Data/restraints/parameters	4539/0/272
Goodness-of-fit on F^2	1.076
Final R indexes [I>=2σ (I)]	R_1 = 0.0248, wR_2 = 0.0611
Final R indexes [all data]	R_1 = 0.0258, wR_2 = 0.0620
Largest diff. peak/hole / e Å^{-3}	0.41/-0.55
Flack parameter	-0.035(8)

Supplementary Table 21. Selected bond distances for compound **44** and for the corresponding α-D-mannopyranose[4]

Entry	Bond	Distance (Å)		Δ Distance (Å)
		Compound 44	α-D-mannopyranose[a]	
1	C1-C2	1.523(4)	1.518(4)	0.005
2	C2-C3	1.503(4)	1.518(3)	0.015
3	C3-C4	1.501(4)	1.517(1)	0.016
4	C4-C5	1.528(4)	1.528(3)	0.000
5	C5-C6	1.516(4)	1.507(3)	0.011
6	C5-O5	1.431(4)	1.440(1)	0.009
7	C1-O5	1.397(3)	1.412(1)	0.015
8	C1-O1	1.437(3)	1.404(1)	0.033
9	C6-O6	1.456(4)	1.408(4)	0.048
10	C2-F2	1.399(3)	1.421(4)	0.022
11	C3-F3	1.399(4)	1.413(3)	0.014
12	C4-F4	1.394(4)	1.439(3)	0.045

[a] For α-D-mannopyranose F2 is O2, F3 is O3, F4 is O4, and F6 is O6.

Supplementary Table 22. Key interatomic distances (intramolecular) for compound **44** and α-D-mannopyranose[4]

Entry	Bond	Distance (Å)		Δ Distance (Å)
		Compound 44	α-D-mannopyranose[a]	
1	F2-F3	2.710(2)	2.797(2)	0.087
2	F3-F4	2.836(3)	2.933(5)	0.097
3	F4-F2	4.170(3)	4.270(4)	0.100
4	F4-O6	3.648(3)	4.200(3)	0.552
5	F3-H2	2.622	2.570(1)	0.052
6	F3-H4	2.522	2.502(1)	0.020

[a] For α-D-mannopyranose F2 is O2, F3 is O3, F4 is O4, and F6 is O6.

Supplementary Table 23. Selected H···F bond distances and angles for compound **44**

Entry	D–H···A	*d*(D–H) (Å)	*d*(H···A) (Å)	*d*(D···A) (Å)	*a*(D–H–A) (°)
1	C_{AR}–H···F_2	0.95	2.399	3.347	175.02
2	C_{6a}–H···F_2	0.99	2.574	3.123	114.96
3	C_{6b}–H···F_2	0.99	2.837	3.123	97.43
4	C_3–H···F_4	1.00	2.562	3.059	110.53
5	C_2–H···F_4	1.00	2.814	3.402	118.19

Supplementary Table 24. Selected bond angles for compound **44** and for the corresponding α-D-mannopyranose[4]

Entry	Bond	Angles (°)		Δ Angles (°)
		**Compound 44**	**α-D-mannopyranose[a]**	
1	C1-C2-C3	110.9(2)	109.9(2)	1.0
2	C2-C3-C4	111.0(2)	109.6(2)	1.4
3	C3-C4-C5	111.0(2)	111.5(1)	0.5
4	C4-C5-O5	111.4(2)	111.4(2)	0.0
5	C5-O5-C1	115.2(2)	114.8(3)	0.4
6	O5-C1-C2	111.6(2)	110.3(3)	1.3
7	O1-C1-O5	112.7(2)	111.9(1)	0.8
8	O1-C1-C2	104.0(2)	107.2(4)	3.2
9	F2-C2-C1	106.3(2)	106.2(3)	0.1
10	F2-C2-C3	108.2(2)	111.6(2)	3.4
11	F2-C2-H2	110.5	116.8(1)	6.3
12	F3-C3-C2	109.1(2)	106.8(2)	2.3
13	F3-C3-C4	109.4(2)	112.4(3)	3.0
14	F3-C3-H3	109.1	112.3(1)	3.2
15	F4-C4-C3	108.6(2)	108.3(3)	0.3
16	F4-C4-C5	108.1(2)	108.9(1)	0.8
17	F4-C4-H4	109.7	109.8(3)	0.1
18	C5-C6-O6	113.4(2)	110.3(4)	3.1

[a] For α-D-mannopyranose F2 is O2, F3 is O3, F4 is O4, and F6 is O6.

Supplementary Table 25. Selected torsion angles for compound **44** and for the corresponding α-D-mannopyranose[4]

Entry	Bond	Torsion angles (°)		Δ Torsion angles (°)
		**Compound 44**	**α-D-mannopyranose[a]**	
1	O5-C5-C6-O6	-64.7(3)	66.7(2)	131.4
2	C1-C2-C3-C4	-52.7(3)	-56.0(2)	3.3
3	C2-C3-C4-C5	52.0(3)	52.4(4)	0.4
4	C3-C4-C5-O5	-51.9(3)	-50.7(4)	1.2
5	C4-C5-O5-C1	55.2(3)	54.9(3)	0.3
6	C5-O5-C1-C2	-56.1(3)	-59.0(2)	2.9
7	O5-C1-C2-C3	53.9(3)	58.8(3)	4.9
8	O5-C1-C2-F2	-63.5(3)	-62.1(2)	1.4
9	C4-C3-C2-F2	63.5(3)	61.5(1)	2.0
10	O1-C1-C2-F2	174.8(2)	175.9(2)	1.1
11	C1-C2-C3-F3	-173.2(2)	-178.1(4)	4.9
12	C5-C4-C3-F3	172.4(2)	171.0(4)	1.4
13	C2-C3-C4-F4	170.7(2)	172.2(1)	1.5
14	O5-C5-C4-F4	-171.0(2)	-170.2(4)	0.8
15	C6-C5-C4-F4	67.7(3)	72.6(5)	4.9
16	F2-C2-C3-F3	-57.0(3)	-60.6(4)	3.6
17	F3-C3-C4-F4	-68.9(3)	-69.2(1)	0.3
18	H1-C1-C2-F2	57.9	57.2(1)	0.7
19	O6-C6-C5-C4	59.1(3)	-175.6(1)	234.7

[a] For α-D-mannopyranose F2 is O2, F3 is O3, F4 is O4, and F6 is O6.

Supplementary Table 26. Crystal data and structure refinement for **47**

Empirical formula	$C_{20}H_{15}Br_2F_3O_5$
Formula weight	552.14
Temperature/K	150
Crystal system	orthorhombic
Space group	$P2_12_12_1$
a/Å	5.95820(10)
b/Å	11.7697(3)
c/Å	28.7068(7)
α/°	90
β/°	90
γ/°	90
Volume/Å^3	2013.10(8)
Z	4
ρ_{calc}g/cm^3	1.822
μ/mm^{-1}	3.726
F(000)	1088.0
Crystal size/mm^3	0.25 × 0.16 × 0.09
Radiation	GaKα (λ = 1.34139)
2Θ range for data collection/°	5.356 to 121.326
Index ranges	$-7 \leq h \leq 7$, $-15 \leq k \leq 15$, $-37 \leq l \leq 37$
Reflections collected	29233
Independent reflections	4629 [R_{int} = 0.0320, R_{sigma} = 0.0182]
Data/restraints/parameters	4629/0/272
Goodness-of-fit on F^2	1.180
Final R indexes [I>=2σ (I)]	R_1 = 0.0272, wR_2 = 0.0630
Final R indexes [all data]	R_1 = 0.0273, wR_2 = 0.0632
Largest diff. peak/hole / e Å^{-3}	0.47/-0.67
Flack parameter	-0.032(5)

Supplementary Table 27. Selected bond distances for compound **47** and for the corresponding α-D-talopyranose

Entry	Bond	Distance (Å)		Δ Distance (Å)
		Compound 47	α-D-talopyranose[a]	
1	C1-C2	1.518(3)	1.532(4)	0.014
2	C2-C3	1.506(3)	1.523(4)	0.017
3	C3-C4	1.508(4)	1.530(2)	0.022
4	C4-C5	1.524(3)	1.533(4)	0.009
5	C5-C6	1.525(3)	1.513(3)	0.012
6	C5-O5	1.432(3)	1.449(2)	0.017
7	C1-O5	1.399(3)	1.438(2)	0.039
8	C1-O1	1.442(3)	1.403(2)	0.039
9	C6-O6	1.438(4)	1.434(4)	0.004
10	C2-F2	1.393(3)	1.423(2)	0.030
11	C3-F3	1.397(3)	1.421(2)	0.024
12	C4-F4	1.395(3)	1.428(2)	0.032

[a] For α-D-talopyranose F2 is O2, F3 is O3, F4 is O4, and F6 is O6.

Supplementary Table 28. Key interatomic distances (intramolecular) for compound **47** and α-D-talopyranose

Entry	Bond	Distance (Å)		Δ Distance (Å)
		Compound 47	α-D-talopyranose[a]	
1	F2-F3	2.732(3)	2.815(4)	0.083
2	F3-F4	2.714(2)	2.852(3)	0.138
3	F4-F2	2.817(2)	2.655(4)	0.162
4	F4-O6	3.211(2)	4.362(2)	1.151
5	F3-H2	2.328	2.576(2)	0.052
6	F3-H4	2.637	2.678(3)	0.041

[a] For α-D-talopyranose F2 is O2, F3 is O3, F4 is O4, and F6 is O6.

Supplementary Table 29. Selected H···F bond distances and angles for compound **47**

Entry	D–H···A	*d*(D–H) (Å)	*d*(H···A) (Å)	*d*(D···A) (Å)	*a*(D–H–A) (°)
1	C_{AR}–H···F_4	0.93	2.527	3.299	140.50
2	C_{AR}–H···F_3	0.93	2.869	3.631	139.98
3	C_5–H···F_2	0.98	2.747	3.364	121.54
4	C_1–H···F_3	0.98	2.916	3.855	160.76

Supplementary Table 30. Selected bond angles for compound **47**, and for the corresponding α-D-talopyranose

Entry	Bond	Angles (°)		Δ Angles (°)
		Compound 47	α-D-talopyranose[a]	
1	C1-C2-C3	110.2(2)	109.5(1)	0.7
2	C2-C3-C4	113.7(2)	110.4(3)	3.3
3	C3-C4-C5	109.9(2)	107.8(1)	2.1
4	C4-C5-O5	111.8(2)	109.9(2)	1.9
5	C5-O5-C1	114.9(2)	113.7(2)	1.2
6	O5-C1-C2	113.0(2)	110.3(1)	2.7
7	O1-C1-O5	111.1(2)	111.9(3)	0.8
8	O1-C1-C2	105.5(2)	108.0(2)	2.5
9	F2-C2-C1	106.9(2)	109.8(2)	2.9
10	F2-C2-C3	110.5(2)	112.5(1)	2.0
11	F2-C2-H2	109.8	105.9(3)	3.9
12	F3-C3-C2	109.3(2)	107.5(4)	1.8
13	F3-C3-C4	109.7(2)	113.4(4)	3.7
14	F3-C3-H3	108.0	108.9(5)	0.9
15	F4-C4-C3	109.7(2)	108.2(2)	1.5
16	F4-C4-C5	109.2(2)	111.1(4)	1.9
17	F4-C4-H4	109.4	109.3(3)	0.1
18	C5-C6-O6	109.8(8)	112.9(1)	3.1

[a] For α-D-talopyranose F2 is O2, F3 is O3, F4 is O4, and F6 is O6.

Supplementary Table 31. Selected torsion angles for compound **47** and for the corresponding α-D-talopyranose

Entry	Bond	Torsion angles (°)		Δ Torsion angles (°)
		Compound 47	α-D-talopyranose[a]	
1	O5-C5-C6-O6	-178.3(2)	70.4(5)	111.3
2	C1-C2-C3-C4	-49.4(3)	-56.6(2)	7.2
3	C2-C3-C4-C5	50.1(3)	57.9(2)	7.8
4	C3-C4-C5-O5	-51.6(3)	-58.5(1)	6.9
5	C4-C5-O5-C1	56.4(2)	60.9(2)	4.5
6	C5-O5-C1-C2	-56.1(2)	-58.7(3)	2.6
7	O5-C1-C2-C3	51.0(3)	55.3(1)	4.3
8	O5-C1-C2-F2	-69.0(2)	-68.7(4)	0.3
9	C4-C3-C2-F2	68.5(3)	65.8(2)	2.7
10	O1-C1-C2-F2	169.4(2)	168.8(2)	0.6
11	C1-C2-C3-F3	-172.3(2)	179.3(4)	8.4
12	C5-C4-C3-F3	172.8(2)	178.6(2)	5.8
13	C2-C3-C4-F4	-70.0(3)	-62.4(1)	7.6
14	O5-C5-C4-F4	68.8(2)	59.9(1)	8.9
15	C6-C5-C4-F4	-47.0(3)	-60.0(5)	13.0
16	F2-C2-C3-F3	-54.5(2)	-58.4(3)	3.9
17	F3-C3-C4-F4	52.7(3)	58.3(4)	5.6
18	H1-C1-C2-F2	52.4	44.2(1)	8.2
19	O6-C6-C5-C4	-57.5(3)	-168.2(4)	110.7

[a] For α-D-talopyranose F2 is O2, F3 is O3, F4 is O4, and F6 is O6.

Supplementary Table 32. Crystal data and structure refinement for **11**

Empirical formula	$C_{10}H_{13}F_3O_5$
Formula weight	270.20
Temperature/K	150
Crystal system	monoclinic
Space group	$P2_1$
a/Å	5.6790(5)
b/Å	7.9737(7)
c/Å	13.1622(11)
α/°	90
β/°	97.917(4)
γ/°	90
Volume/Å^3	590.34(9)
Z	2
ρ_{calc}g/cm^3	1.520
μ/mm^{-1}	0.847
F(000)	280.0
Crystal size/mm^3	0.12 × 0.11 × 0.08
Radiation	GaKα (λ = 1.34139)
2Θ range for data collection/°	5.898 to 121.378
Index ranges	$-7 \leq h \leq 7$, $-10 \leq k \leq 10$, $-17 \leq l \leq 16$
Reflections collected	10994
Independent reflections	2683 [R_{int} = 0.0563, R_{sigma} = 0.0435]
Data/restraints/parameters	2683/1/166
Goodness-of-fit on F^2	1.063
Final R indexes [I>=2σ (I)]	R_1 = 0.0506, wR_2 = 0.1350
Final R indexes [all data]	R_1 = 0.0537, wR_2 = 0.1395
Largest diff. peak/hole / e Å^{-3}	0.27/-0.27
Flack parameter	0.2(3)

Supplementary Table 33. Selected bond distances for compound **11** and for the corresponding β-D-allopyranose[5]

Entry	Bond	Distance (Å)		Δ Distance (Å)
		Compound 11	β-D-allopyranose[a]	
1	C1-C2	1.522(4)	1.528(4)	0.006
2	C2-C3	1.514(4)	1.522(4)	0.008
3	C3-C4	1.512(4)	1.508(4)	0.004
4	C4-C5	1.530(4)	1.521(5)	0.009
5	C5-C6	1.510(4)	1.503(5)	0.007
6	C5-O5	1.432(4)	1.447(3)	0.015
7	C1-O5	1.415(3)	1.432(4)	0.017
8	C1-O1	1.413(4)	1.386(4)	0.027
9	C6-O6	1.441(4)	1.424(5)	0.017
10	C2-F2	1.399(3)	1.422(4)	0.023
11	C3-F3	1.397(3)	1.428(4)	0.031
12	C4-F4	1.400(3)	1.425(4)	0.025

[a] For β-D-allopyranose F2 is O2, F3 is O3, F4 is O4, and F6 is O6.

Supplementary Table 34. Key interatomic distances (intramolecular) for compound **11** and β-D-allopyranose[5]

Entry	Bond	Distance (Å)		Δ Distance (Å)
		Compound 11	β-D-allopyranose[a]	
1	F2-F3	2.725(3)	2.831(4)	0.106
2	F3-F4	2.669(3)	2.836(3)	0.167
3	F4-F2	4.723(3)	4.821(4)	0.098
4	F4-O6	3.423(3)	3.245(3)	0.178
5	F3-H2	3.223	3.230(4)	0.007
6	F3-H4	3.225	3.180(3)	0.045

[a] For β-D-allopyranose F2 is O2, F3 is O3, F4 is O4, and F6 is O6.

Supplementary Table 35. Selected H···F bond distances and angles for compound **11**

Entry	D–H···A	*d*(D–H) (Å)	*d*(H···A) (Å)	*d*(D···A) (Å)	*a*(D–H–A) (°)
1	C_2–H···F_4	1.000	2.397	3.386	169.97
2	C_3–H···F_2	1.001	2.555	3.299	131.05
3	C_3–H···F_3	1.001	2.720	3.440	129.09

Supplementary Table 36. Selected bond angles for compound **11**, and for the corresponding β-D-allopyranose[5]

Entry	Bond	Angles (°)		Δ Angles (°)
		Compound 11	β-D-allopyranose[a]	
1	C1-C2-C3	109.9(2)	108.2(2)	1.7
2	C2-C3-C4	109.5(2)	108.4(2)	1.1
3	C3-C4-C5	110.1(2)	111.2(2)	1.1
4	C4-C5-O5	107.5(2)	107.8(2)	0.3
5	C5-O5-C1	111.9(2)	112.3(2)	0.4
6	O5-C1-C2	107.9(2)	108.3(2)	0.4
7	O1-C1-O5	106.0(2)	107.0(2)	1.0
8	O1-C1-C2	108.4(2)	114.1(2)	5.7
9	F2-C2-C1	109.6(2)	112.3(2)	2.7
10	F2-C2-C3	109.2(2)	109.3(2)	0.1
11	F2-C2-H2	109.3	110.0(2)	0.7
12	F3-C3-C2	108.6(2)	109.4(2)	0.8
13	F3-C3-C4	108.4(2)	108.6(2)	0.2
14	F3-C3-H3	110.1	111.0(2)	0.9
15	F4-C4-C3	108.2(2)	111.4(2)	3.2
16	F4-C4-C5	107.8(2)	110.1(2)	2.3
17	F4-C4-H4	110.2	106.0(2)	4.2
18	C5-C6-O6	107.8(2)	112.2(3)	4.4

[a] For β-D-allopyranose F2 is O2, F3 is O3, F4 is O4, and F6 is O6.

Supplementary Table 37. Selected torsion angles for compound **11** and for the corresponding β-D-allopyranose[5]

Entry	Bond	Torsion angles (°)		Δ Torsion angles (°)
		Compound 11	β-D-allopyranose[a]	
1	O5-C5-C6-O6	-64.3(3)	-75.3(3)	11.0
2	C1-C2-C3-C4	-54.3(3)	-58.0(3)	3.7
3	C2-C3-C4-C5	54.1(3)	57.0(3)	2.9
4	C3-C4-C5-O5	-58.3(3)	-57.3(3)	1.0
5	C4-C5-O5-C1	66.0(3)	62.0(3)	4.0
6	C5-O5-C1-C2	-66.6(3)	-65.1(3)	1.5
7	O5-C1-C2-C3	59.3(3)	61.6(3)	2.3
8	O5-C1-C2-F2	179.4(2)	-177.7(2)	2.9
9	C4-C3-C2-F2	-174.6(2)	179.5(2)	5.9
10	O1-C1-C2-F2	-66.2(3)	-58.7(3)	7.5
11	C1-C2-C3-F3	63.9(3)	60.2(3)	3.7
12	C5-C4-C3-F3	-64.3(3)	-61.7(3)	2.6
13	C2-C3-C4-F4	171.6(2)	-179.7(2)	8.7
14	O5-C5-C4-F4	-176.1(2)	178.7(2)	5.2
15	C6-C5-C4-F4	64.1(3)	58.4(3)	5.7
16	F2-C2-C3-F3	-56.4(3)	-62.3(3)	5.9
17	F3-C3-C4-F4	53.3(3)	61.5(3)	8.2
18	H1-C1-C2-F2	56.7	59.0(2)	2.3
19	O6-C6-C5-C4	54.9(3)	44.6(4)	10.3

[a] For β-D-allopyranose F2 is O2, F3 is O3, F4 is O4, and F6 is O6.

Supplementary Figures

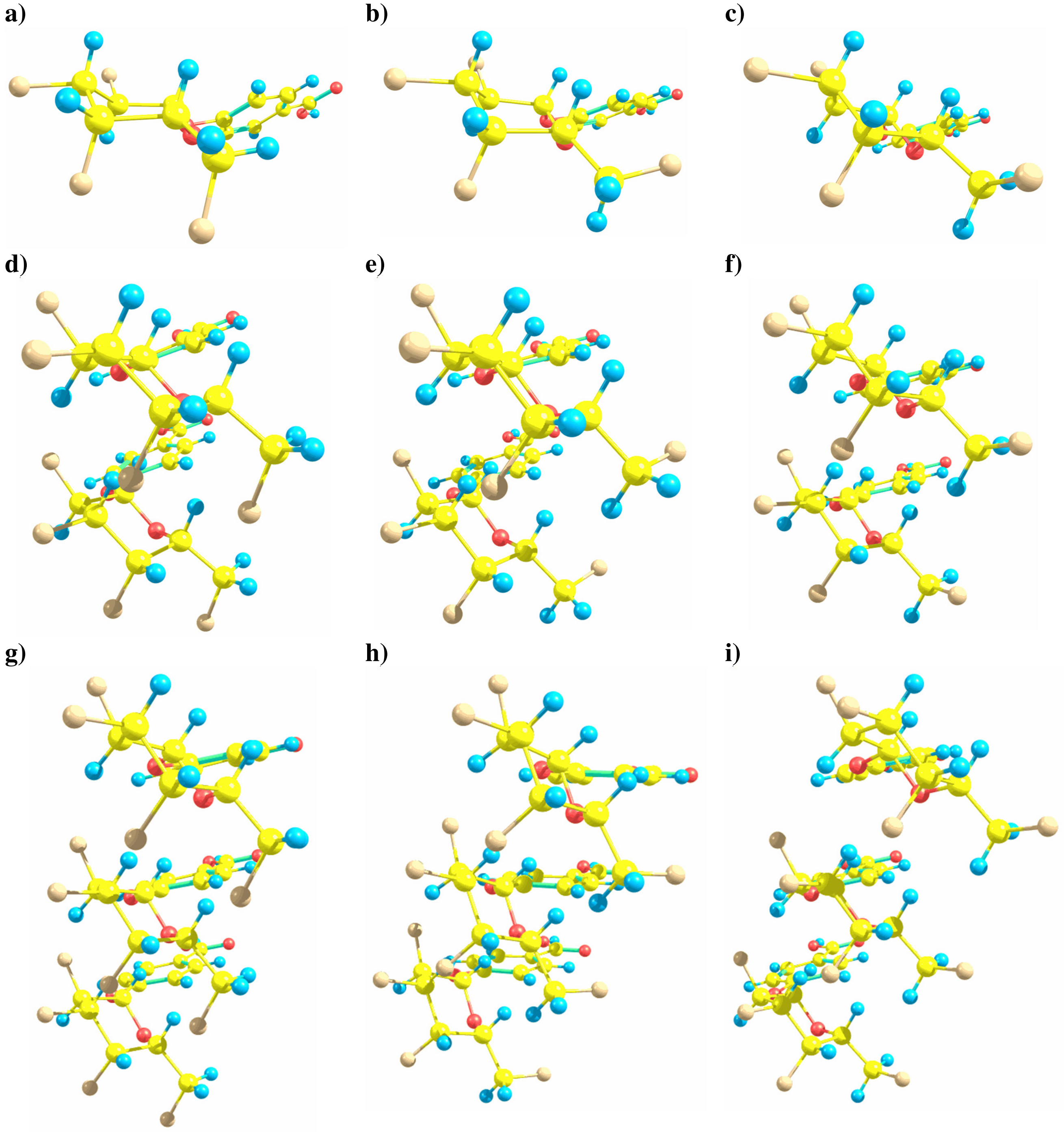

Supplementary Figure 1. Structure of the a) GG conformer of molecule **27**; b) GT conformer of molecule **27**; c) TG conformer of molecule **27**; d) Dimer of the GG conformer of molecule **27**; e) Dimer of the GT conformer of molecule **27**; f) Dimer of the TG conformer of molecule **27**; g) Trimer of the GG conformer of molecule **27**; h) Trimer of the GT conformer of molecule **27**; and i) Trimer of the TG conformer of molecule **27**. Carbon (yellow), hydrogen (blue), oxygen (red), fluorine (tan).

a) b)

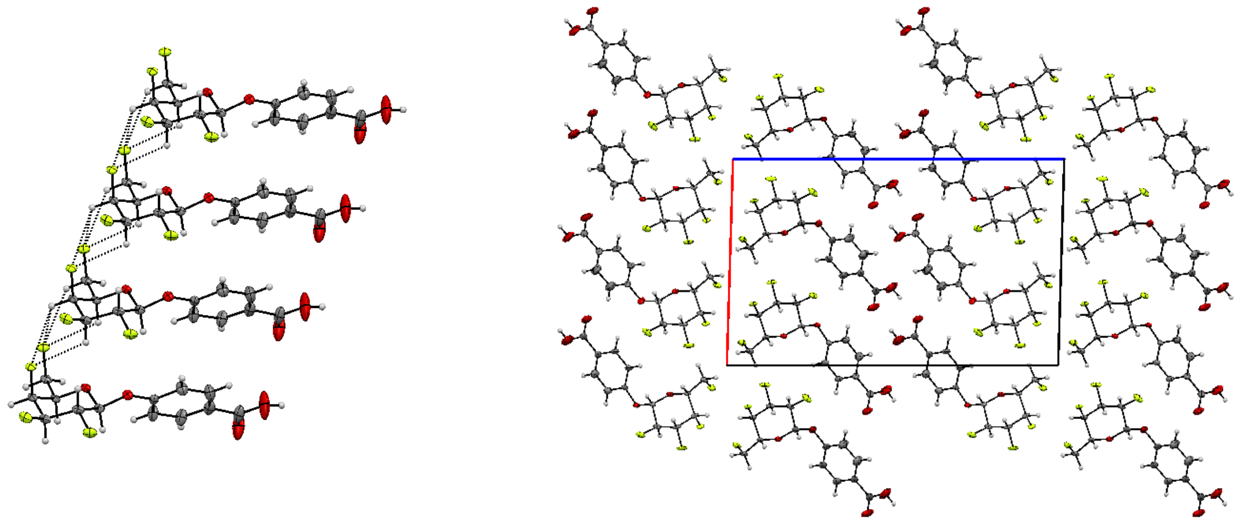

Supplementary Figure 2. Crystal packing of galactose analog **27**. a) showing part of the hydrogen bonded sheet and b) viewed down the *b* axis, showing segregation between polarized pyrans and aromatic groups along the *c* axis.

a) b)

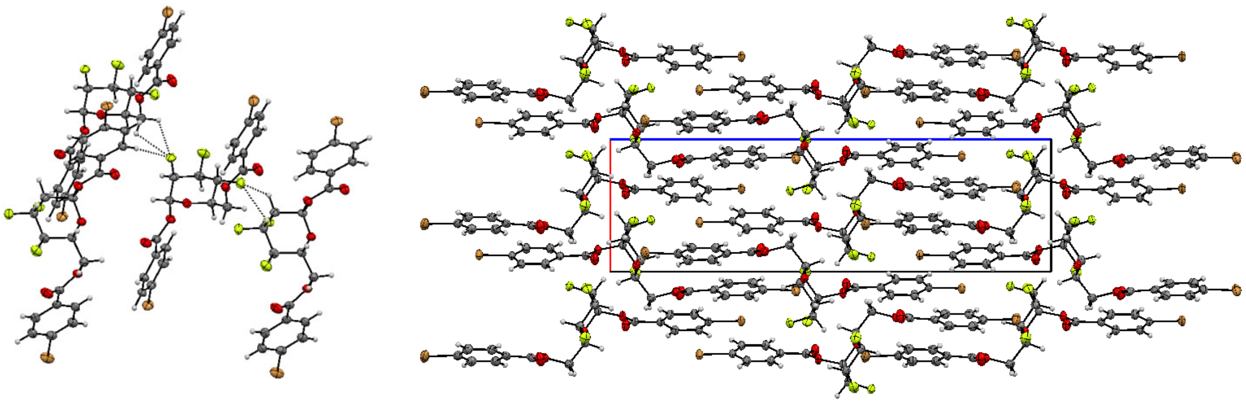

Supplementary Figure 3. Crystal packing of mannose analog **44**. a) showing part of the hydrogen bonded sheet and b) viewed down the *b* axis, showing segregation between polarized pyrans and aromatic groups along the *c* axis.

a) b)

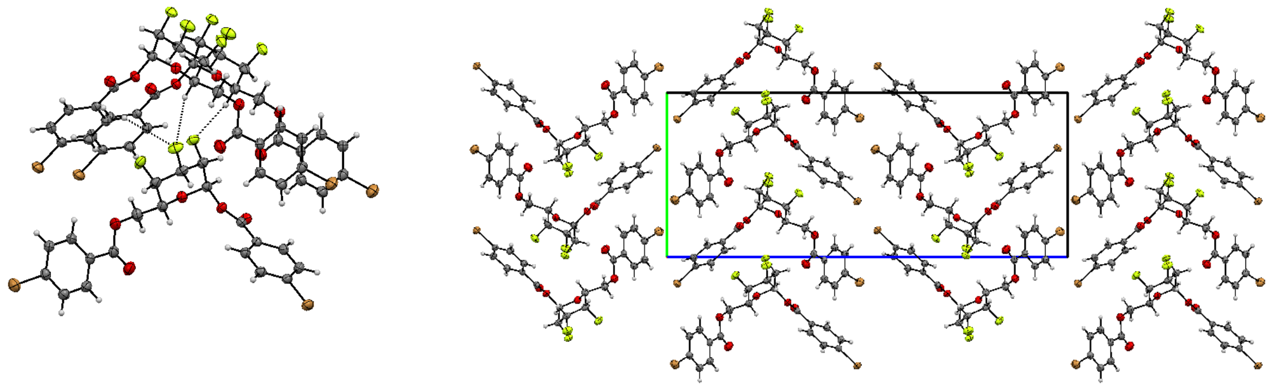

Supplementary Figure 4. Crystal packing of talose analog **47**. a) showing part of the hydrogen bonded sheet and b) viewed down the *a* axis, showing segregation between polarized pyrans and aromatic groups along the *c* axis. Dot line represent possible intermolecular C–F···H–C interactions.

a) b)

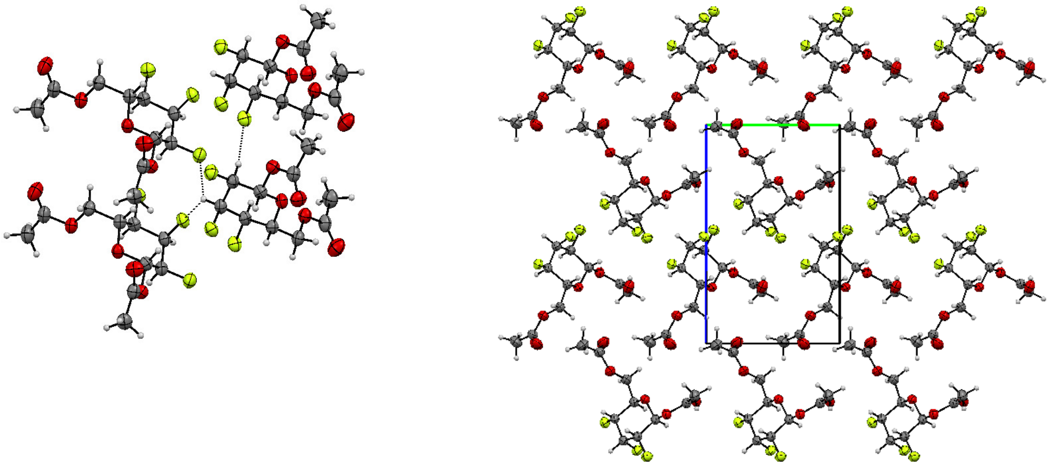

Supplementary Figure 5. Crystal packing of allose analog **11**. a) showing part of the hydrogen bonded sheet and b) viewed down the *a* axis, showing segregation between polarized pyrans and aromatic groups along the *b* axis. Dot line represent possible intermolecular C–F···H–C interactions.

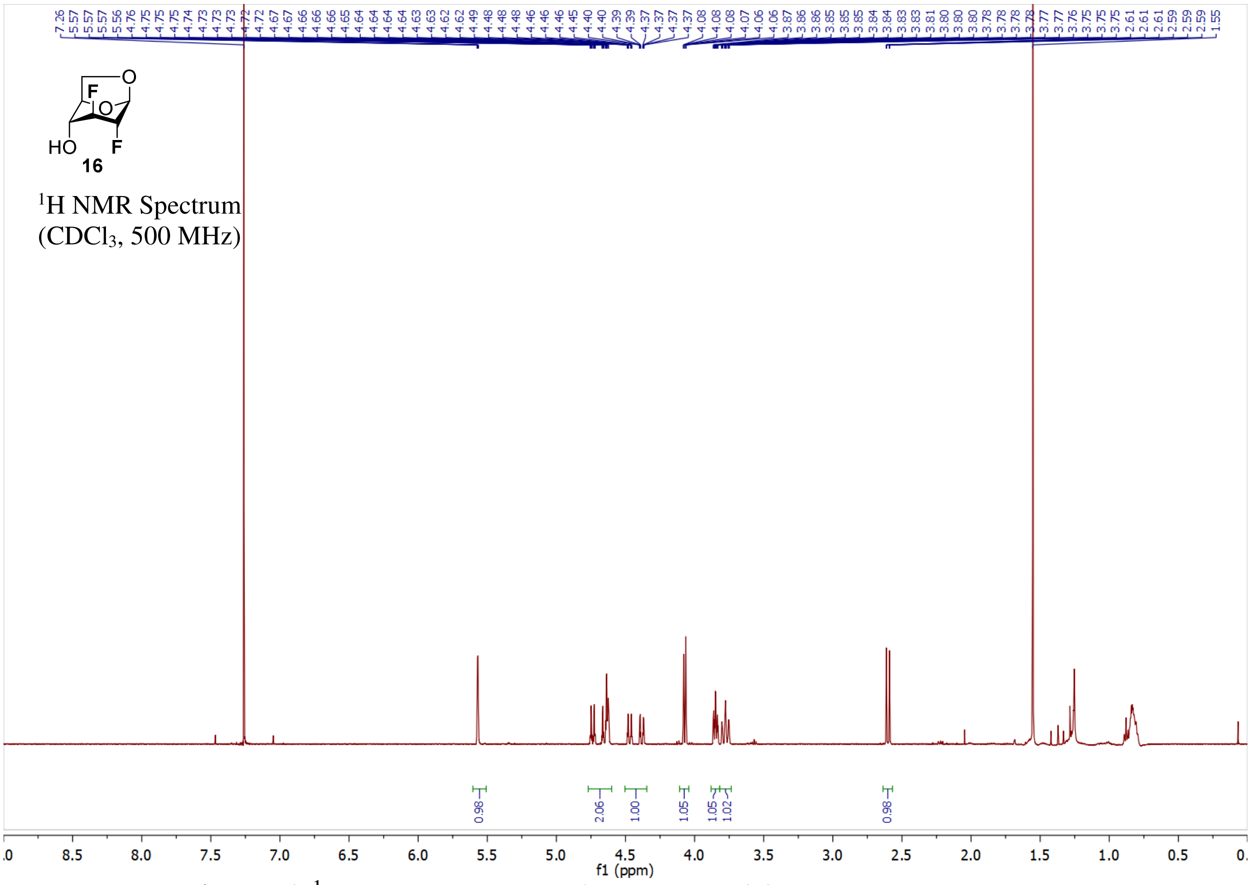

Supplementary Figure 6. ^{1}H NMR spectrum of compound **16**

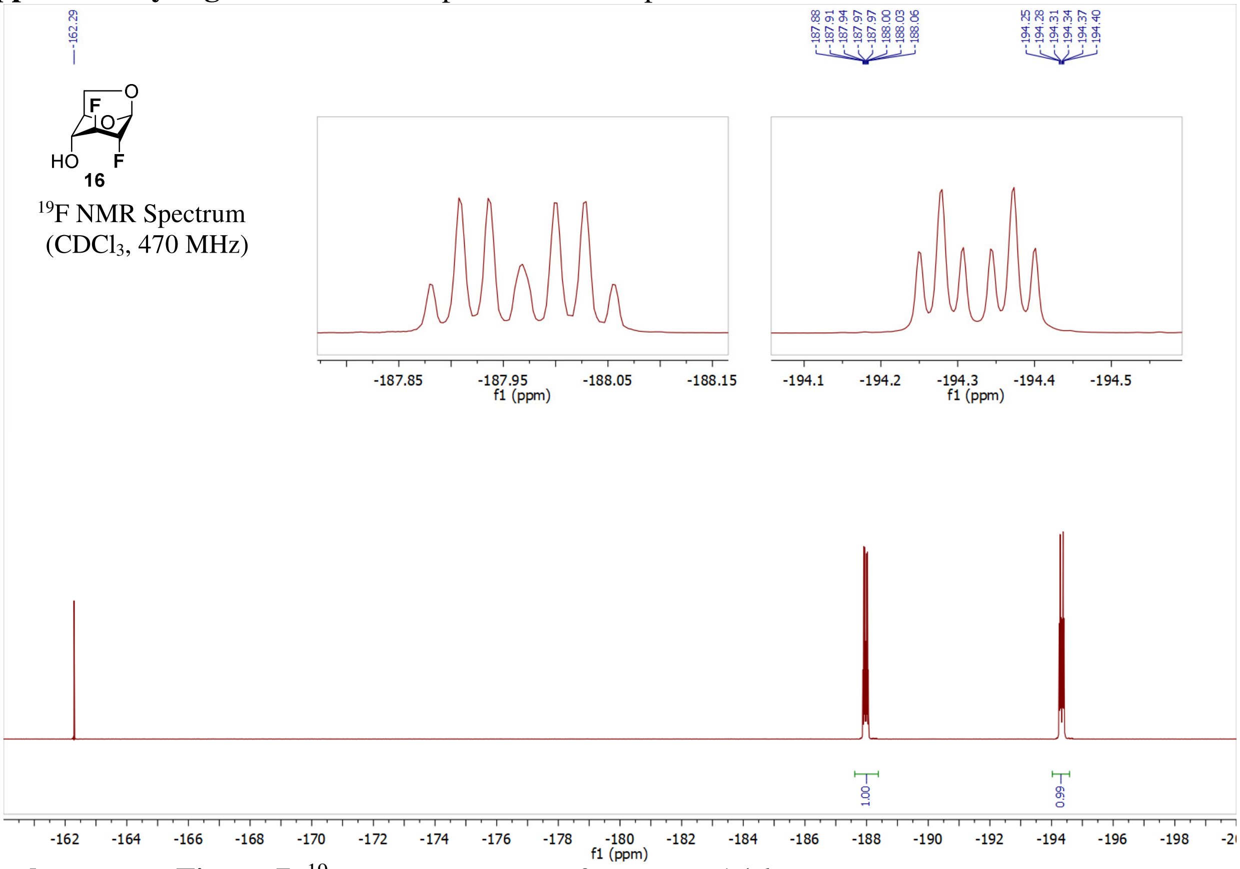

Supplementary Figure 7. ^{19}F NMR spectrum of compound **16**

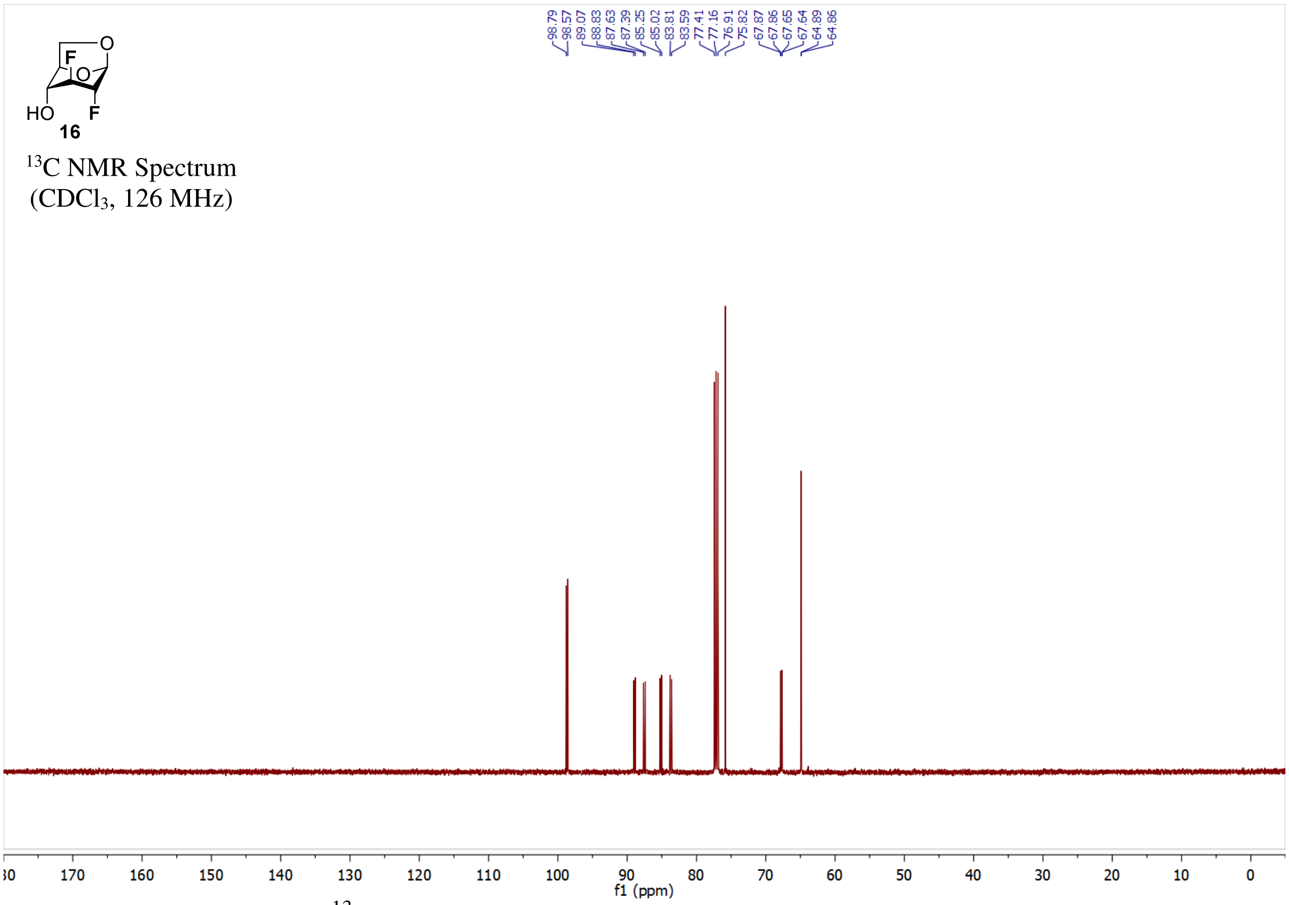

Supplementary Figure 8. ^{13}C NMR spectrum of compound **16**

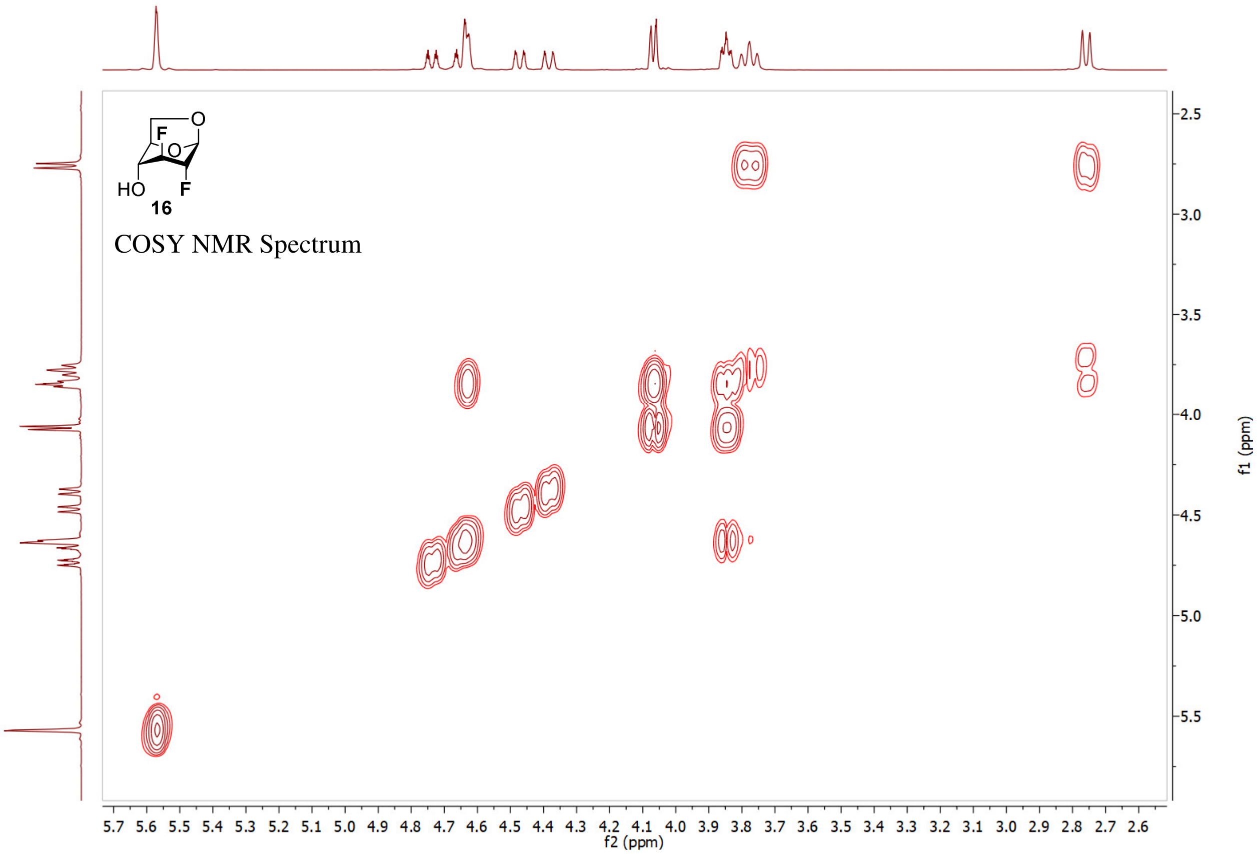

Supplementary Figure 9. COSY NMR spectrum of compound **16**

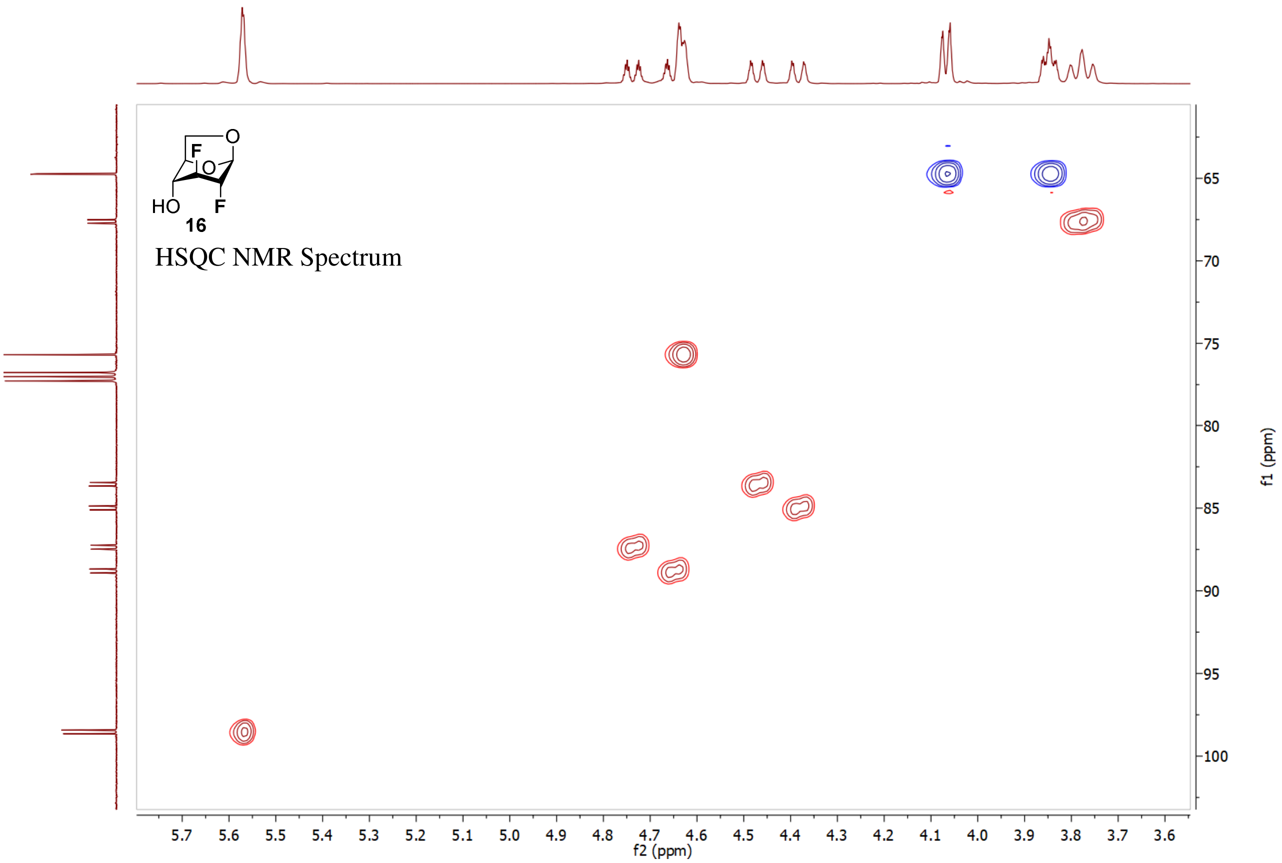

Supplementary Figure 10. HSQC NMR spectrum of compound **16**

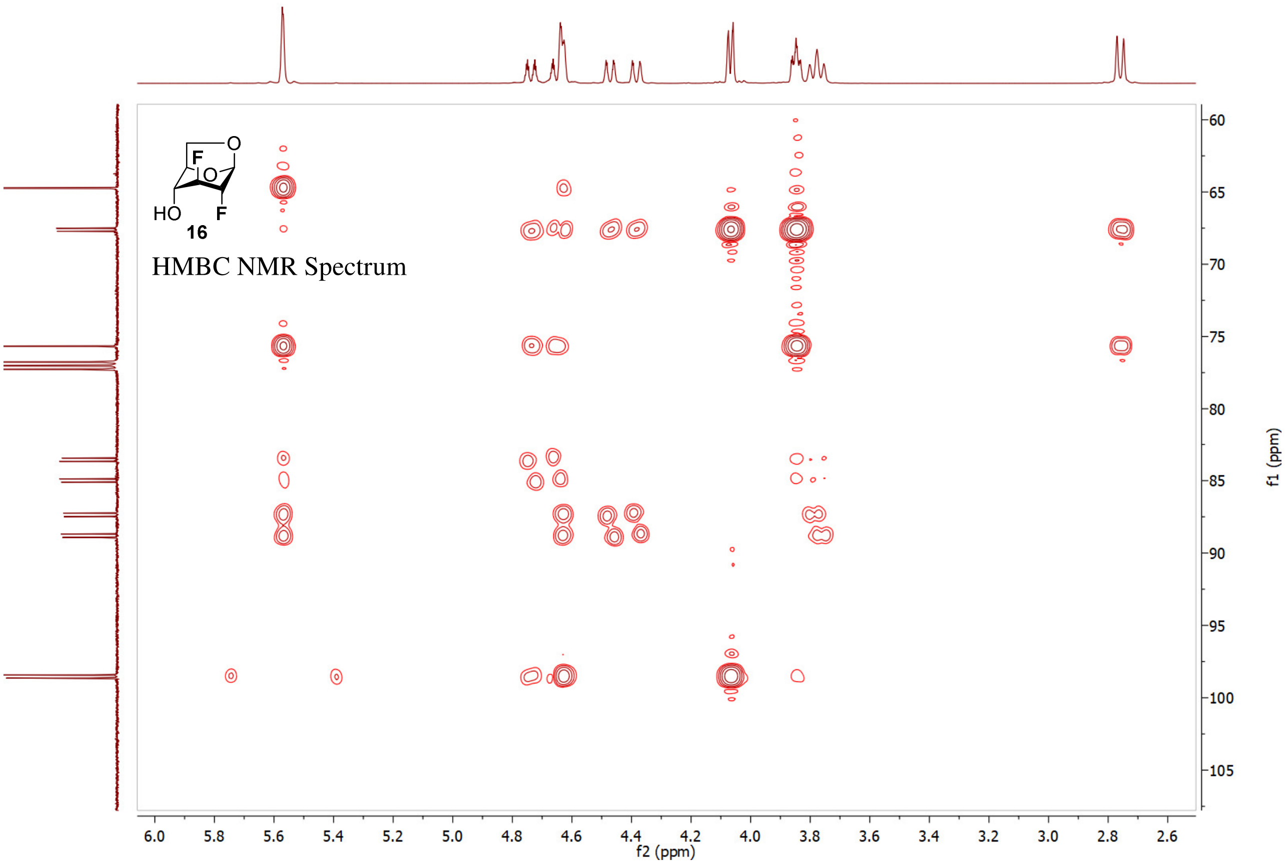

Supplementary Figure 11. HMBC NMR spectrum of compound **16**

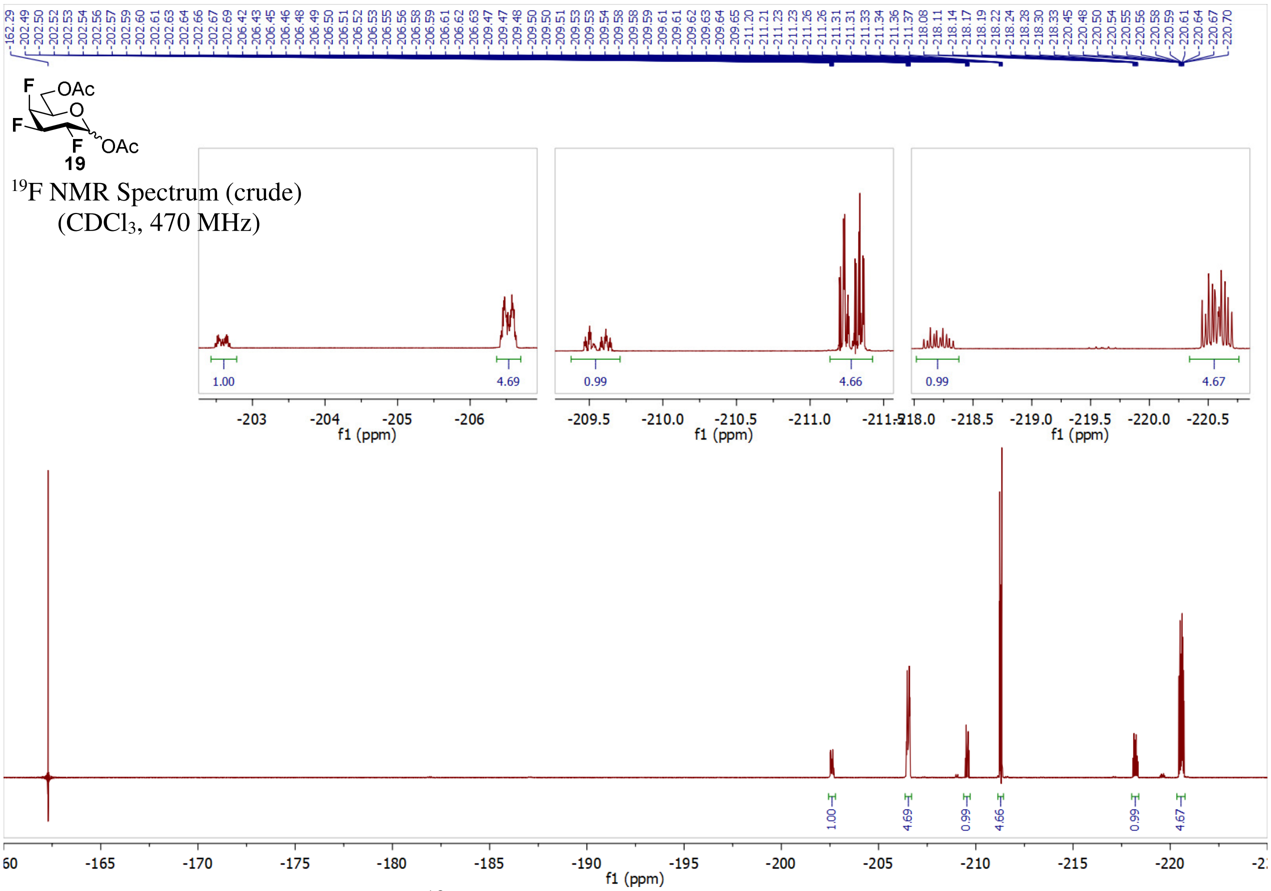

Supplementary Figure 12. Crude ^{19}F NMR spectrum of compound **19**

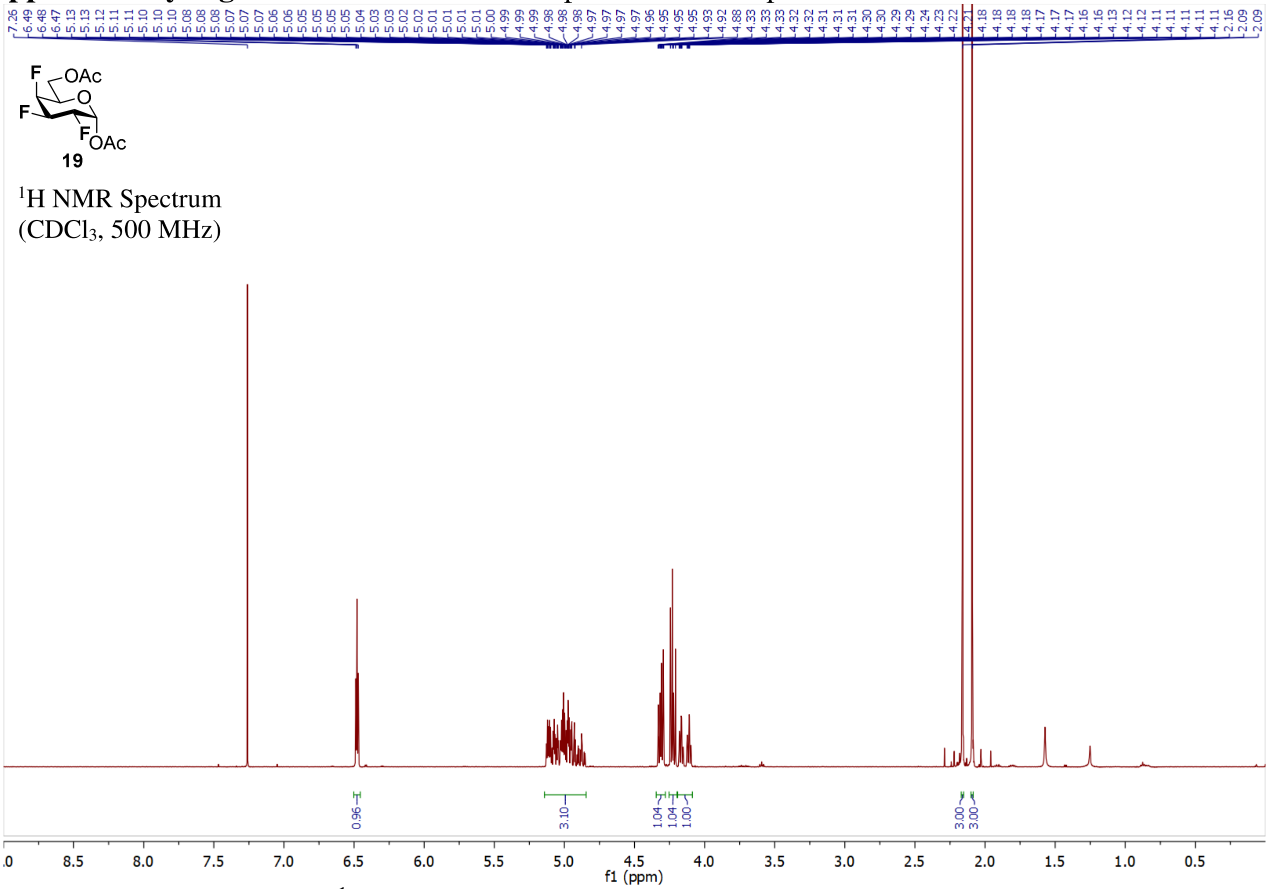

Supplementary Figure 13. ^{1}H NMR spectrum of compound **19**

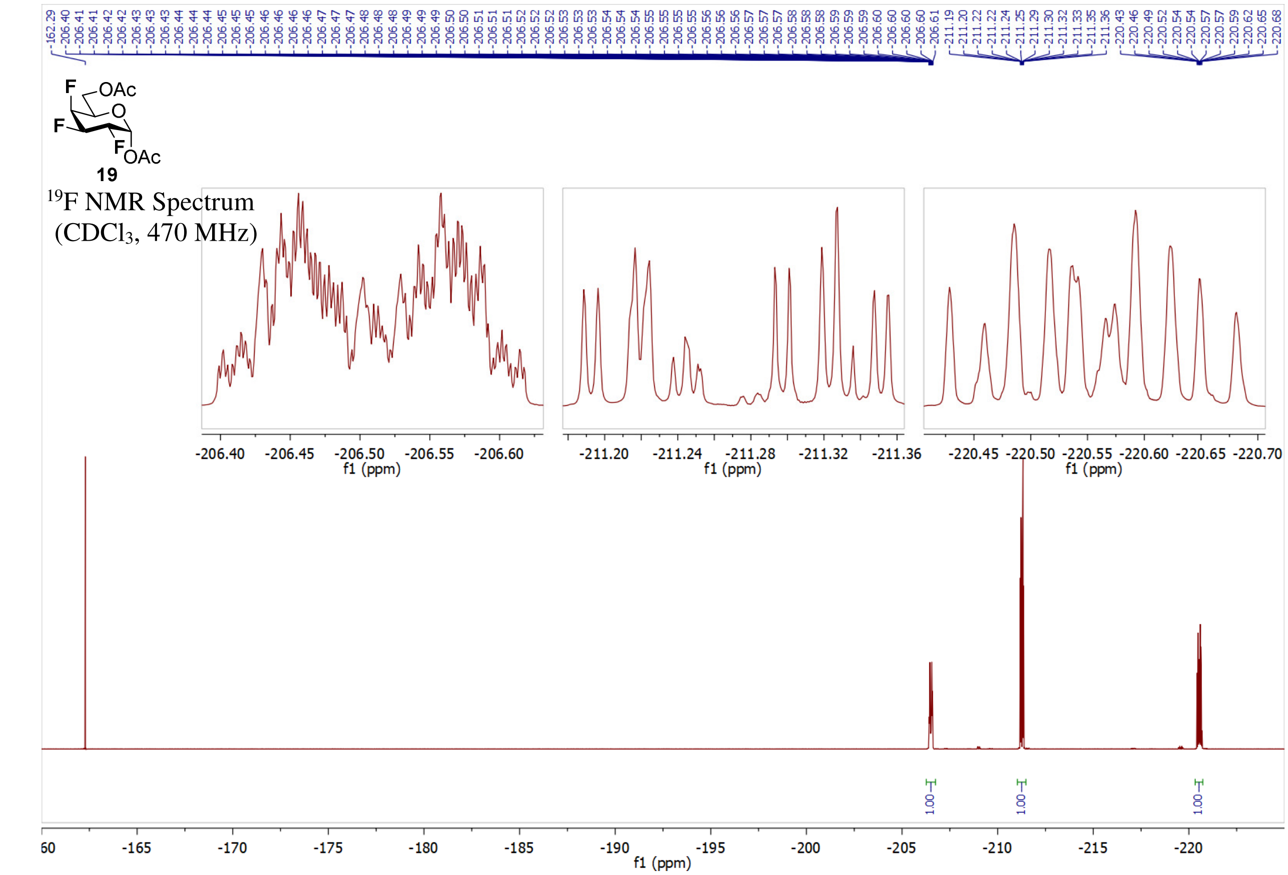

Supplementary Figure 14. ^{19}F NMR spectrum of compound **19**

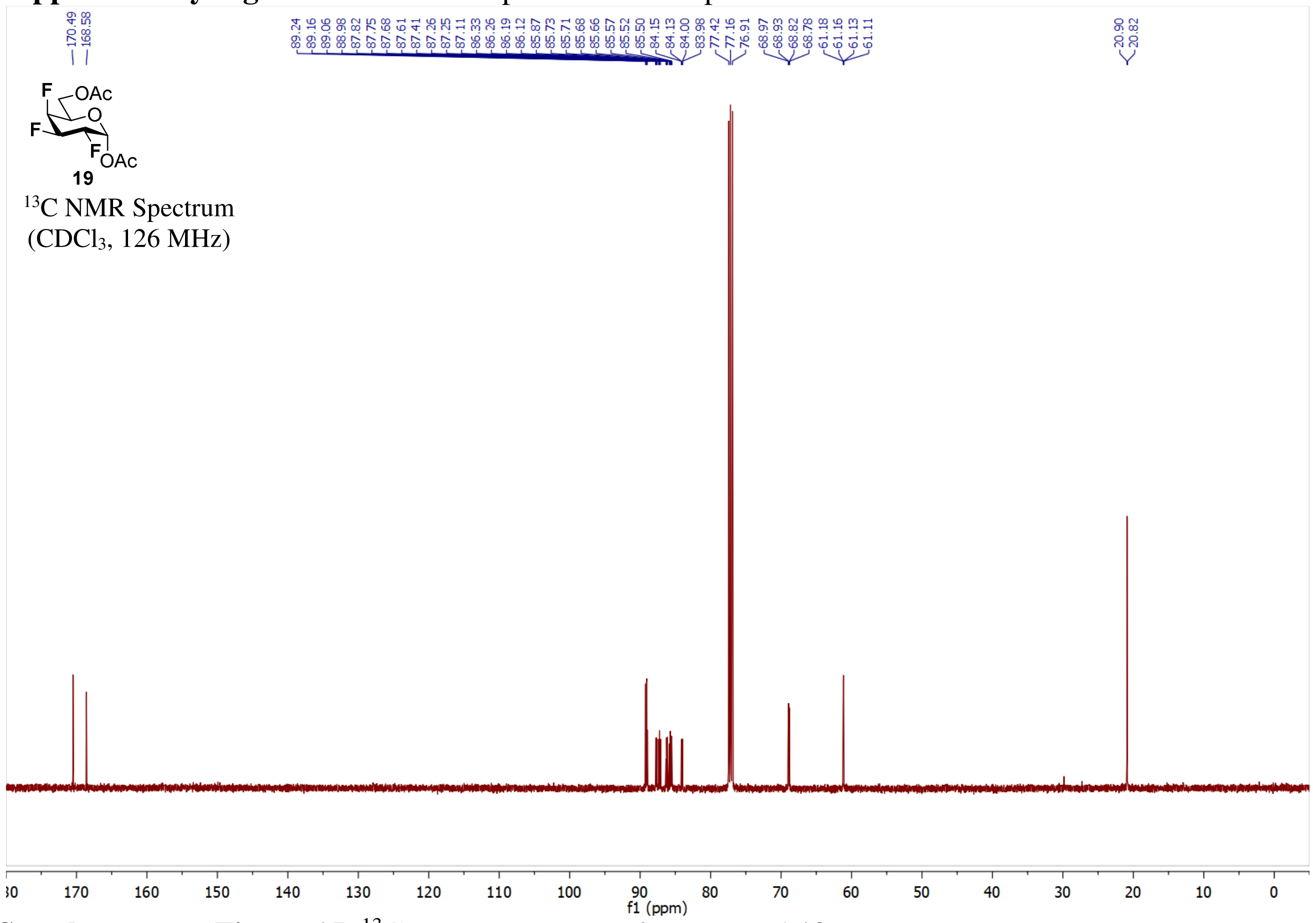

Supplementary Figure 15. ^{13}C NMR spectrum of compound **19**

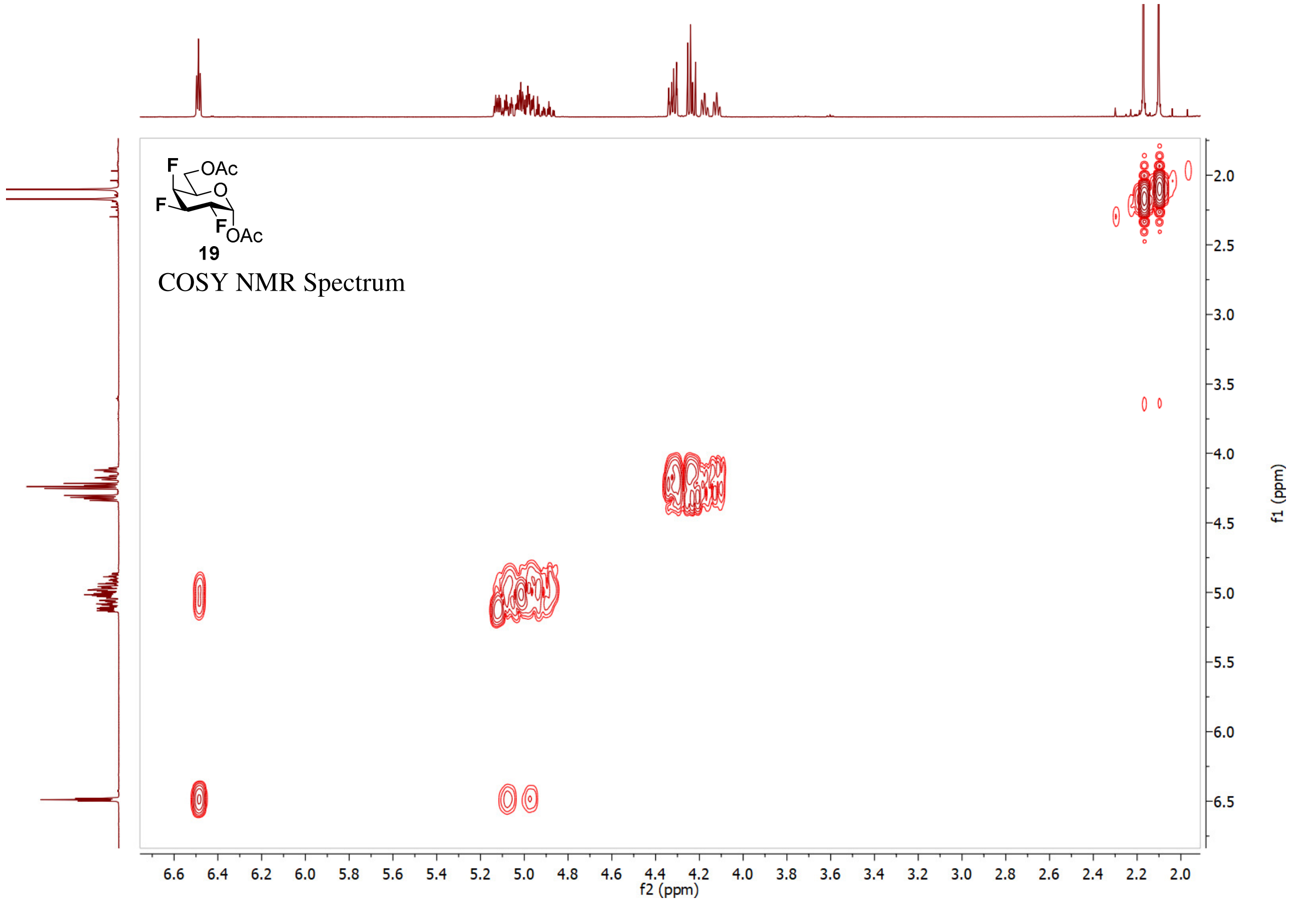

Supplementary Figure 16. COSY NMR spectrum of compound **19**

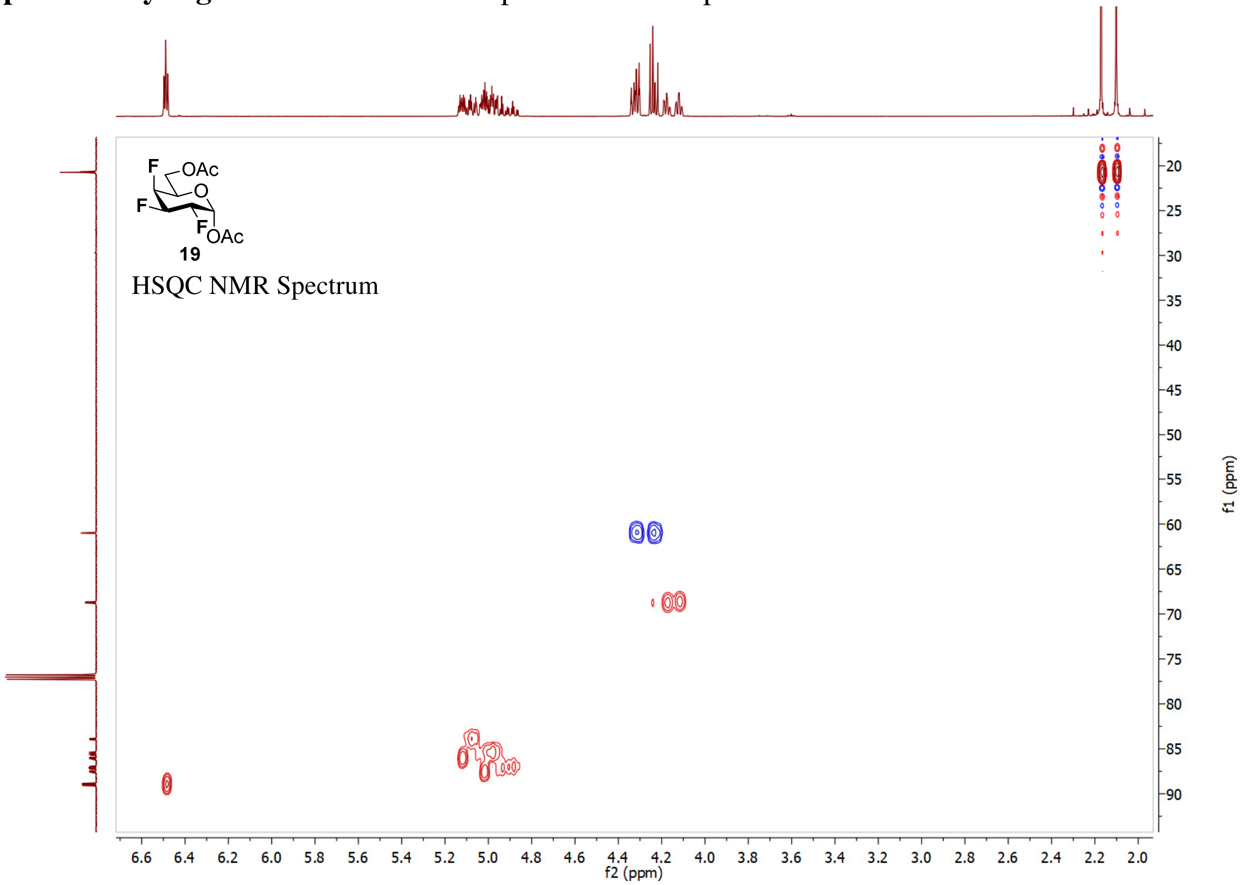

Supplementary Figure 17. HSQC NMR spectrum of compound **19**

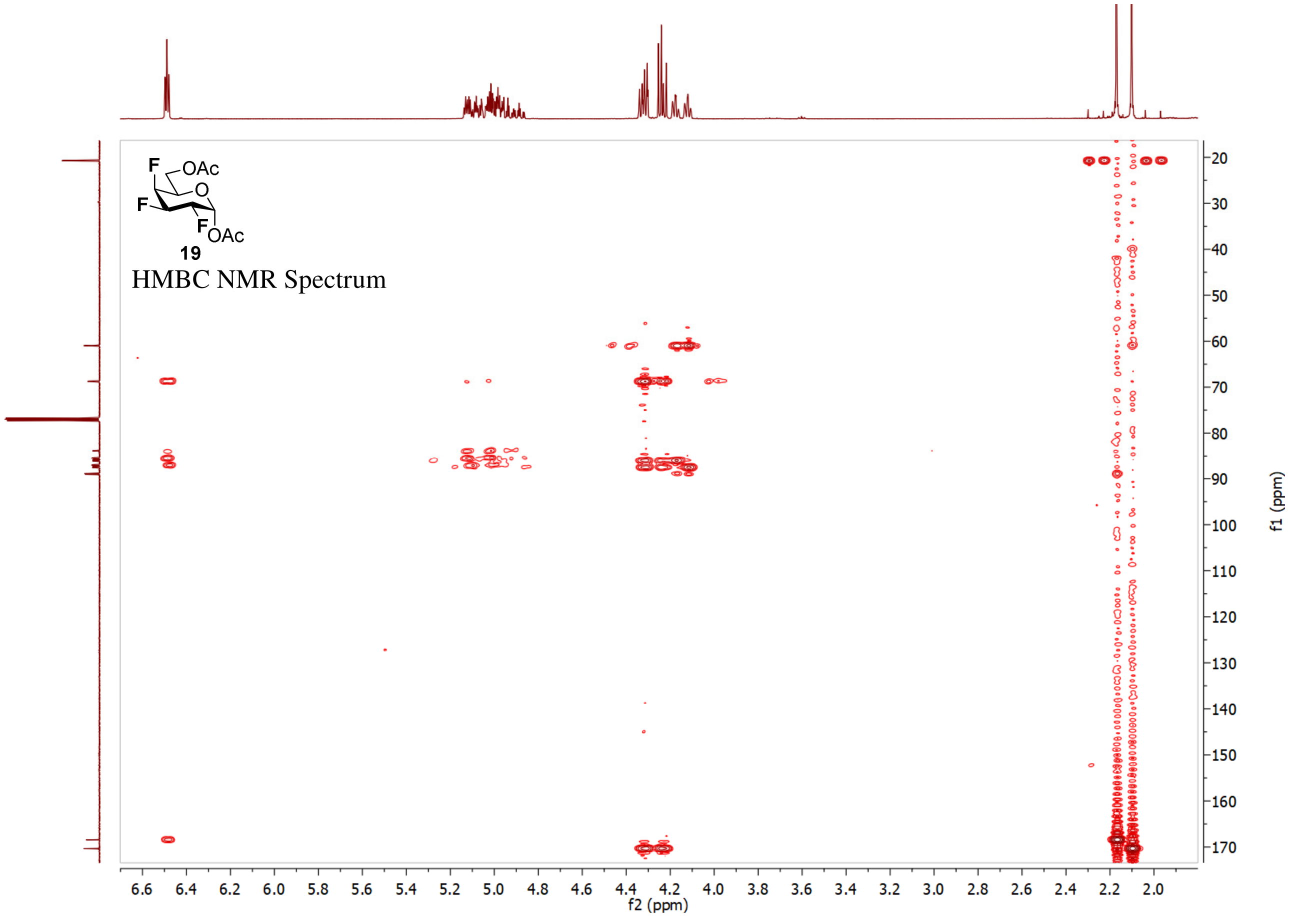

Supplementary Figure 18. HMBC NMR spectrum of compound **19**

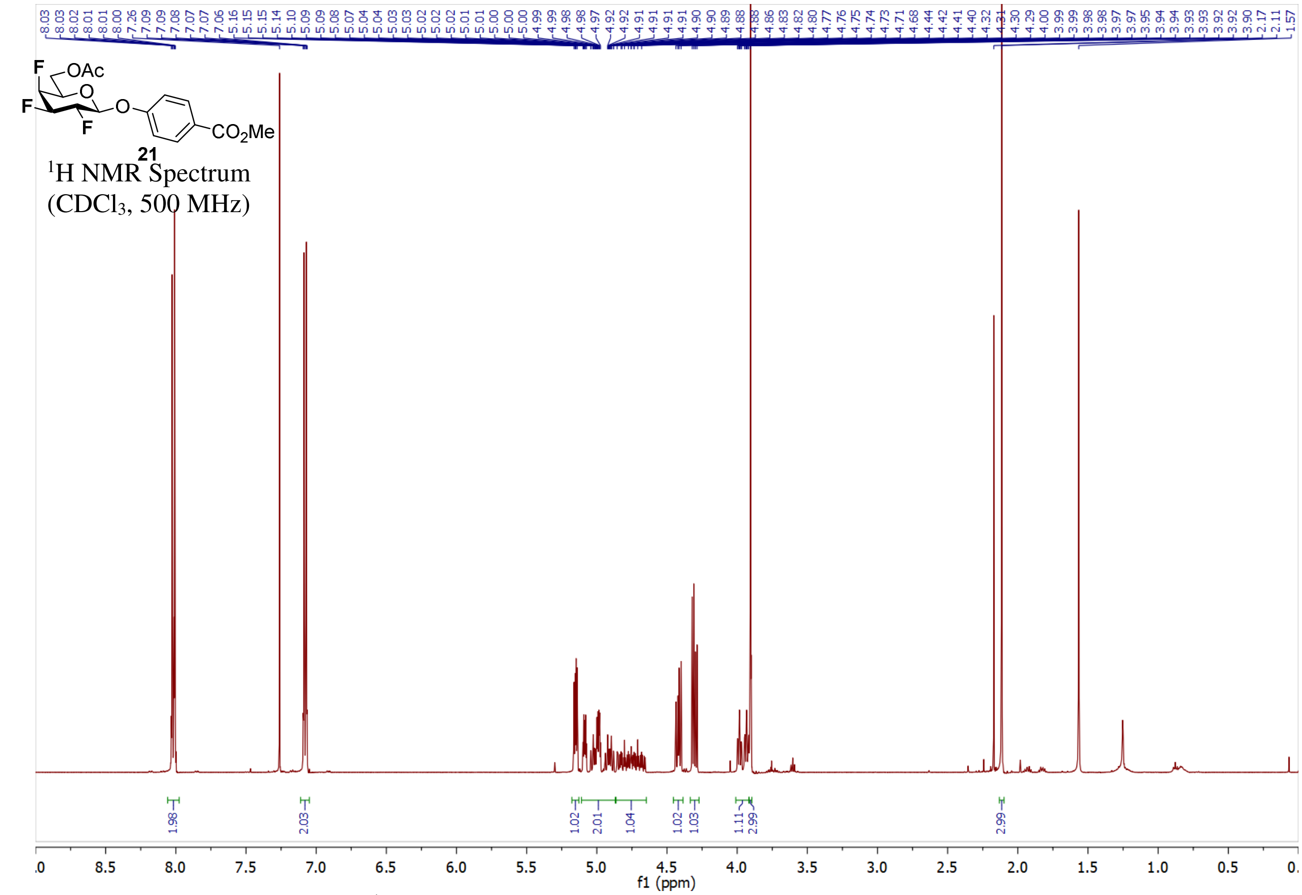

Supplementary Figure 19. ^{1}H NMR spectrum of compound **21**

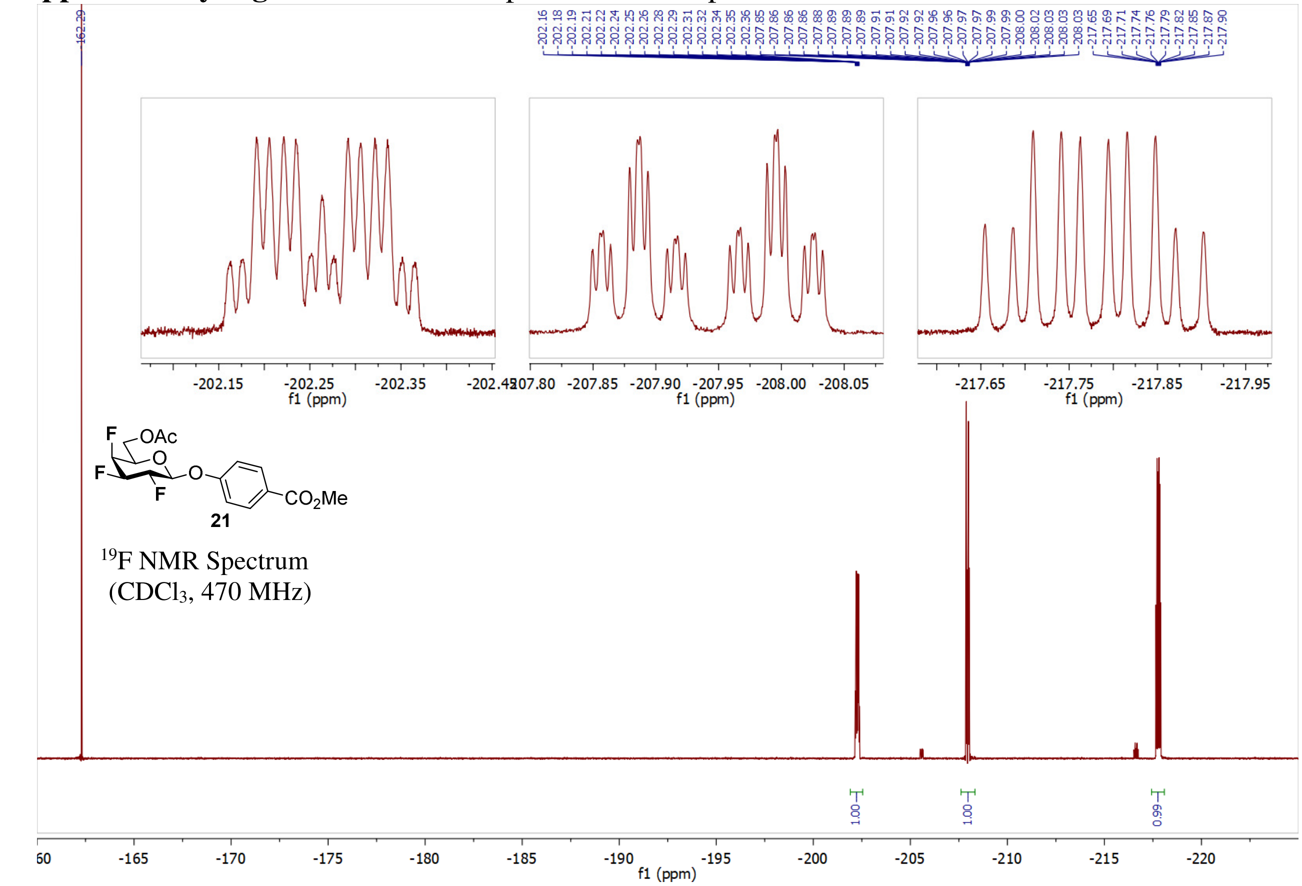

Supplementary Figure 20. ^{19}F NMR spectrum of compound **21**

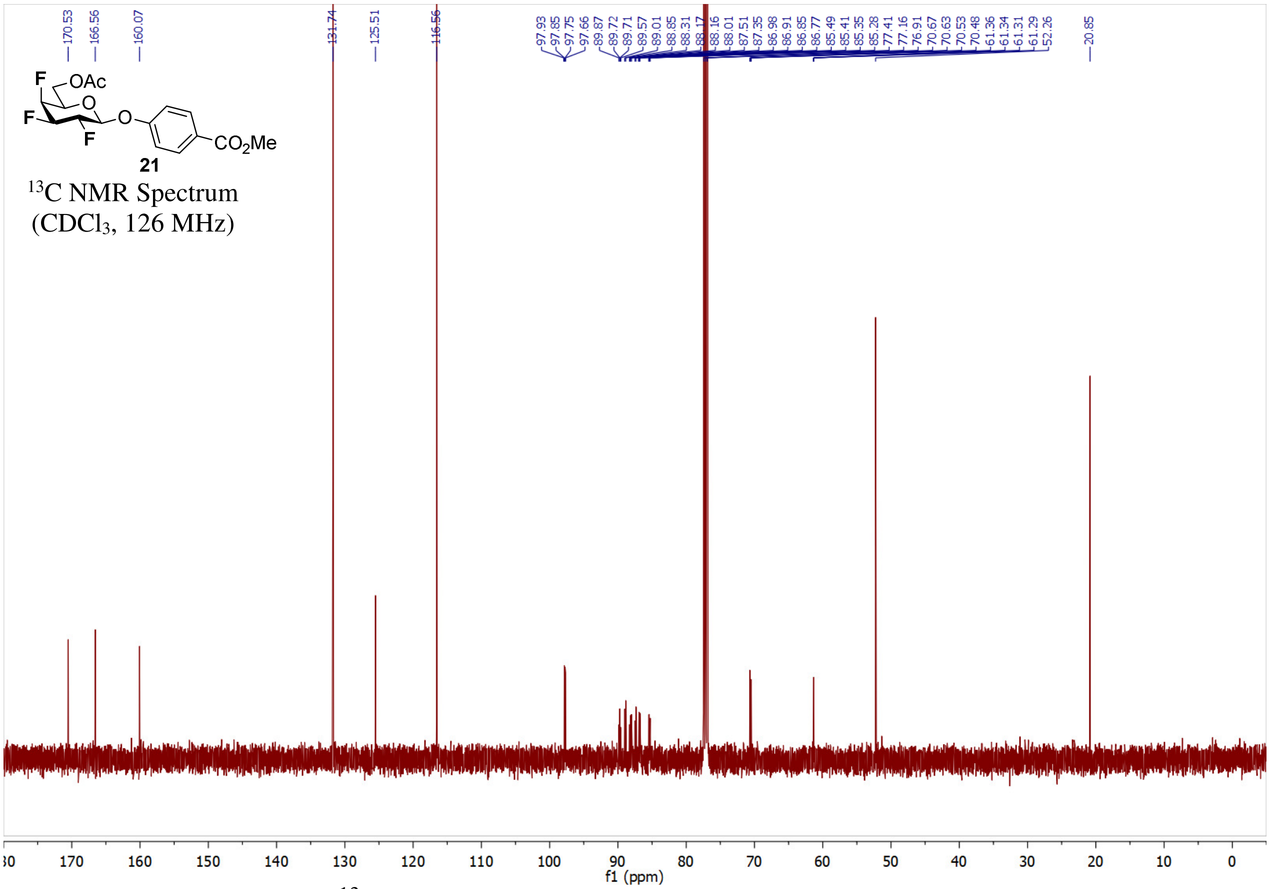

Supplementary Figure 21. ^{13}C NMR spectrum of compound **21**

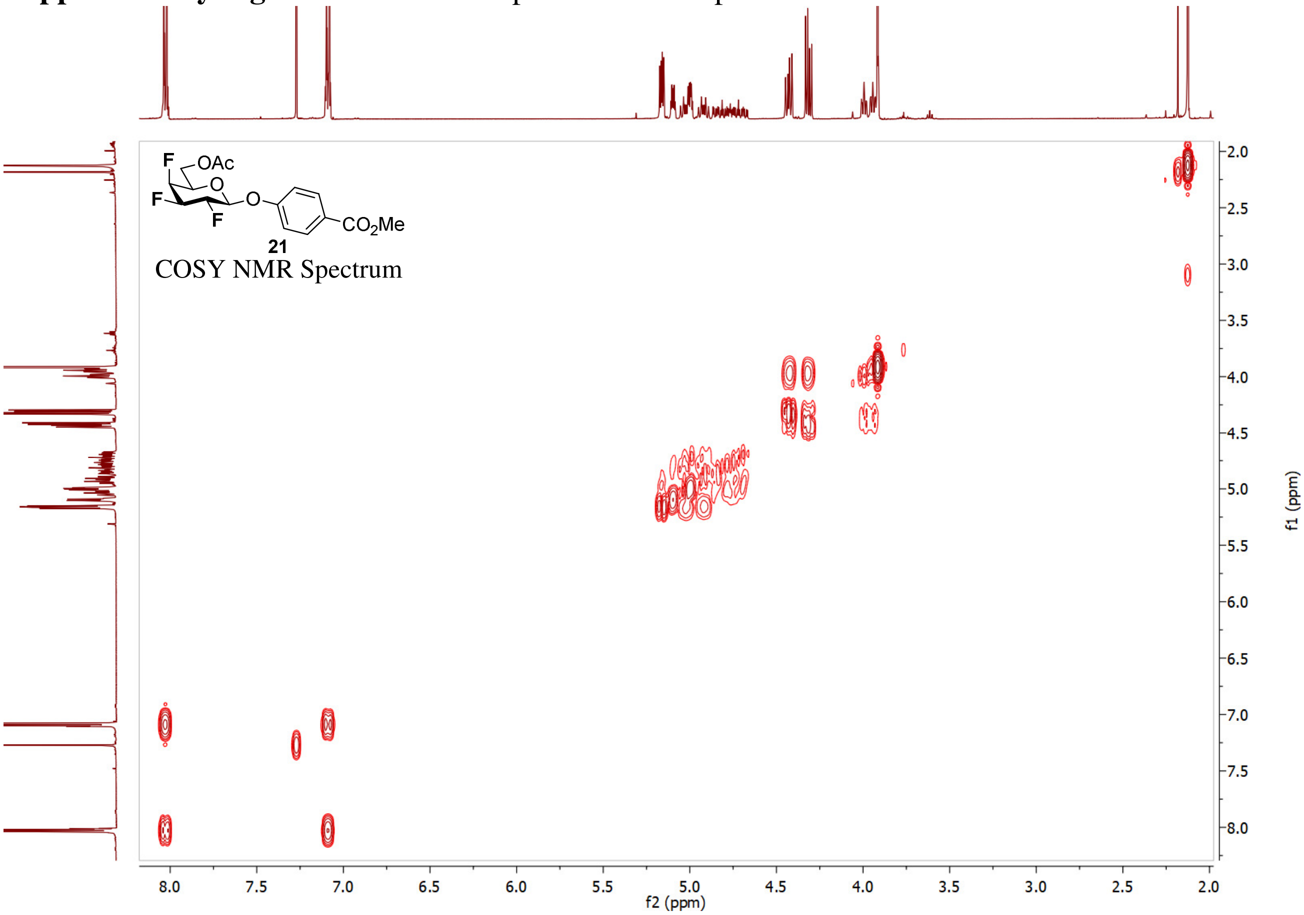

Supplementary Figure 22. COSY NMR spectrum of compound **21**

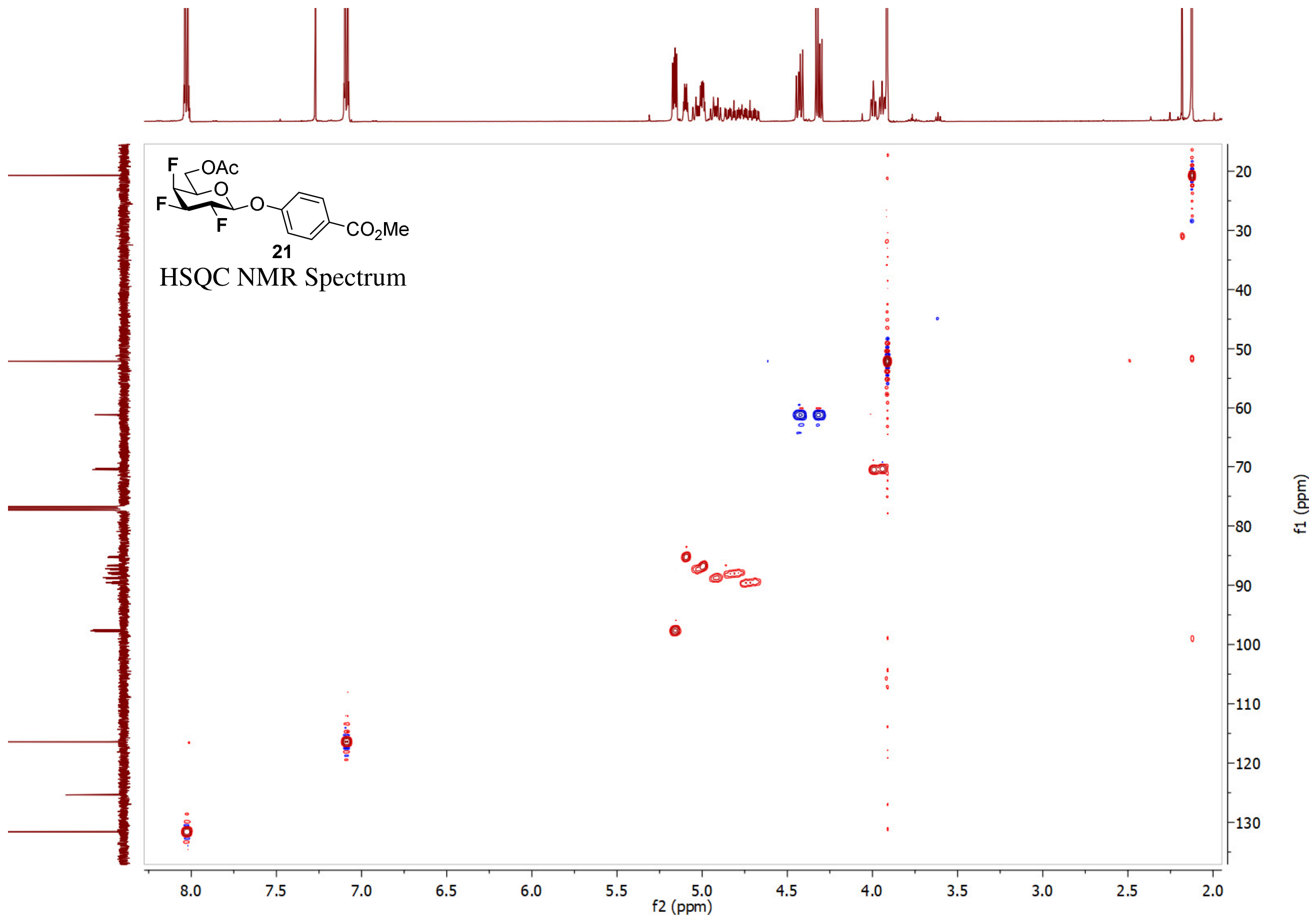

Supplementary Figure 23. HSQC NMR spectrum of compound **21**

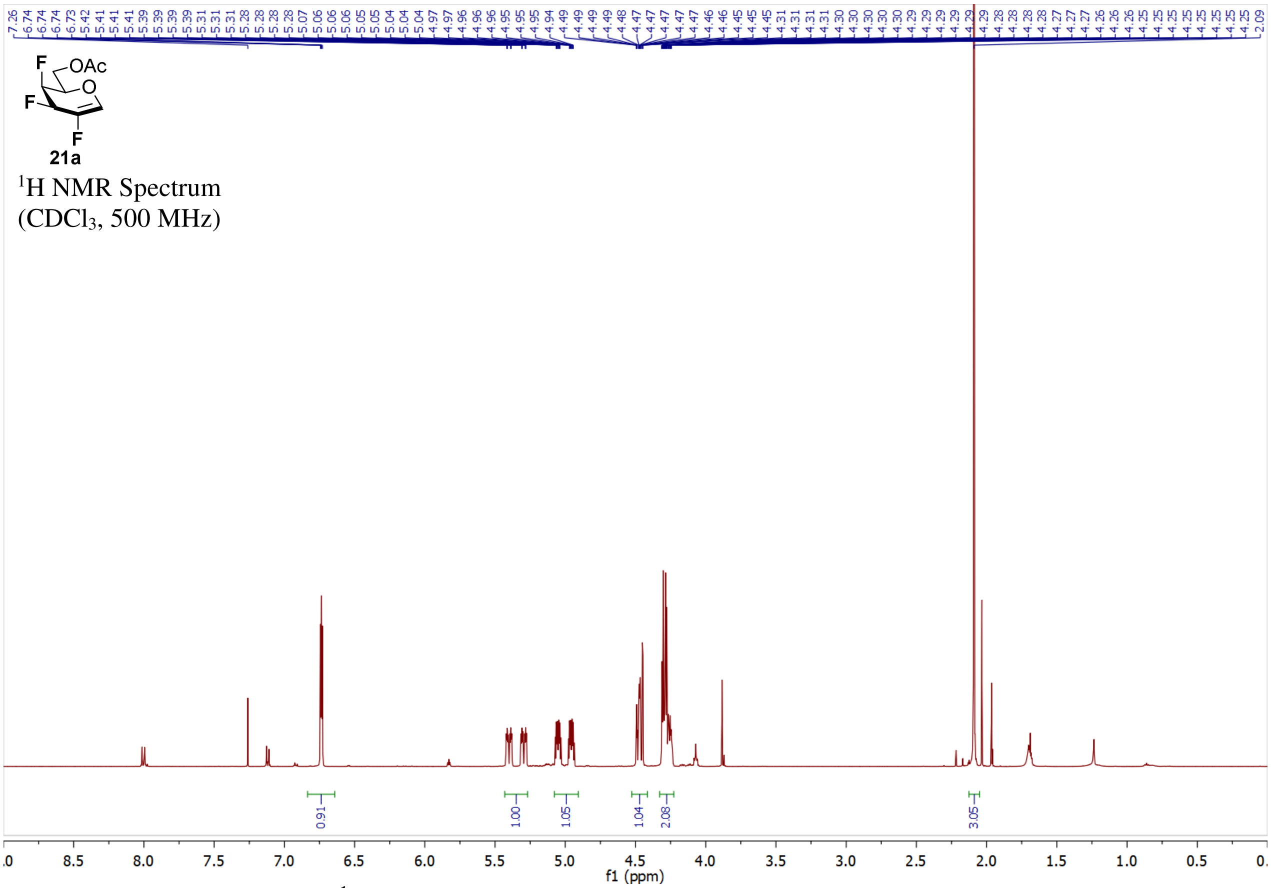

Supplementary Figure 24. ^{1}H NMR spectrum of compound **21a**

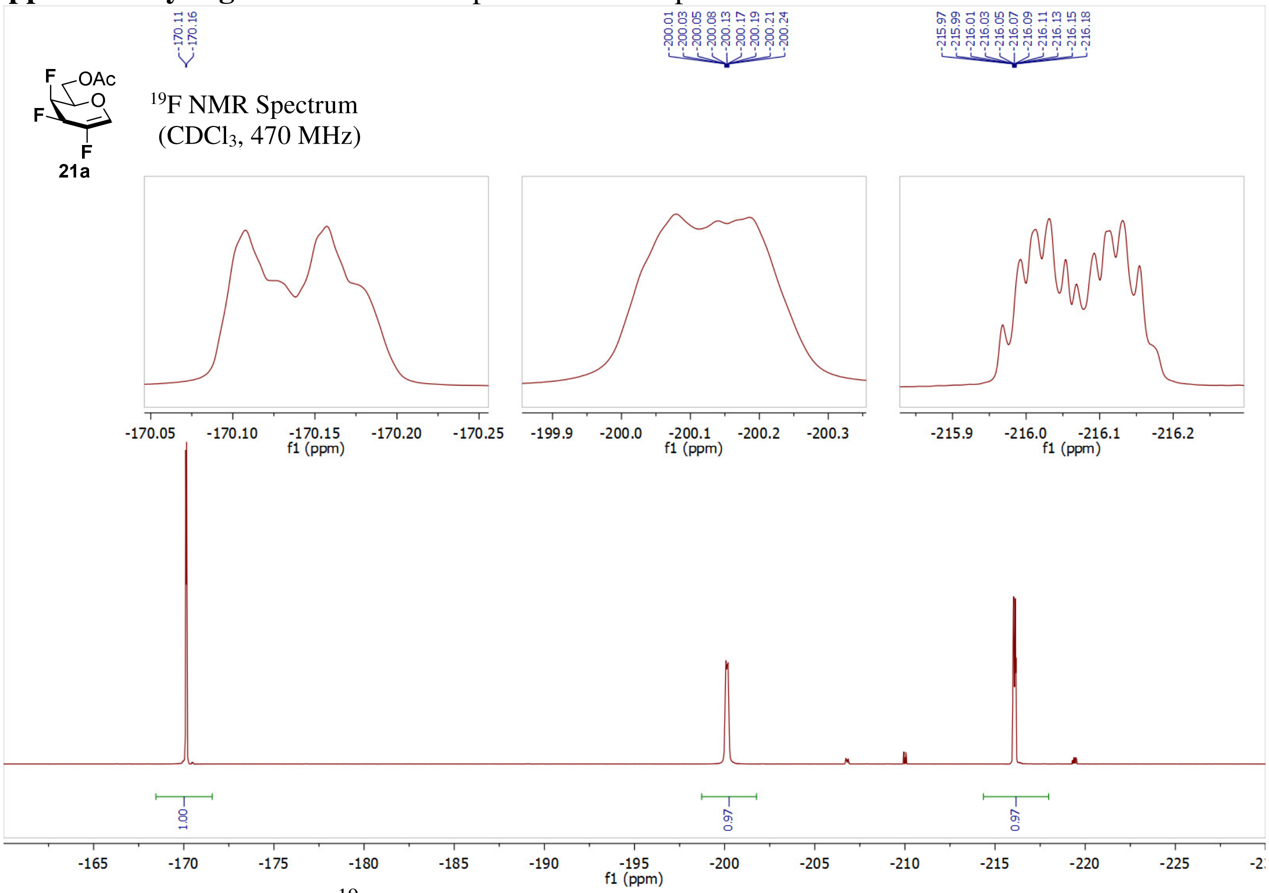

Supplementary Figure 25. ^{19}F NMR spectrum of compound **21a**

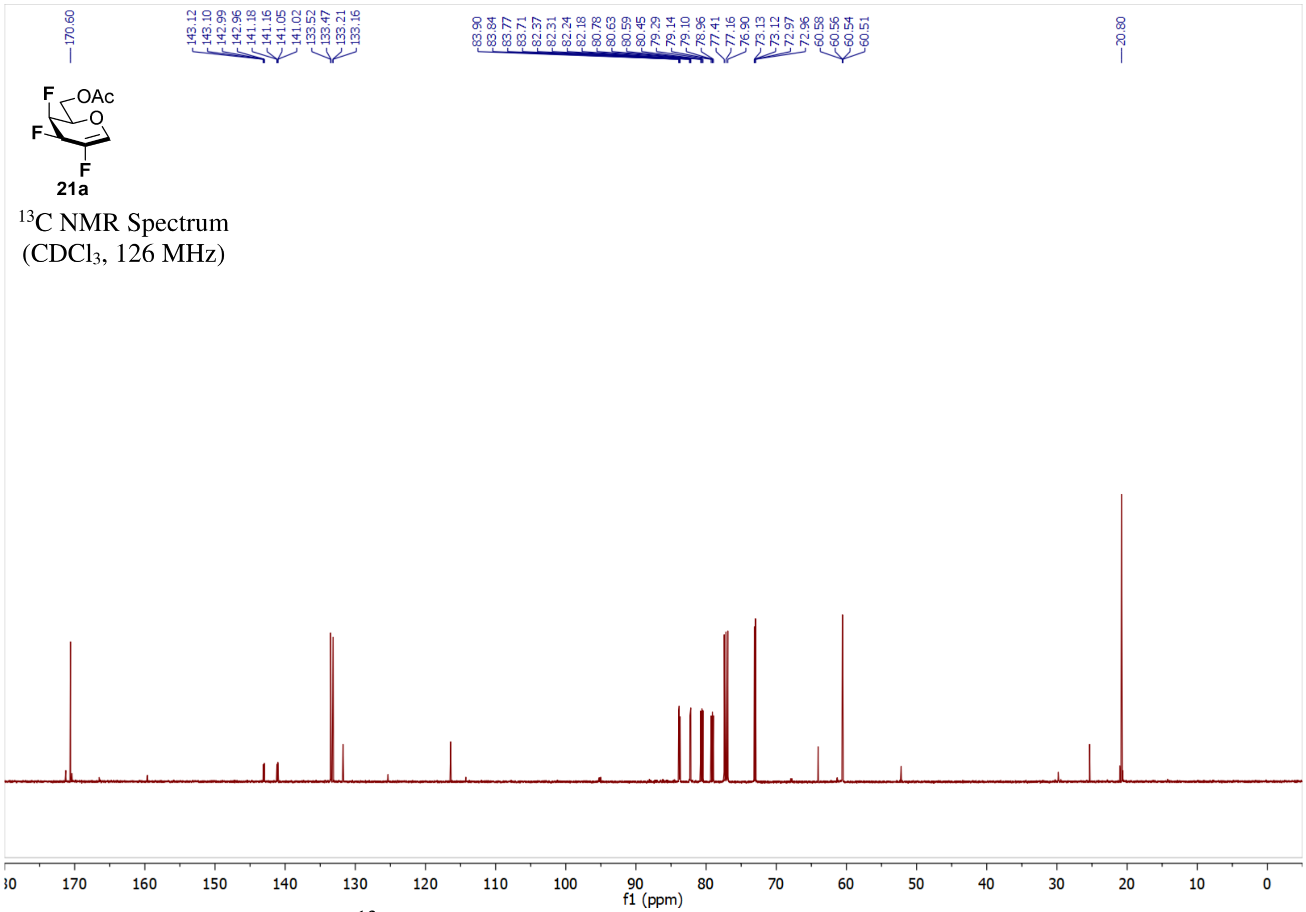

Supplementary Figure 26. ^{13}C NMR spectrum of compound **21a**

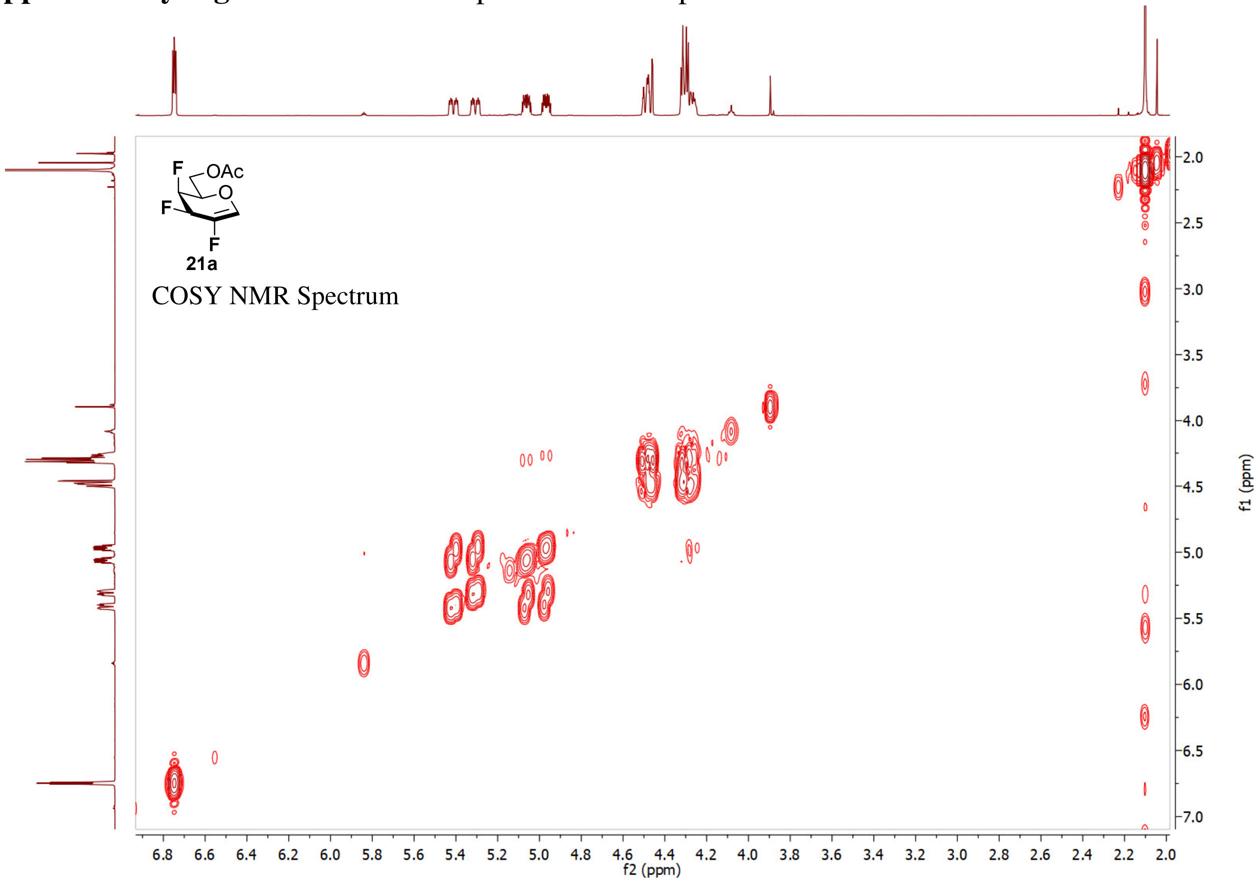

Supplementary Figure 27. COSY NMR spectrum of compound **21a**

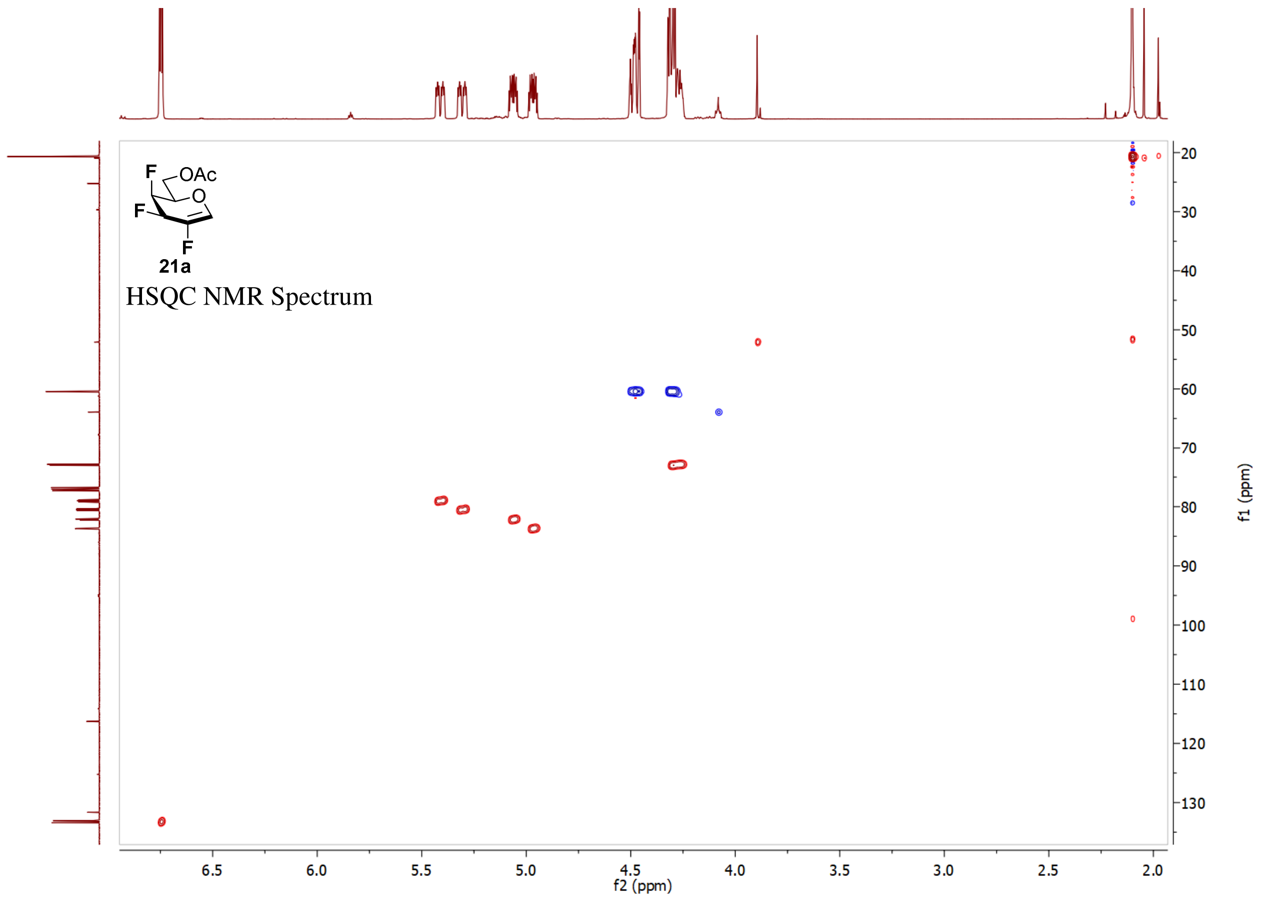

Supplementary Figure 28. HSQC NMR spectrum of compound **21a**

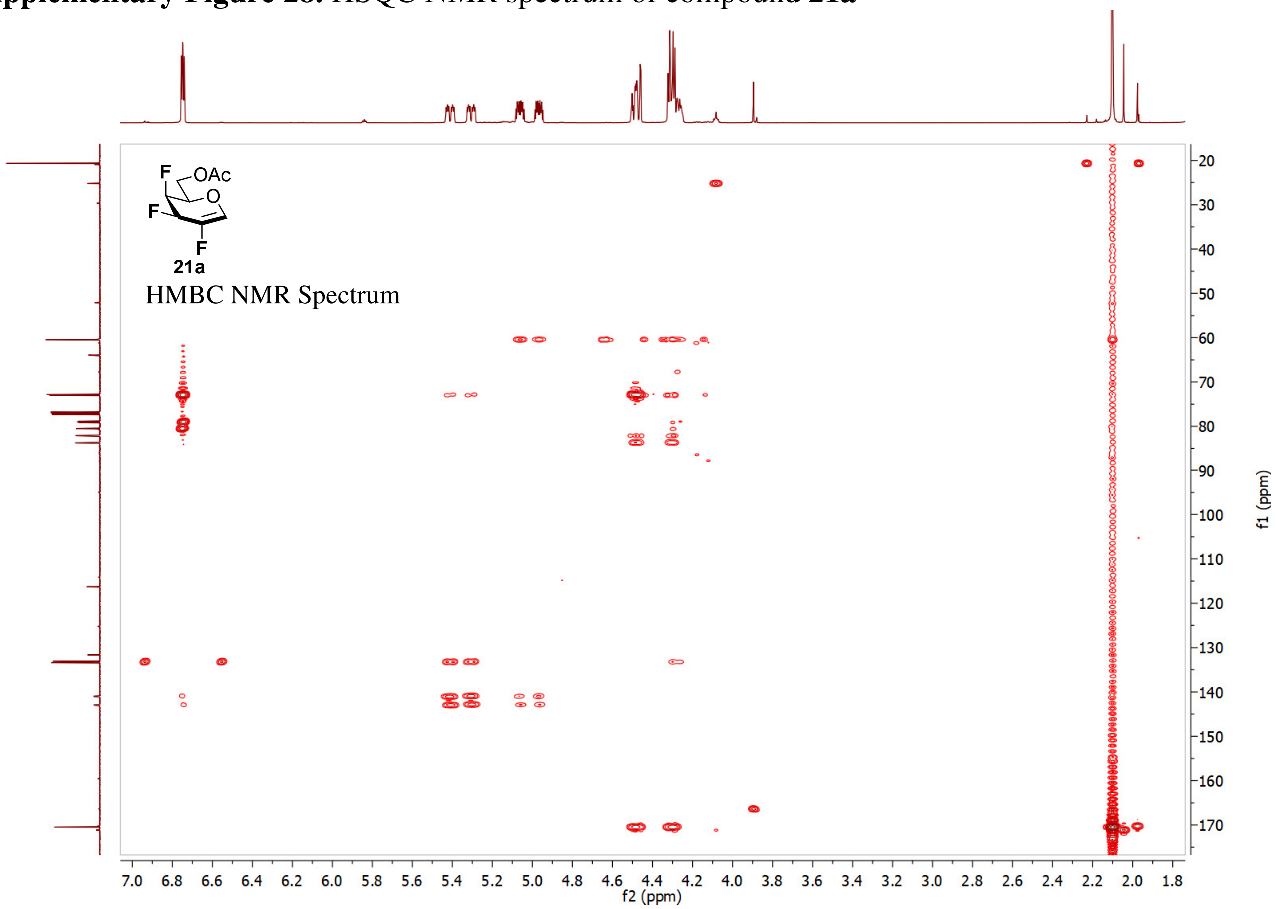

Supplementary Figure 29. HMBC NMR spectrum of compound **21a**

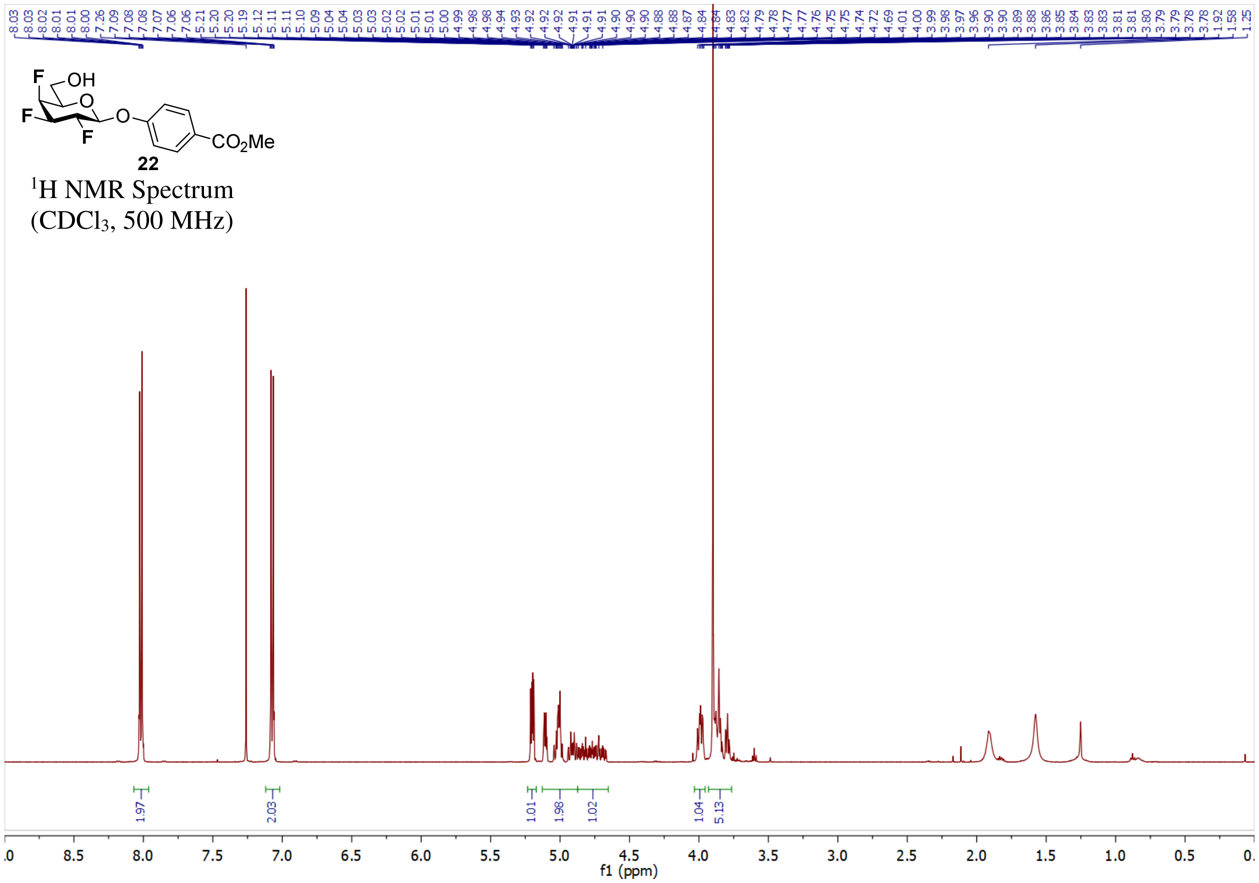

Supplementary Figure 30. ^{1}H NMR spectrum of compound **22**

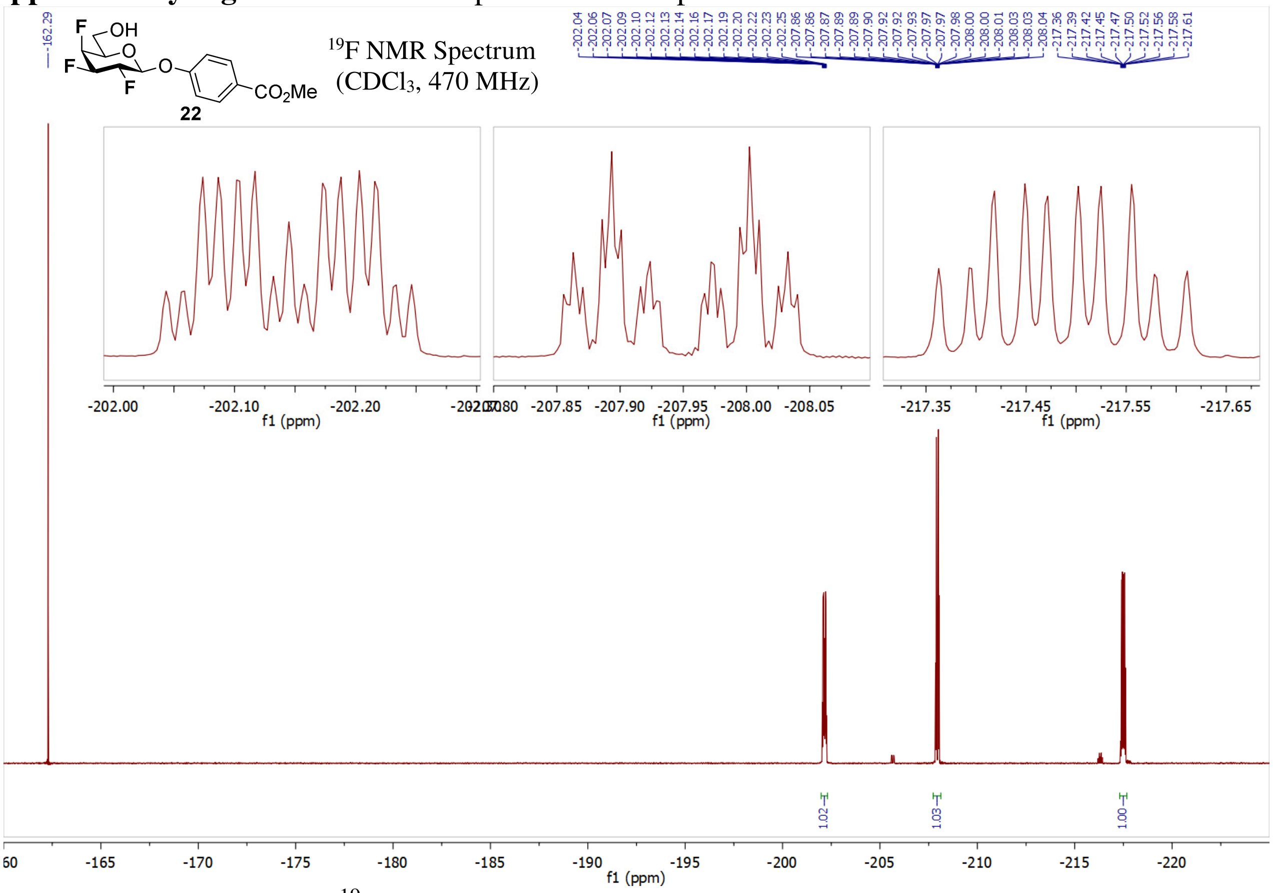

Supplementary Figure 31. ^{19}F NMR spectrum of compound **22**

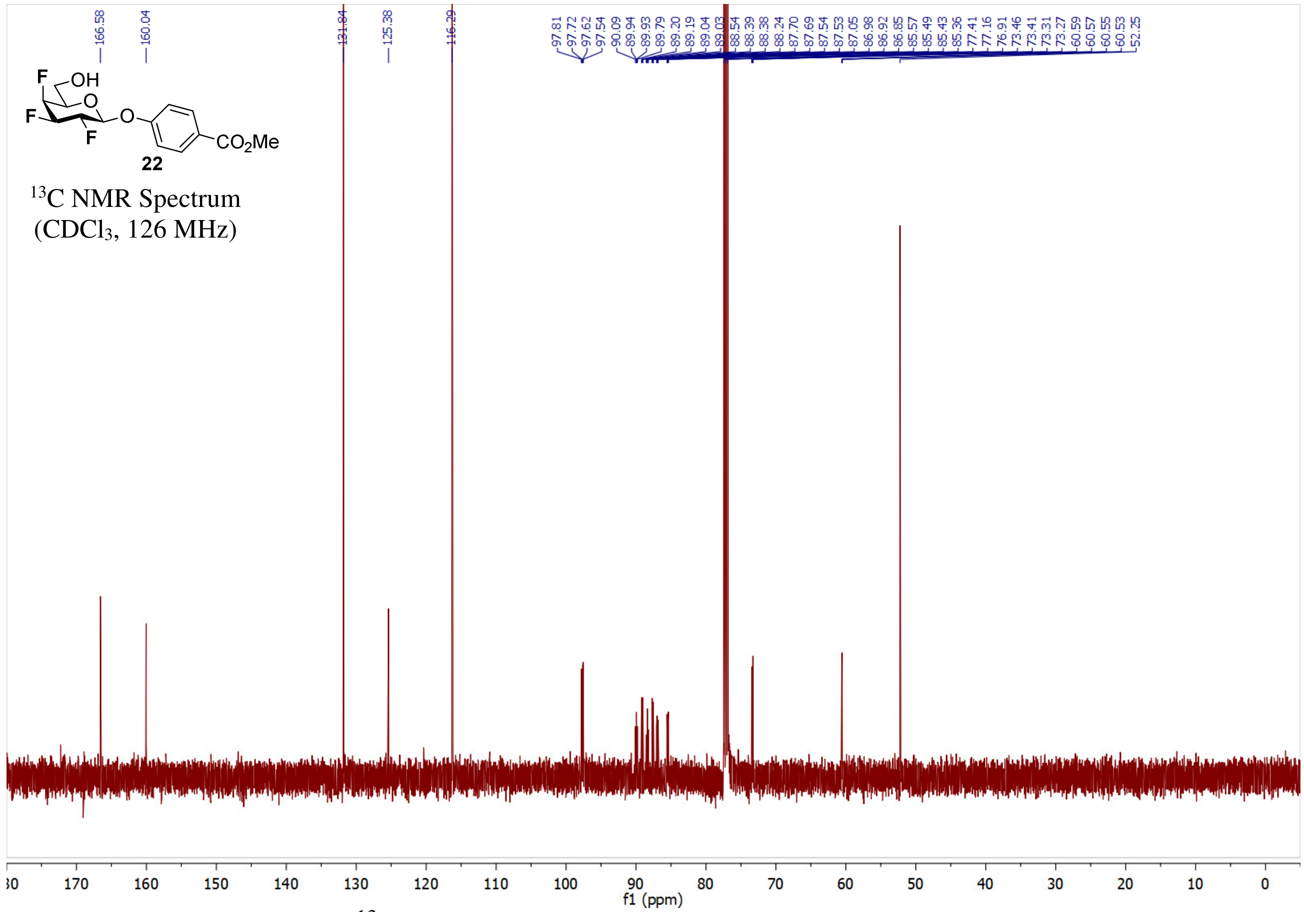

Supplementary Figure 32. ^{13}C NMR spectrum of compound **22**

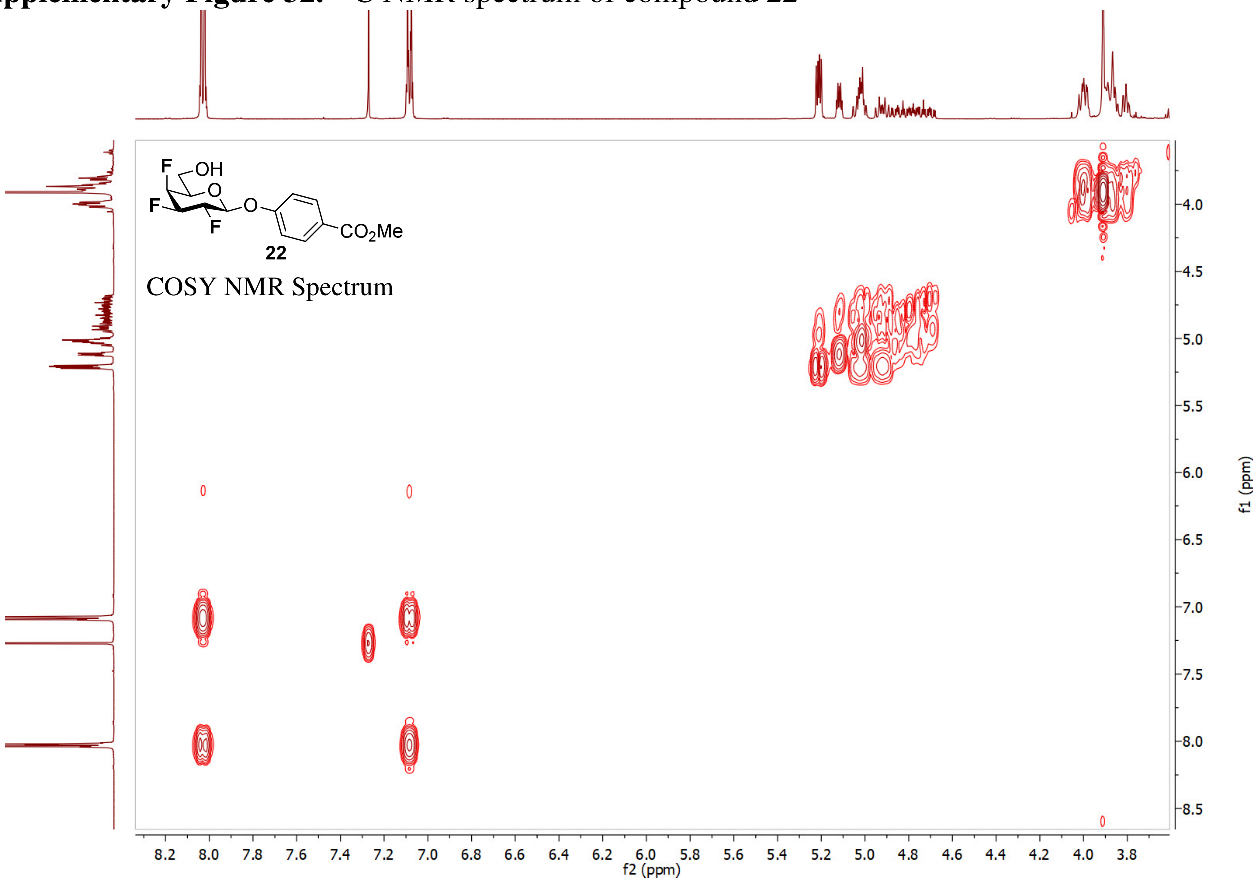

Supplementary Figure 33. COSY NMR spectrum of compound **22**

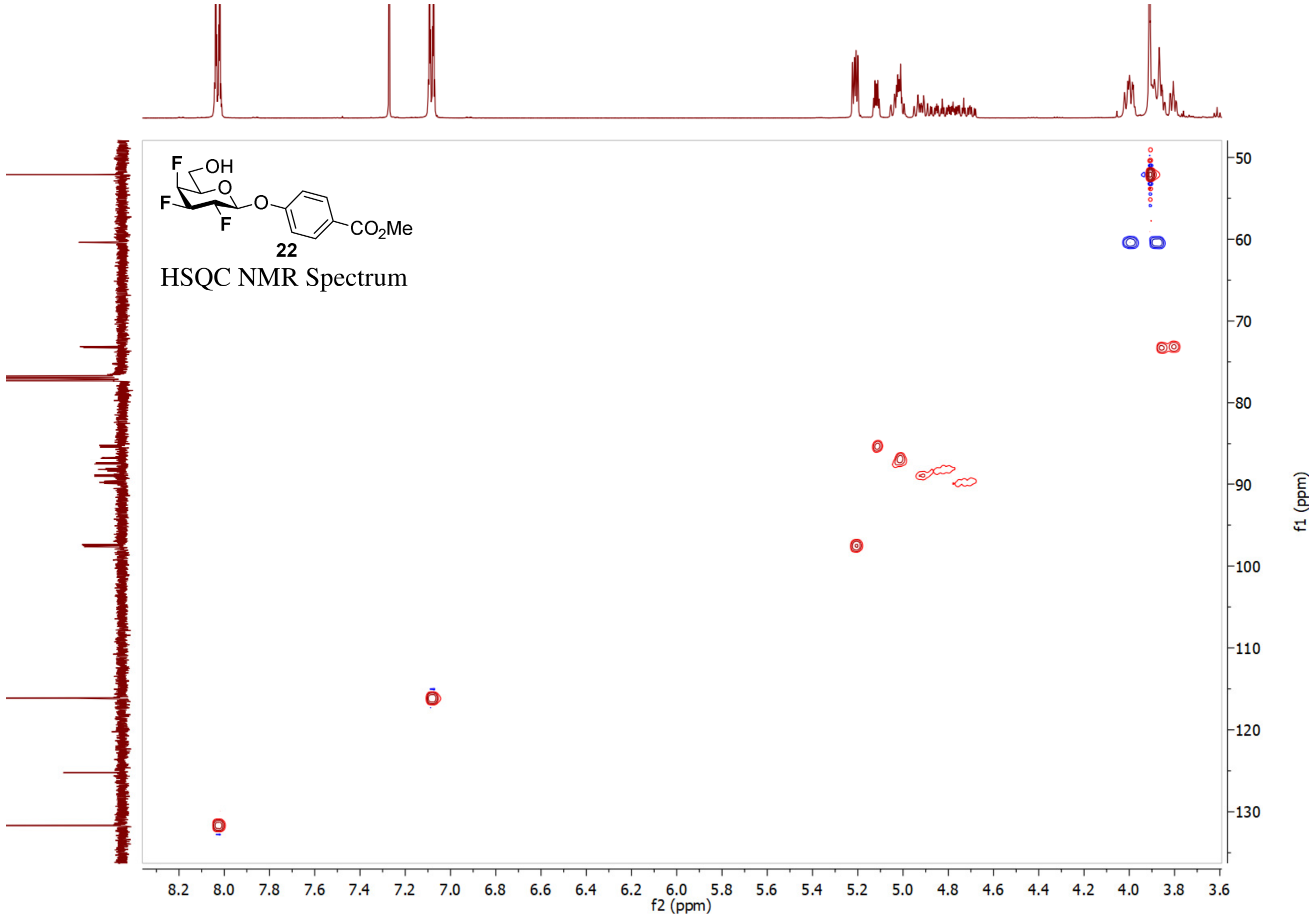

Supplementary Figure 34. HSQC NMR spectrum of compound **22**

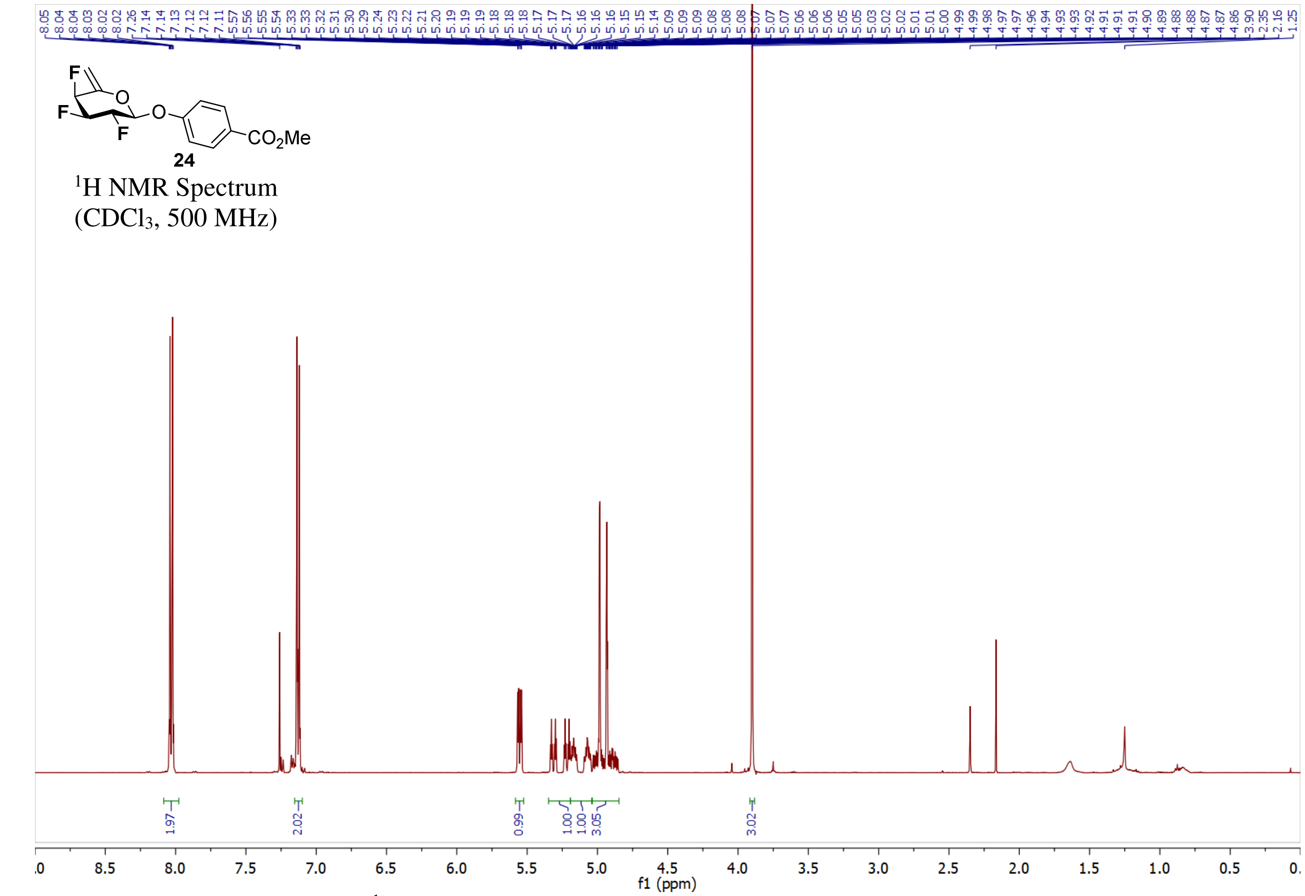

Supplementary Figure 35. ^{1}H NMR spectrum of compound **24**

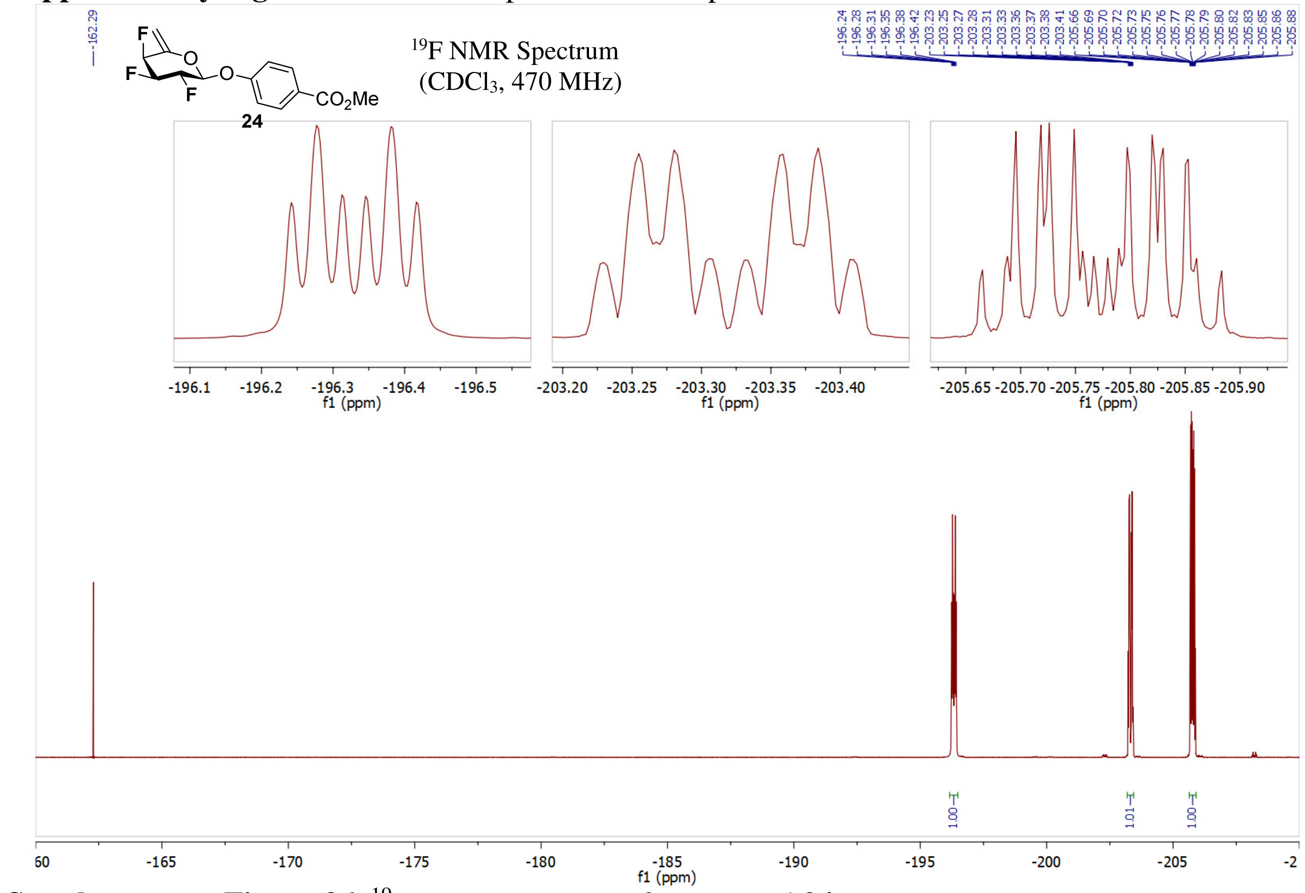

Supplementary Figure 36. ^{19}F NMR spectrum of compound **24**

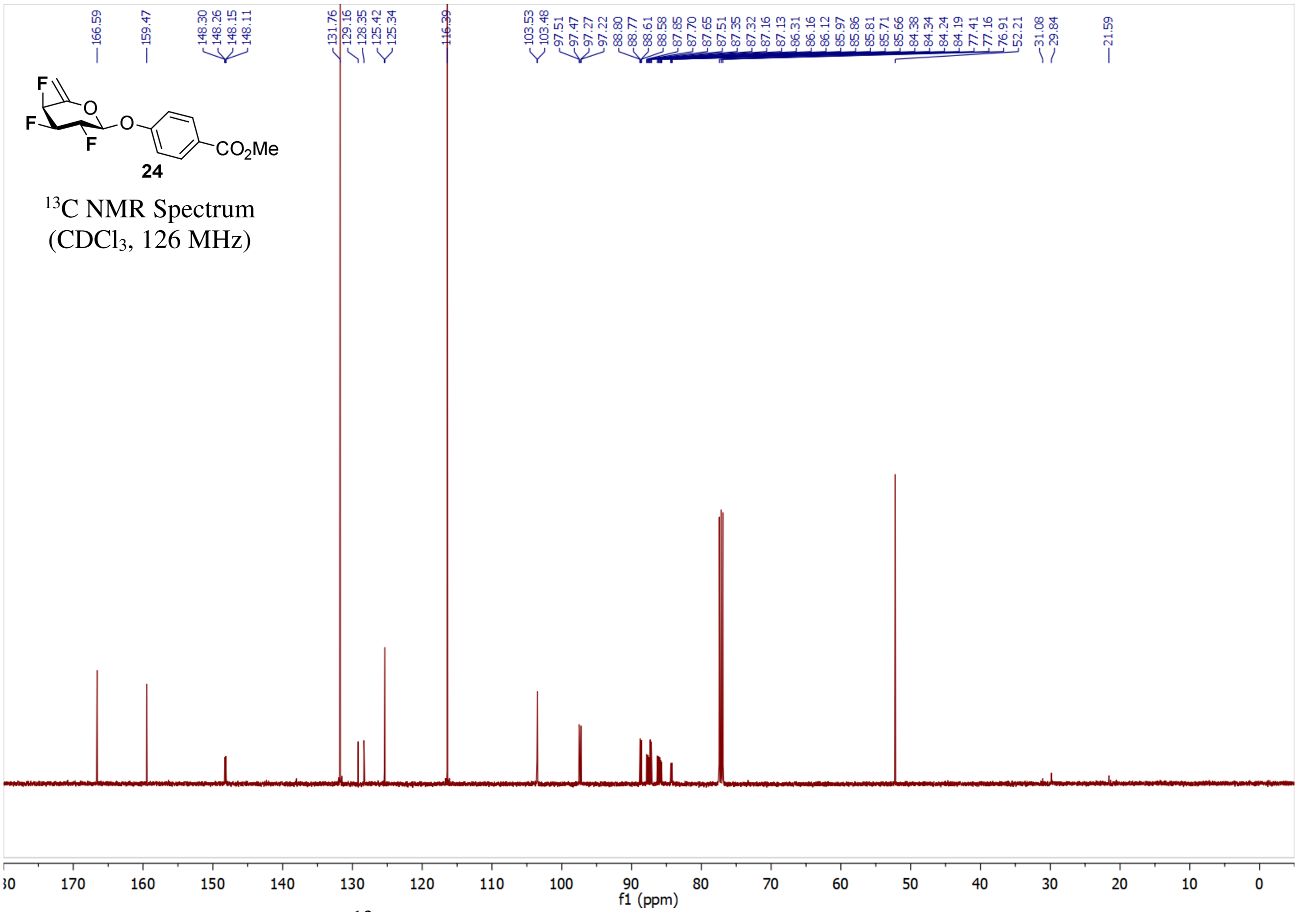

Supplementary Figure 37. ^{13}C NMR spectrum of compound **24**

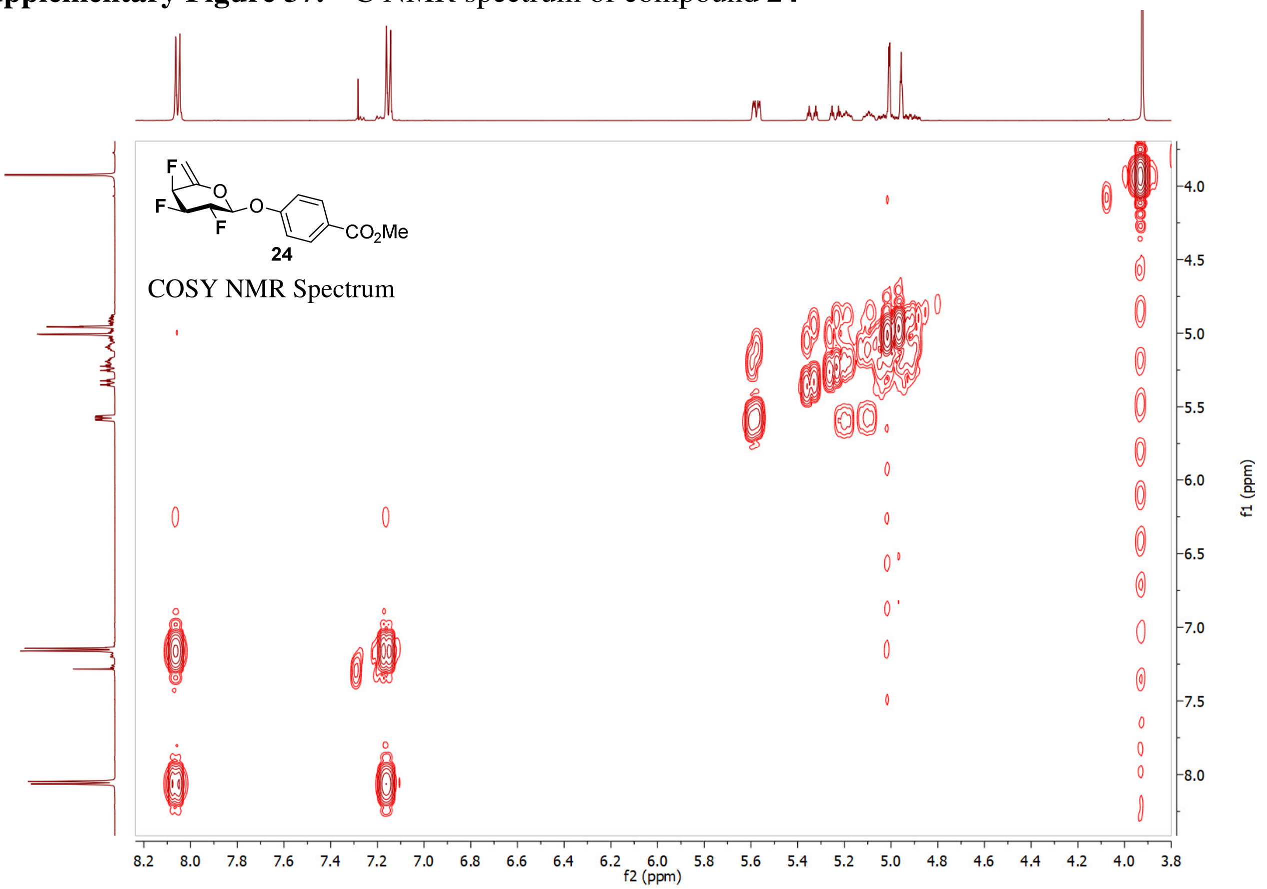

Supplementary Figure 38. COSY NMR spectrum of compound **24**

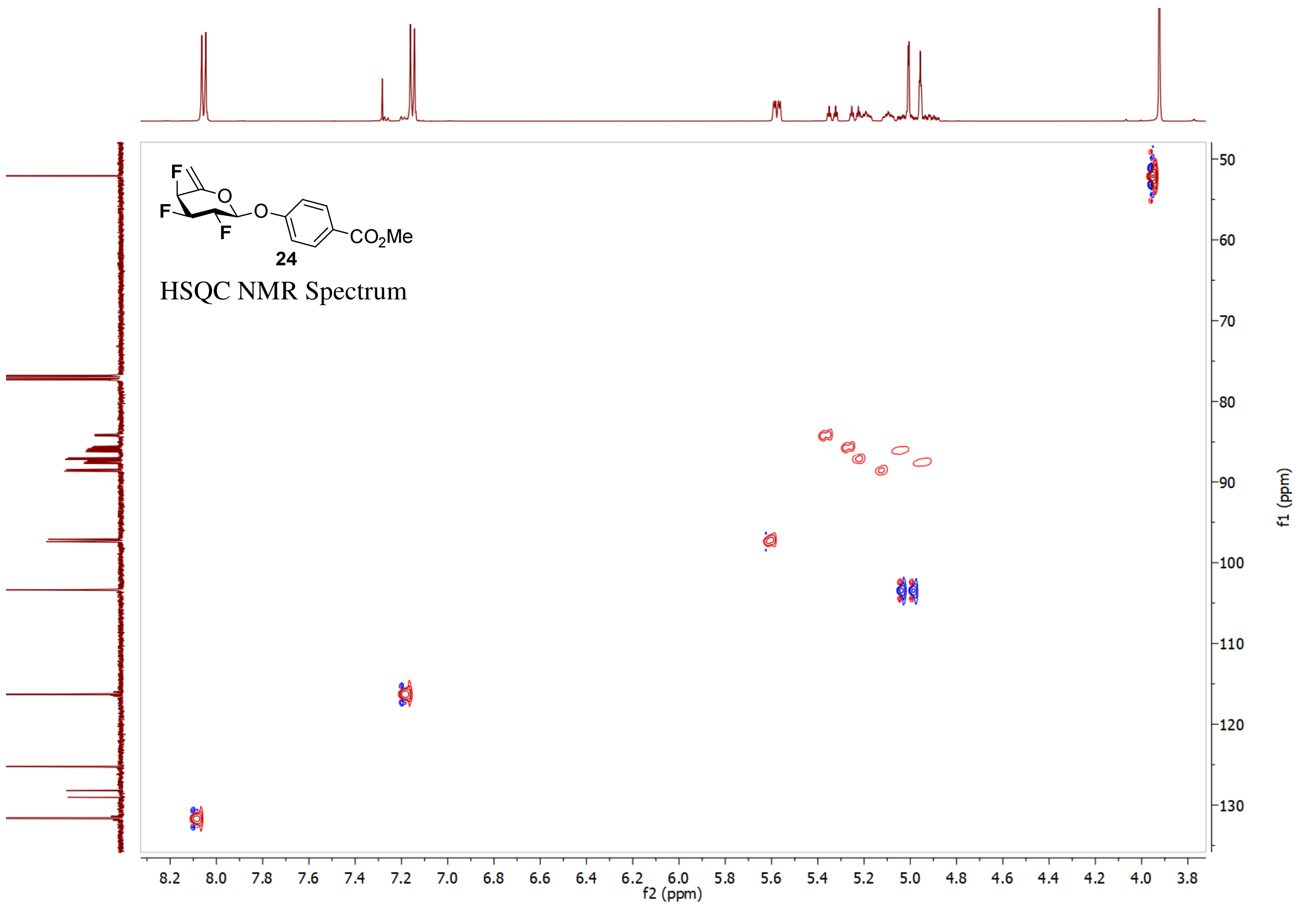

Supplementary Figure 39. HSQC NMR spectrum of compound **24**

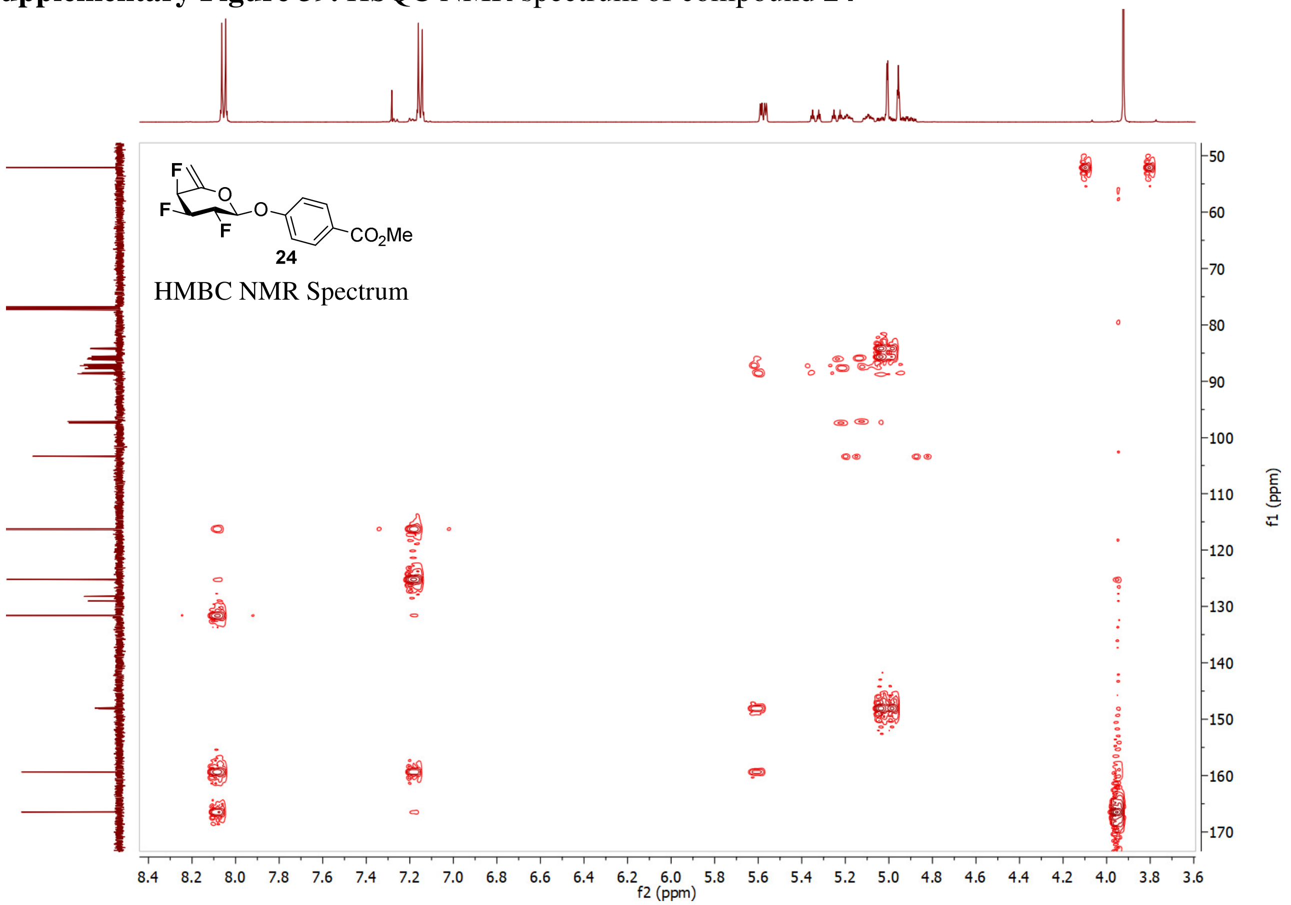

Supplementary Figure 40. HMBC NMR spectrum of compound **24**

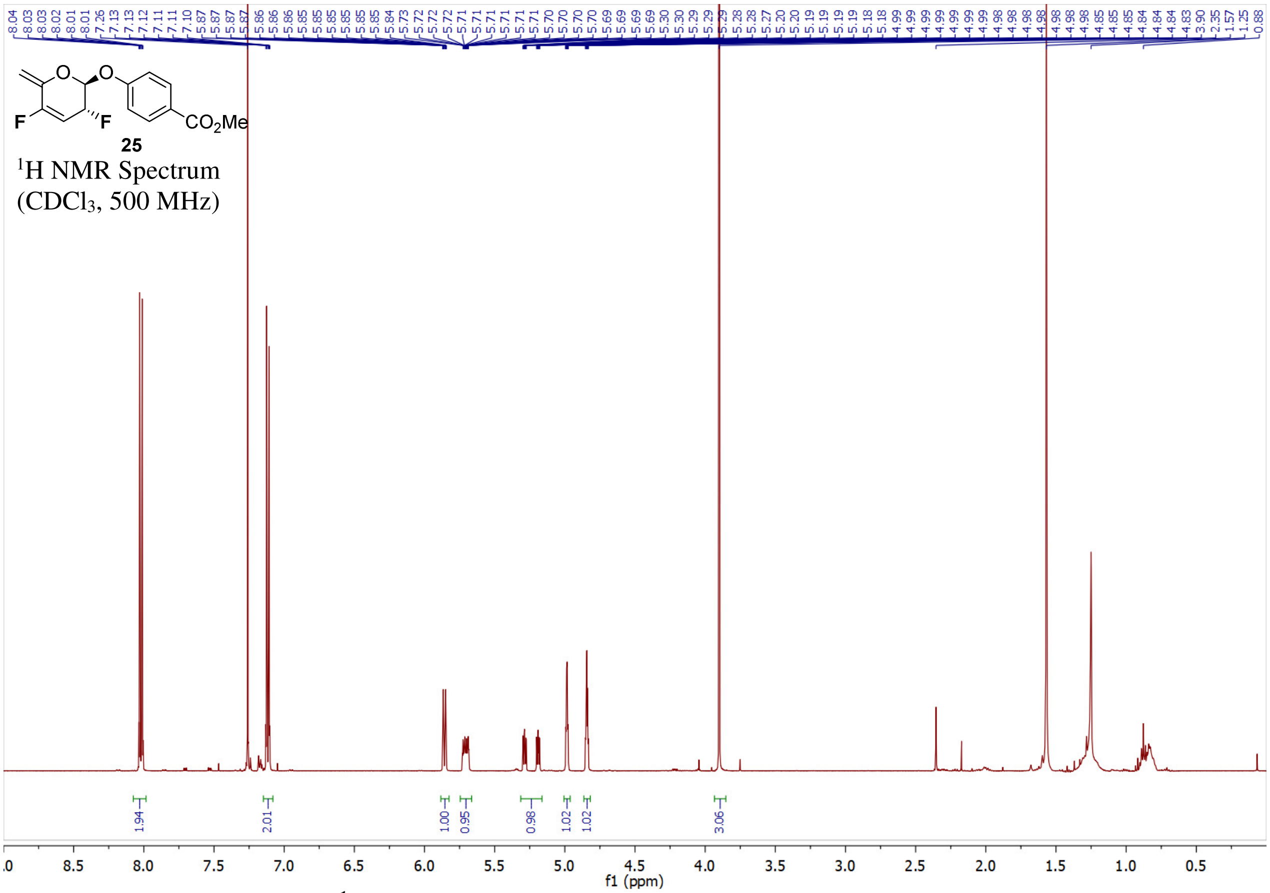

Supplementary Figure 41. ^{1}H NMR spectrum of compound **25**

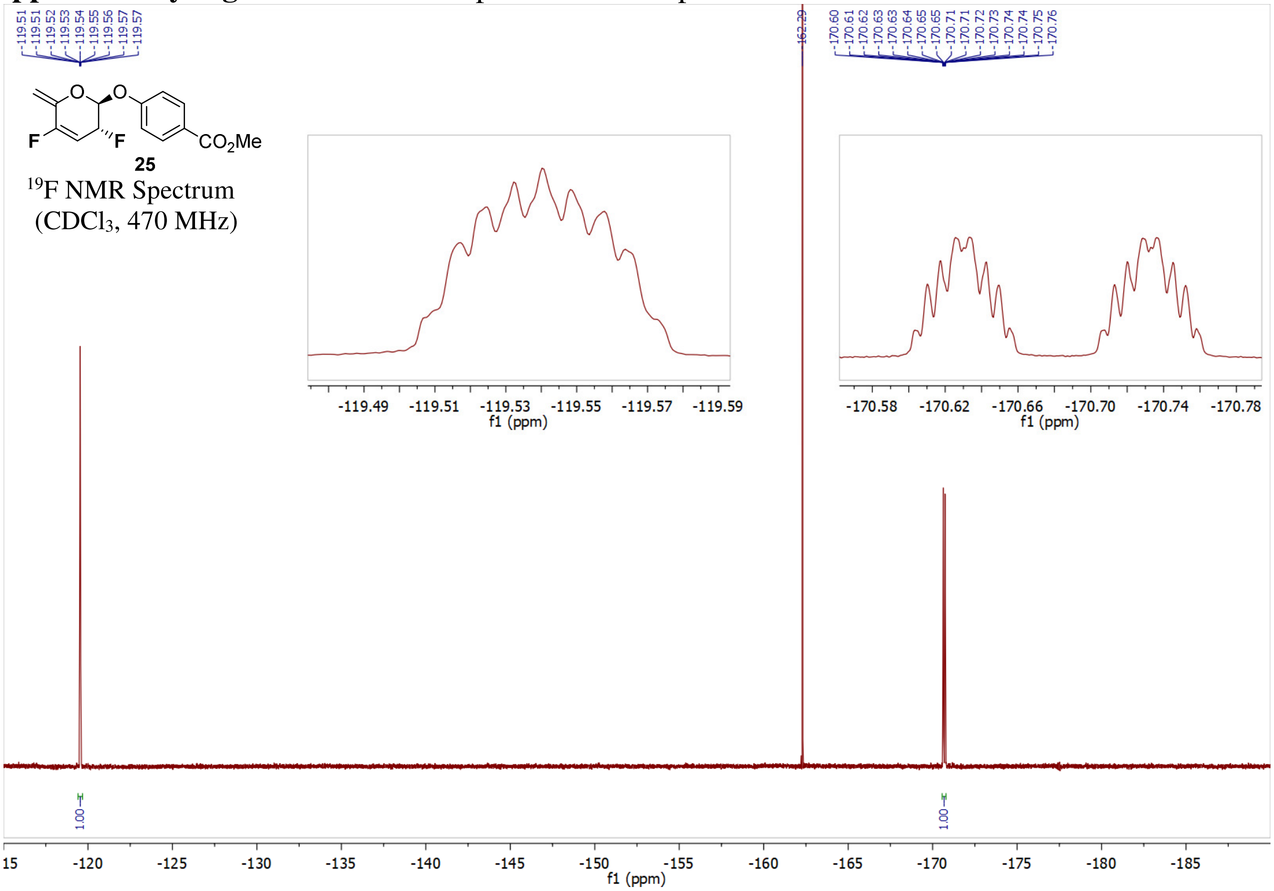

Supplementary Figure 42. ^{19}F NMR spectrum of compound **25**

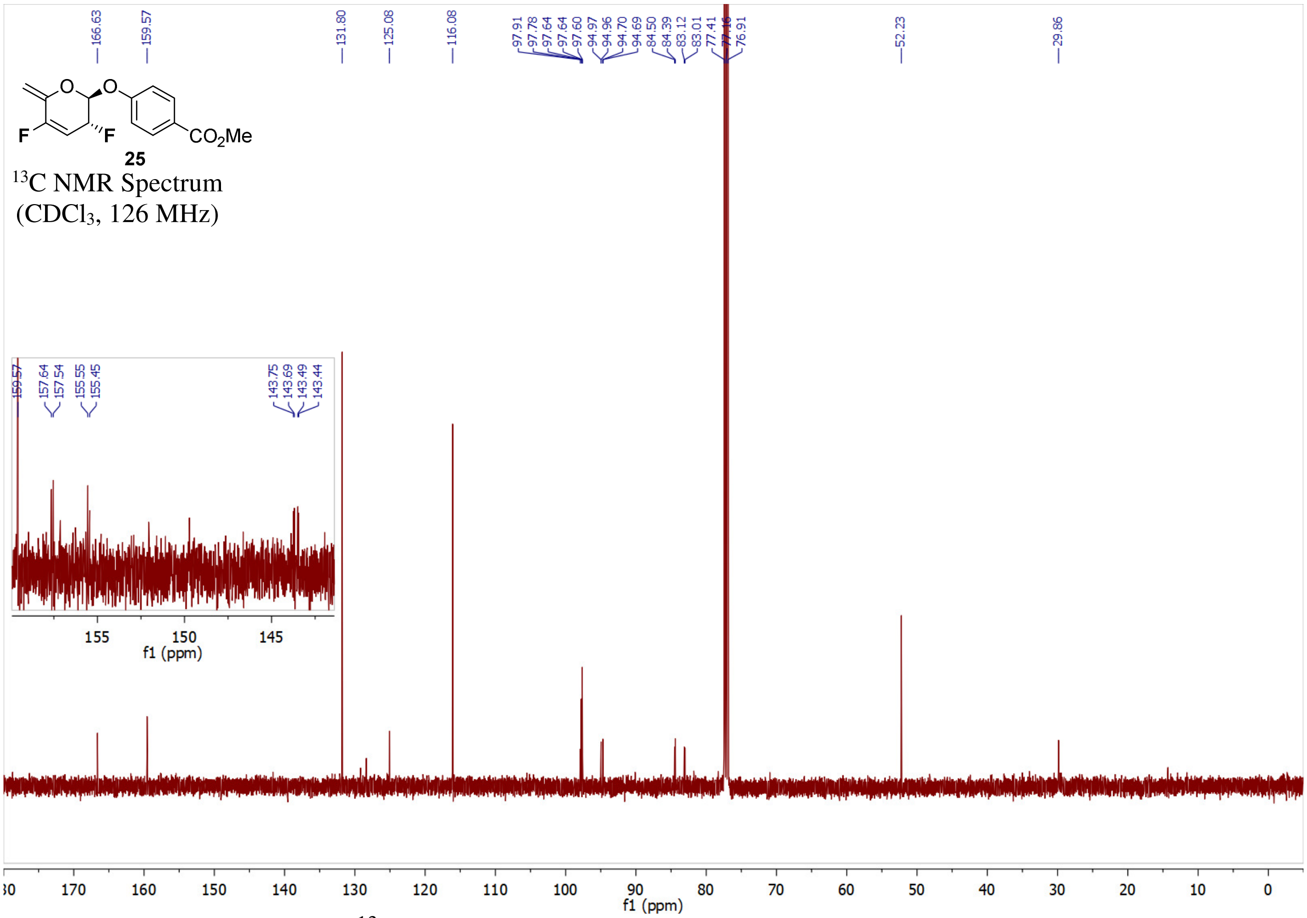

Supplementary Figure 43. ^{13}C NMR spectrum of compound **25**

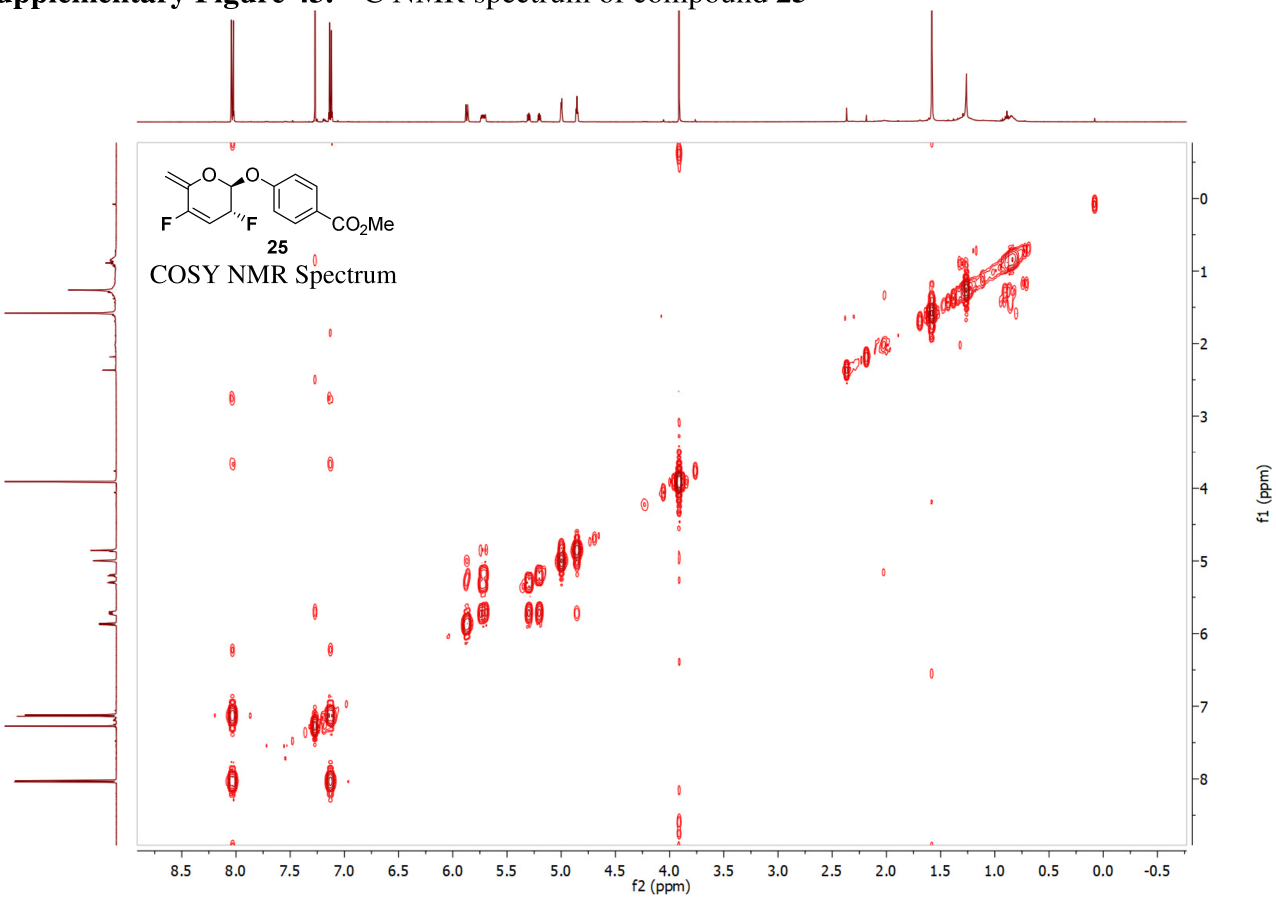

Supplementary Figure 44. COSY NMR spectrum of compound **25**

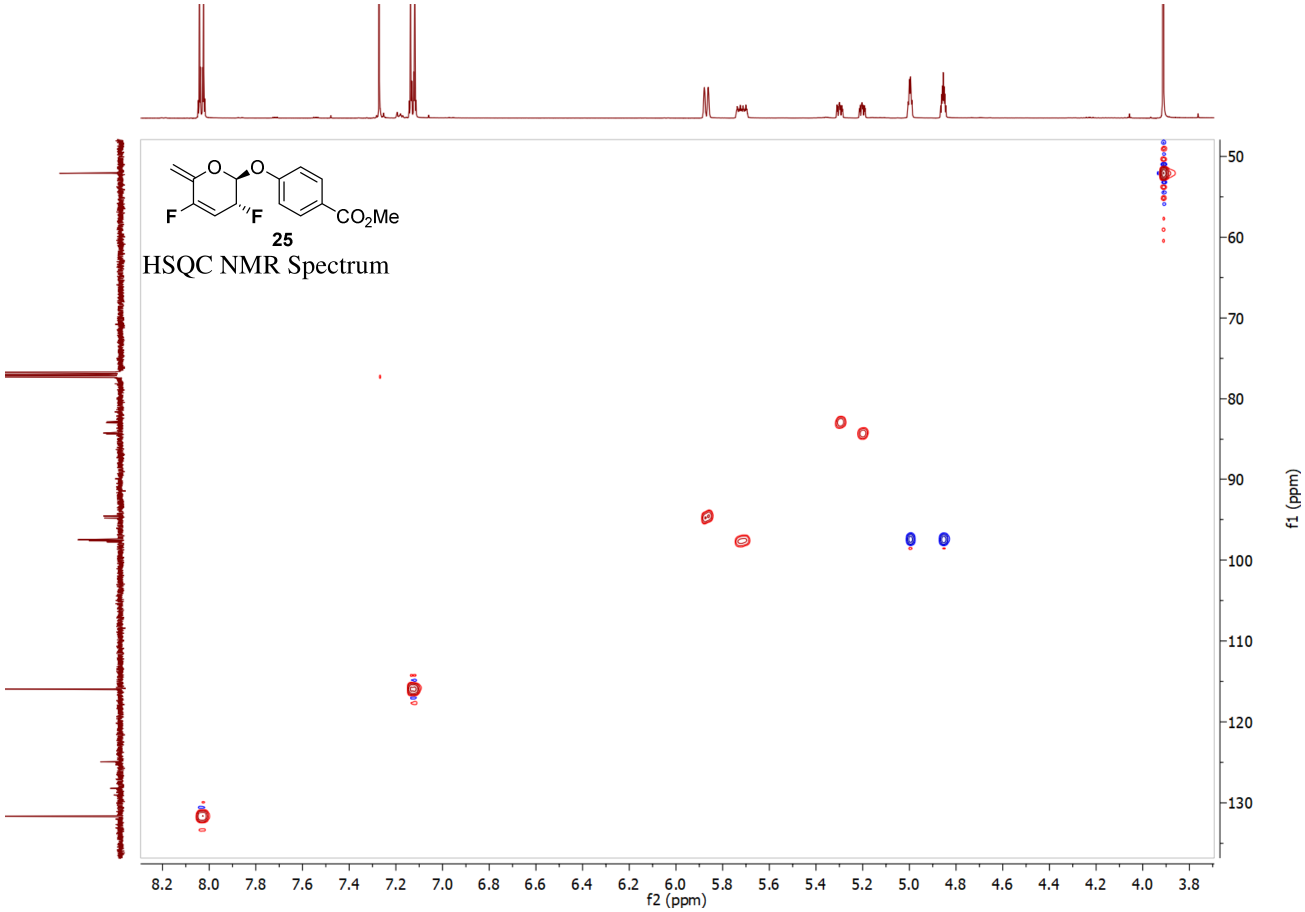

Supplementary Figure 45. HSQC NMR spectrum of compound **25**

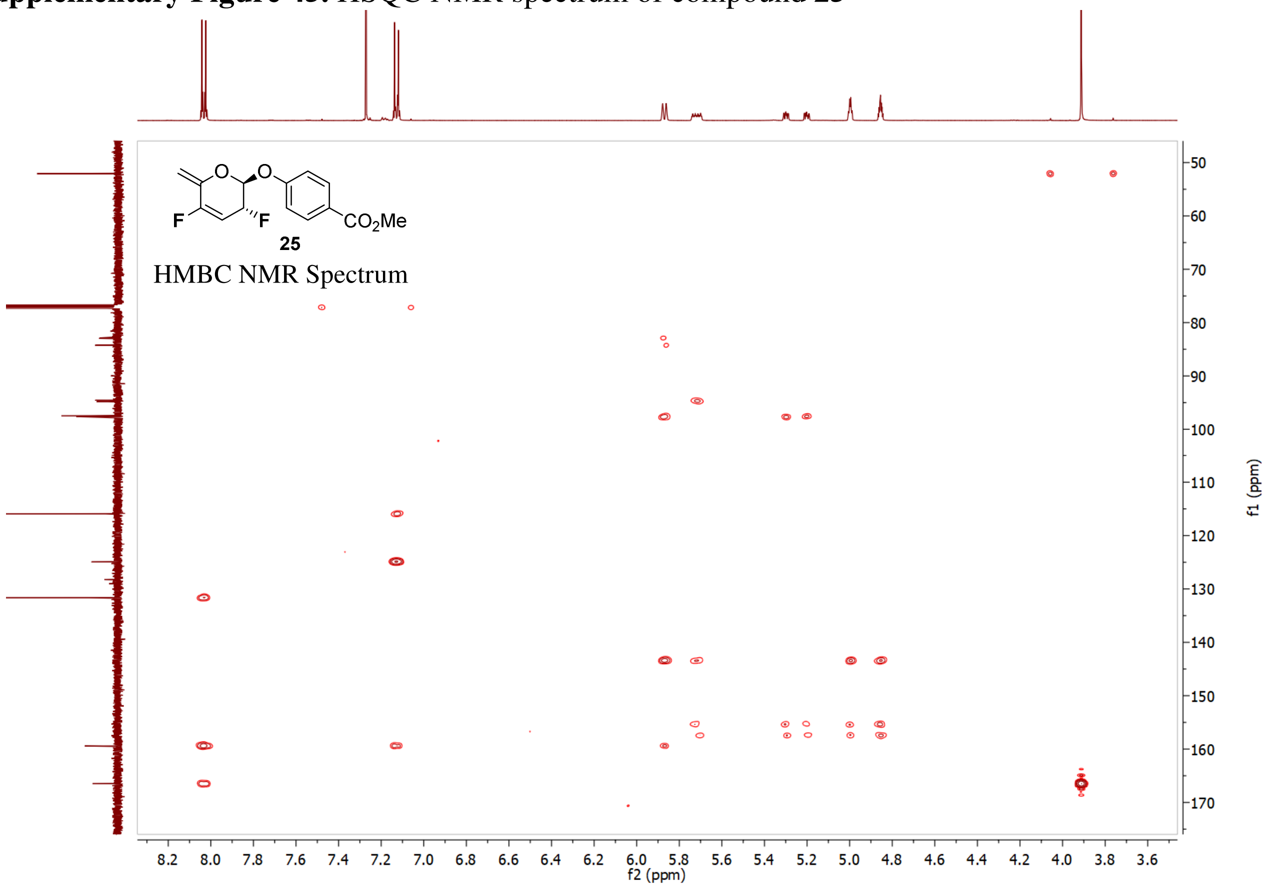

Supplementary Figure 46. HMBC NMR spectrum of compound **25**

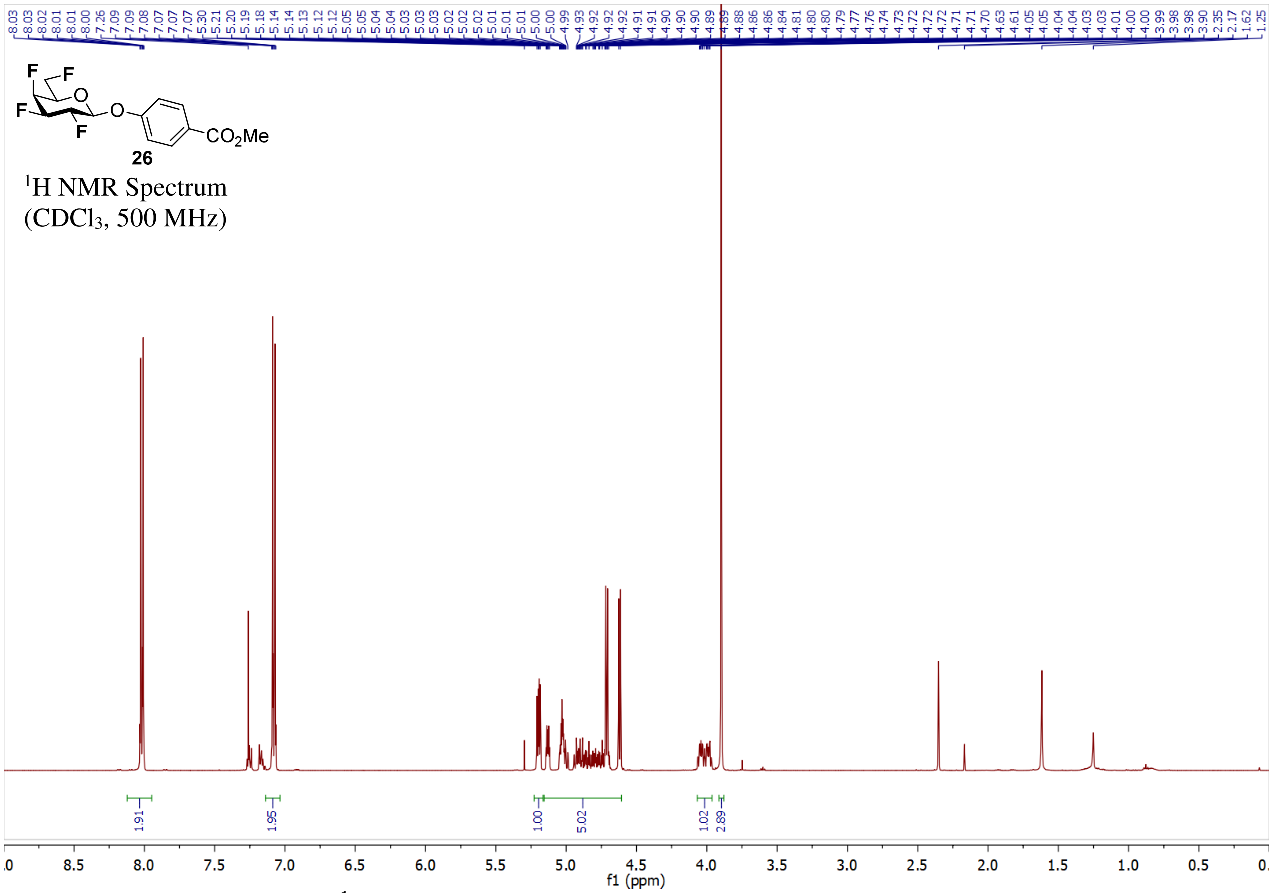

Supplementary Figure 47. ^{1}H NMR spectrum of compound **26**

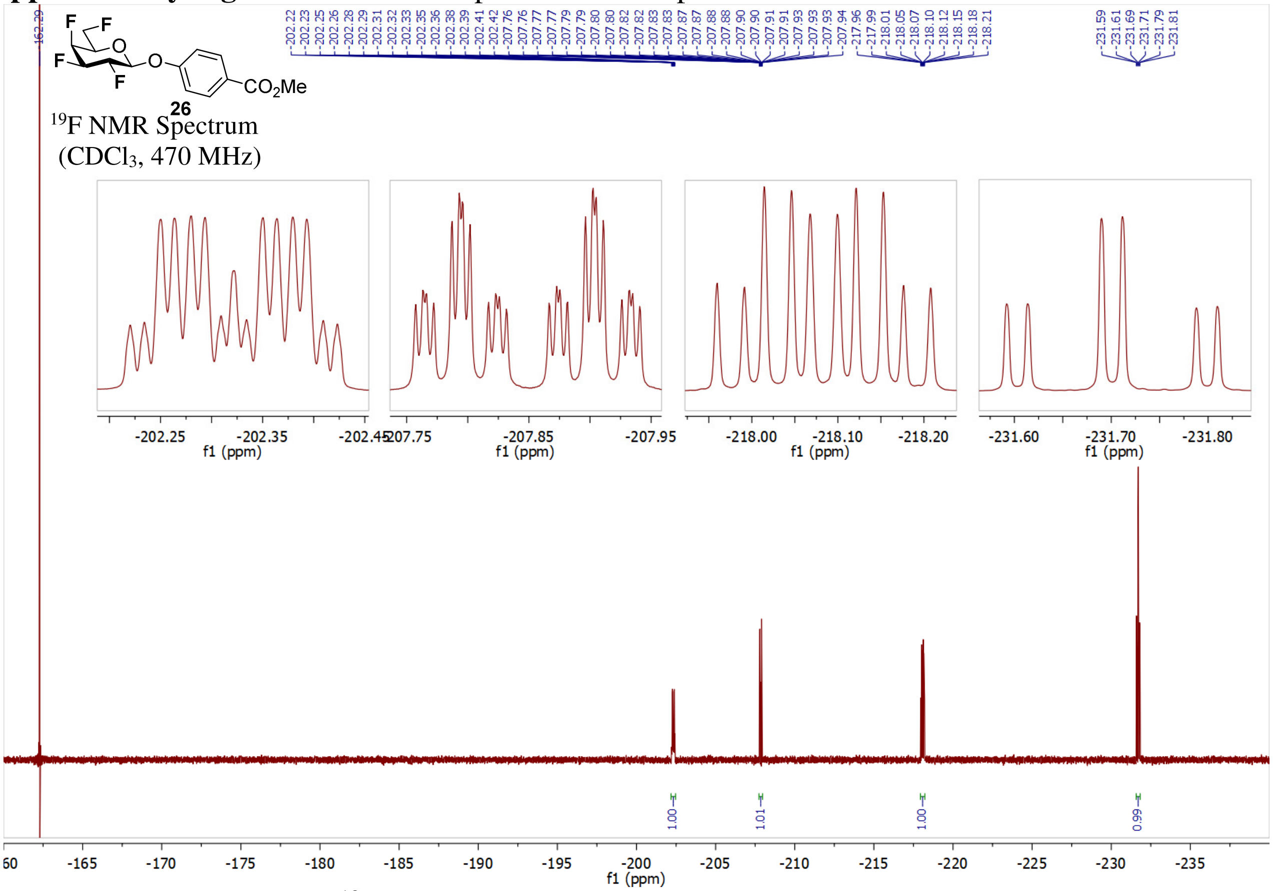

Supplementary Figure 48. ^{19}F NMR spectrum of compound **26**

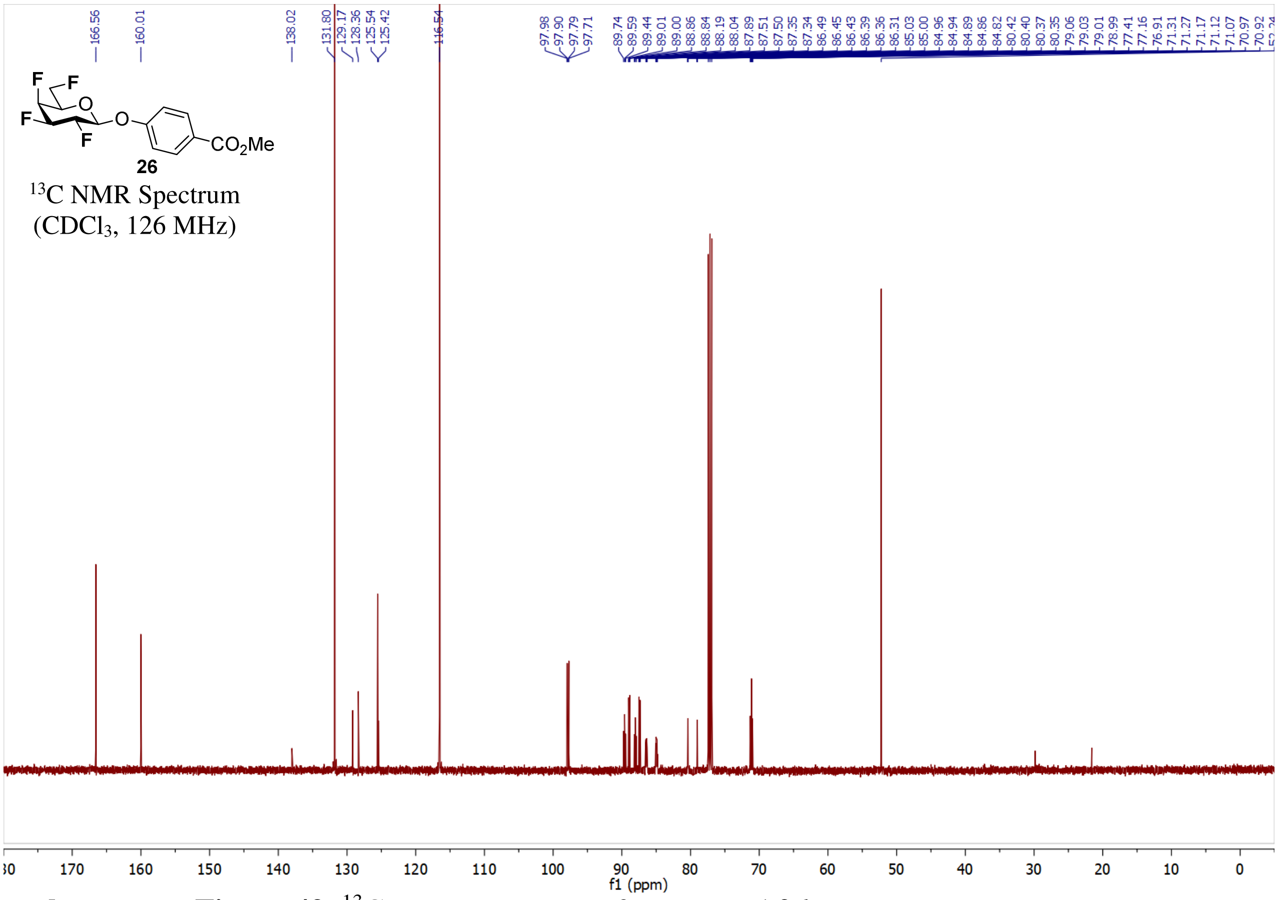

Supplementary Figure 49. ^{13}C NMR spectrum of compound **26**

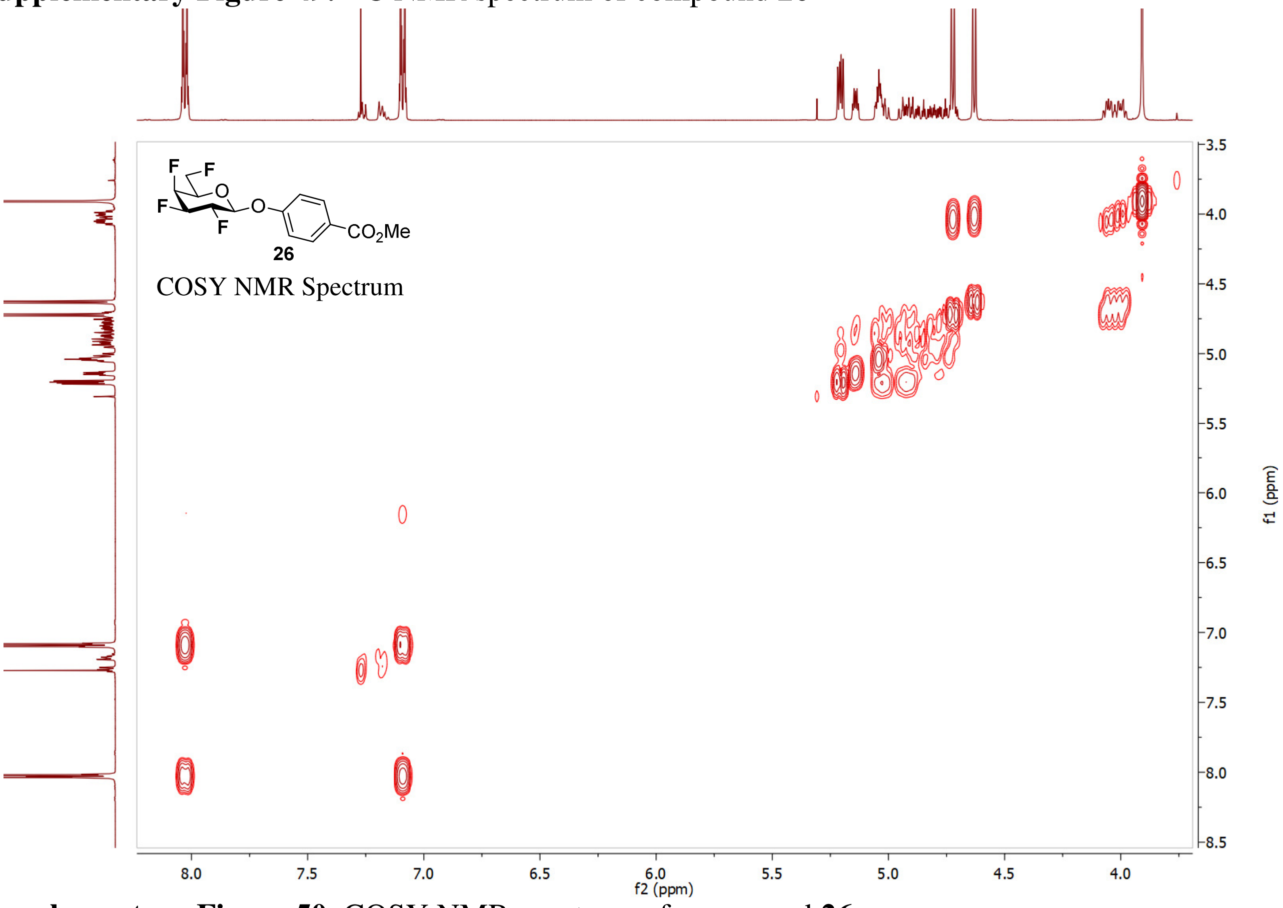

Supplementary Figure 50. COSY NMR spectrum of compound **26**

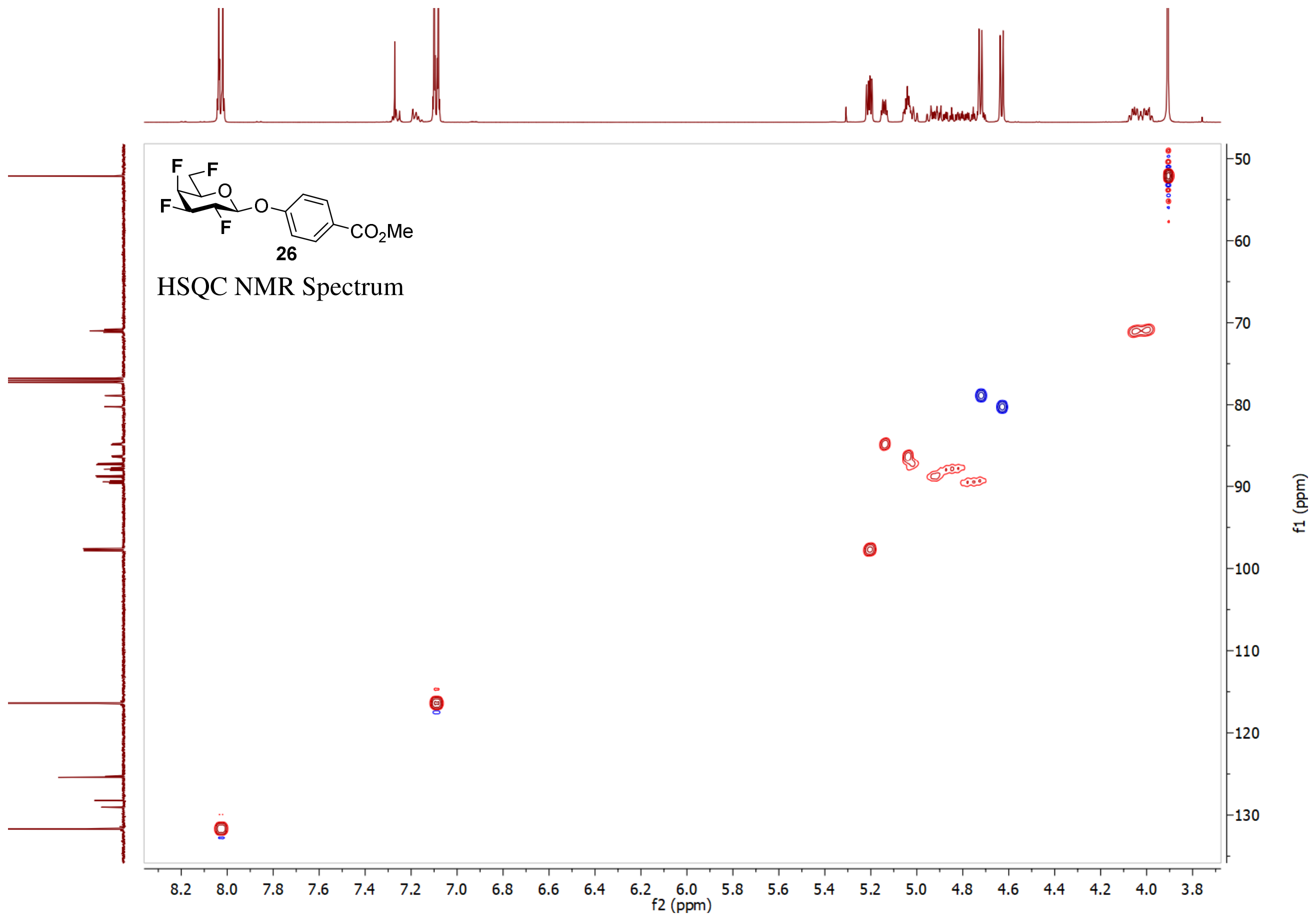

Supplementary Figure 51. HSQC NMR spectrum of compound **26**

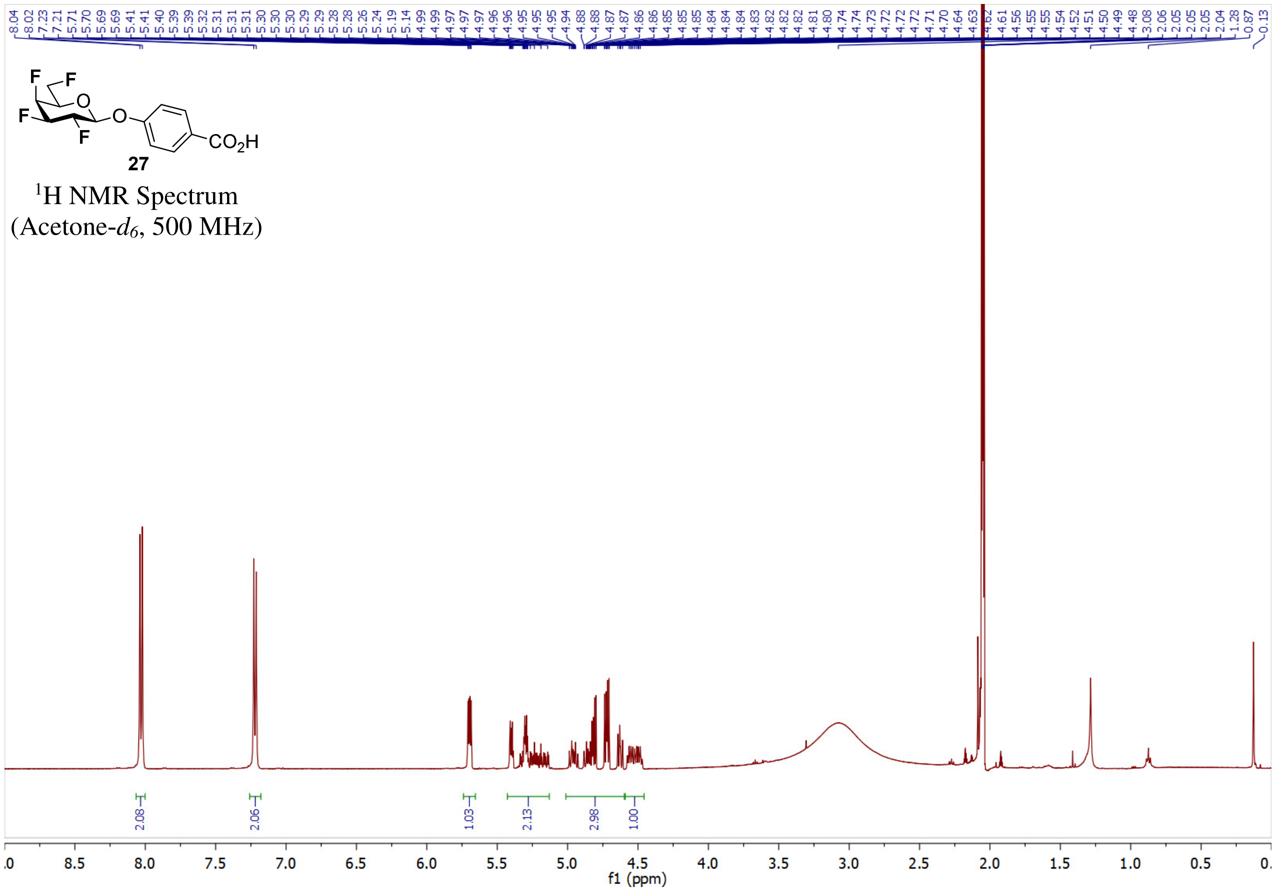

Supplementary Figure 52. ^{1}H NMR spectrum of compound **27**

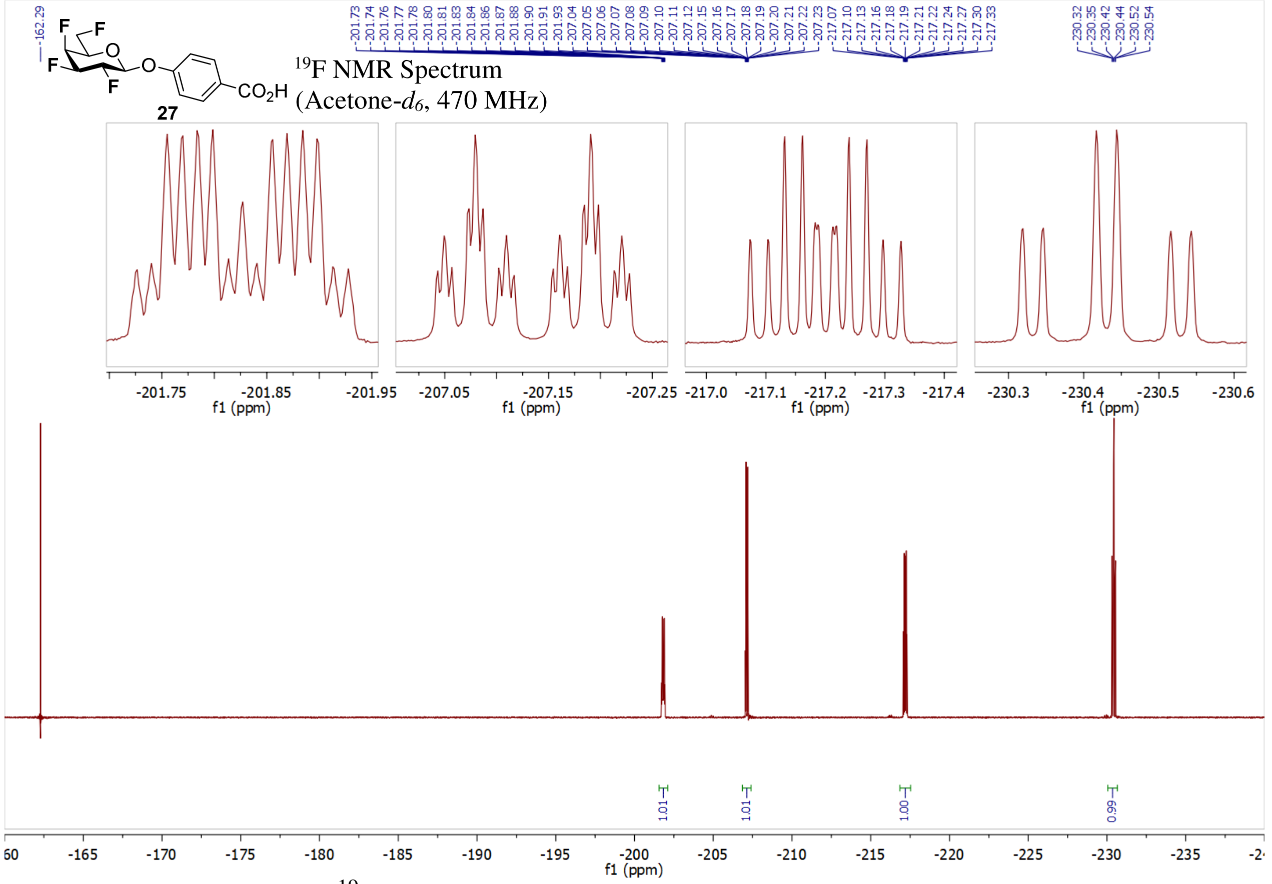

Supplementary Figure 53. ^{19}F NMR spectrum of compound **27**

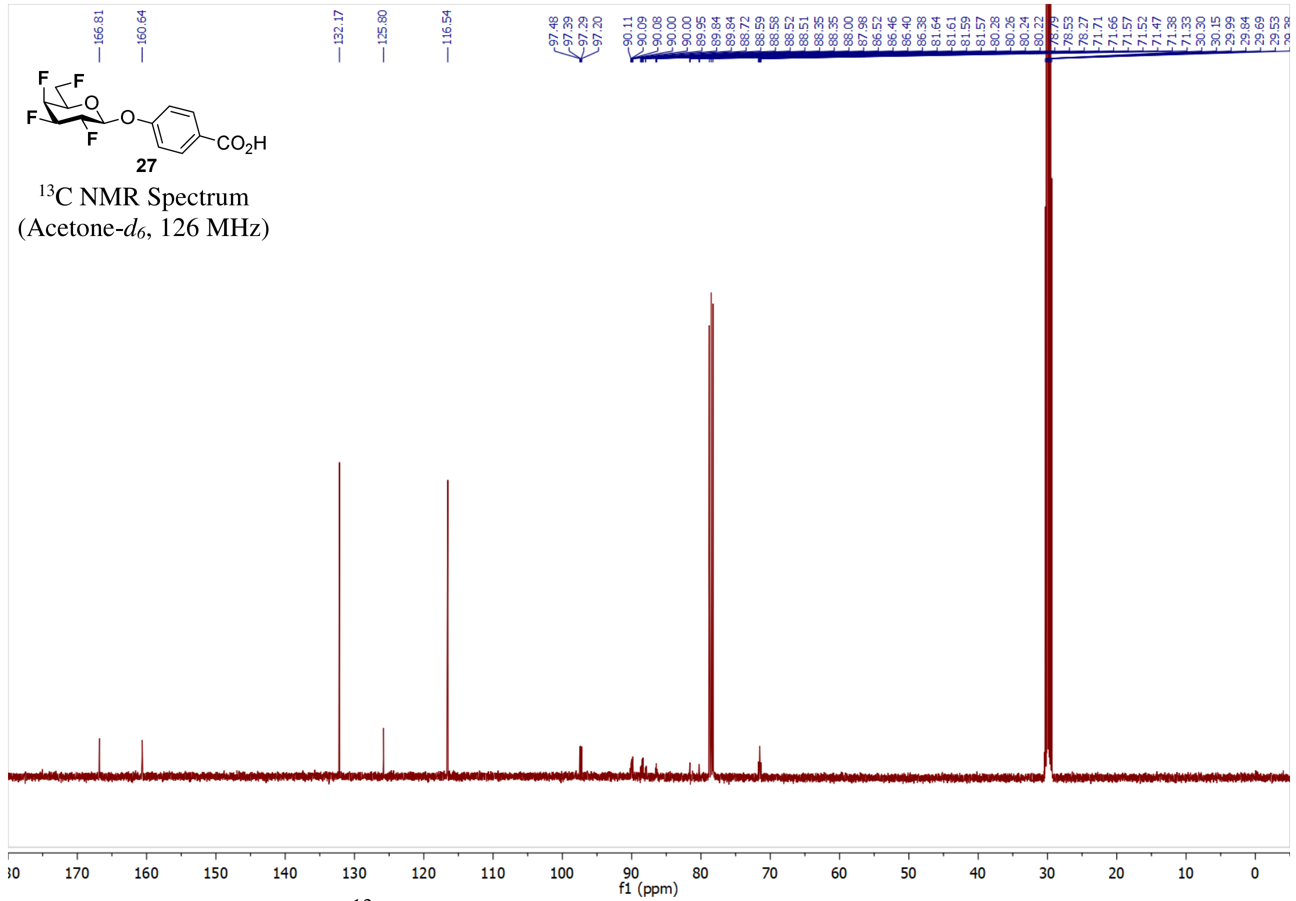

Supplementary Figure 54. ^{13}C NMR spectrum of compound **27**

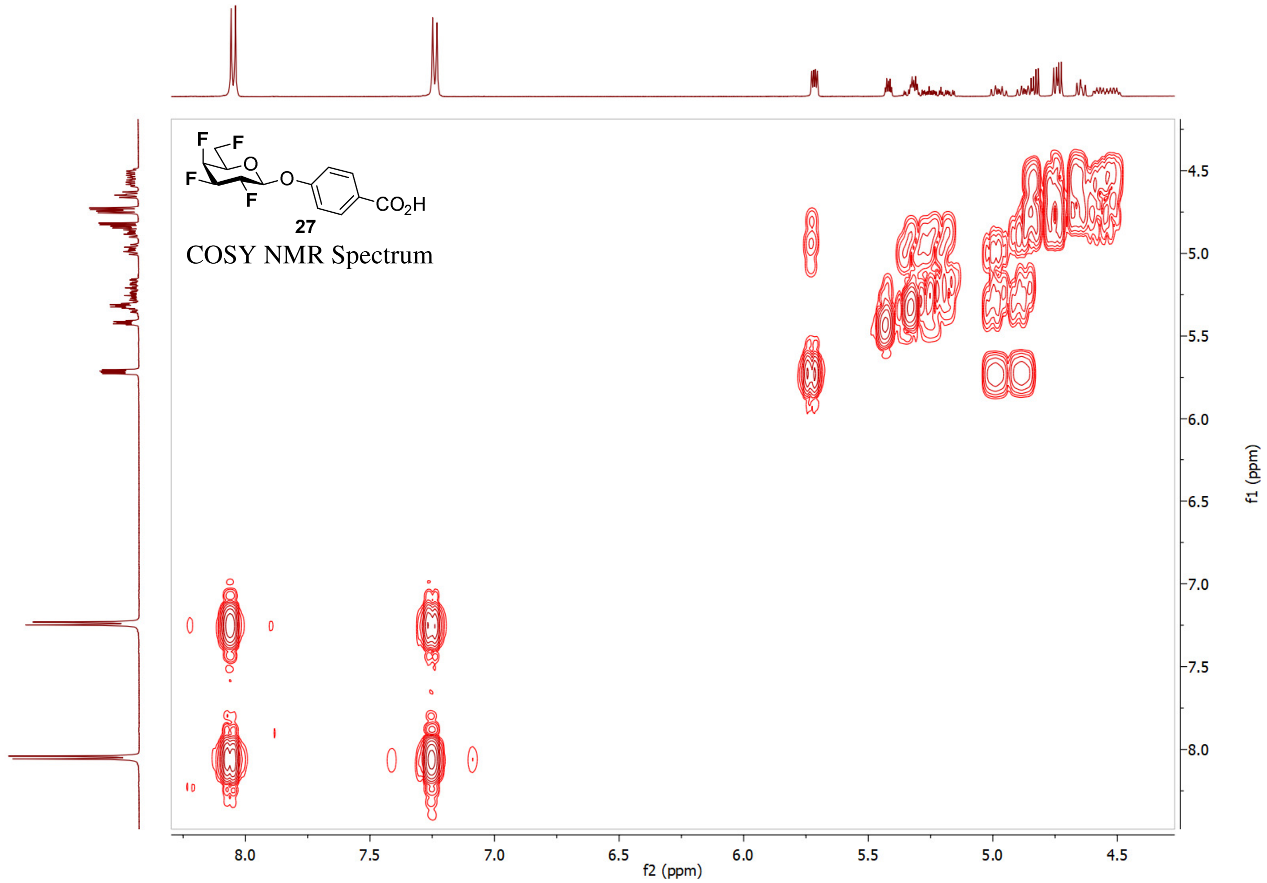

Supplementary Figure 55. COSY NMR spectrum of compound **27**

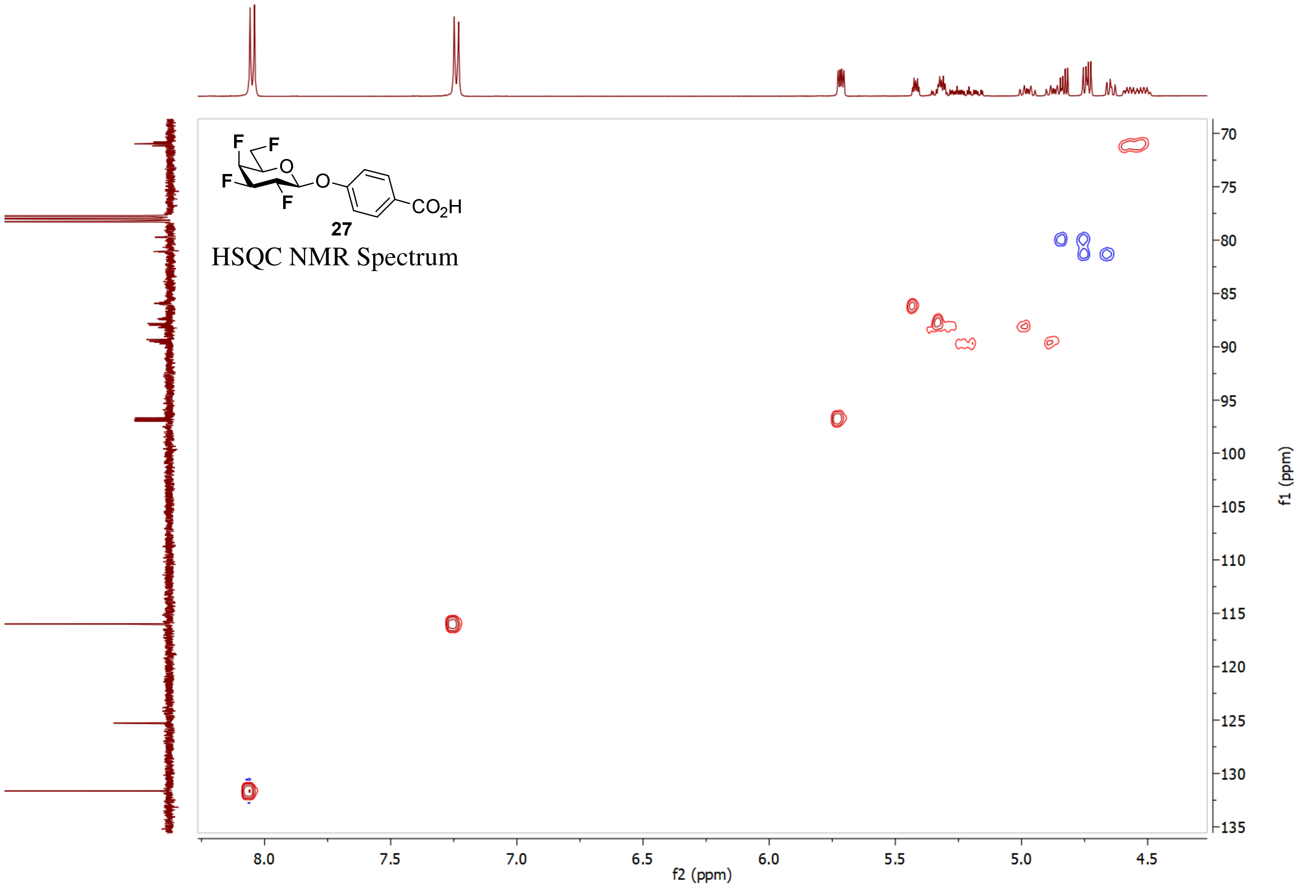

Supplementary Figure 56. HSQC NMR spectrum of compound **27**

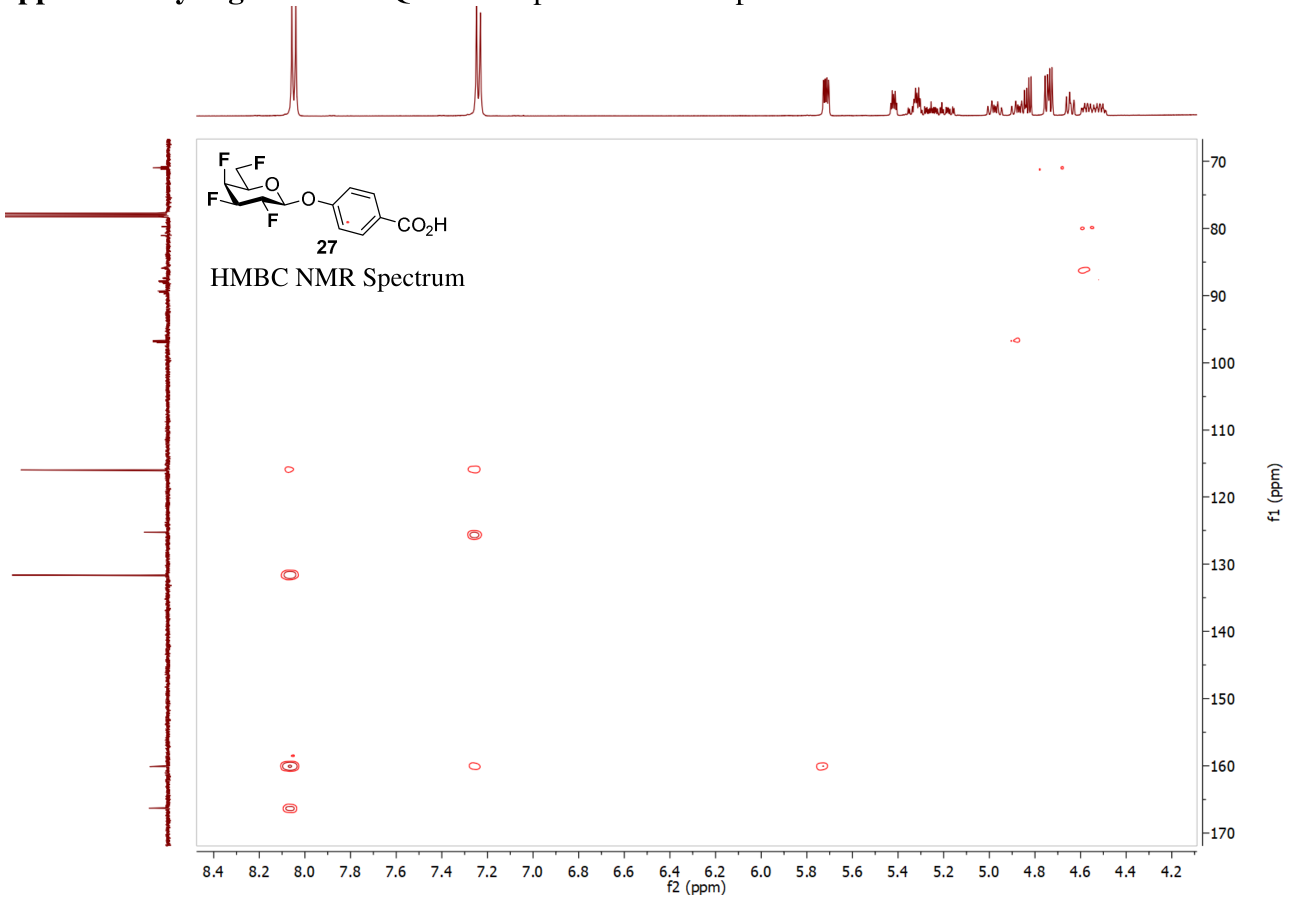

Supplementary Figure 57. HMBC NMR spectrum of compound **27**

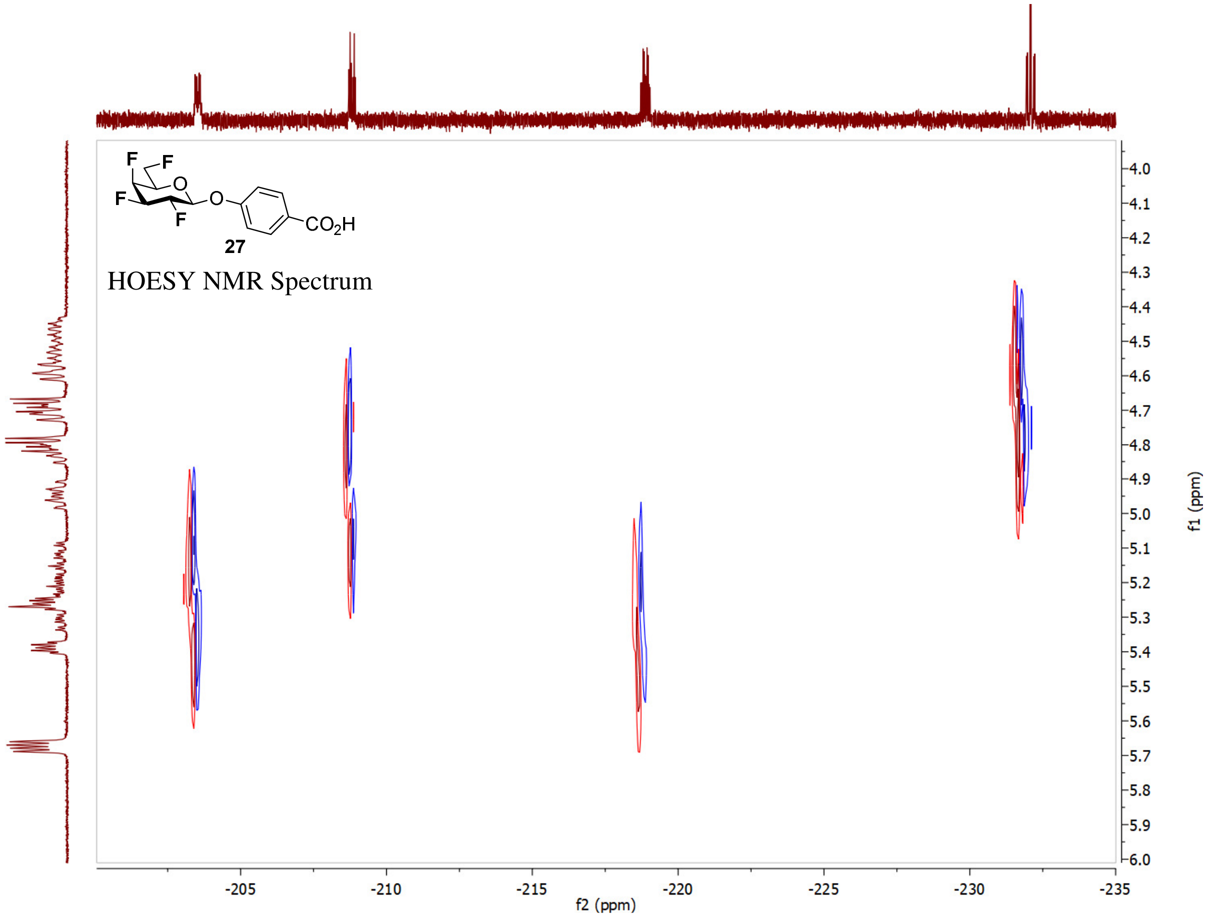

Supplementary Figure 58. HOESY NMR spectrum of compound **27**

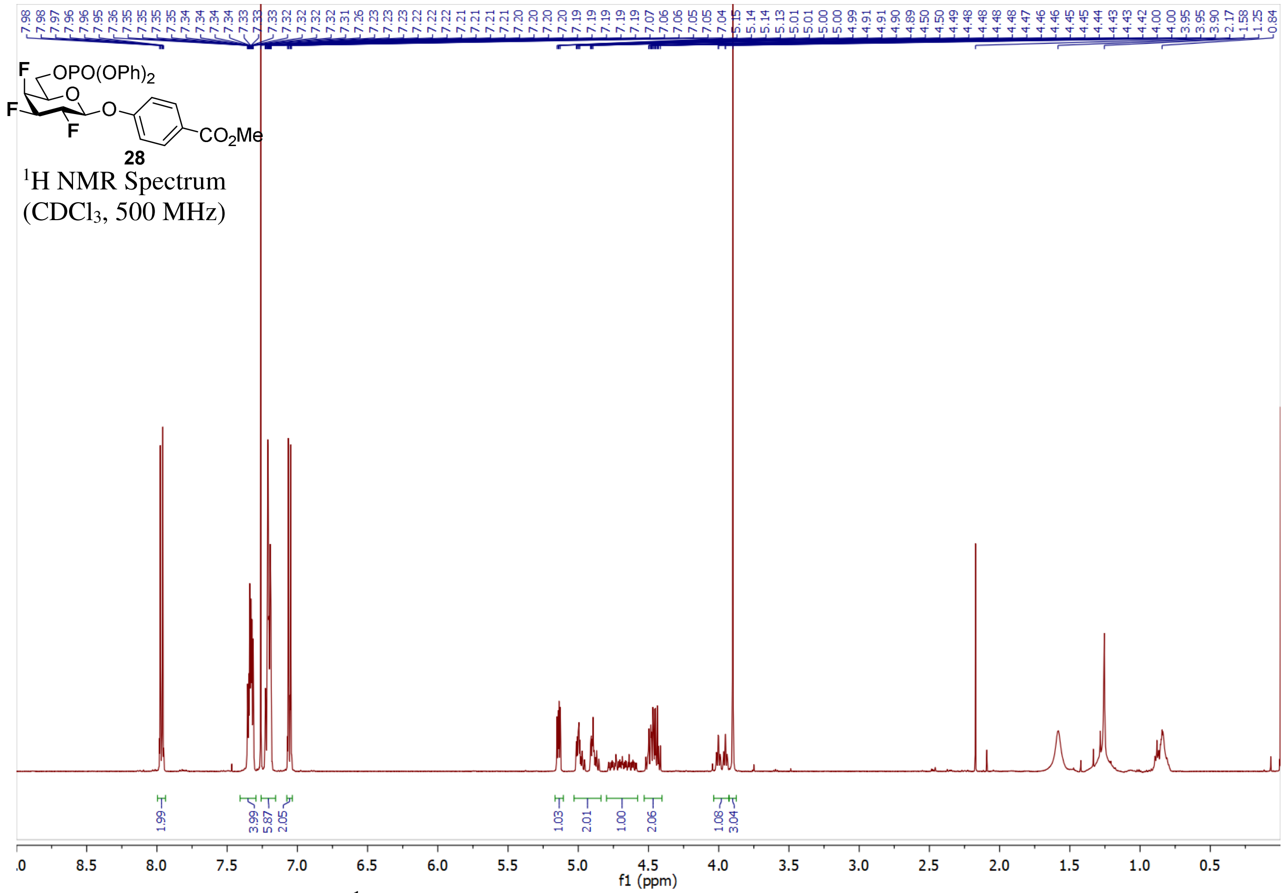

Supplementary Figure 59. ^{1}H NMR spectrum of compound **28**

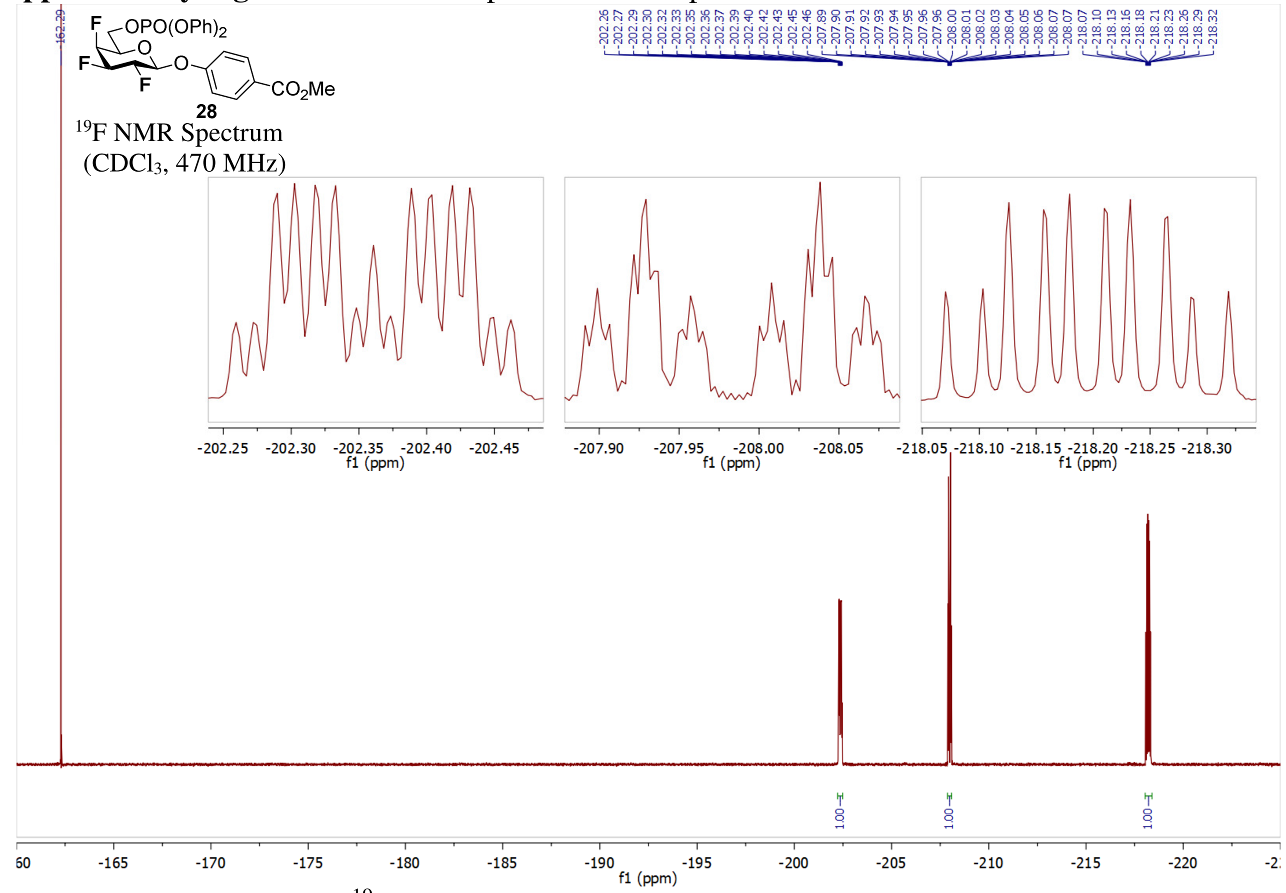

Supplementary Figure 60. ^{19}F NMR spectrum of compound **28**

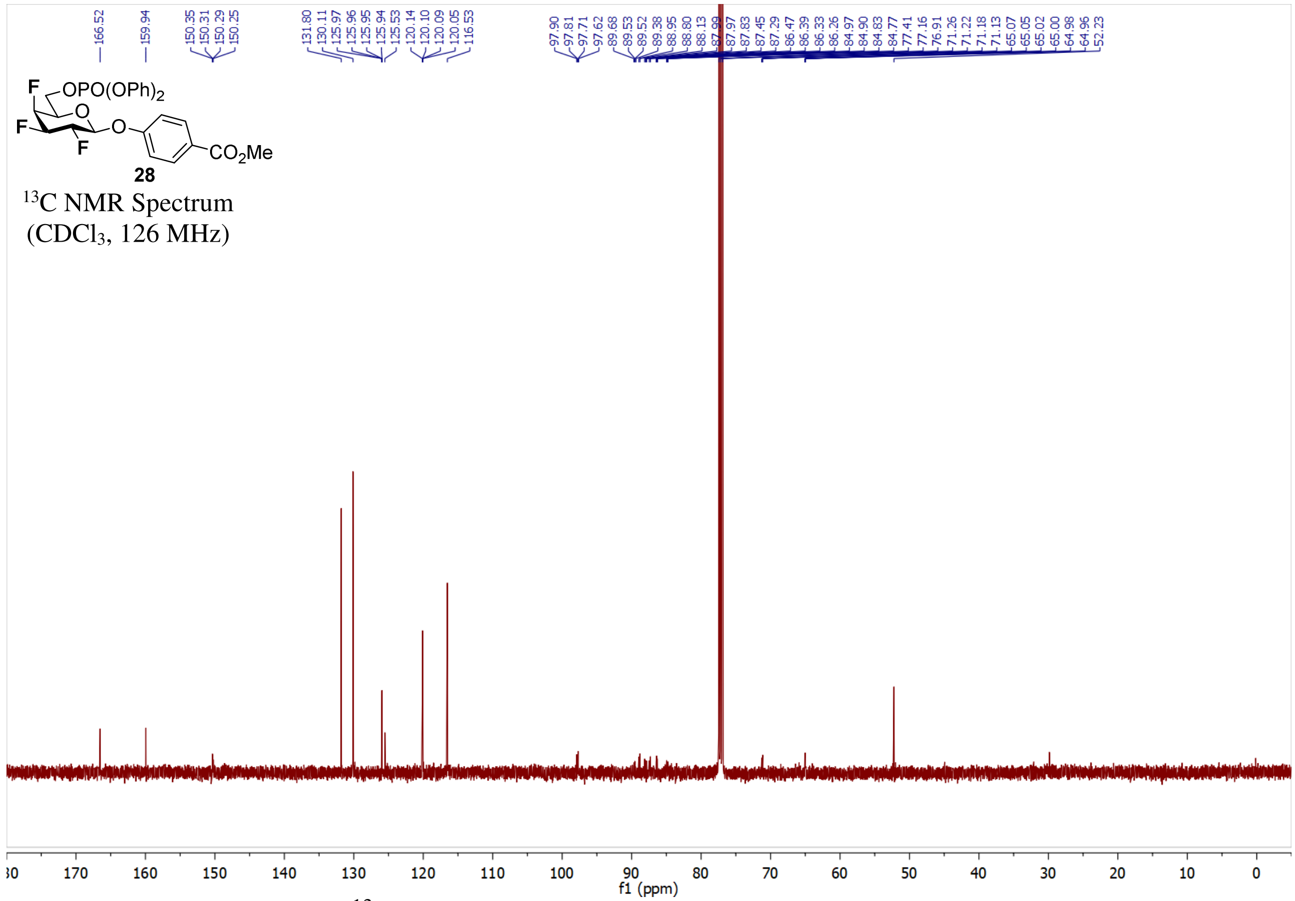

Supplementary Figure 61. ^{13}C NMR spectrum of compound **28**

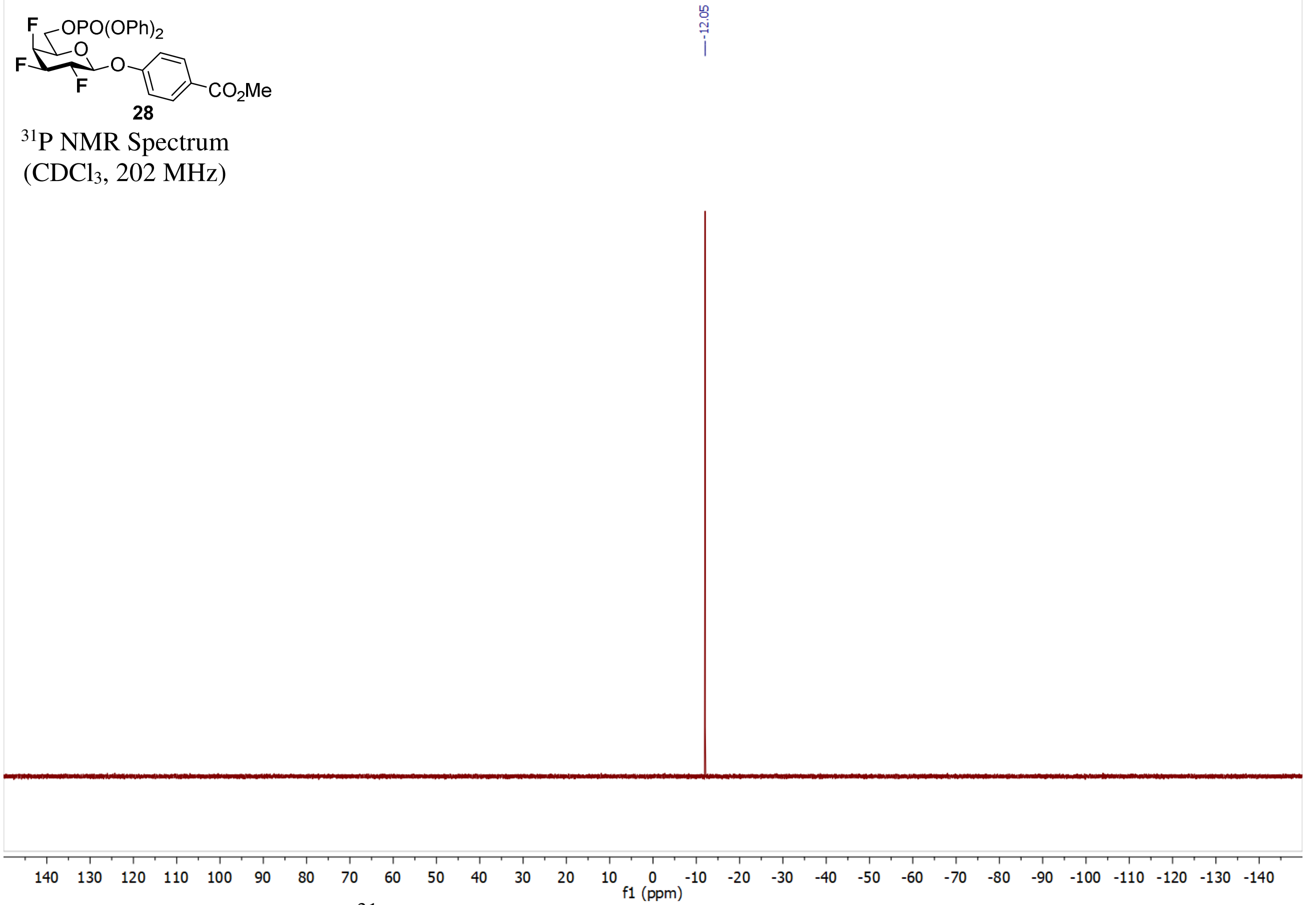

Supplementary Figure 62. ^{31}P NMR spectrum of compound **28**

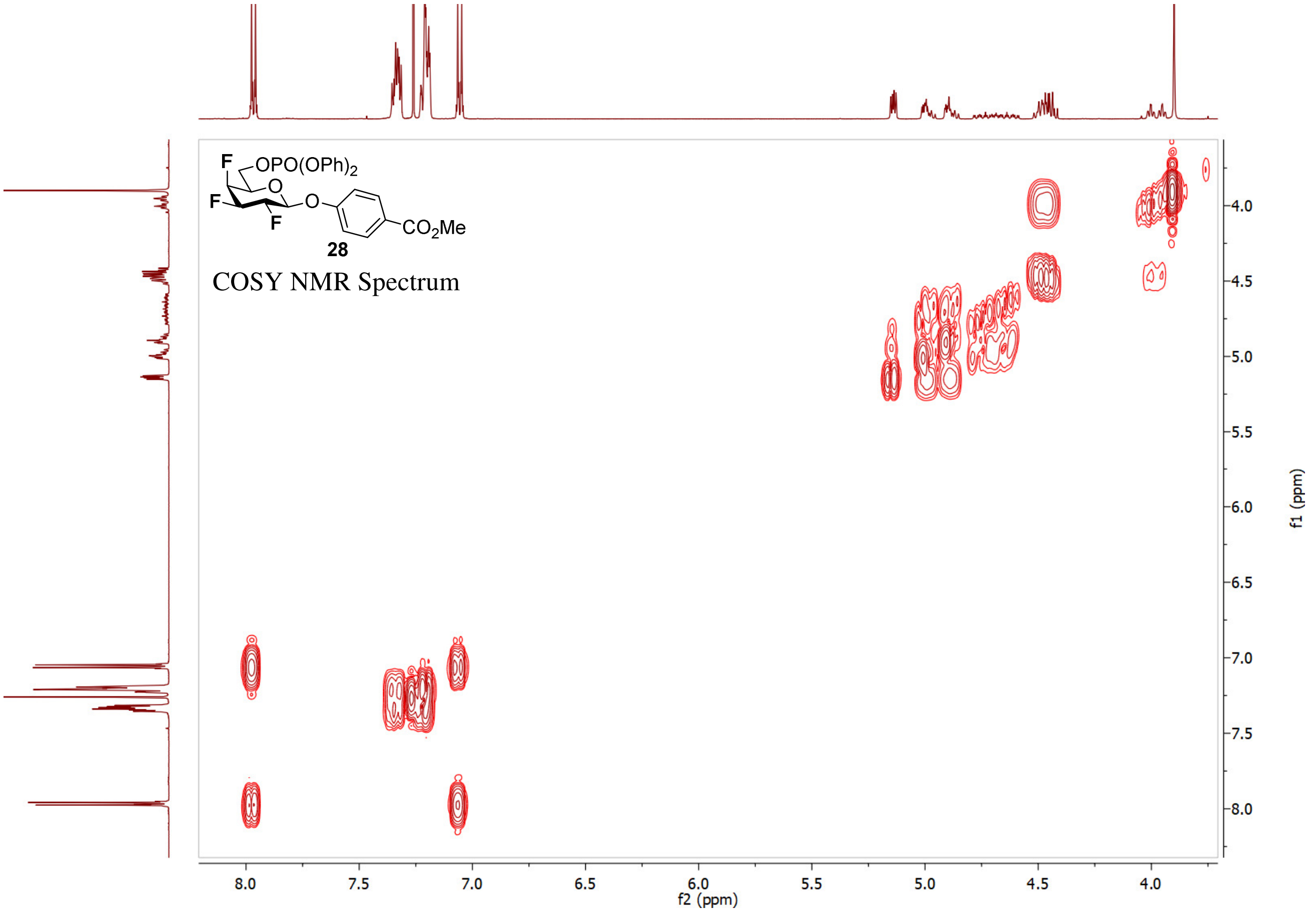

Supplementary Figure 63. COSY NMR spectrum of compound **28**

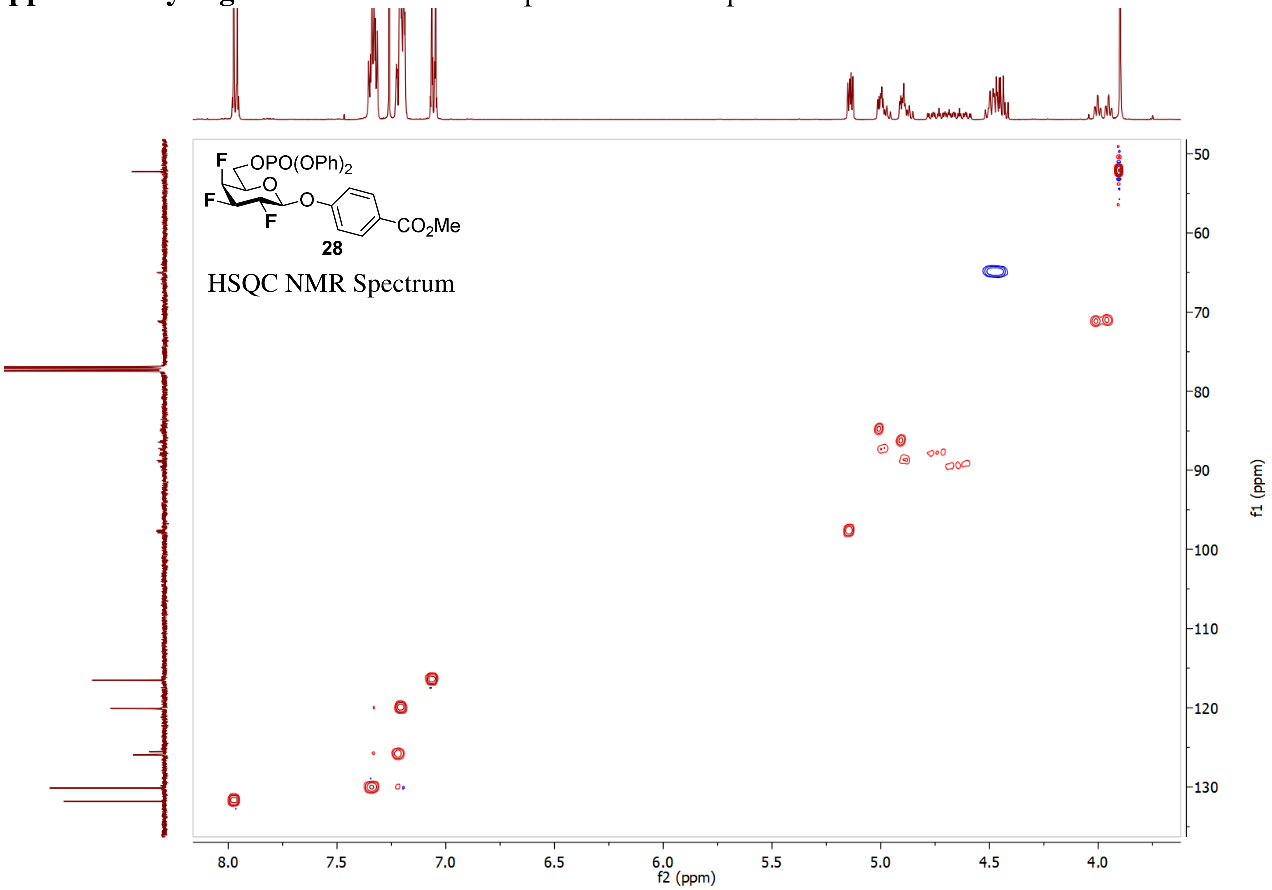

Supplementary Figure 64. HSQC NMR spectrum of compound **28**

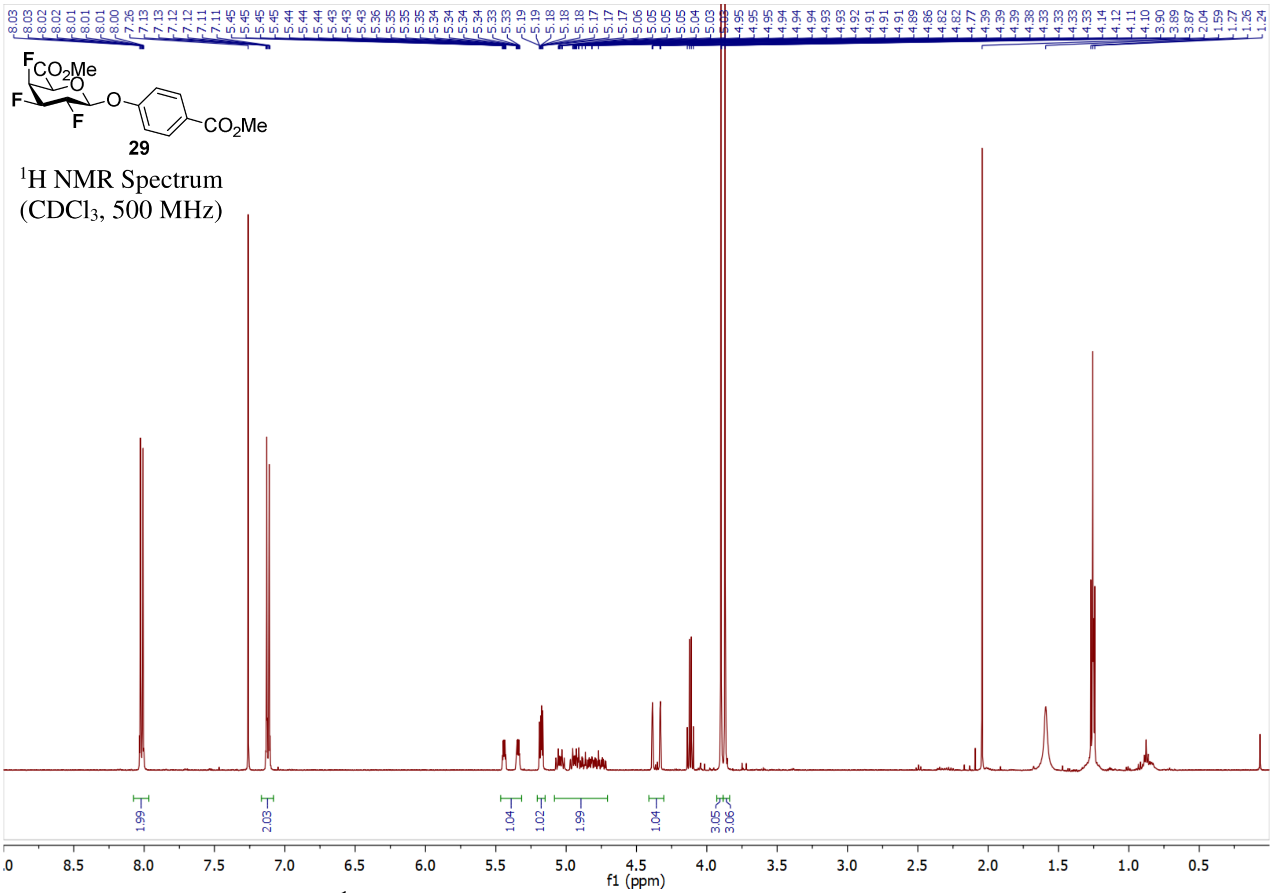

Supplementary Figure 65. ^{1}H NMR spectrum of compound **29**

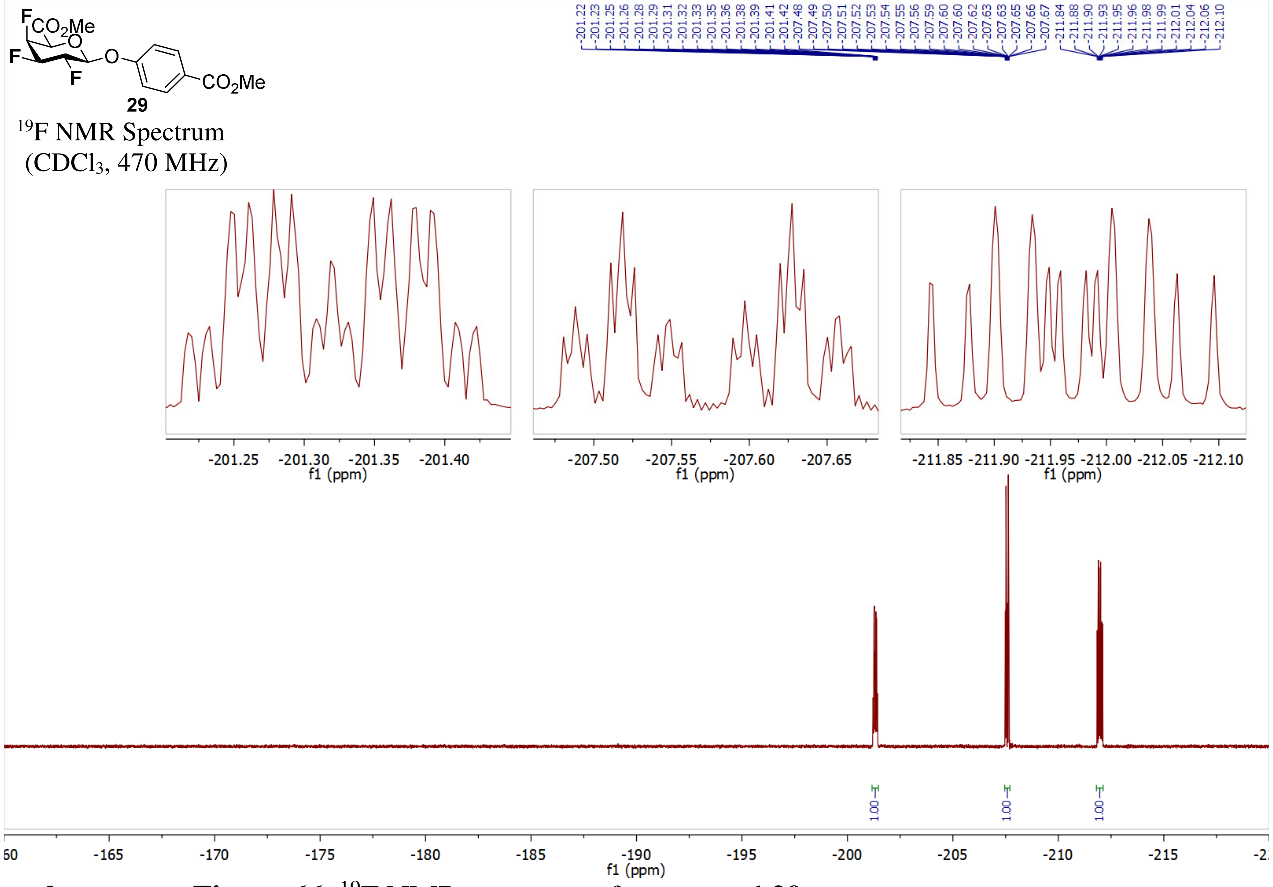

Supplementary Figure 66. ^{19}F NMR spectrum of compound **29**

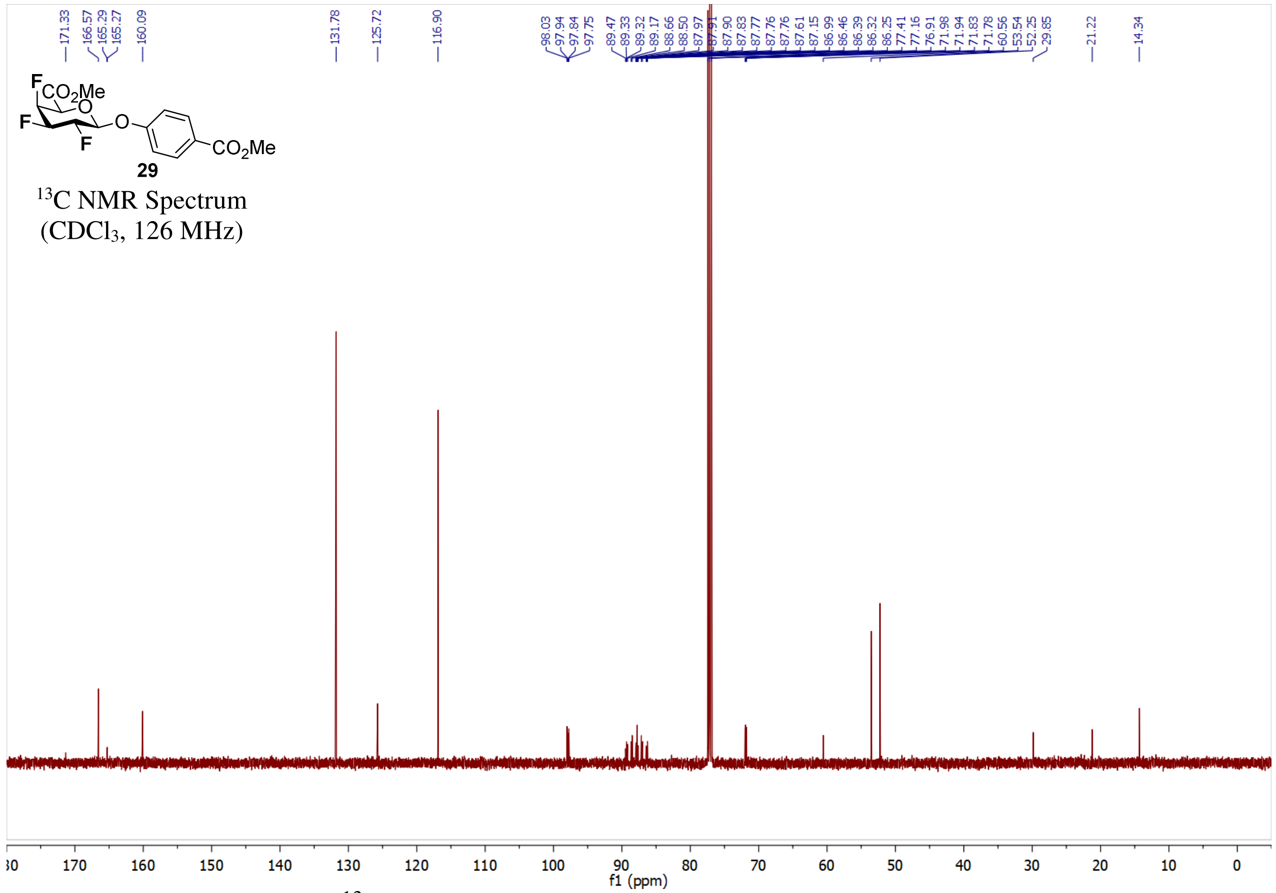

Supplementary Figure 67. ^{13}C NMR spectrum of compound **29**

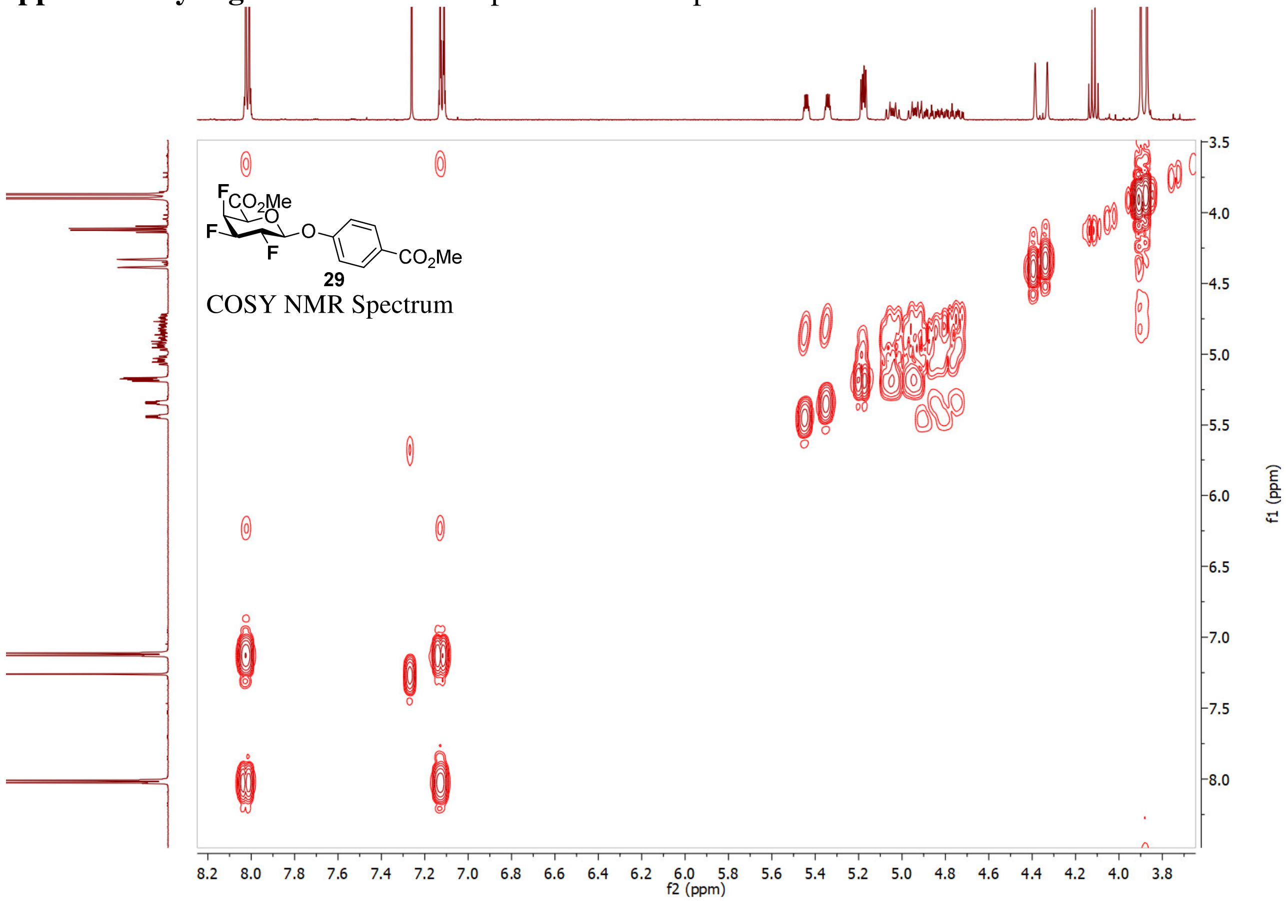

Supplementary Figure 68. COSY NMR spectrum of compound **29**

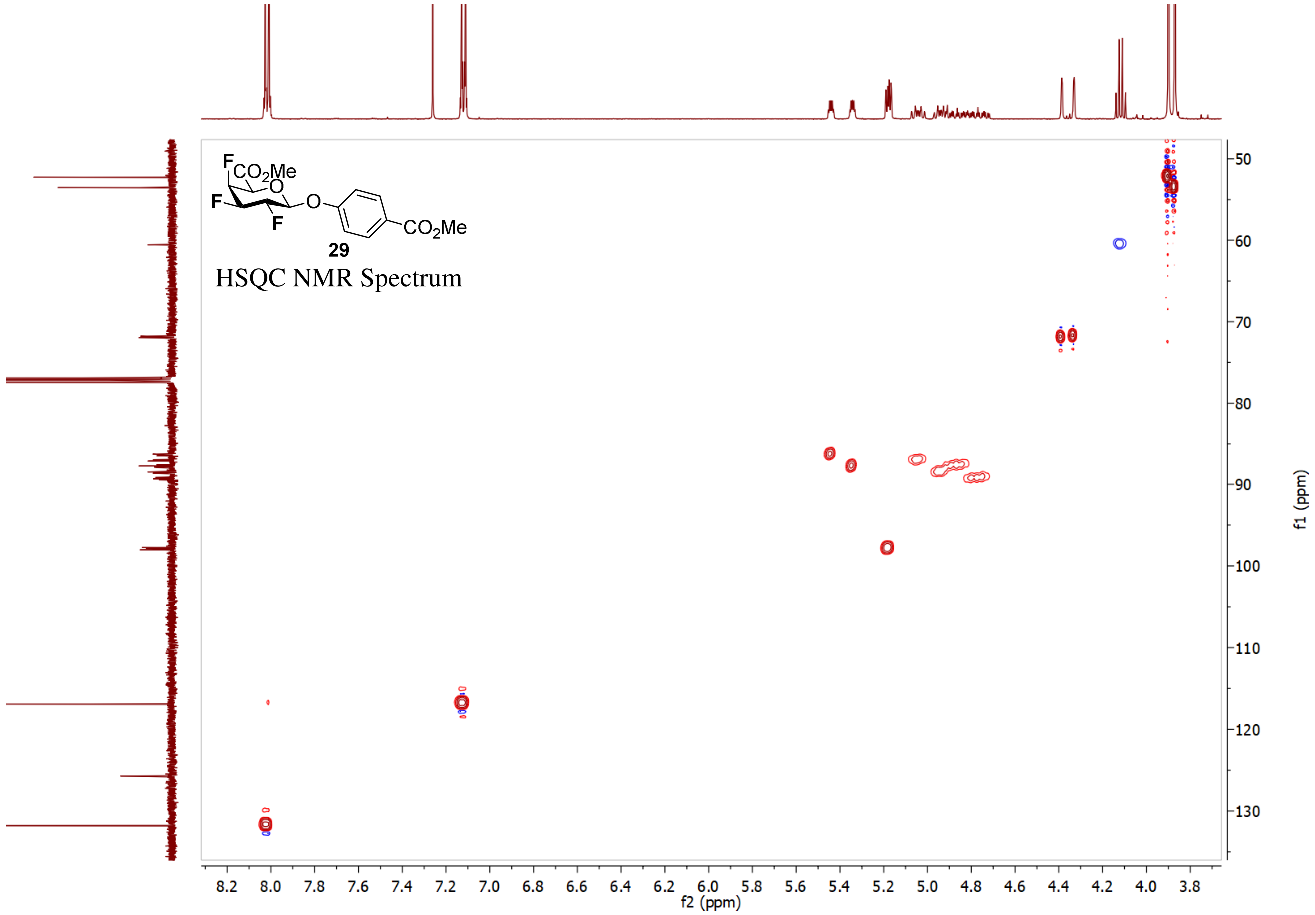

Supplementary Figure 69. HSQC NMR spectrum of compound **29**

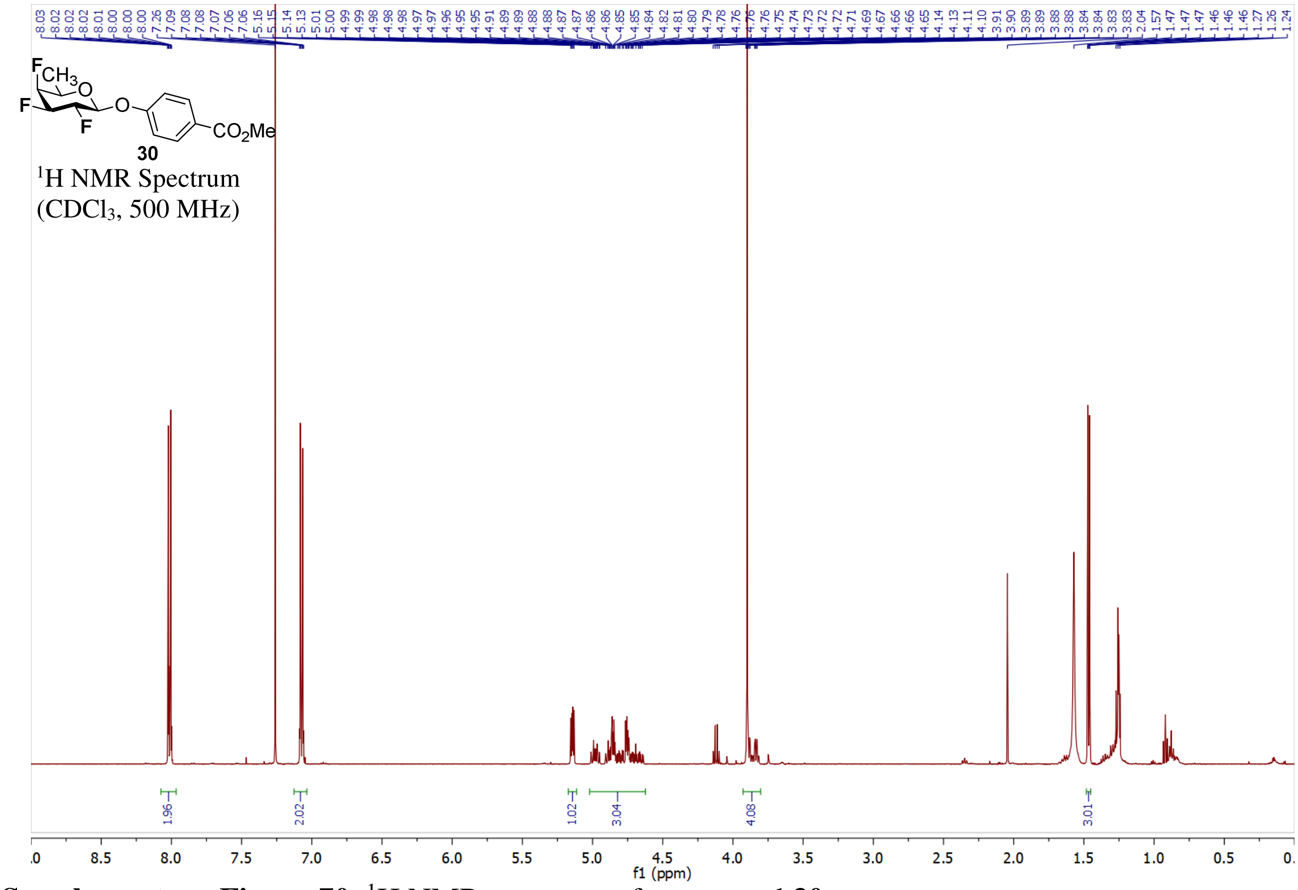

Supplementary Figure 70. ^{1}H NMR spectrum of compound **30**

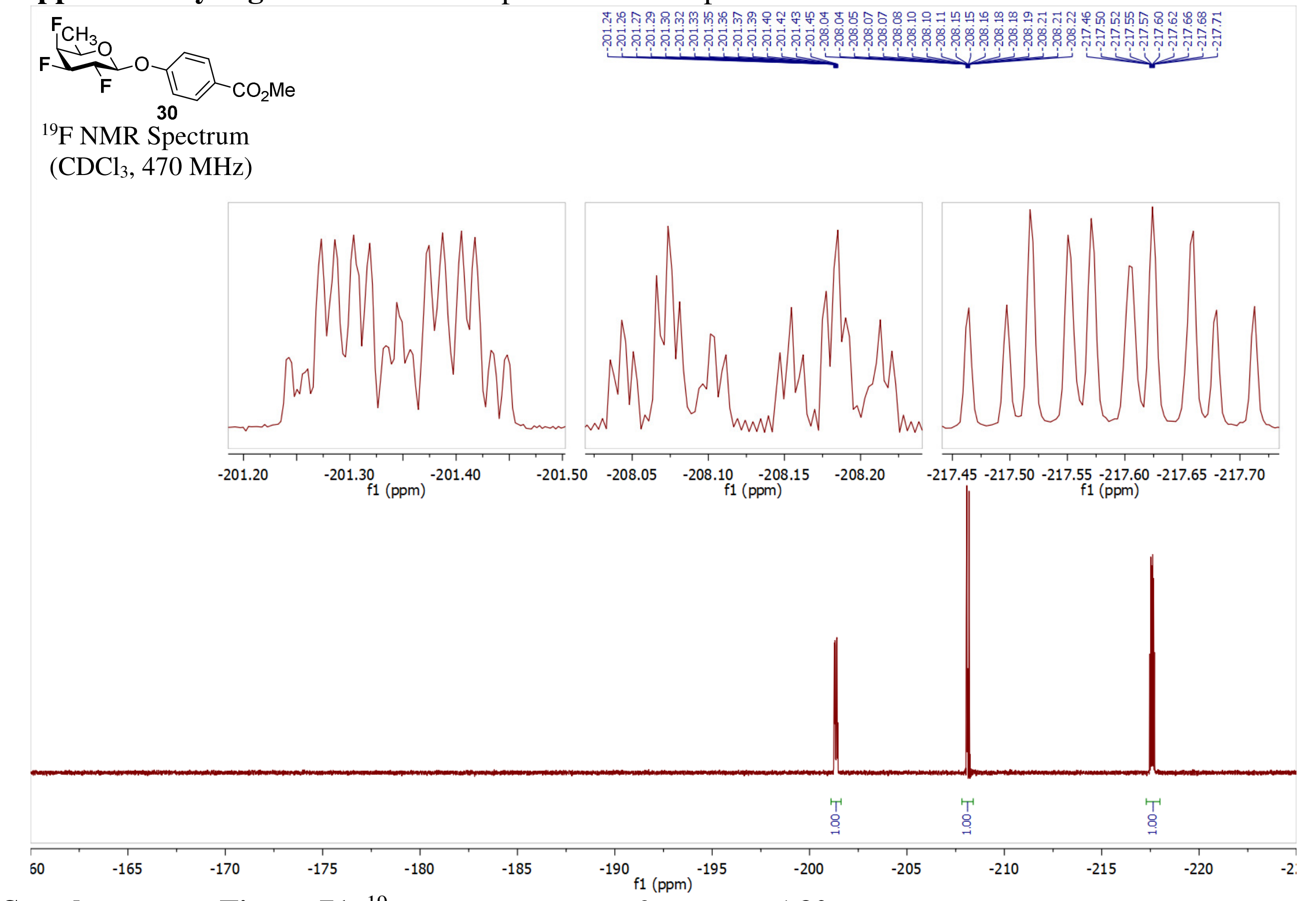

Supplementary Figure 71. ^{19}F NMR spectrum of compound **30**

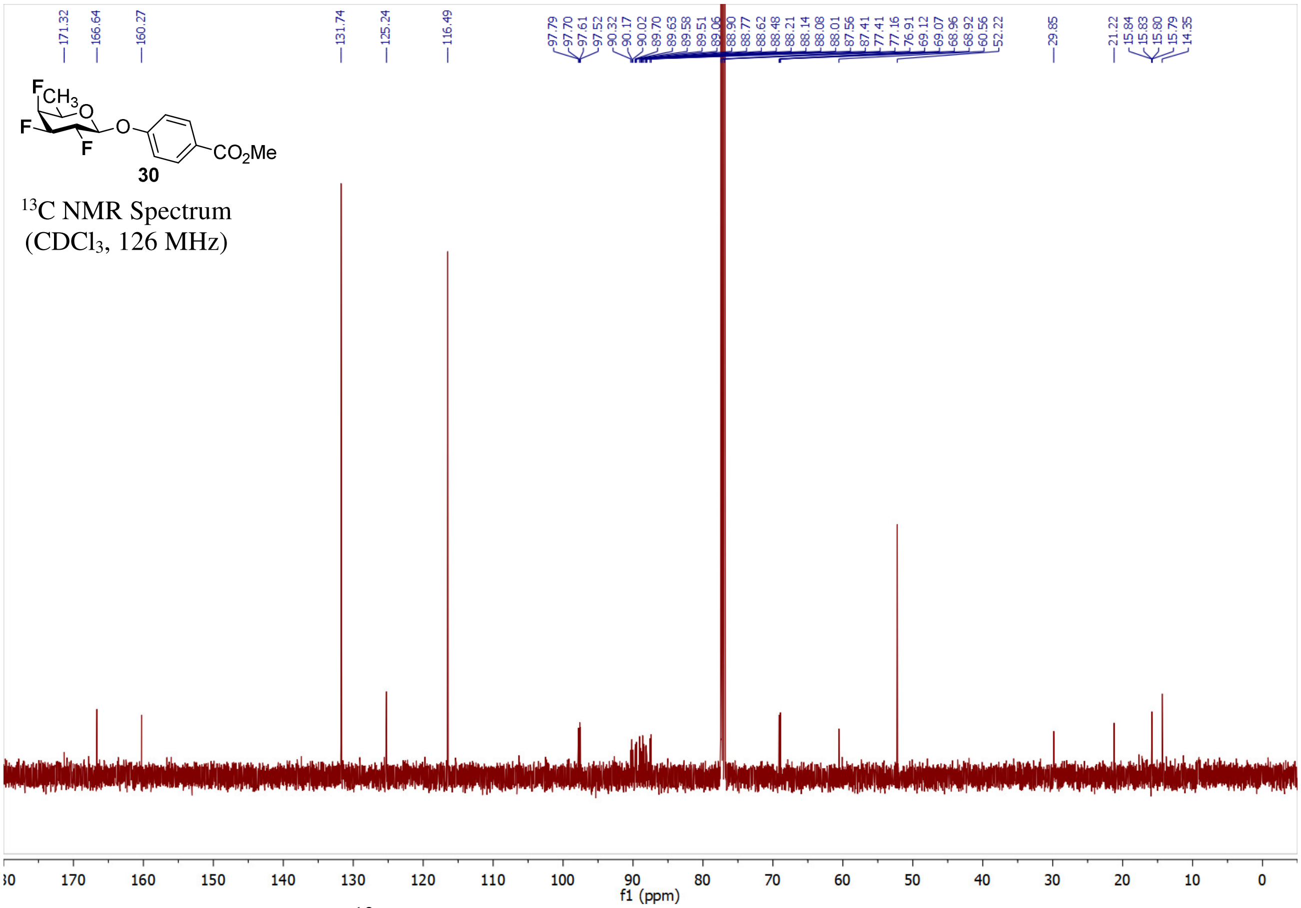

Supplementary Figure 72. ^{13}C NMR spectrum of compound **30**

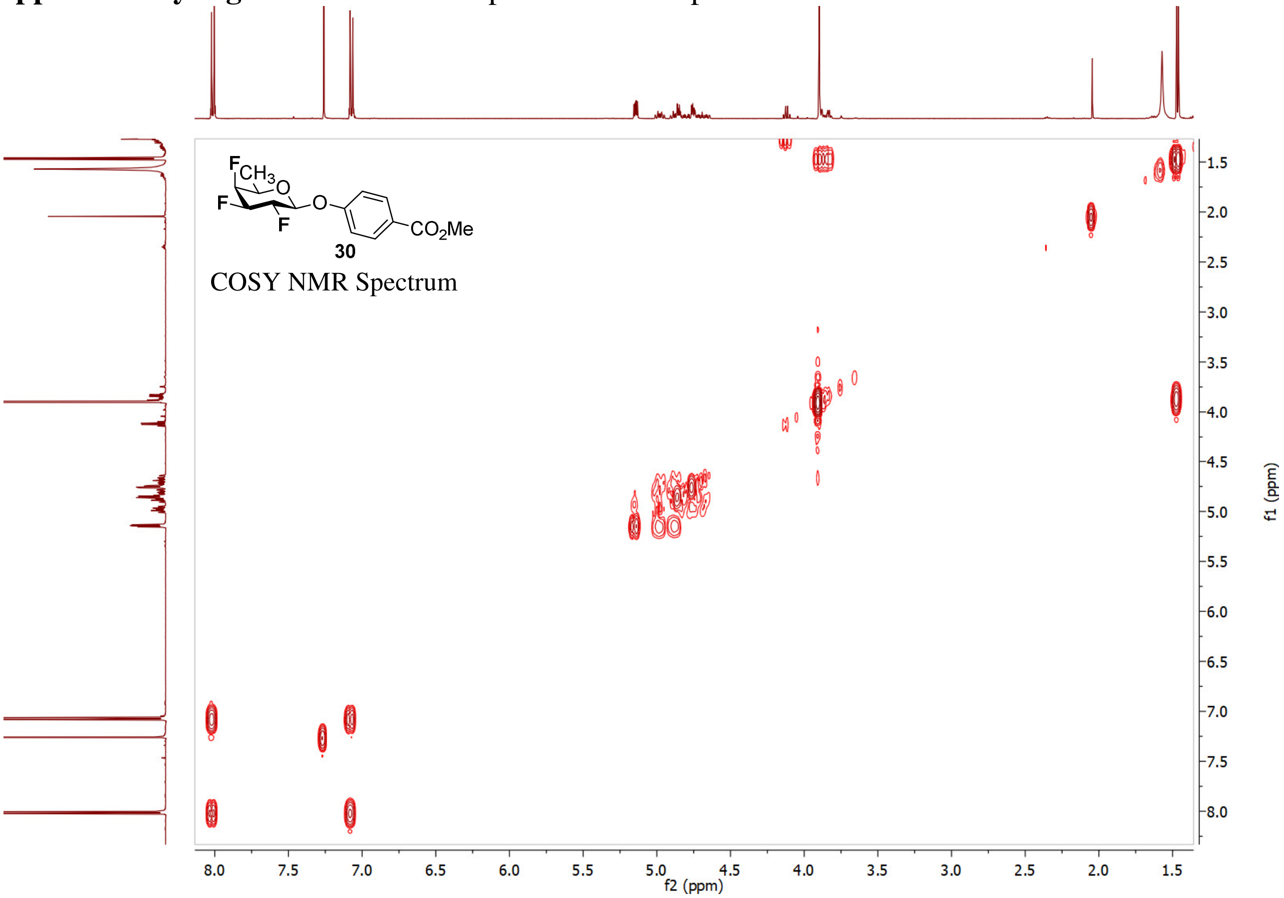

Supplementary Figure 73. COSY NMR spectrum of compound **30**

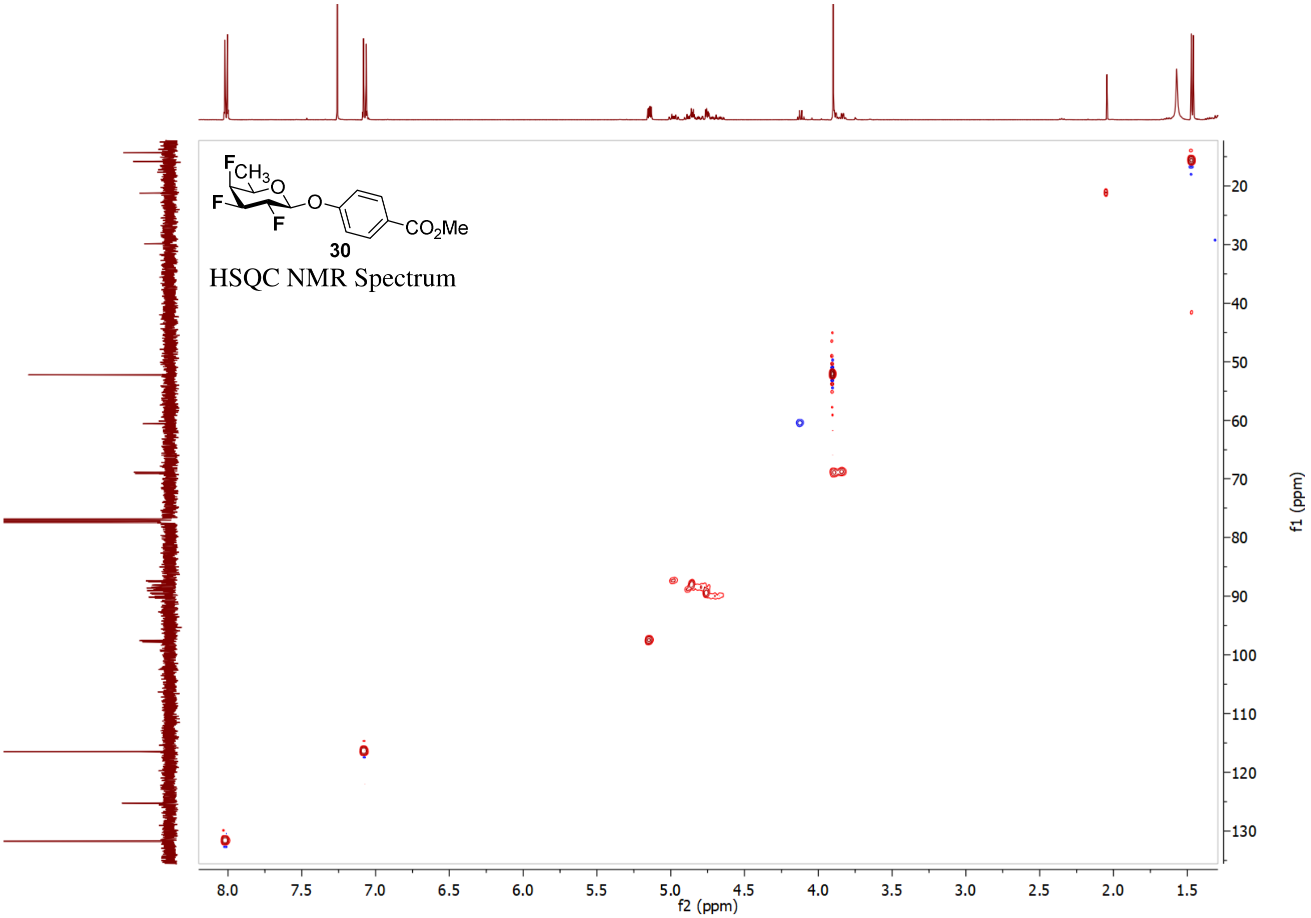

Supplementary Figure 74. HSQC NMR spectrum of compound **30**

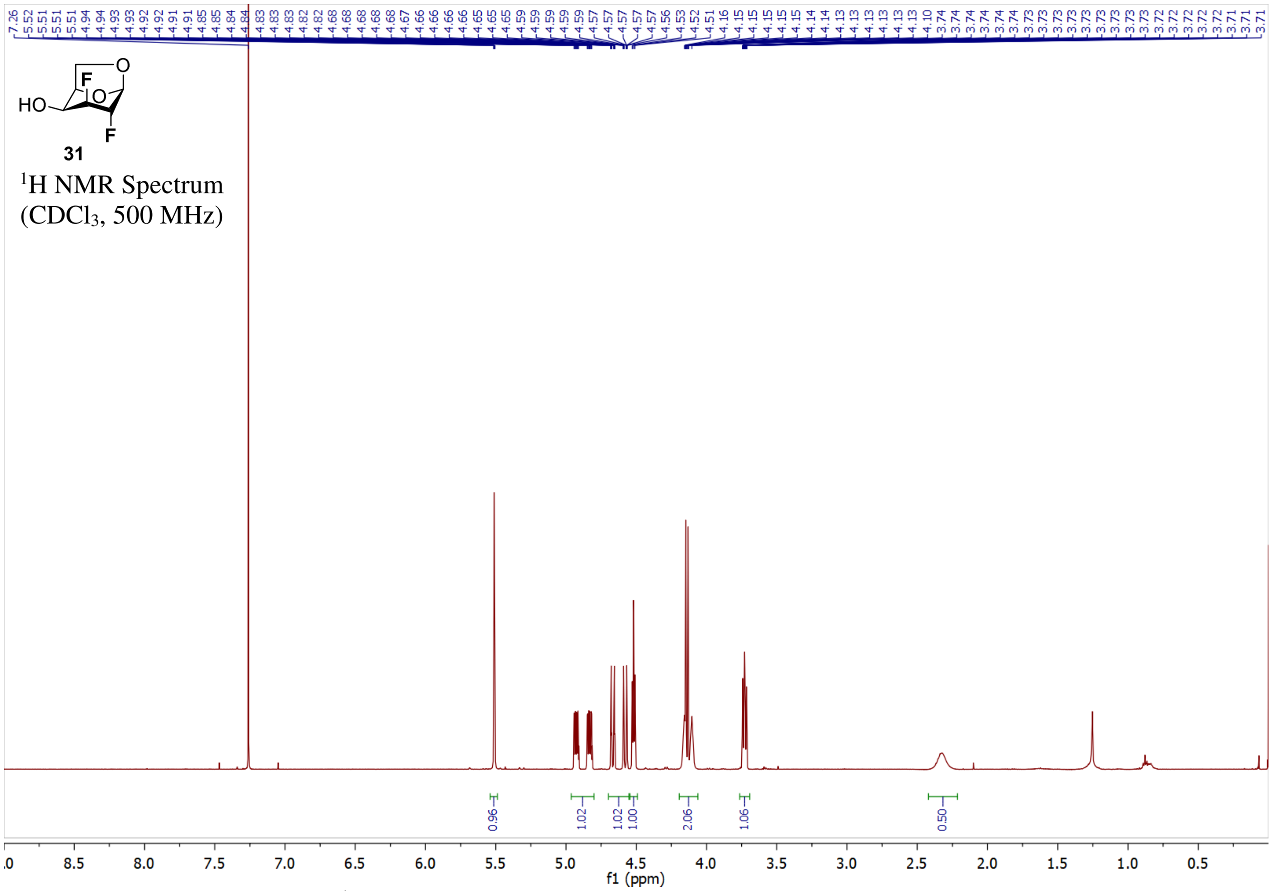

Supplementary Figure 75. ^{1}H NMR spectrum of compound **31**

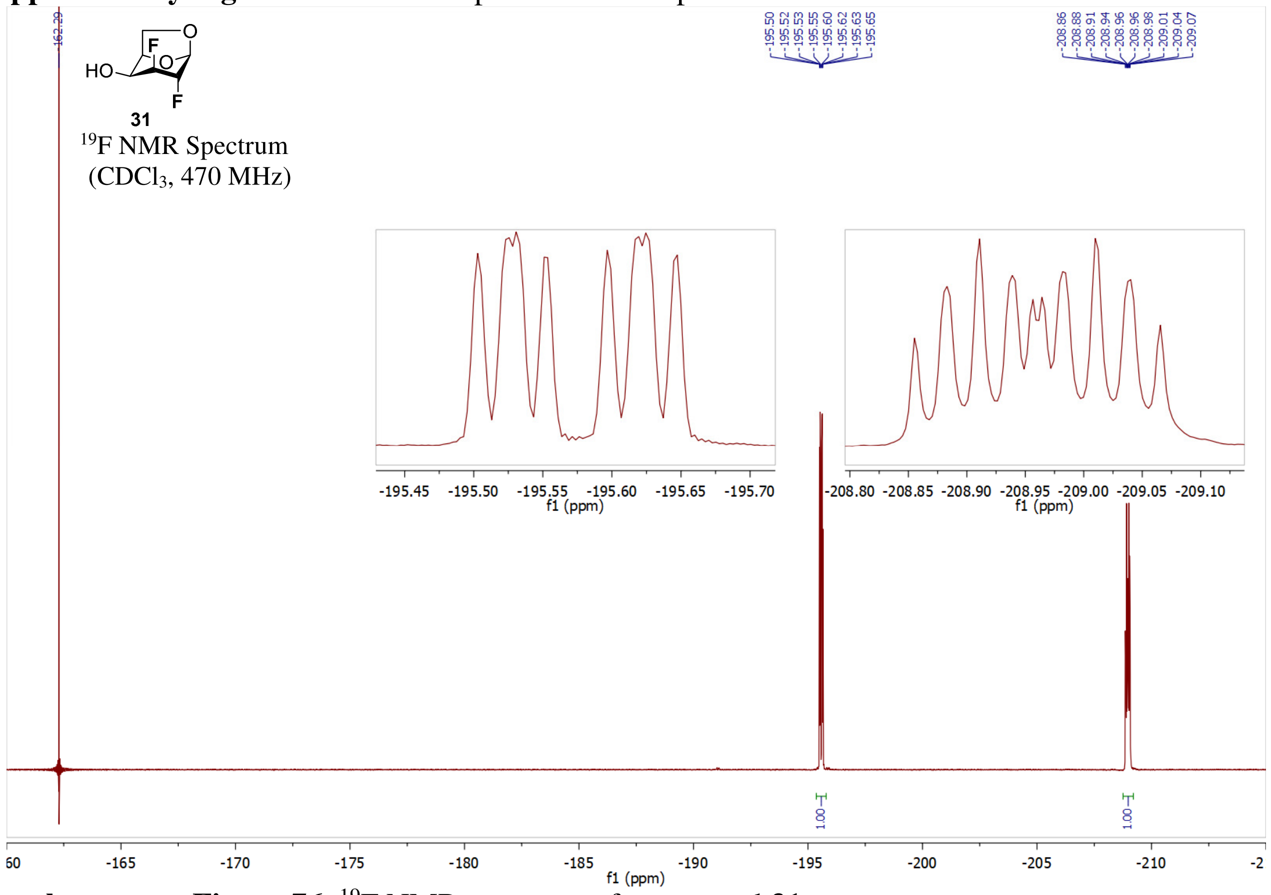

Supplementary Figure 76. ^{19}F NMR spectrum of compound **31**

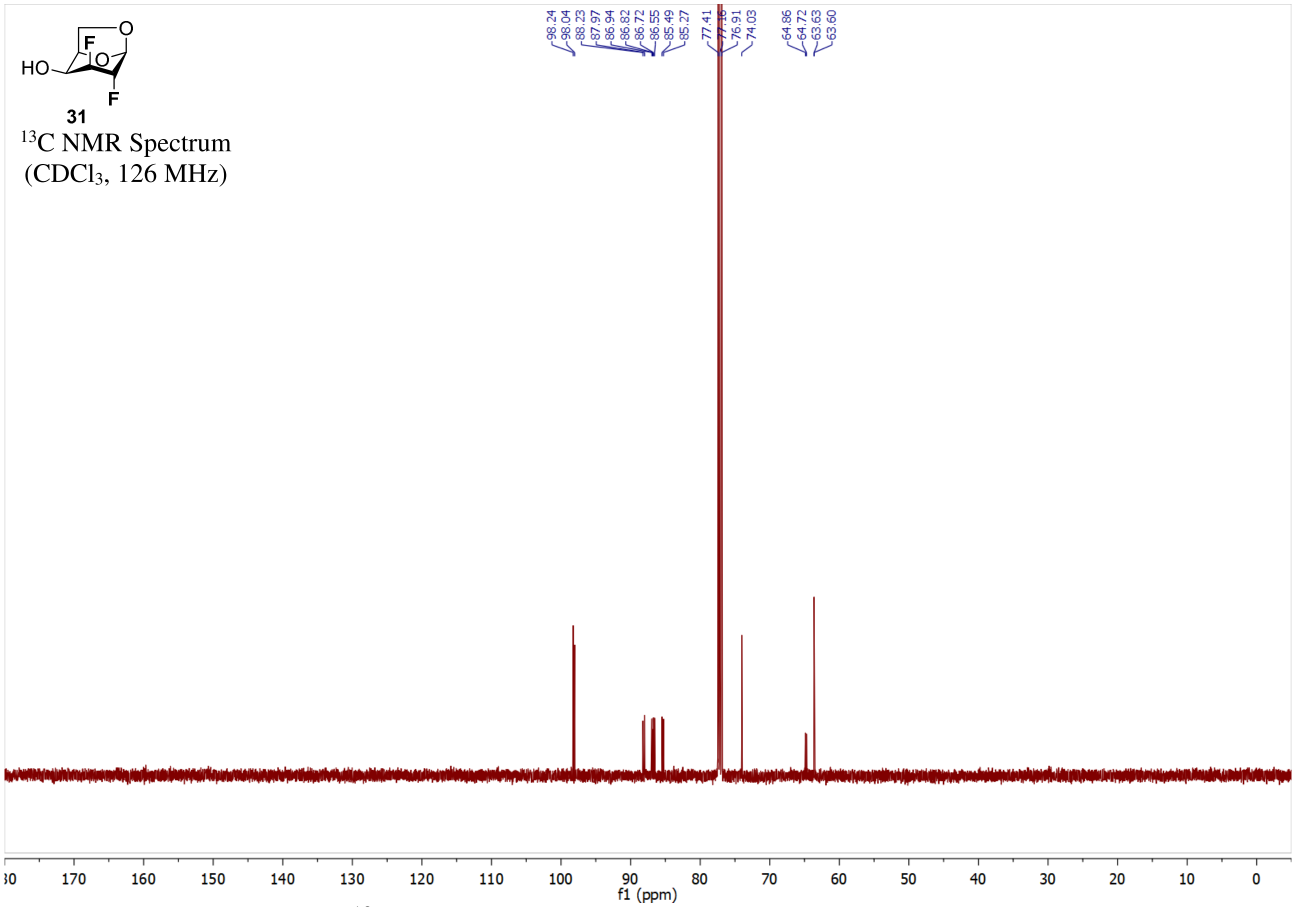

Supplementary Figure 77. ^{13}C NMR spectrum of compound **31**

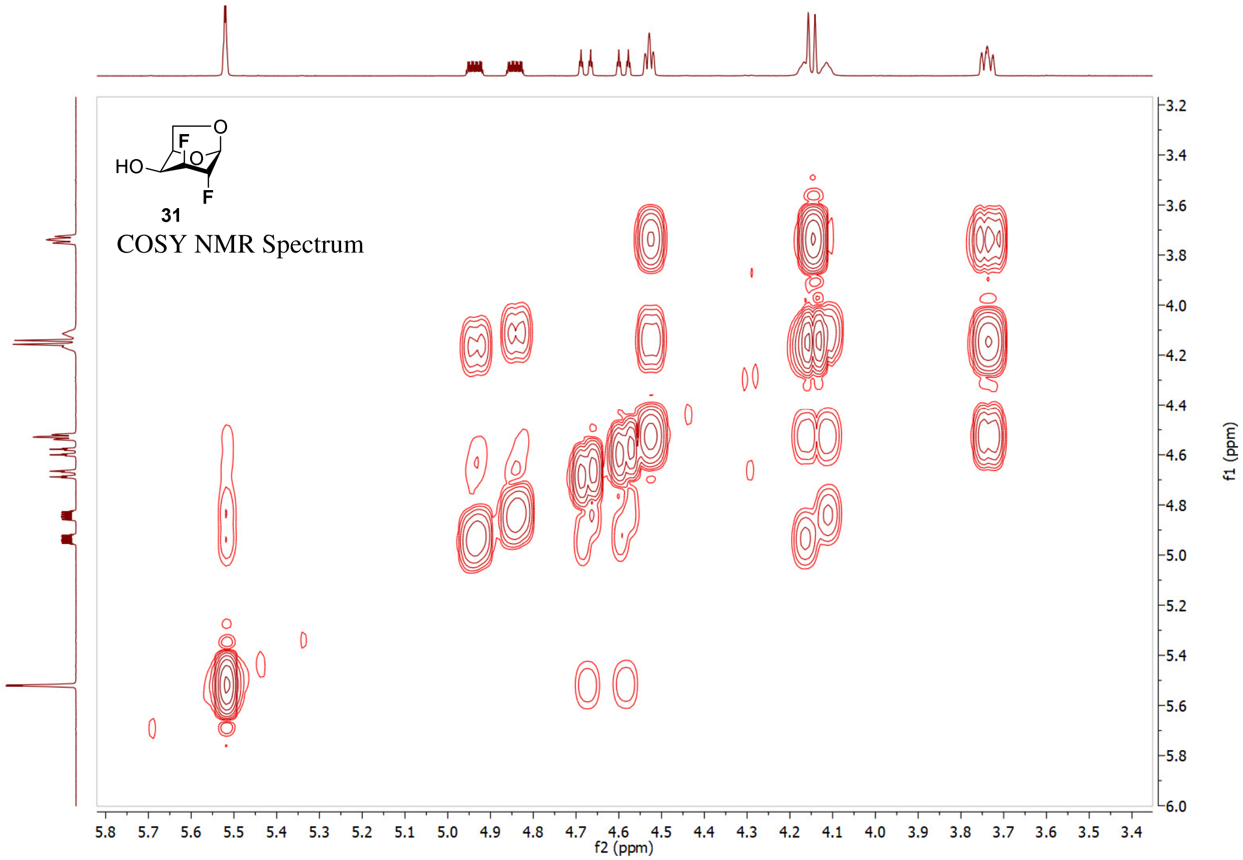

Supplementary Figure 78. COSY NMR spectrum of compound **31**

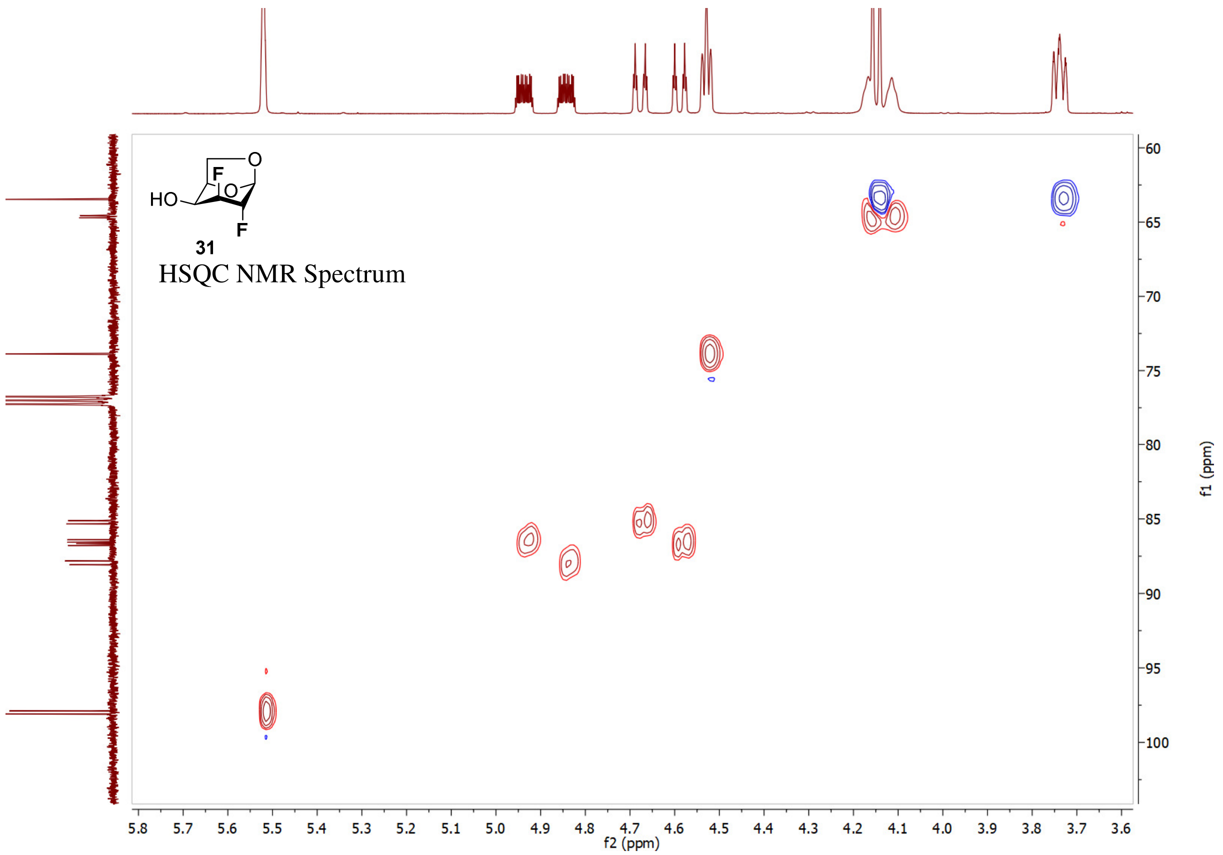

Supplementary Figure 79. HSQC NMR spectrum of compound **31**

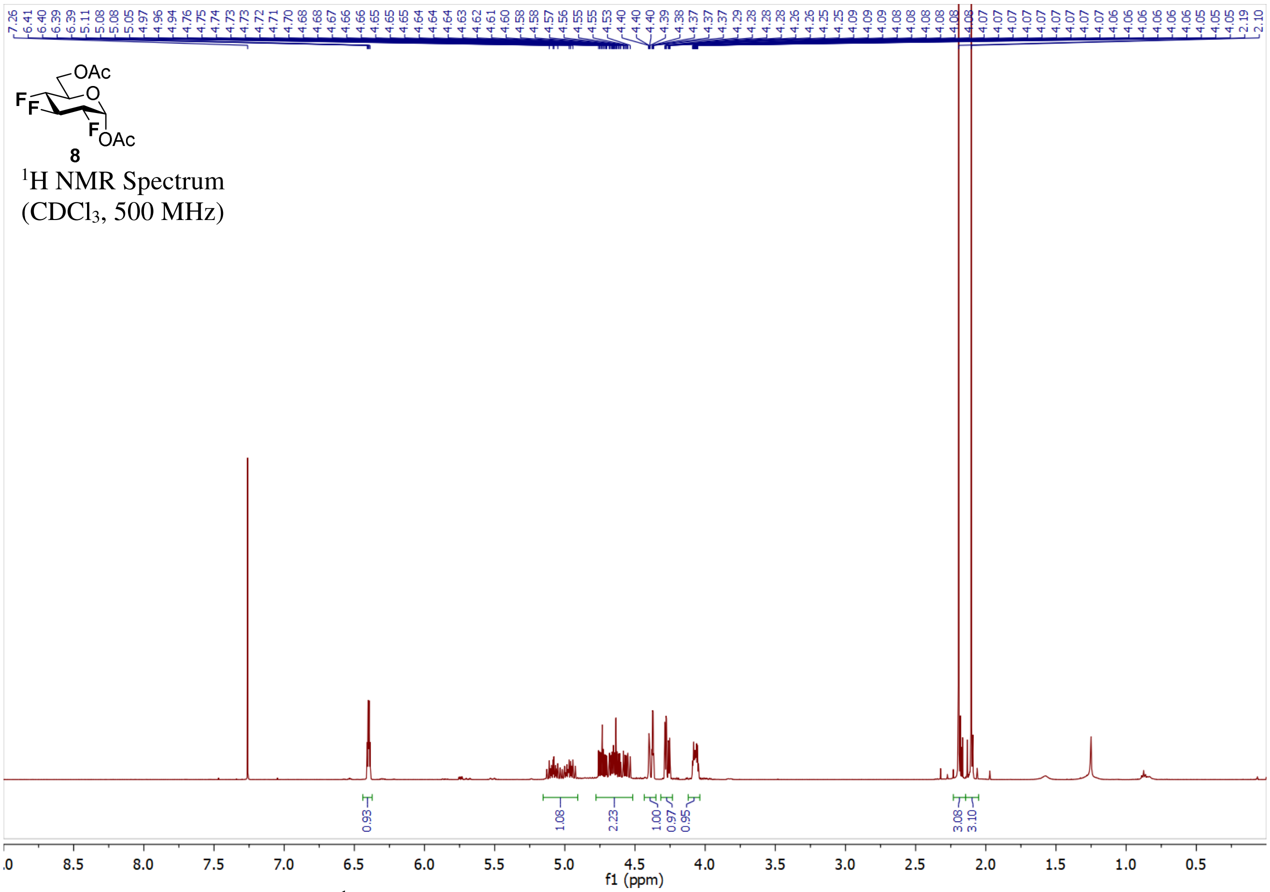

Supplementary Figure 80. ^{1}H NMR spectrum of compound **8**

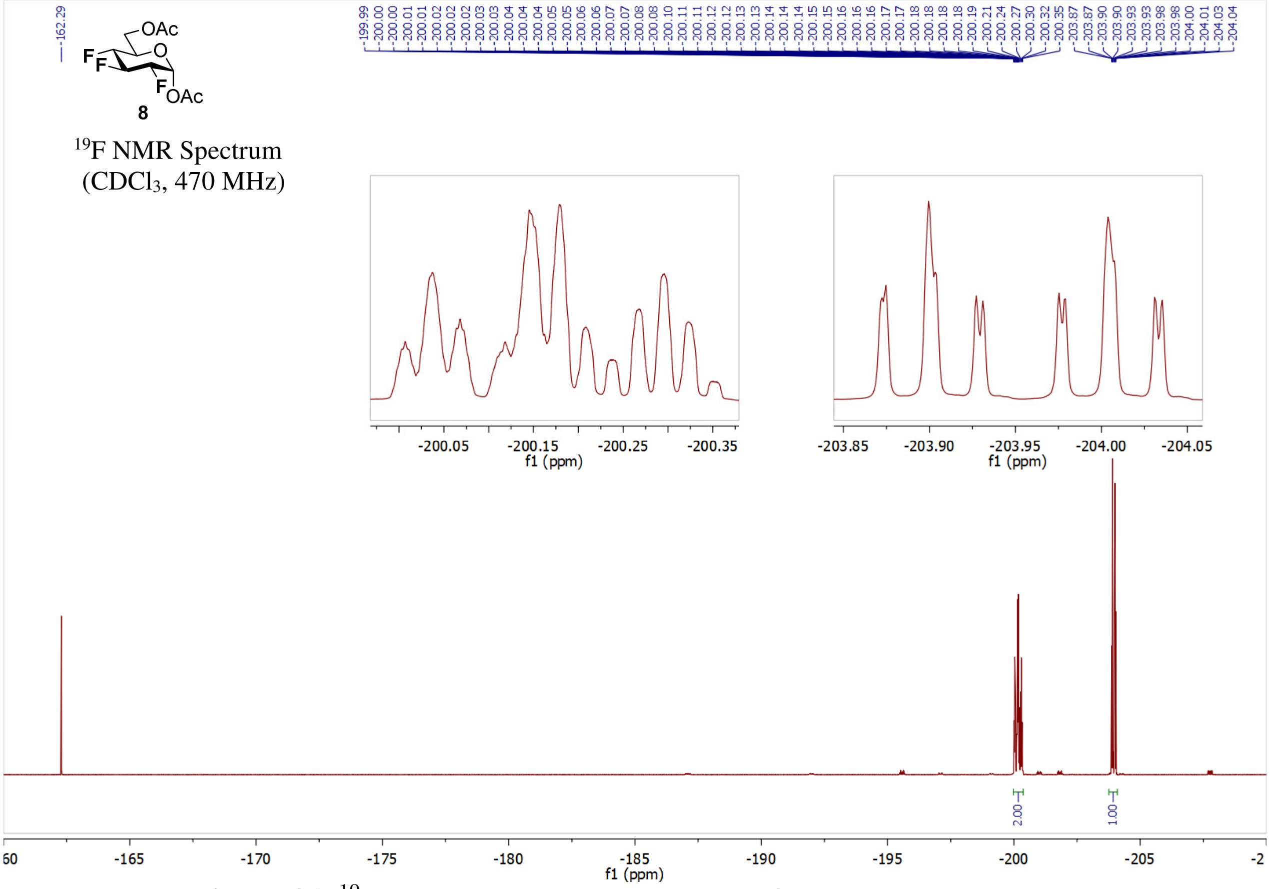

Supplementary Figure 81. ^{19}F NMR spectrum of compound **8**

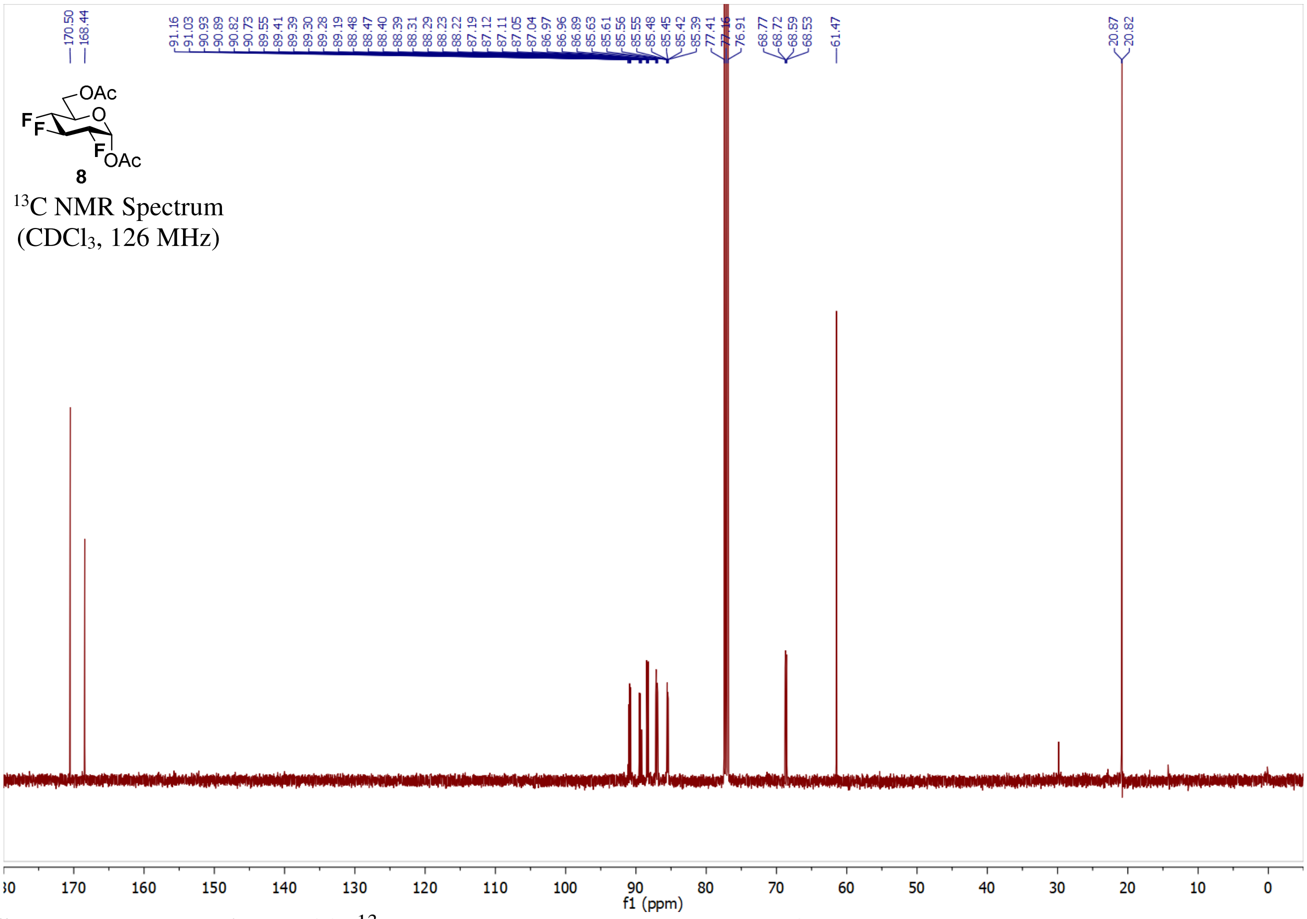

Supplementary Figure 82. ^{13}C NMR spectrum of compound **8**

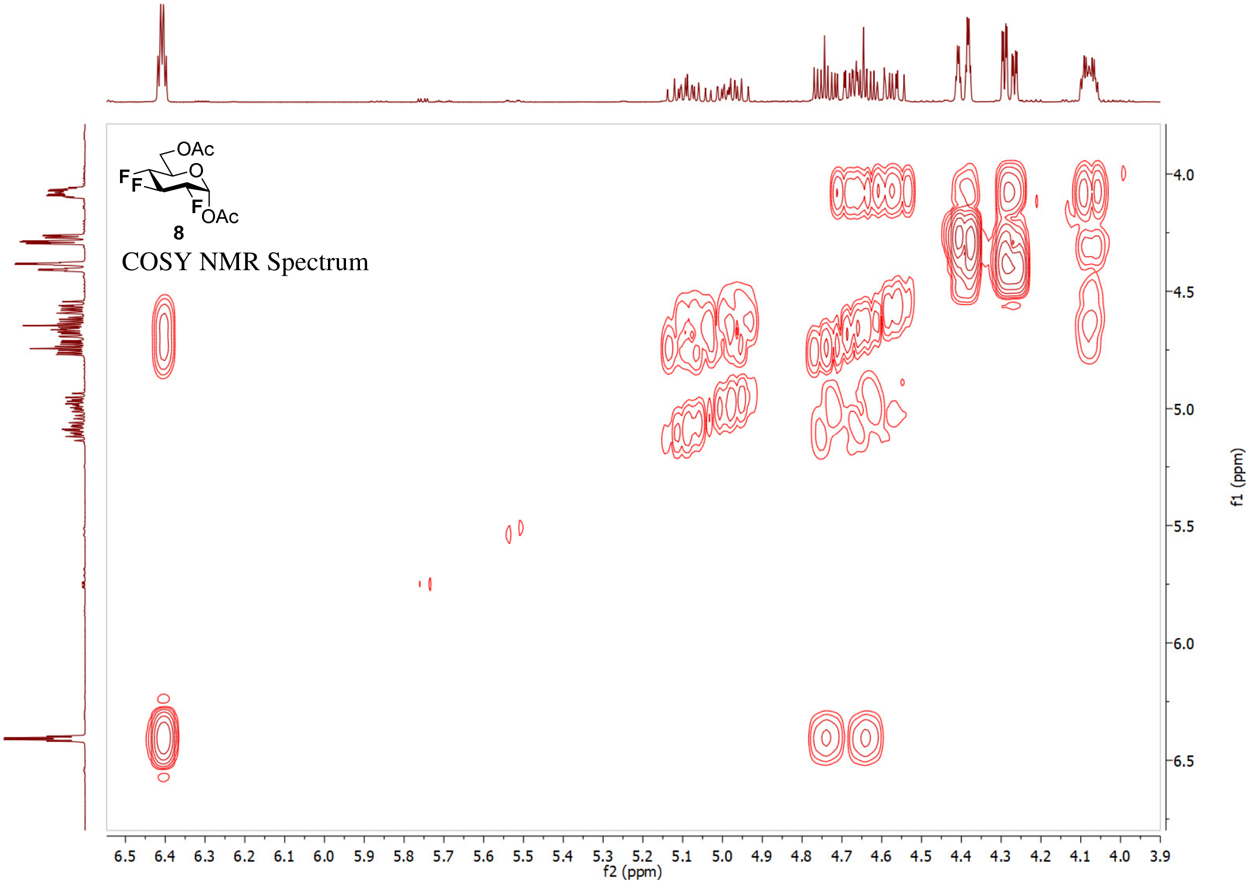

Supplementary Figure 83. COSY NMR spectrum of compound **8**

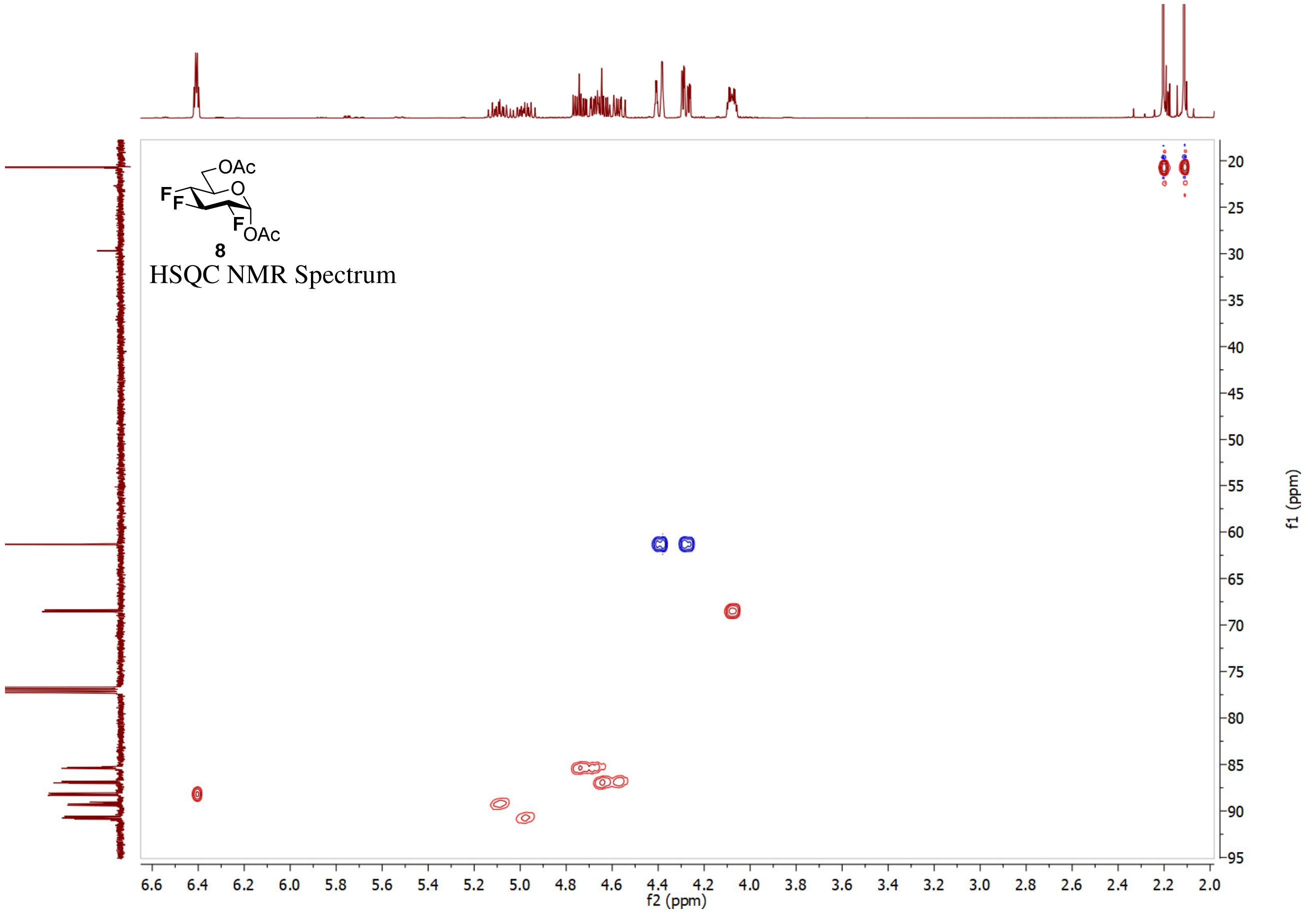

Supplementary Figure 84. HSQC NMR spectrum of compound **8**

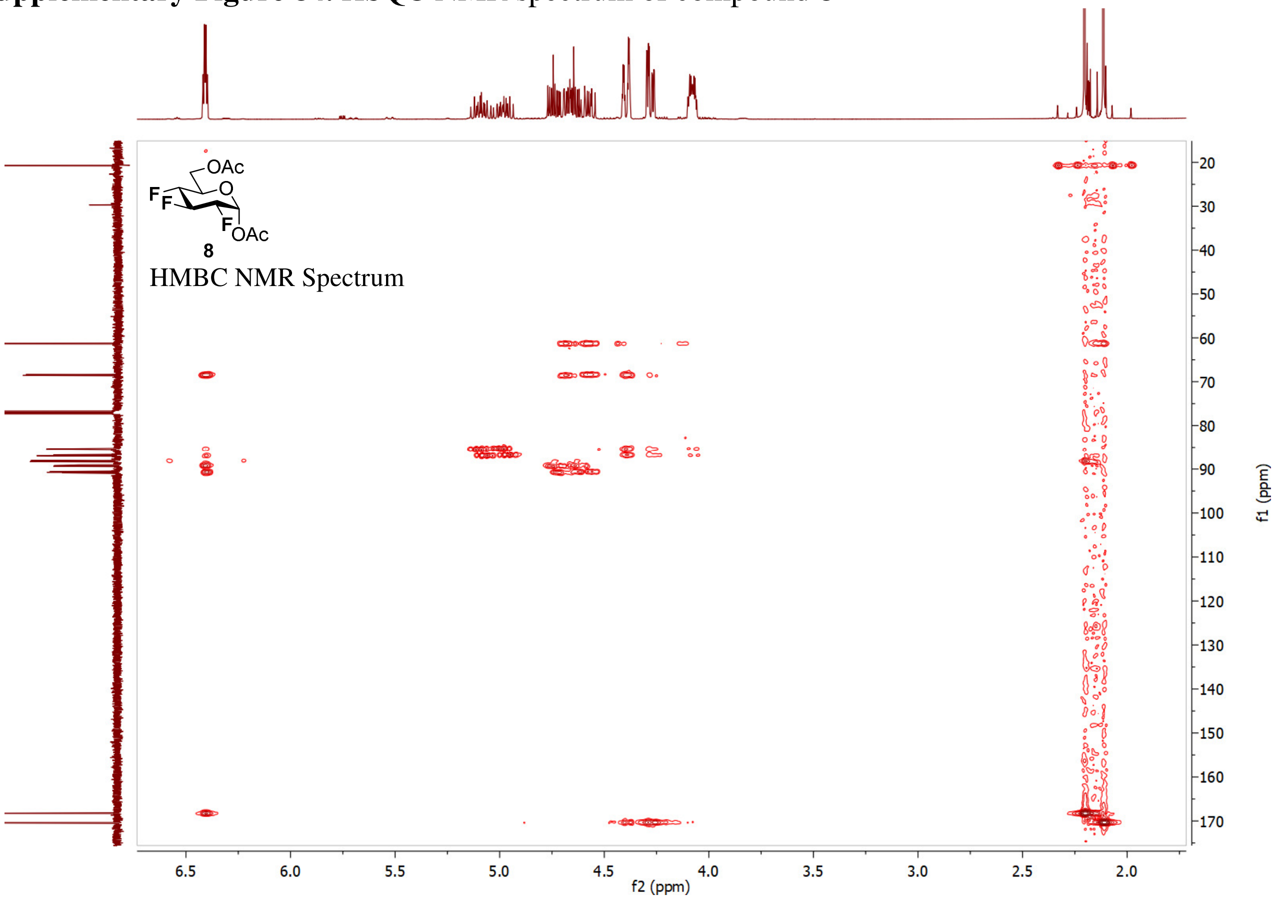

Supplementary Figure 85. HMBC NMR spectrum of compound **8**

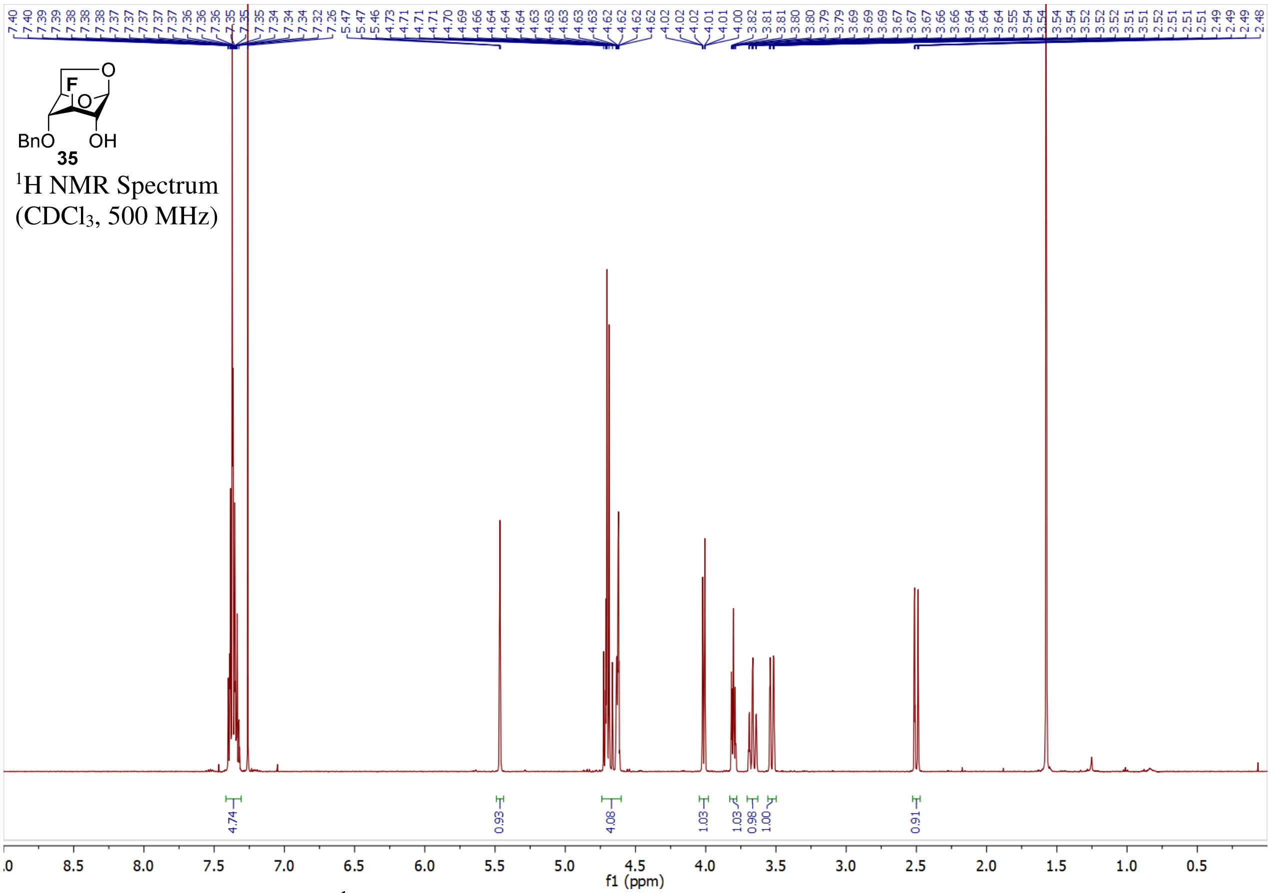

Supplementary Figure 86. ^{1}H NMR spectrum of compound **35**

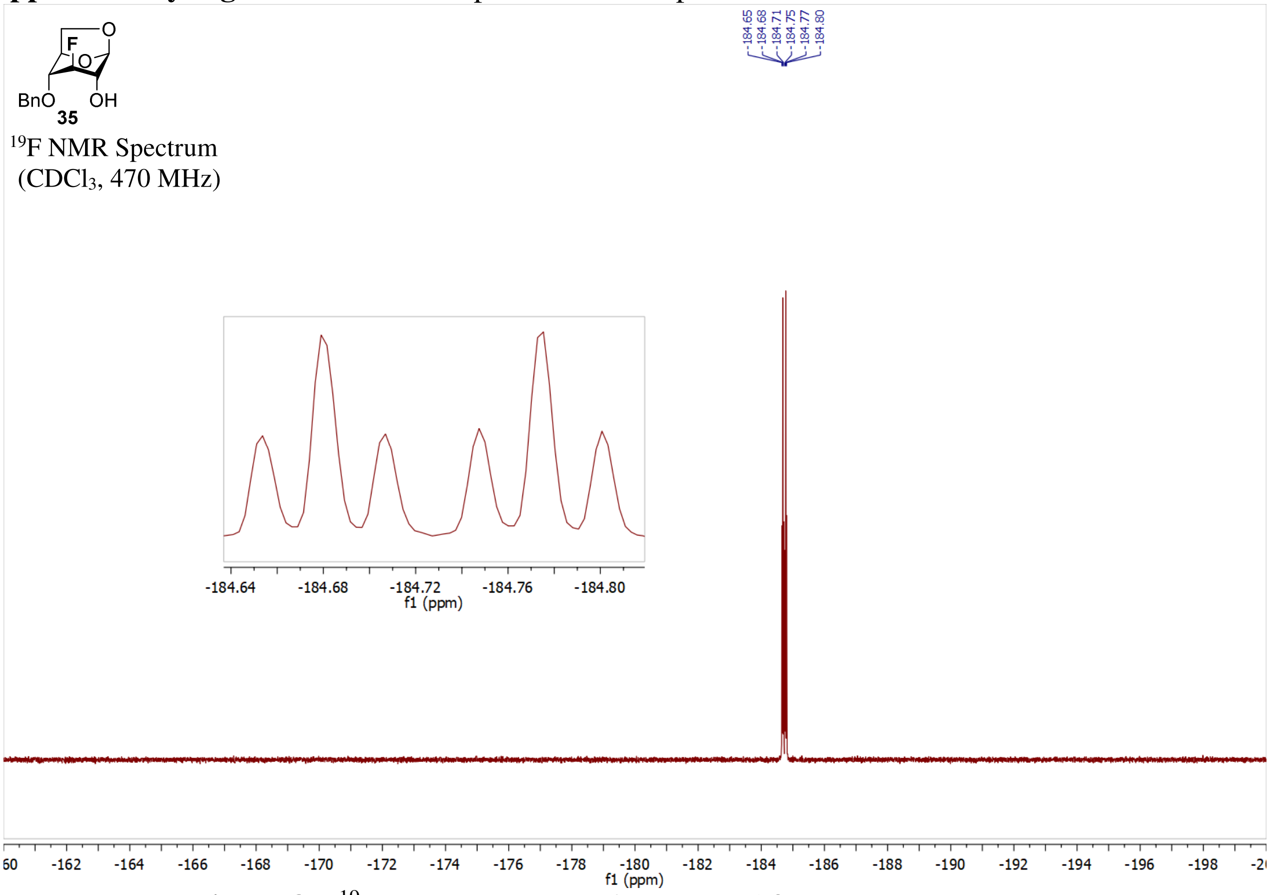

Supplementary Figure 87. ^{19}F NMR spectrum of compound **35**

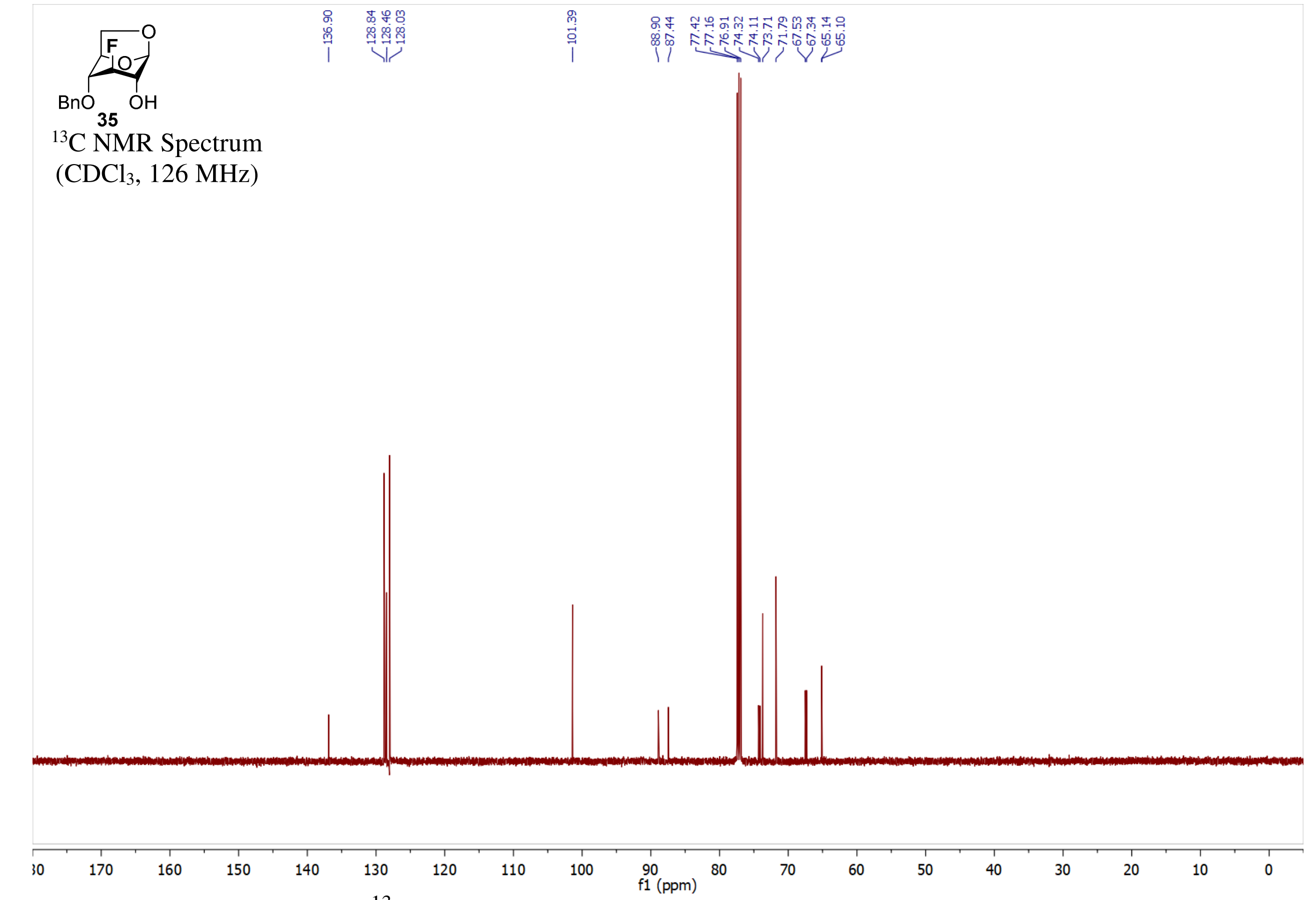

Supplementary Figure 88. ^{13}C NMR spectrum of compound **35**

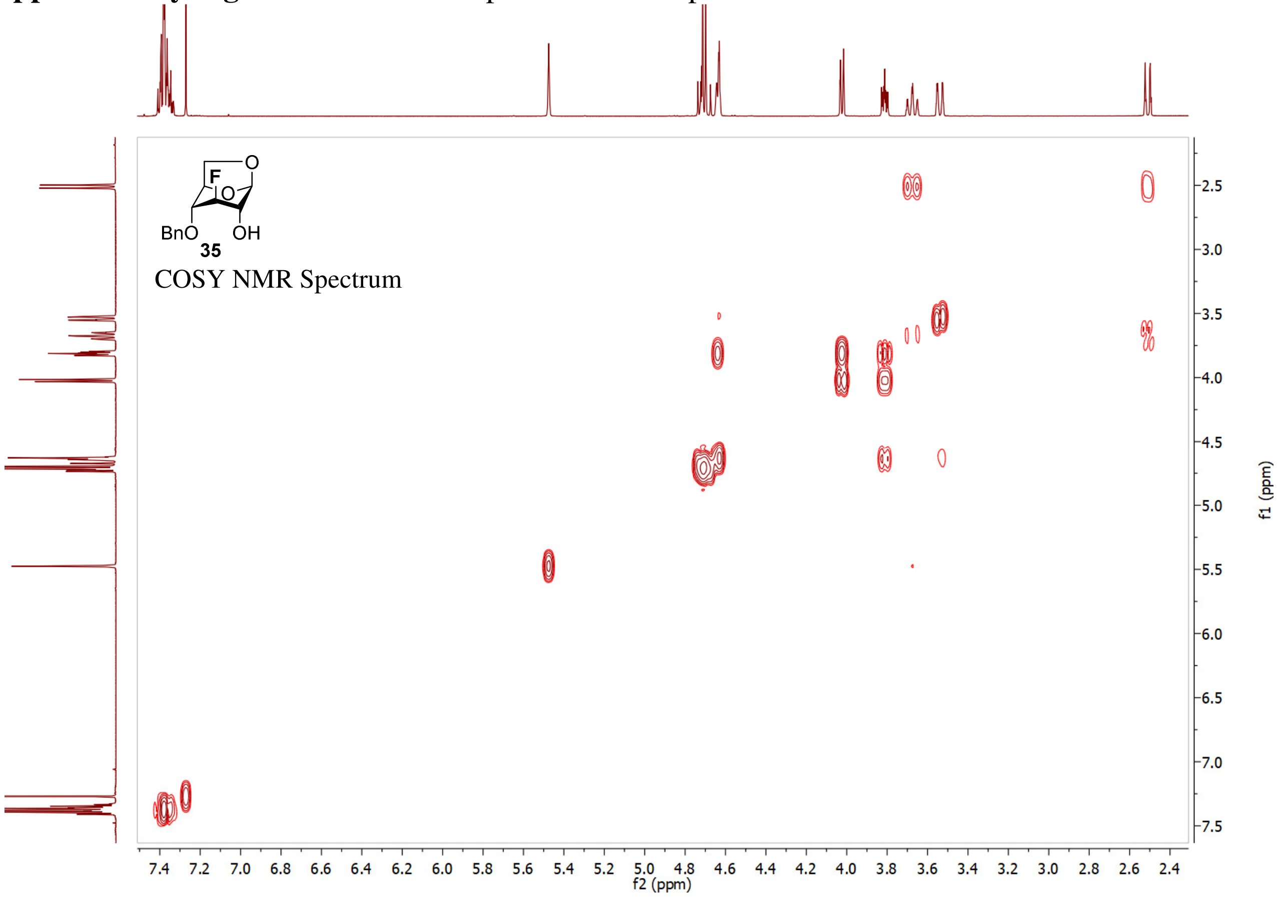

Supplementary Figure 89. COSY NMR spectrum of compound **35**

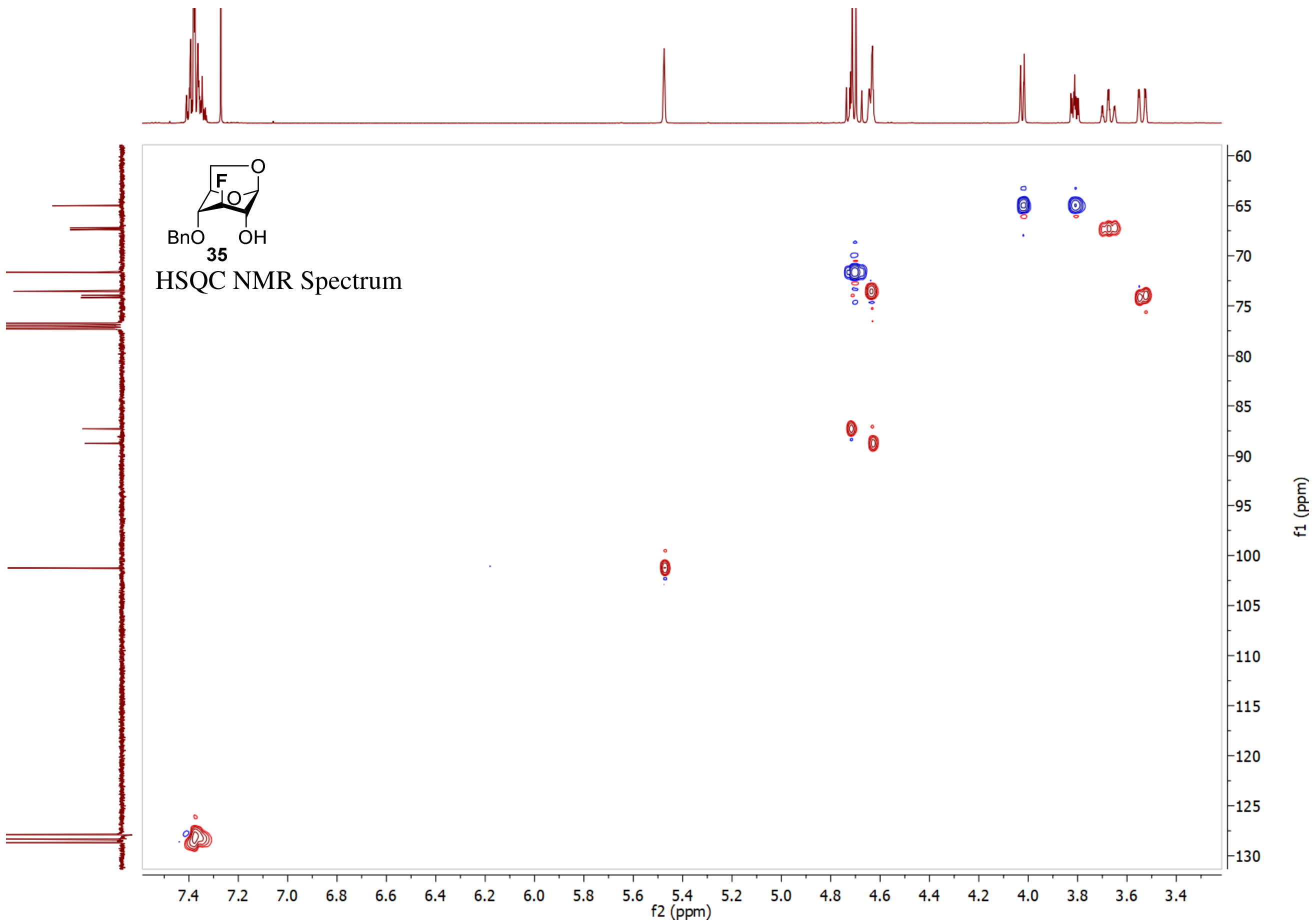

Supplementary Figure 90. HSQC NMR spectrum of compound **35**

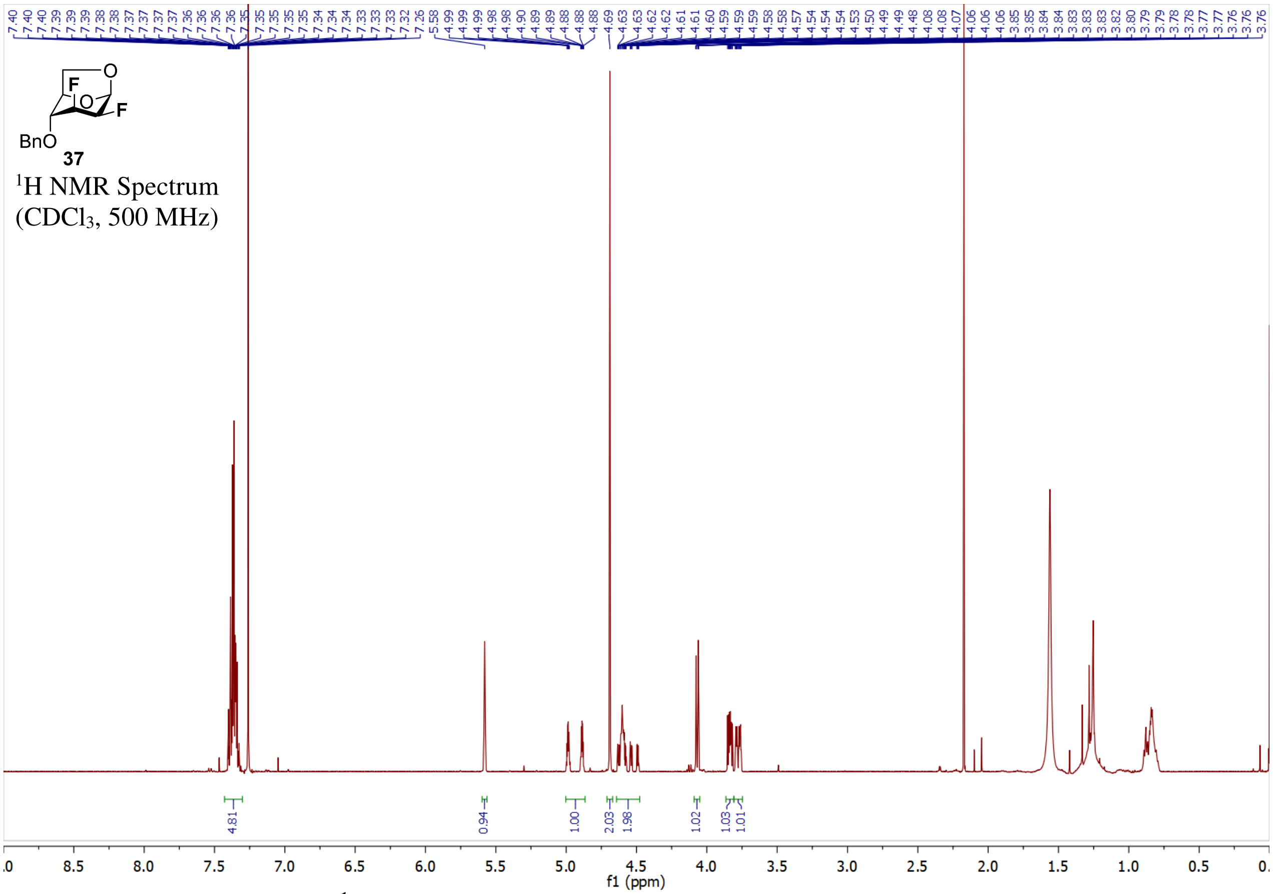

Supplementary Figure 91. ^{1}H NMR spectrum of compound **37**

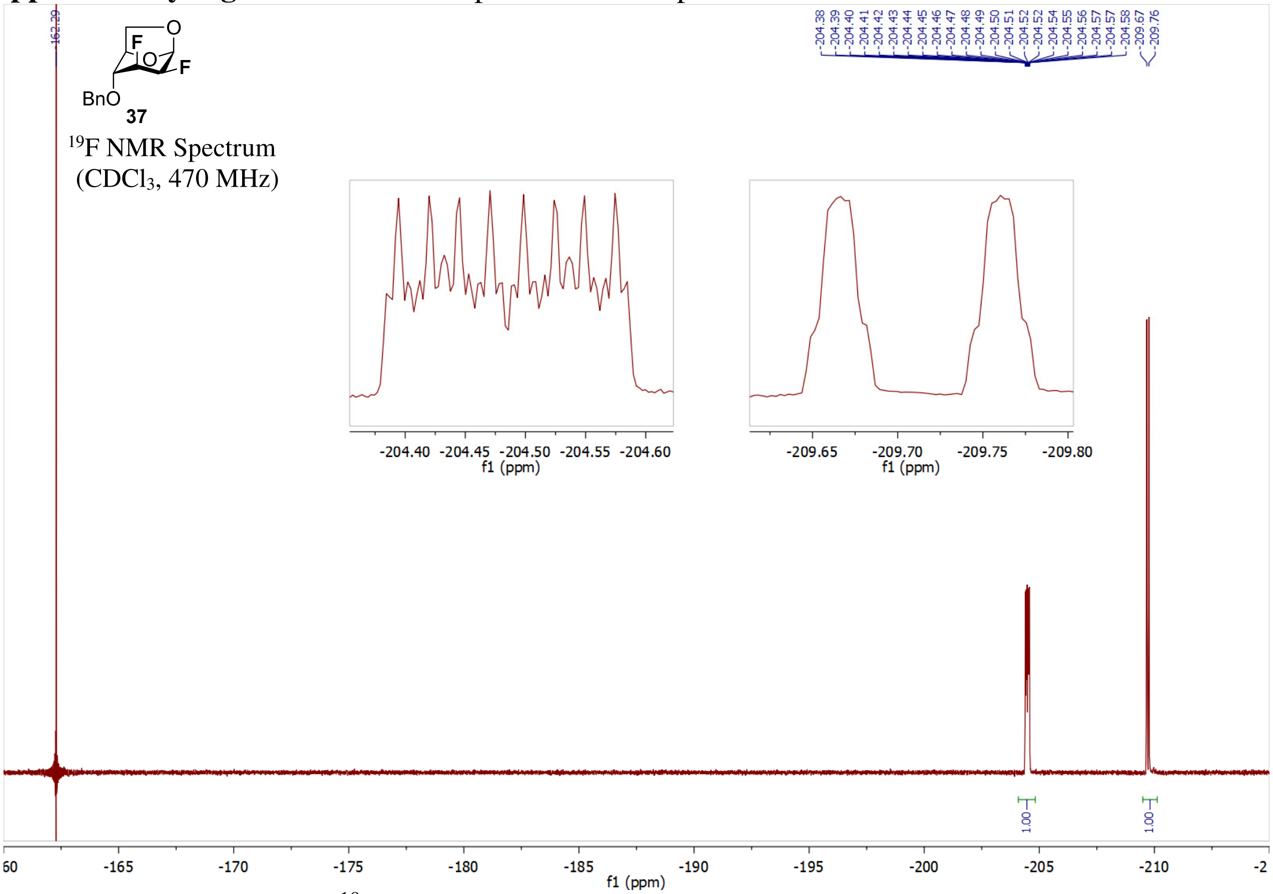

Supplementary Figure 92. ^{19}F NMR spectrum of compound **37**

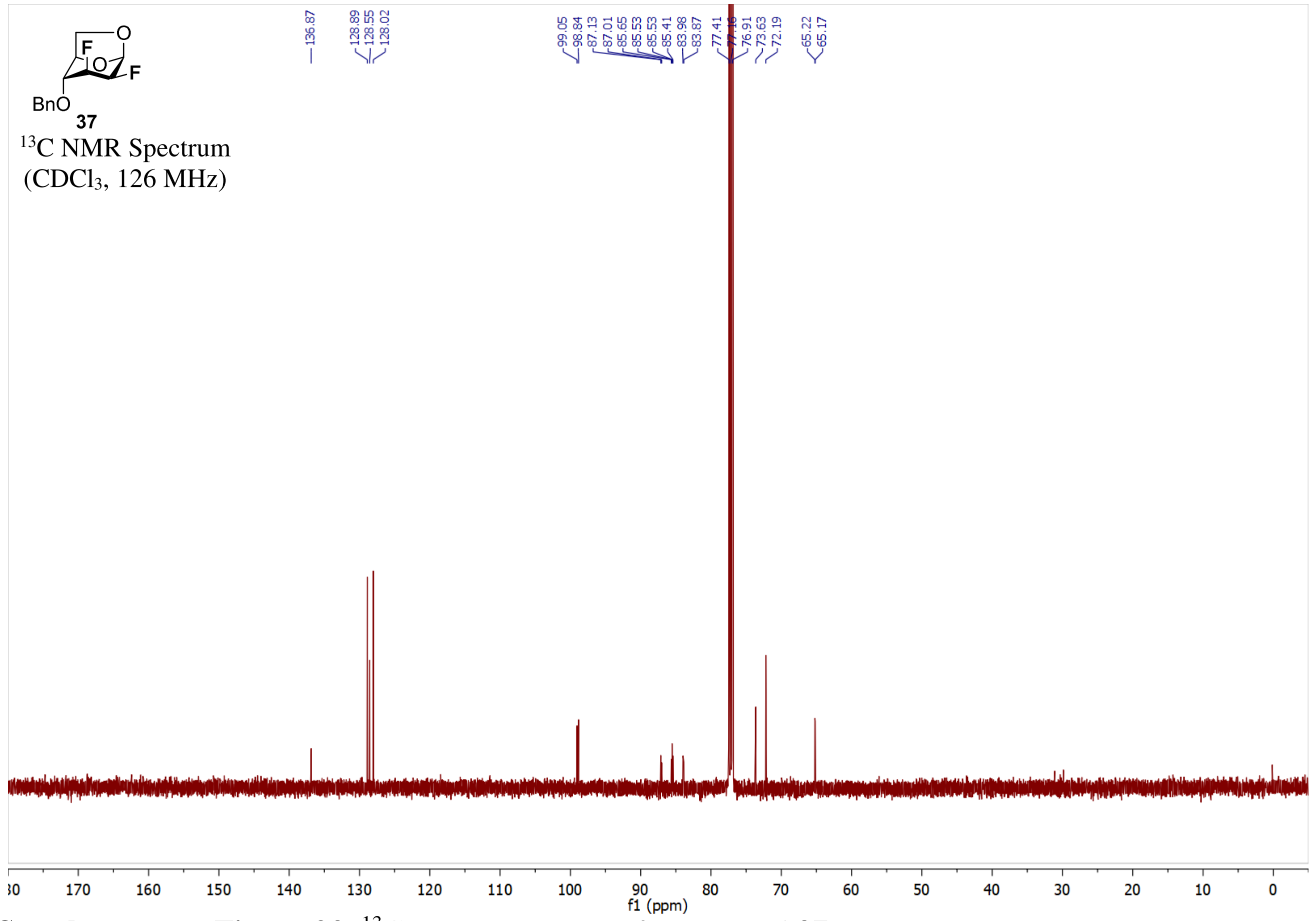

Supplementary Figure 93. ^{13}C NMR spectrum of compound **37**

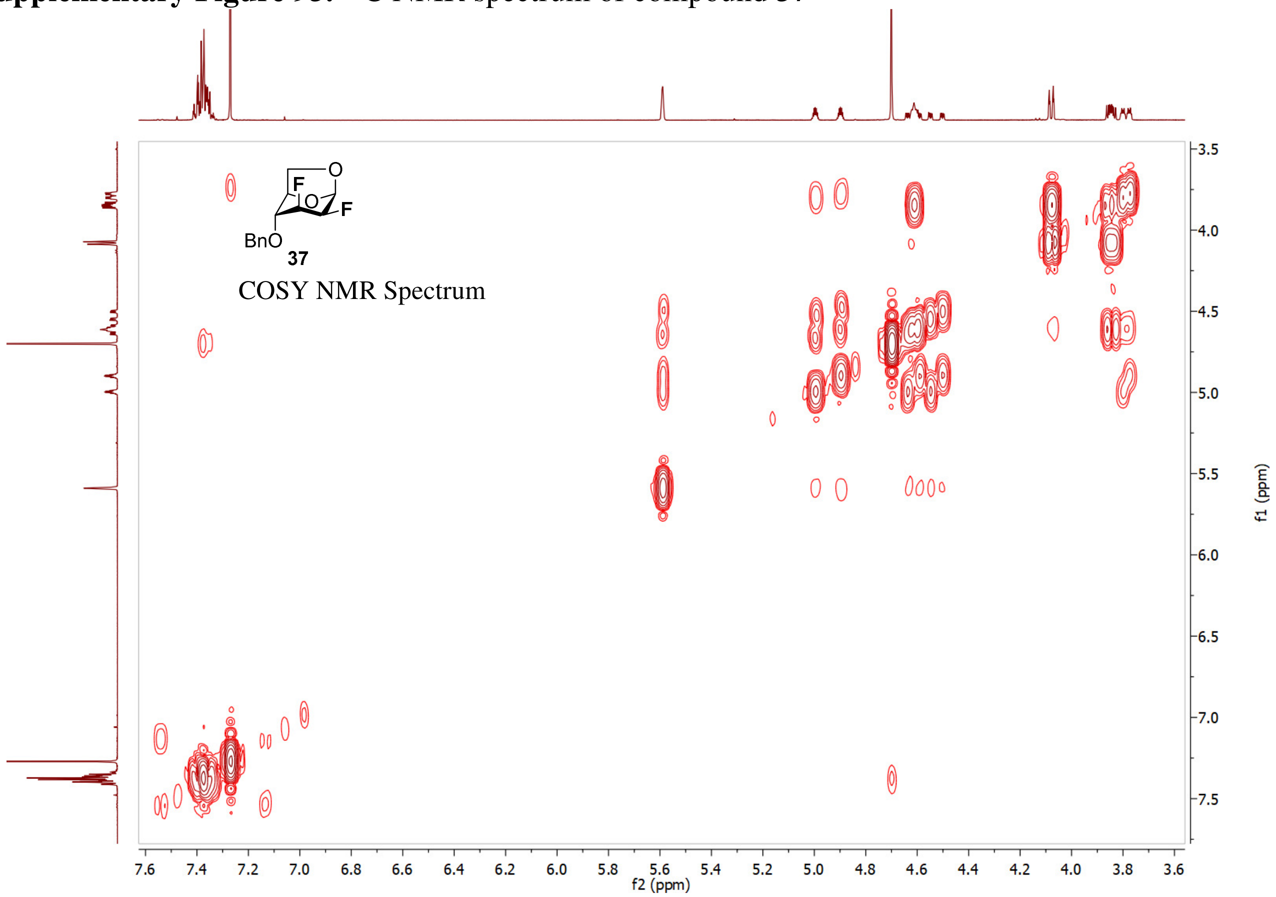

Supplementary Figure 94. COSY NMR spectrum of compound **37**

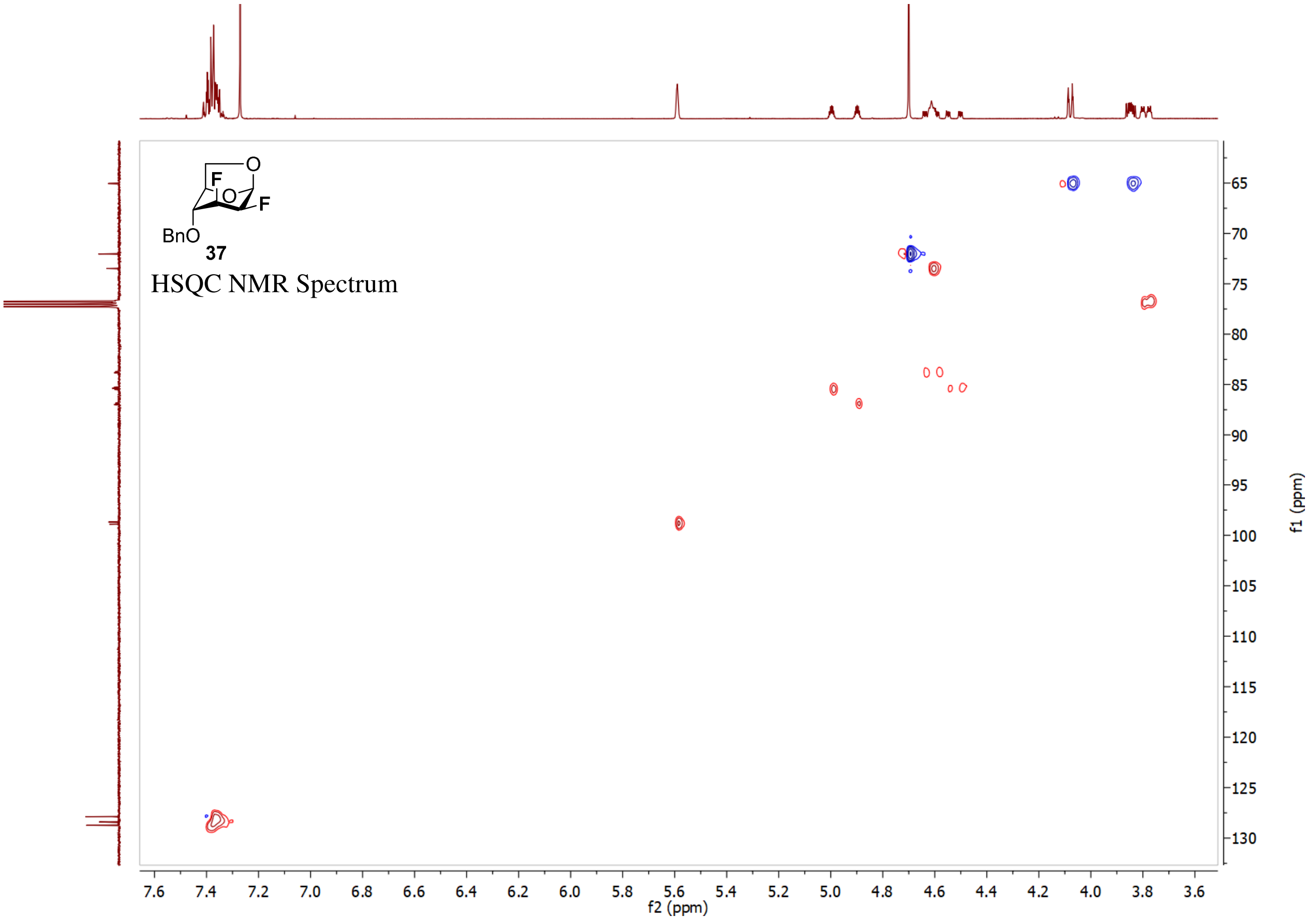

Supplementary Figure 95. HSQC NMR spectrum of compound **37**

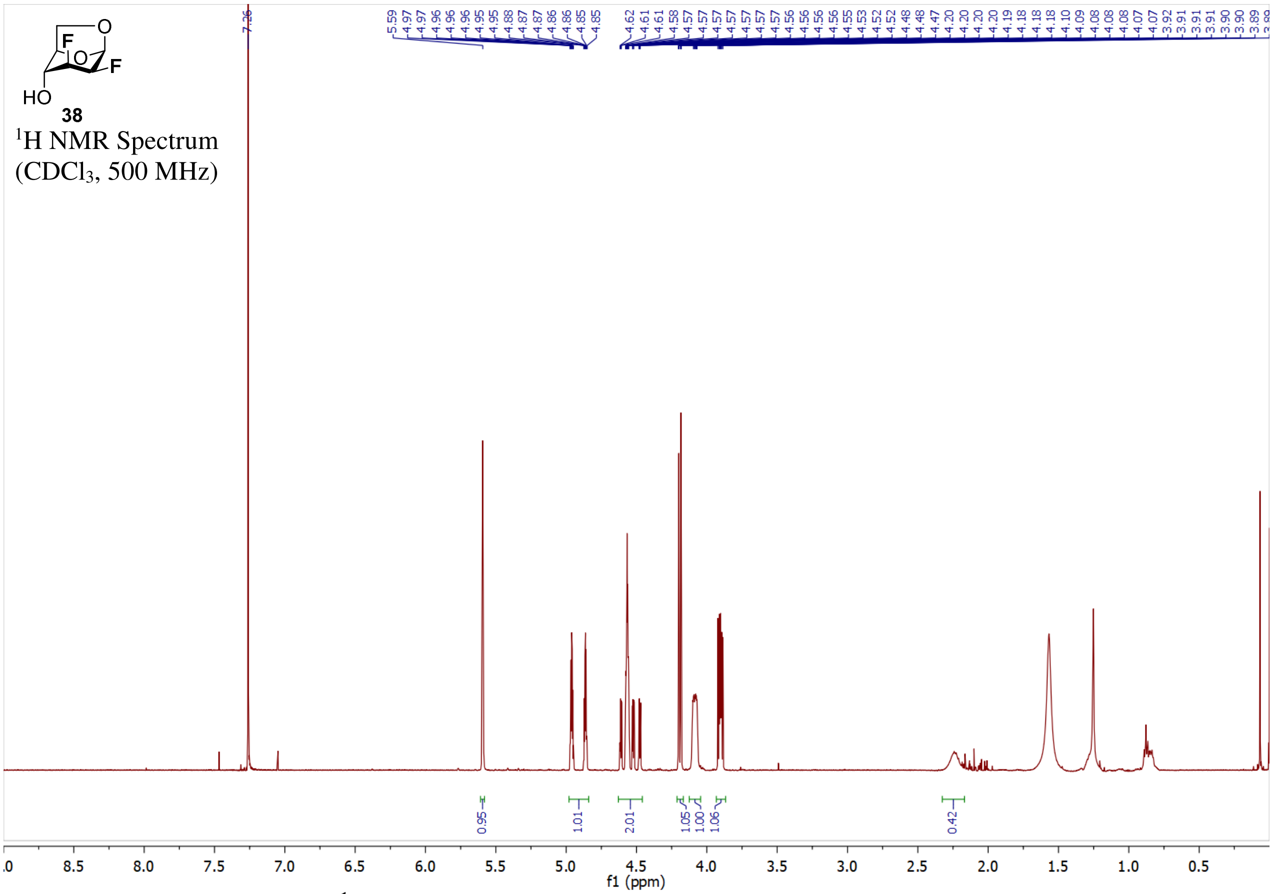

Supplementary Figure 96. ^{1}H NMR spectrum of compound **38**

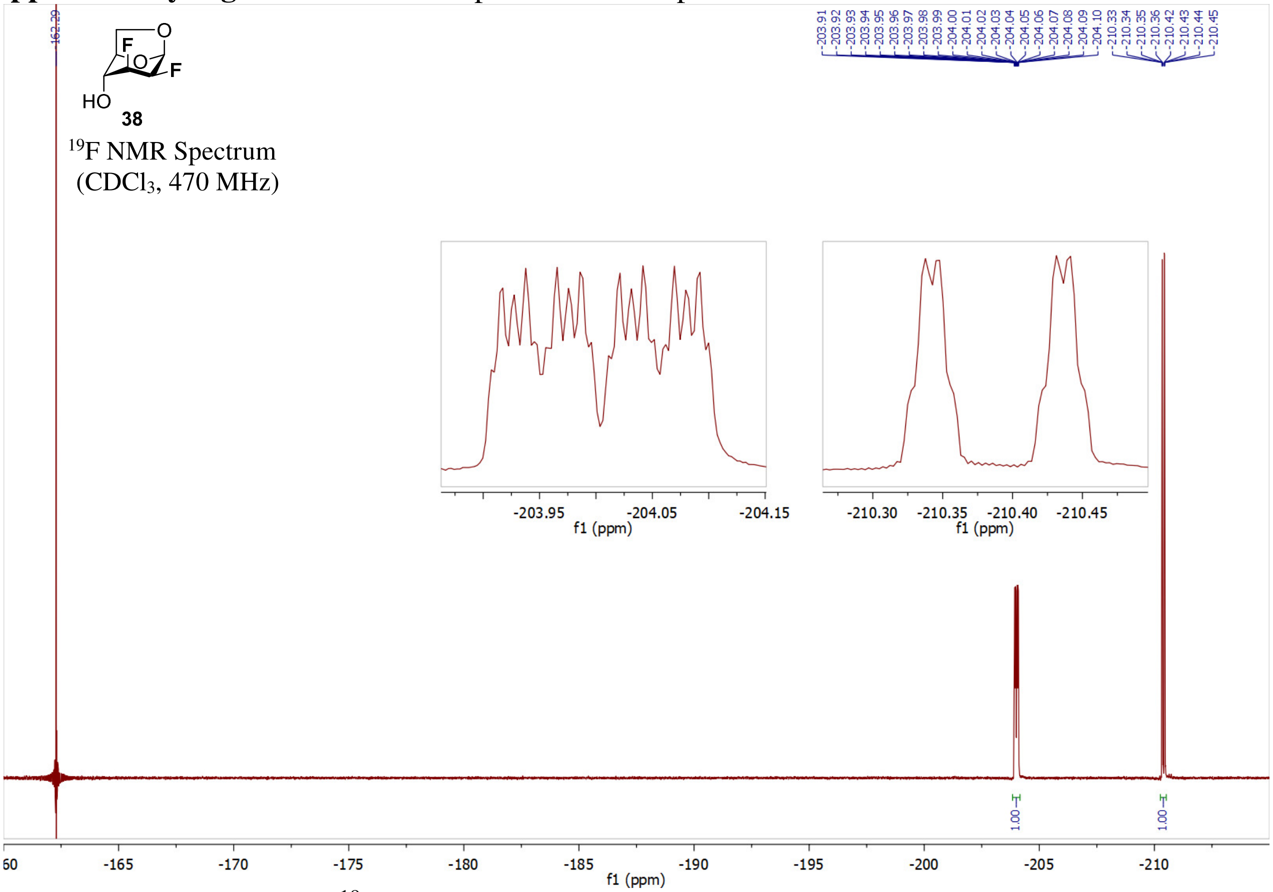

Supplementary Figure 97. ^{19}F NMR spectrum of compound **38**

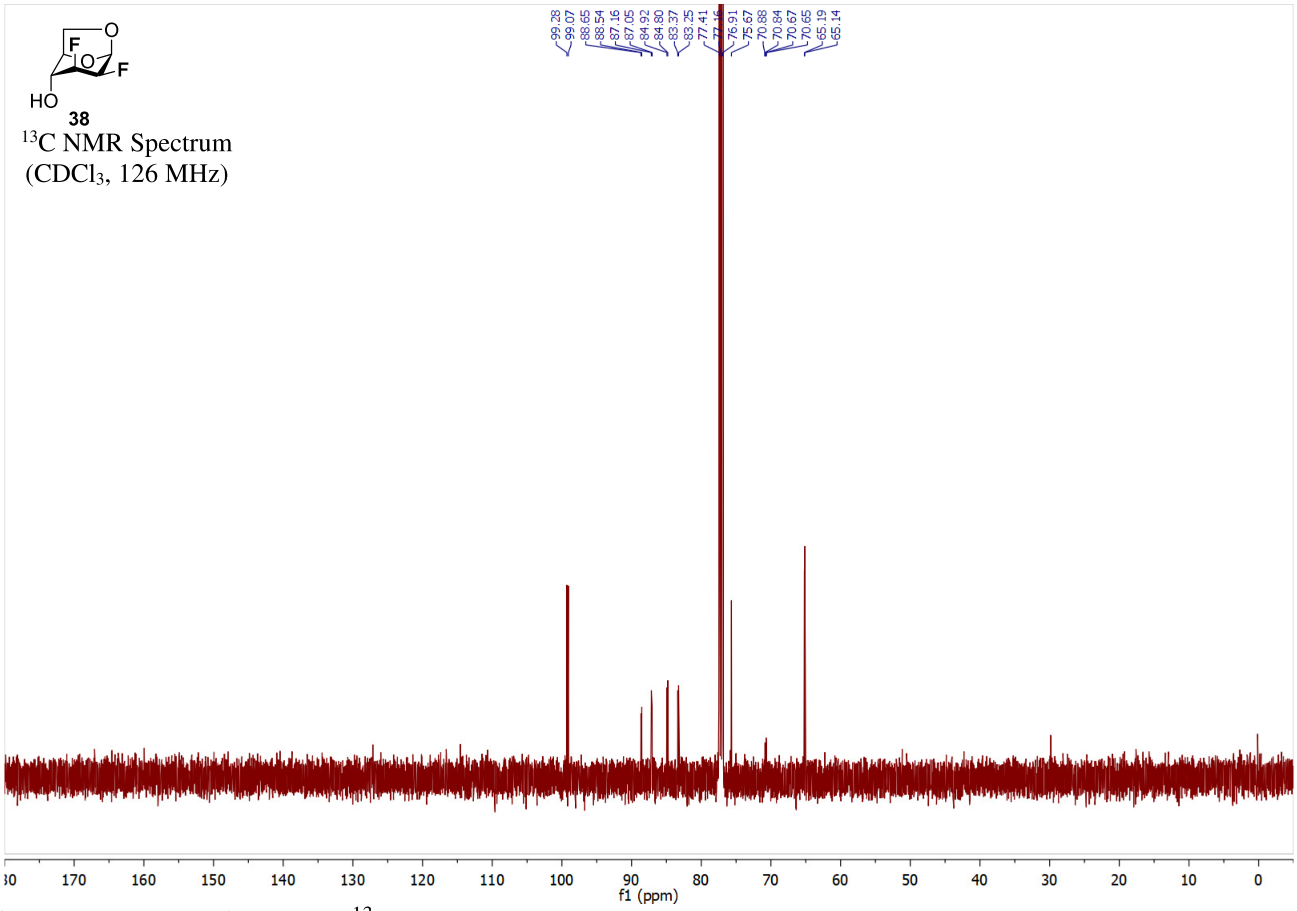

Supplementary Figure 98. ^{13}C NMR spectrum of compound **38**

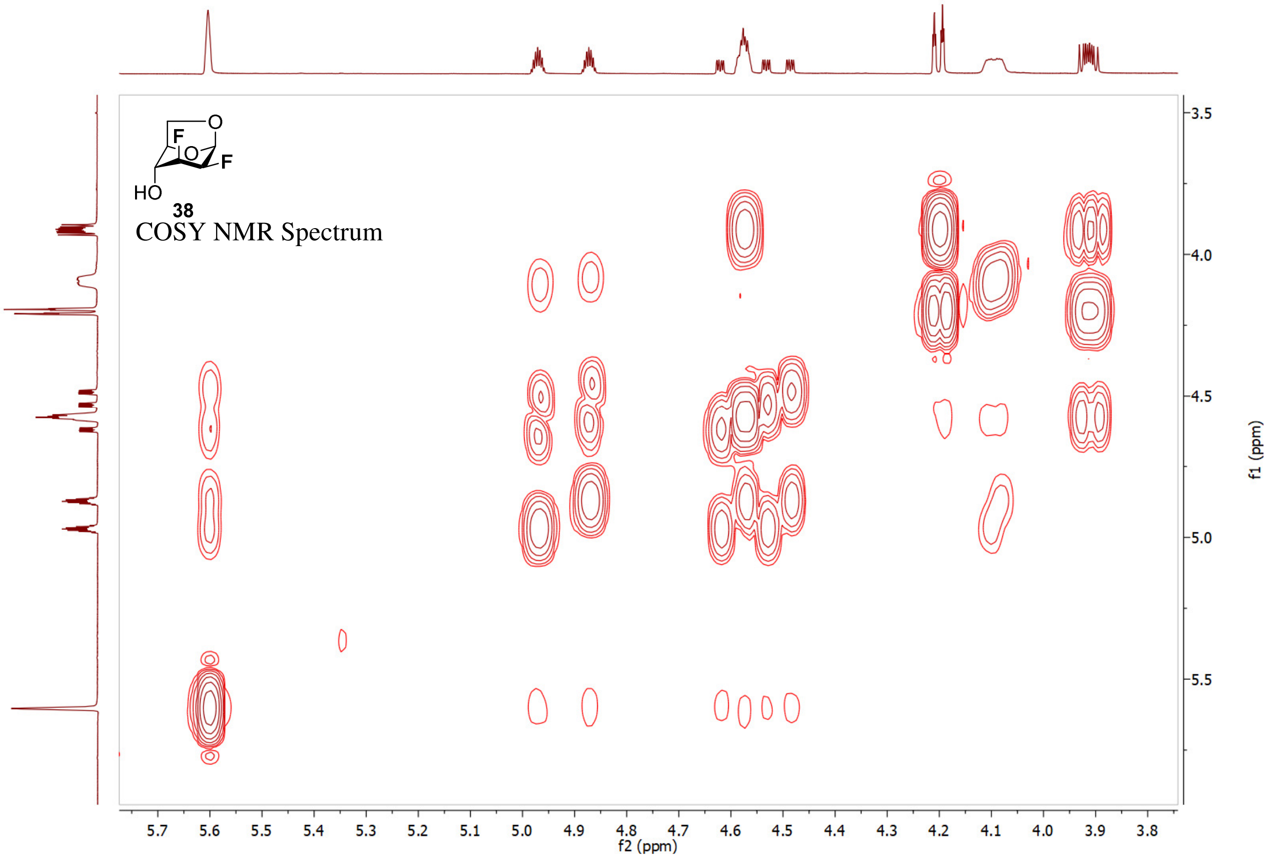

Supplementary Figure 99. COSY NMR spectrum of compound **38**

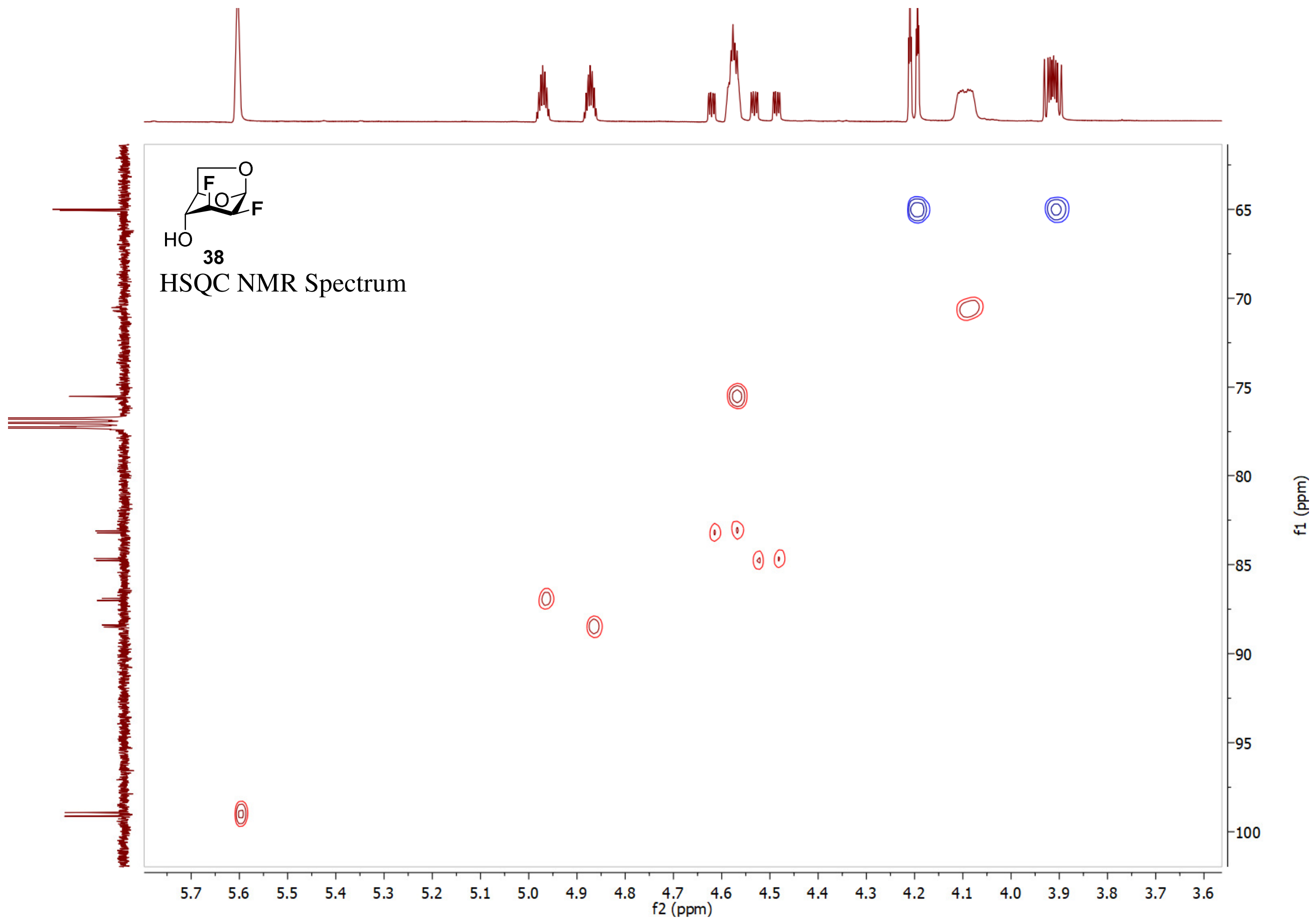

Supplementary Figure 100. HSQC NMR spectrum of compound **38**

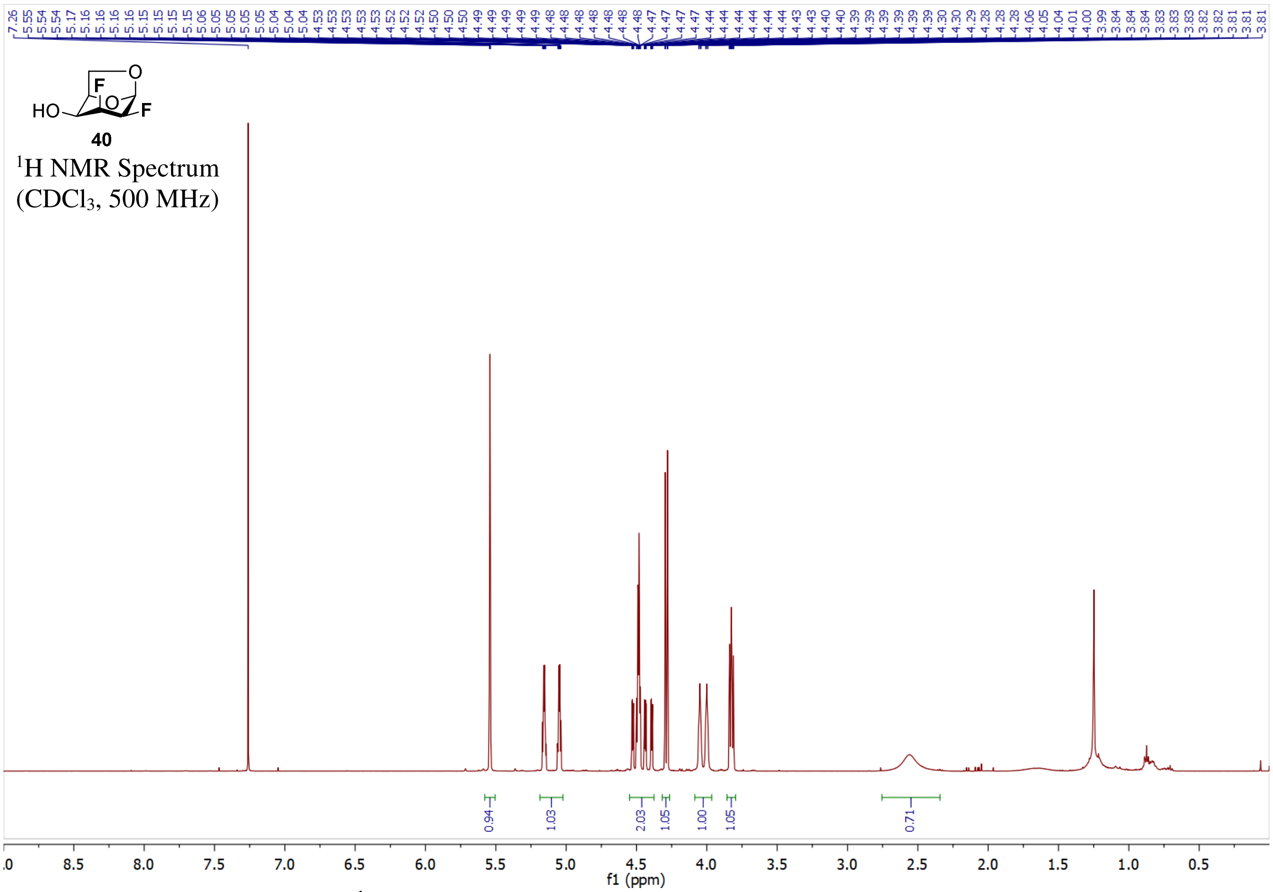

Supplementary Figure 101. ^{1}H NMR spectrum of compound **40**

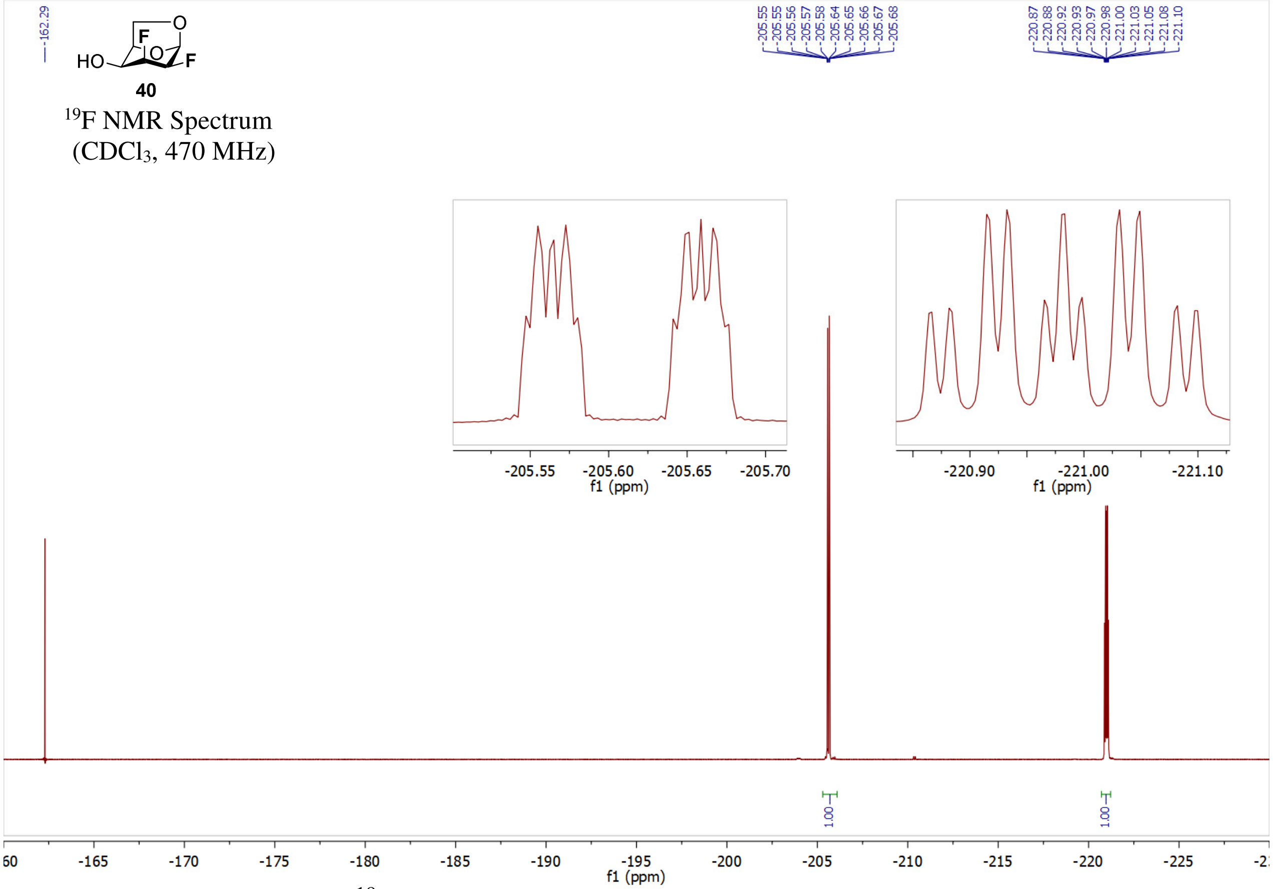

Supplementary Figure 102. ^{19}F NMR spectrum of compound **40**

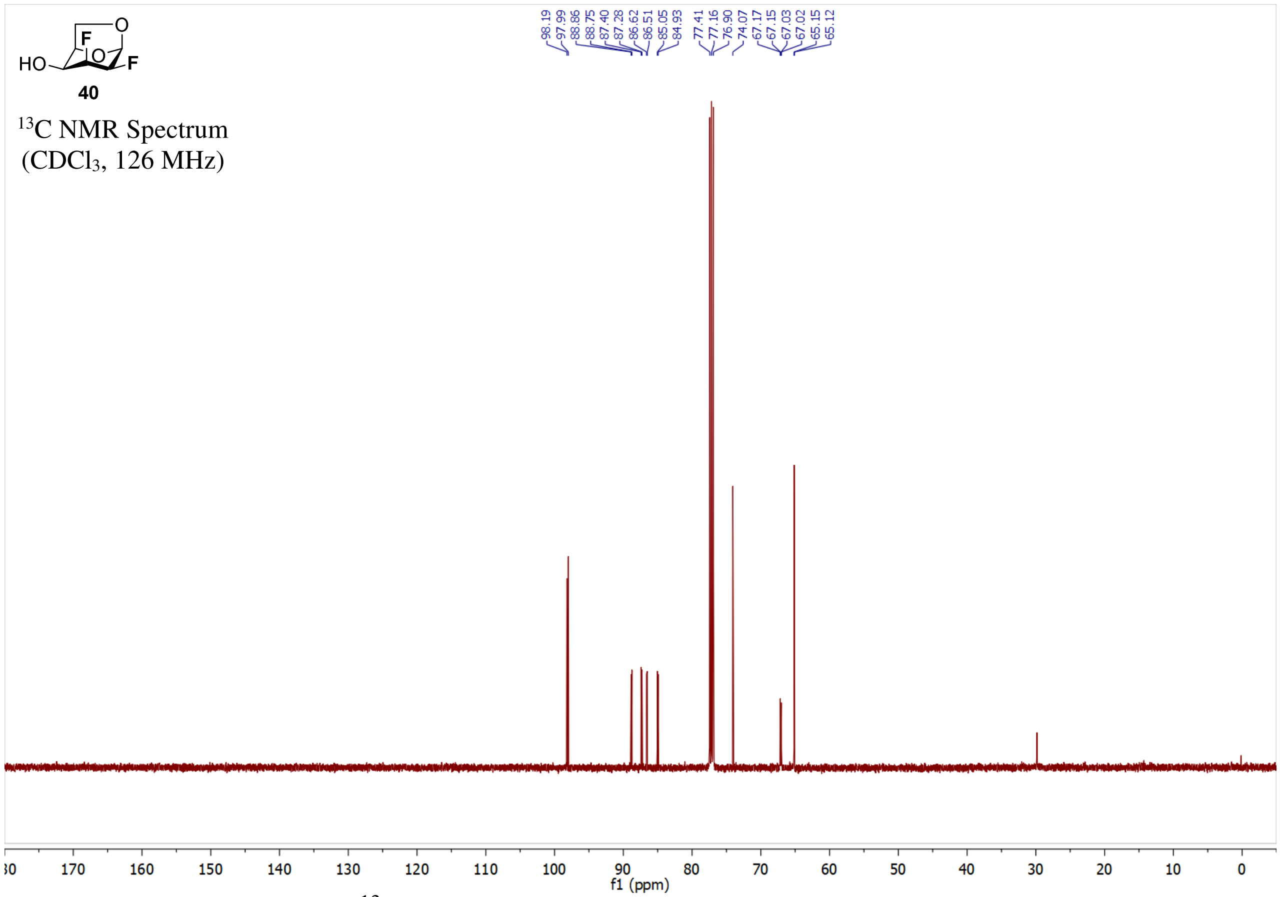

Supplementary Figure 103. ^{13}C NMR spectrum of compound **40**

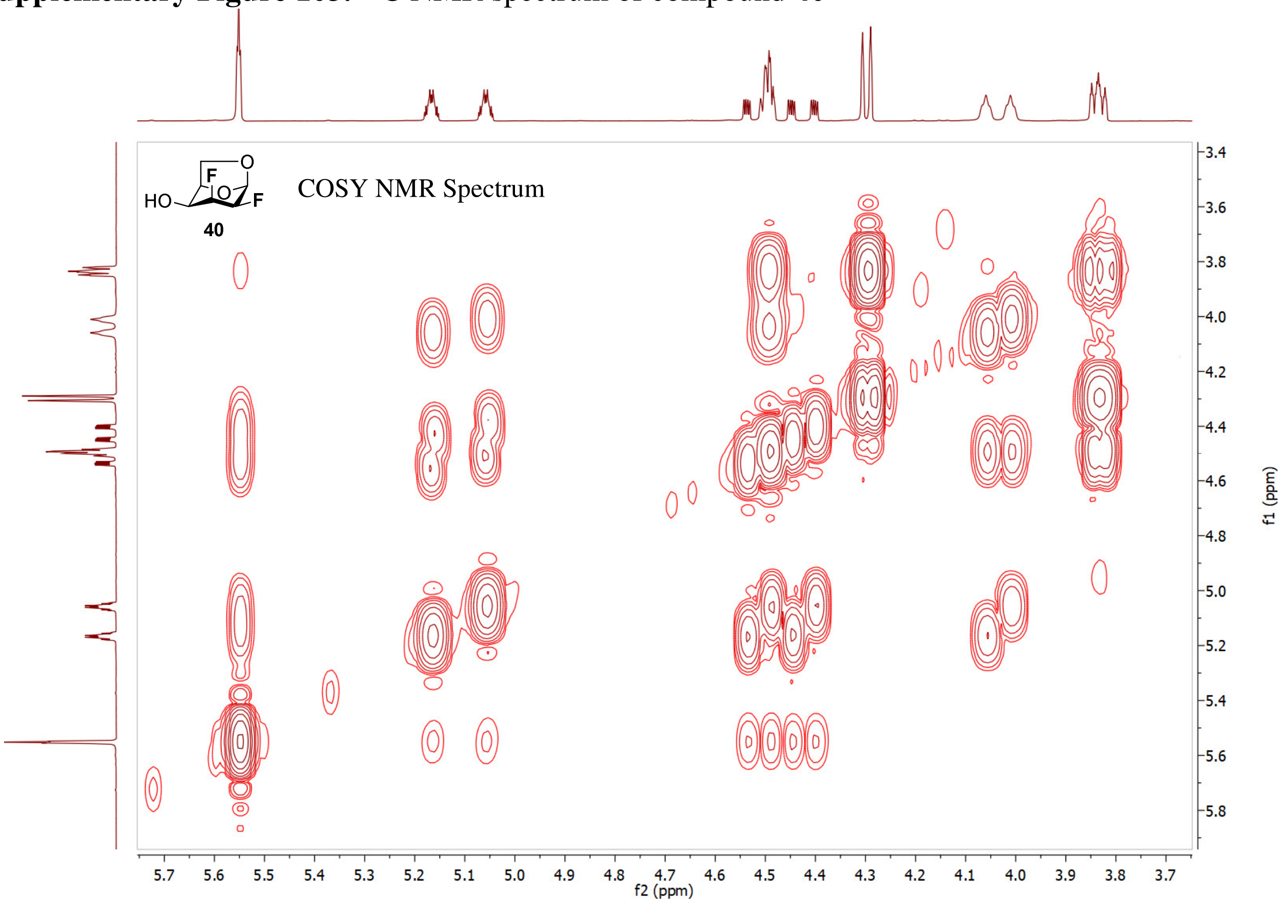

Supplementary Figure 104. COSY NMR spectrum of compound **40**

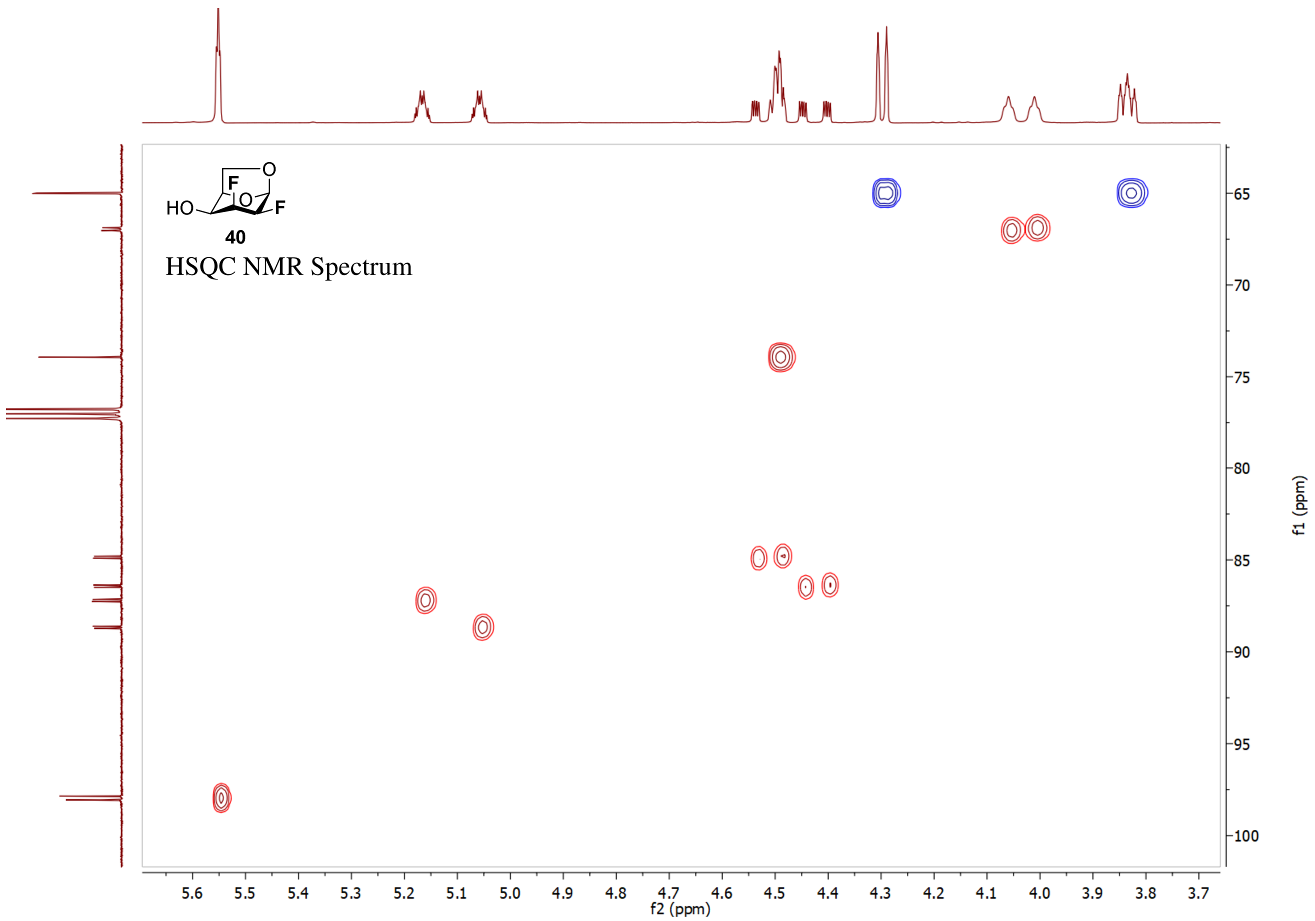

Supplementary Figure 105. HSQC NMR spectrum of compound **40**

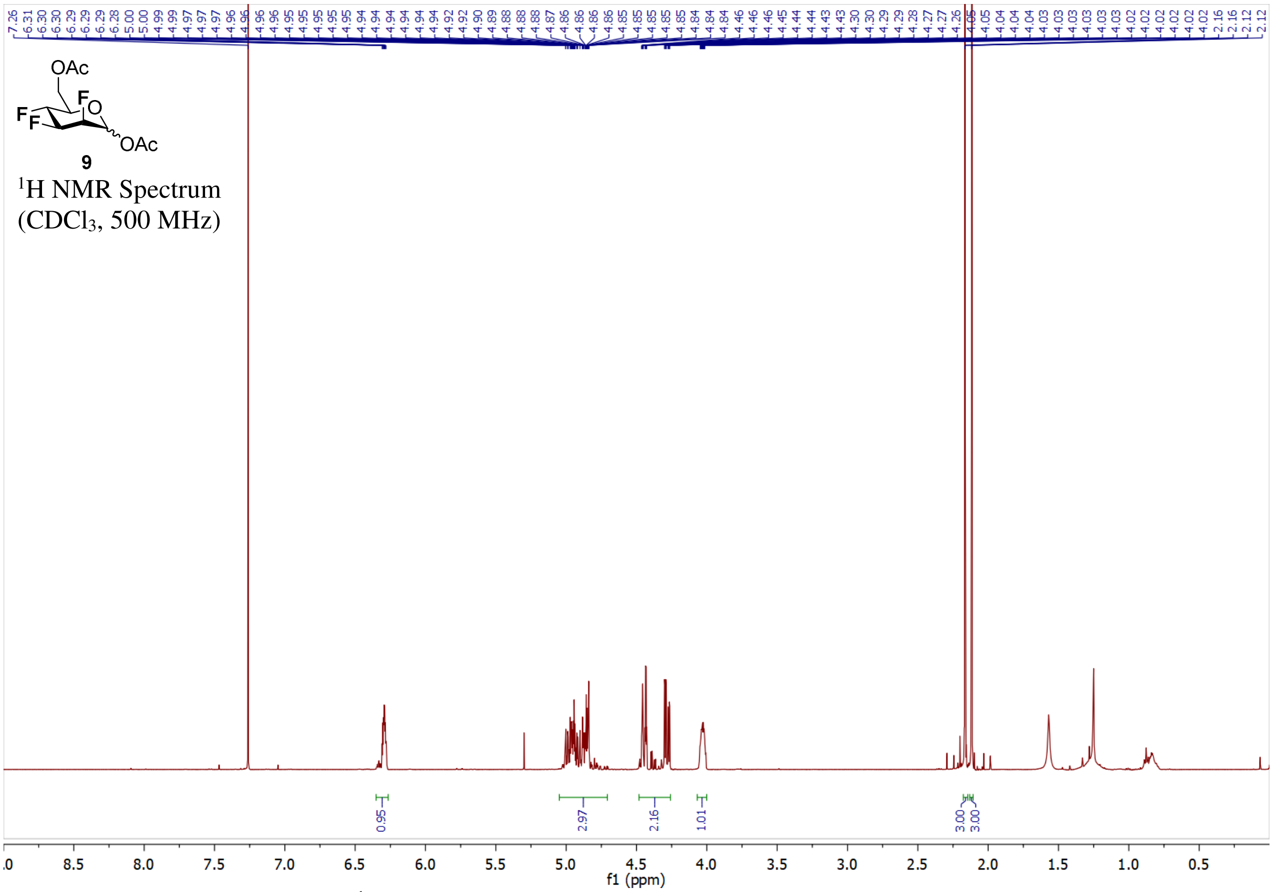

Supplementary Figure 106. ^{1}H NMR spectrum of compound **9**

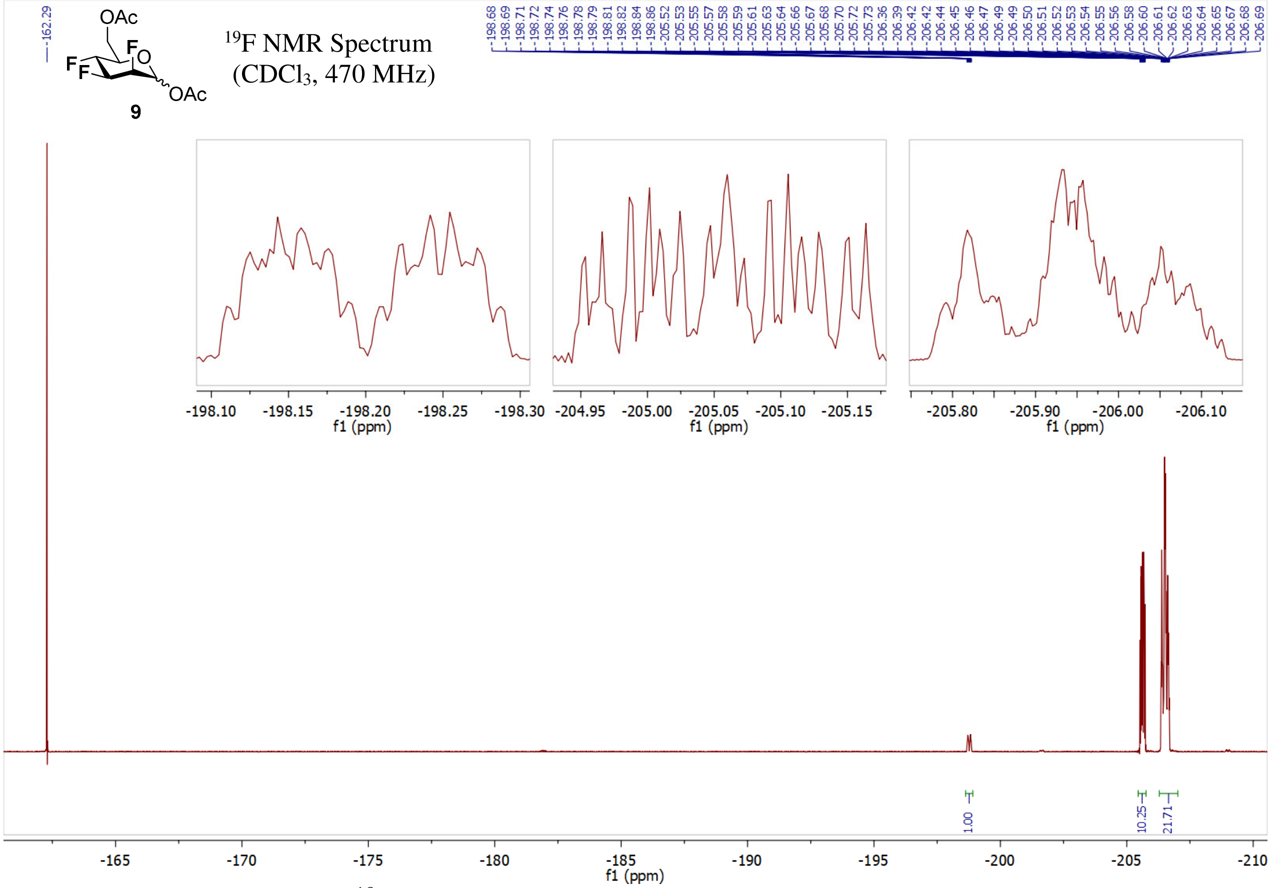

Supplementary Figure 107. ^{19}F NMR spectrum of compound **9**

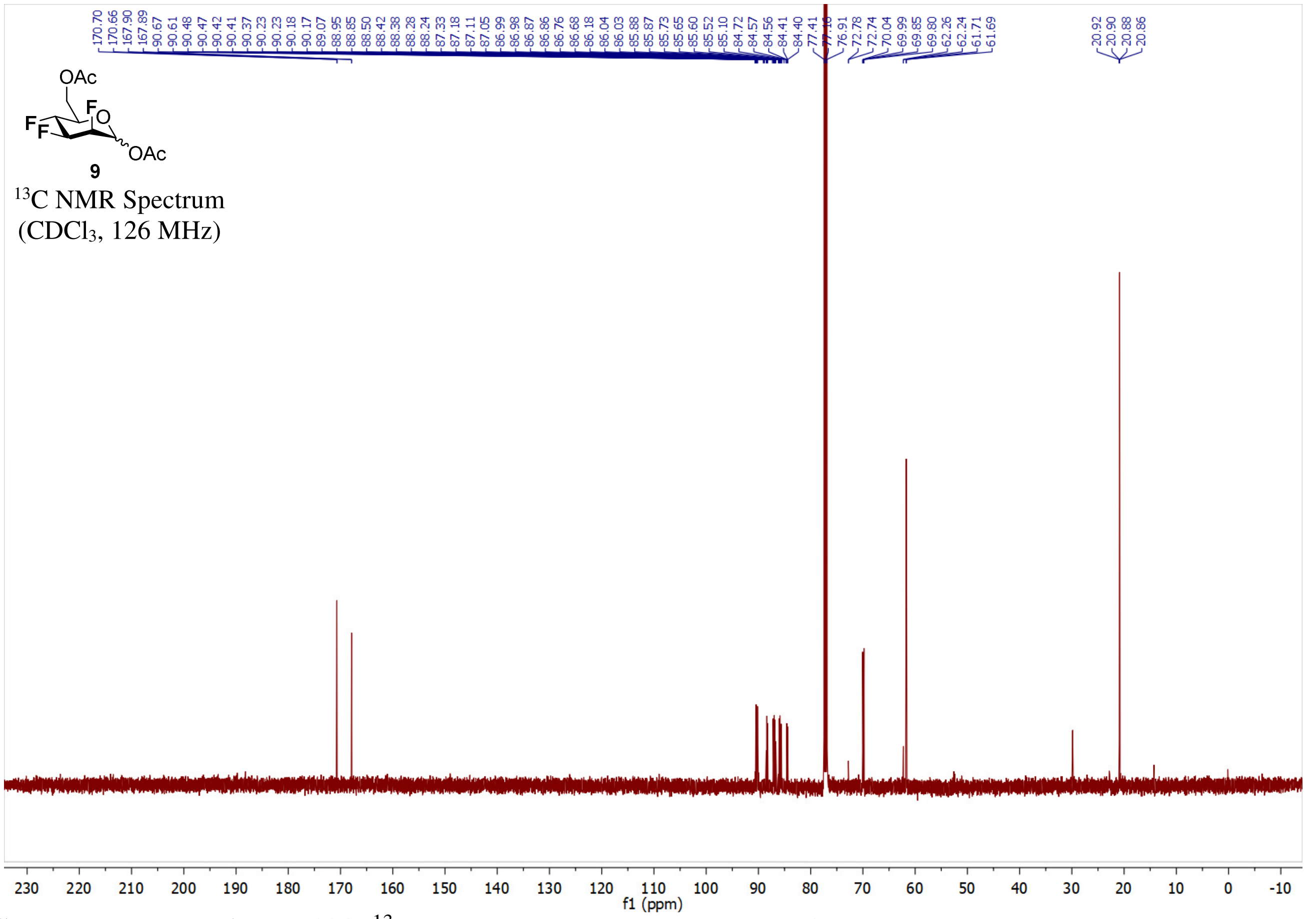

Supplementary Figure 108. ^{13}C NMR spectrum of compound **9**

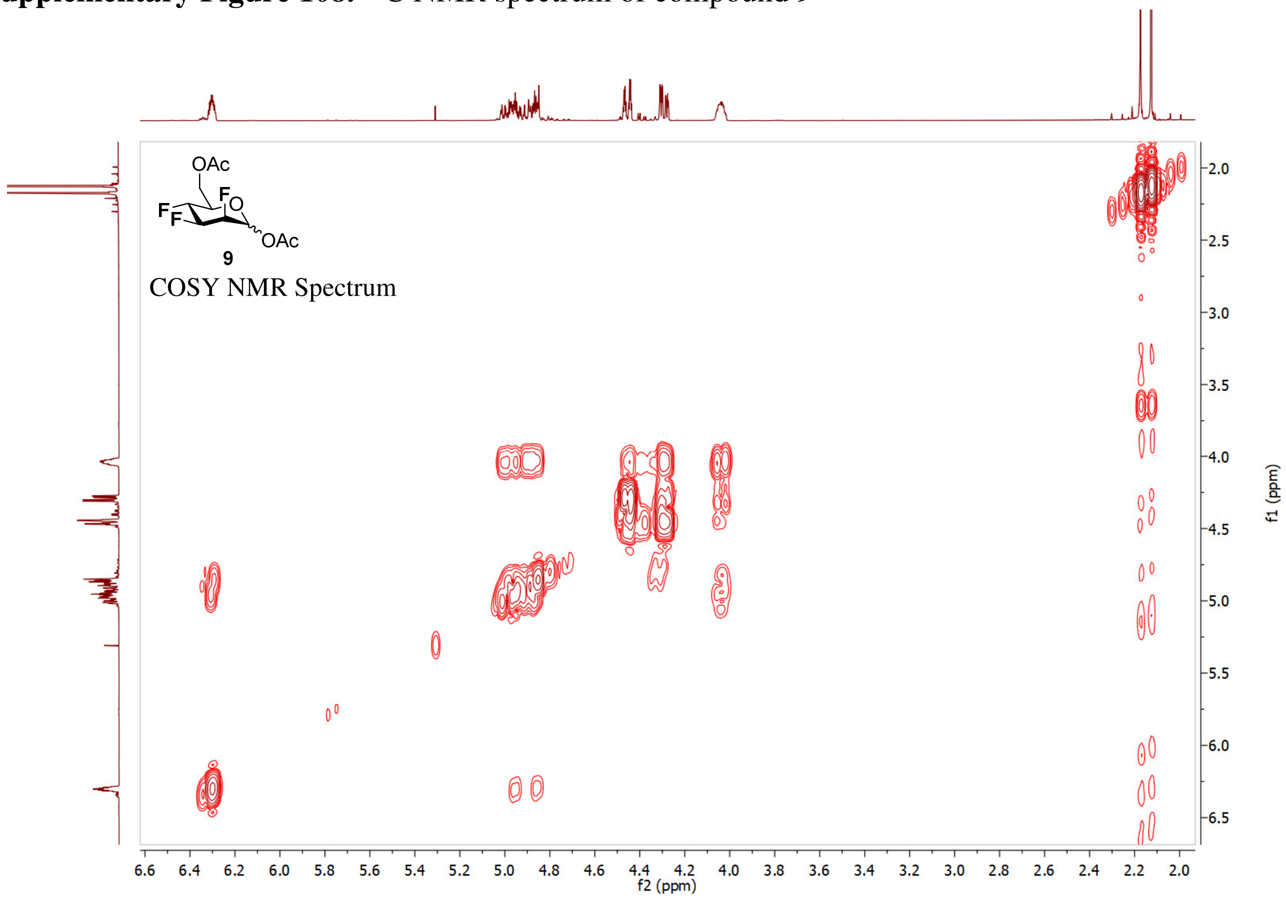

Supplementary Figure 109. COSY NMR spectrum of compound **9**

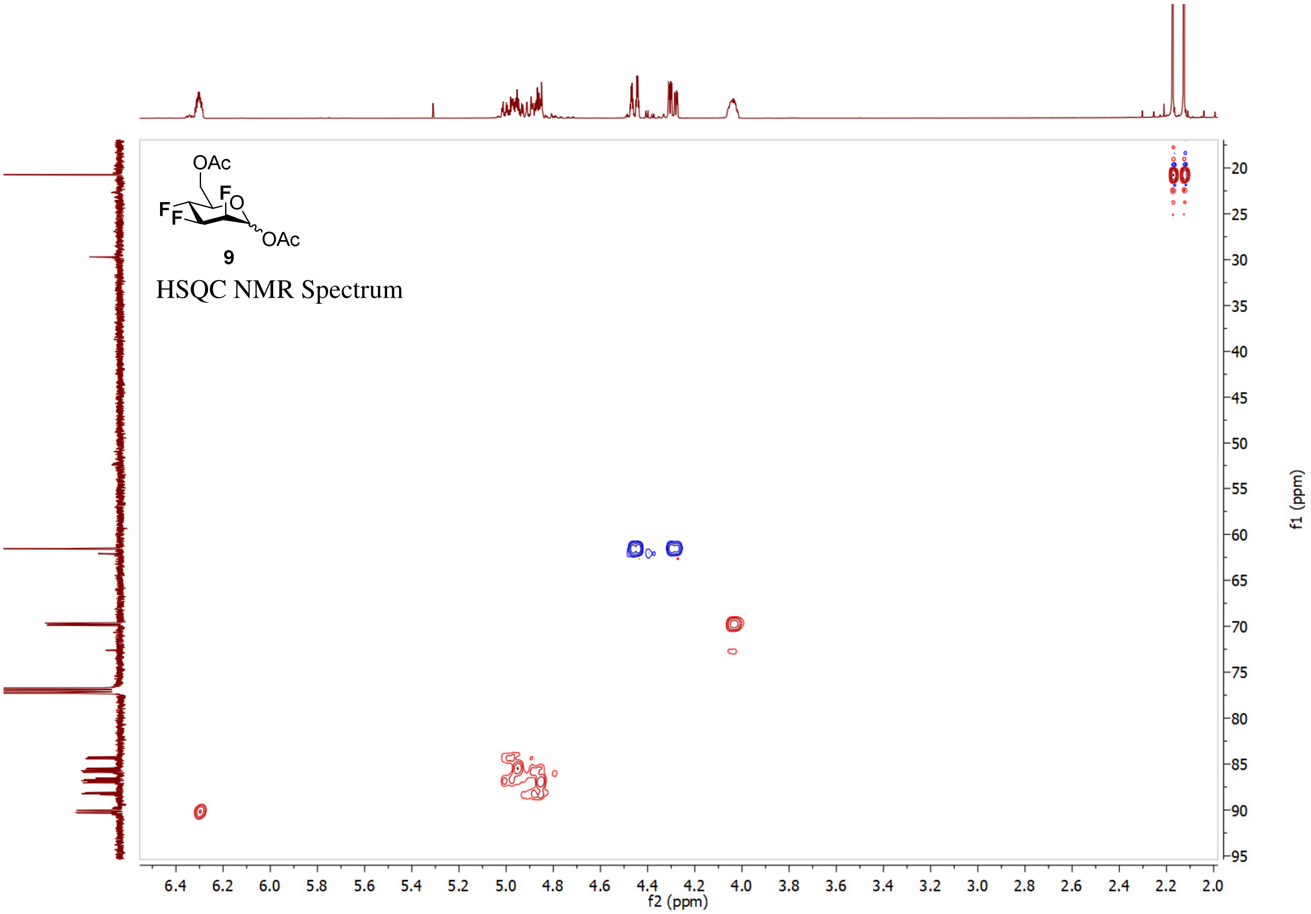

Supplementary Figure 110. HSQC NMR spectrum of compound **9**

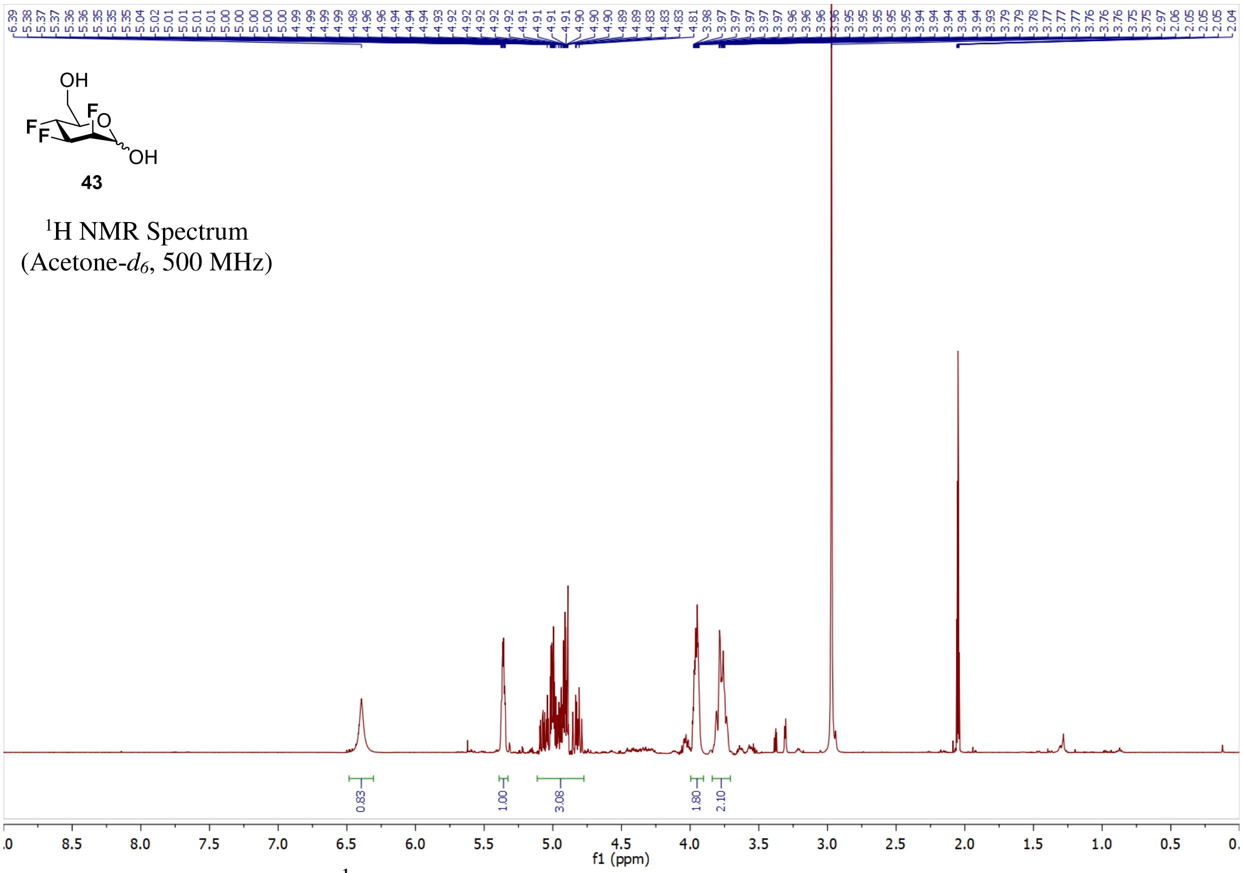

Supplementary Figure 111. ^{1}H NMR spectrum of compound **43**

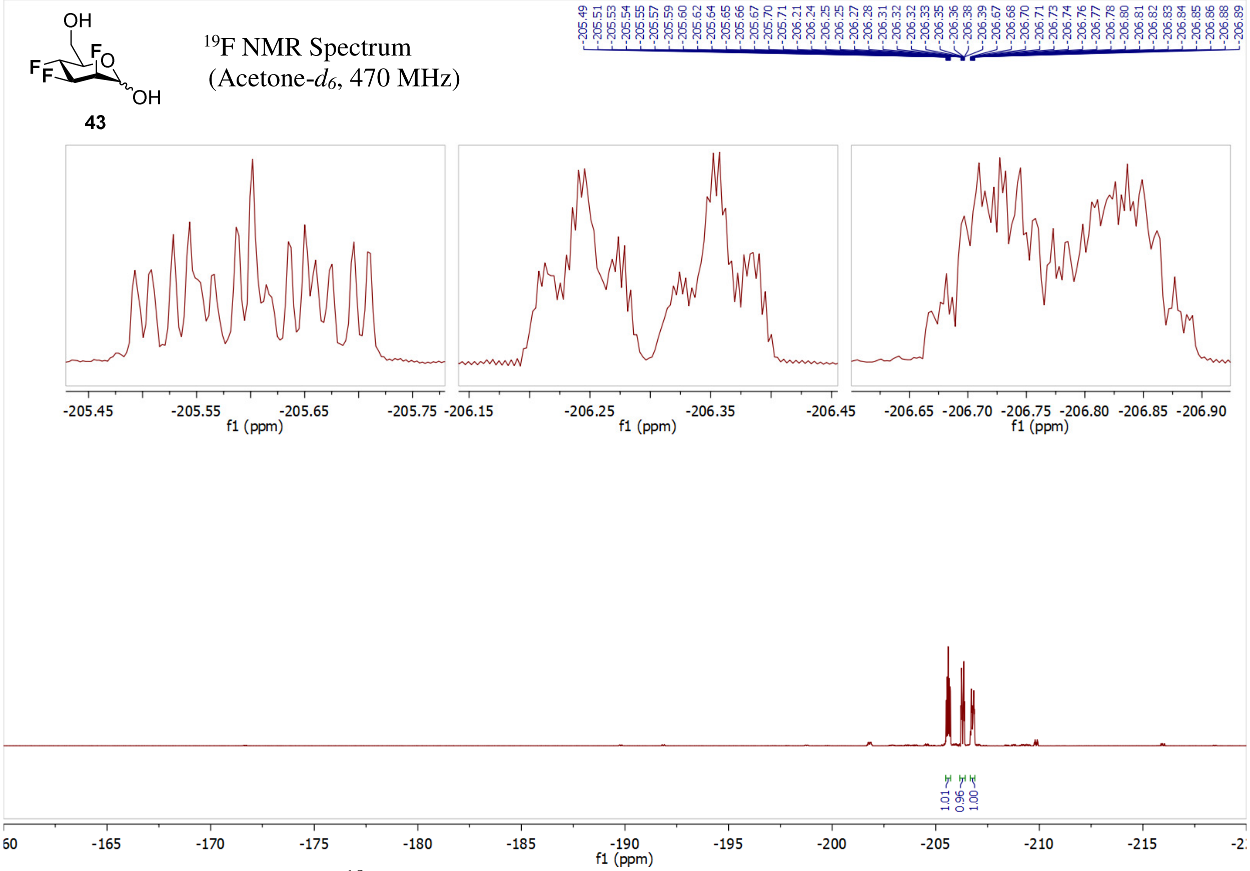

Supplementary Figure 112. ^{19}F NMR spectrum of compound **43**

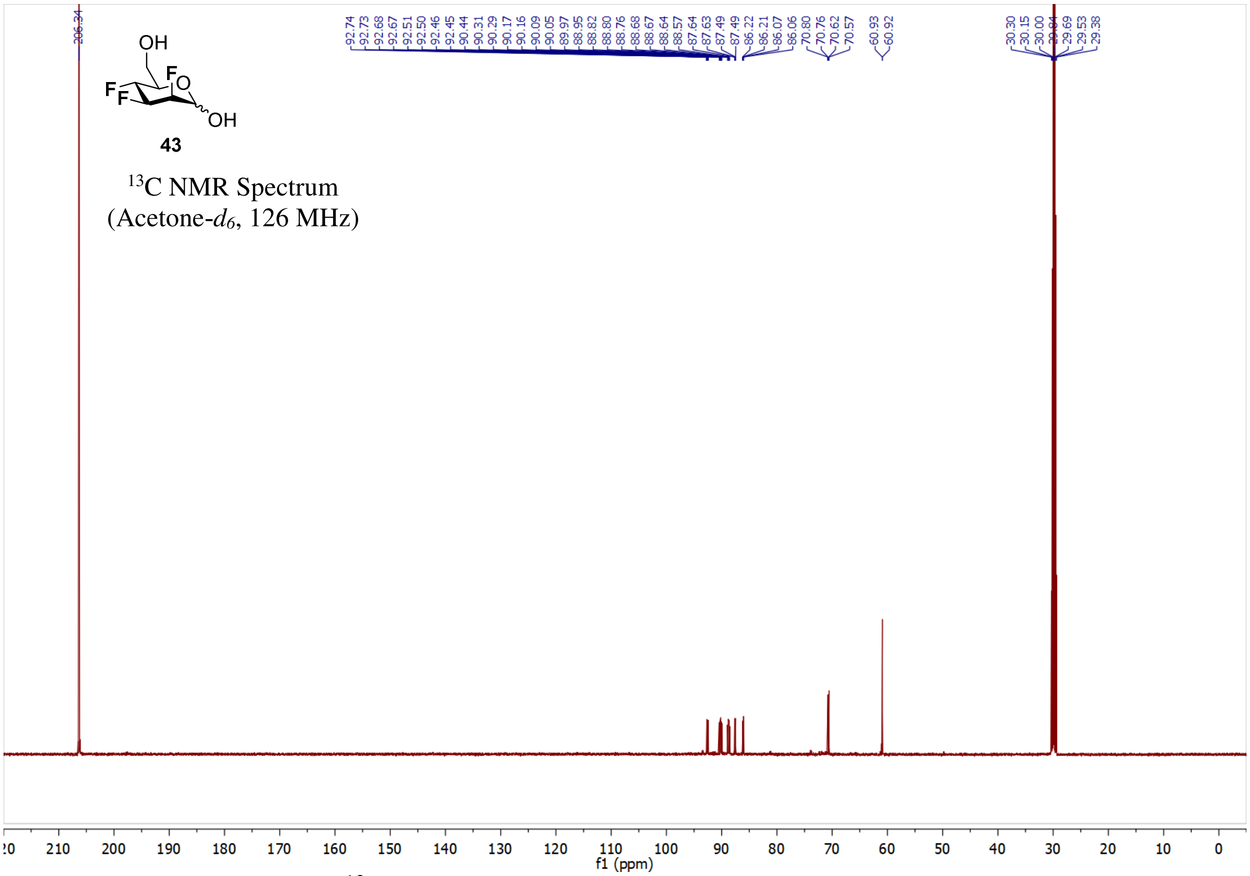

Supplementary Figure 113. ^{13}C NMR spectrum of compound **43**

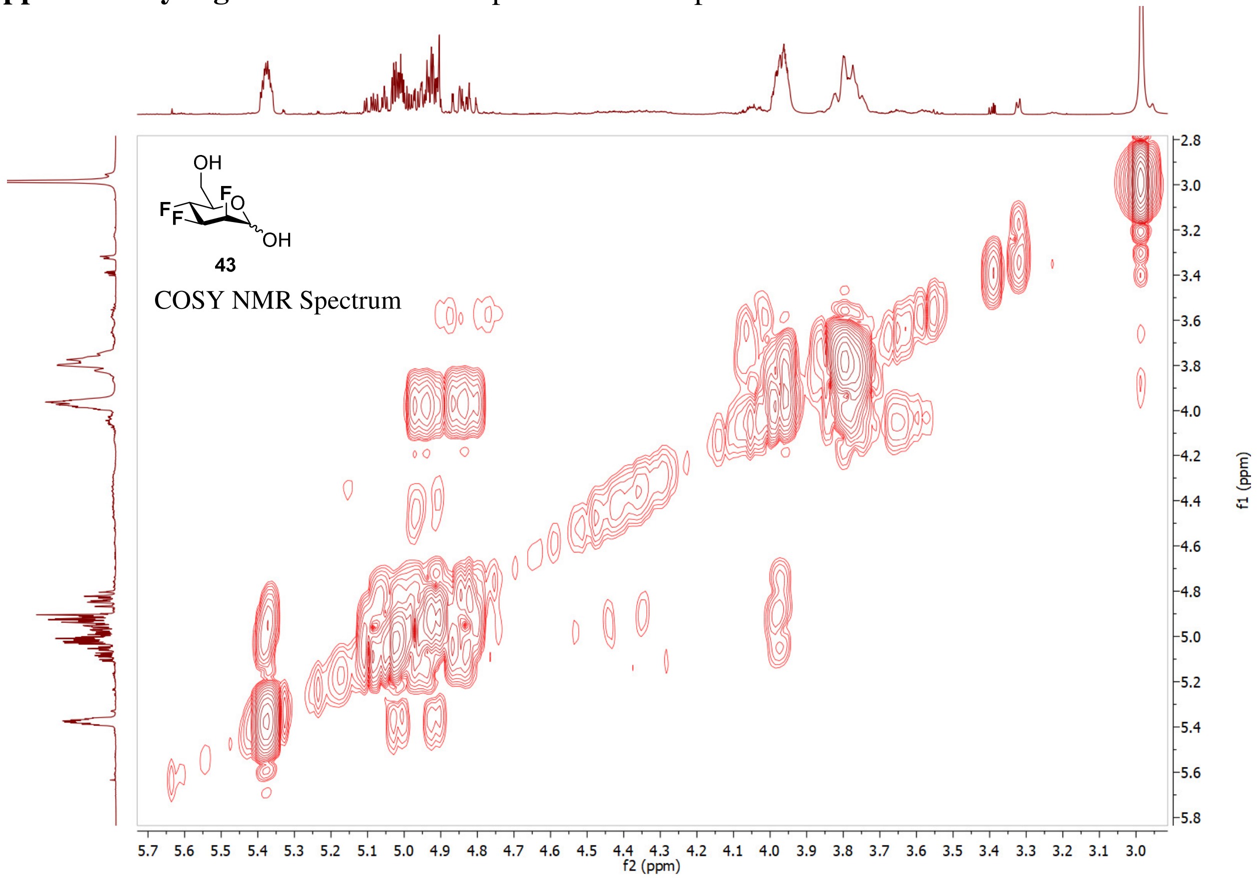

Supplementary Figure 114. COSY NMR spectrum of compound **43**

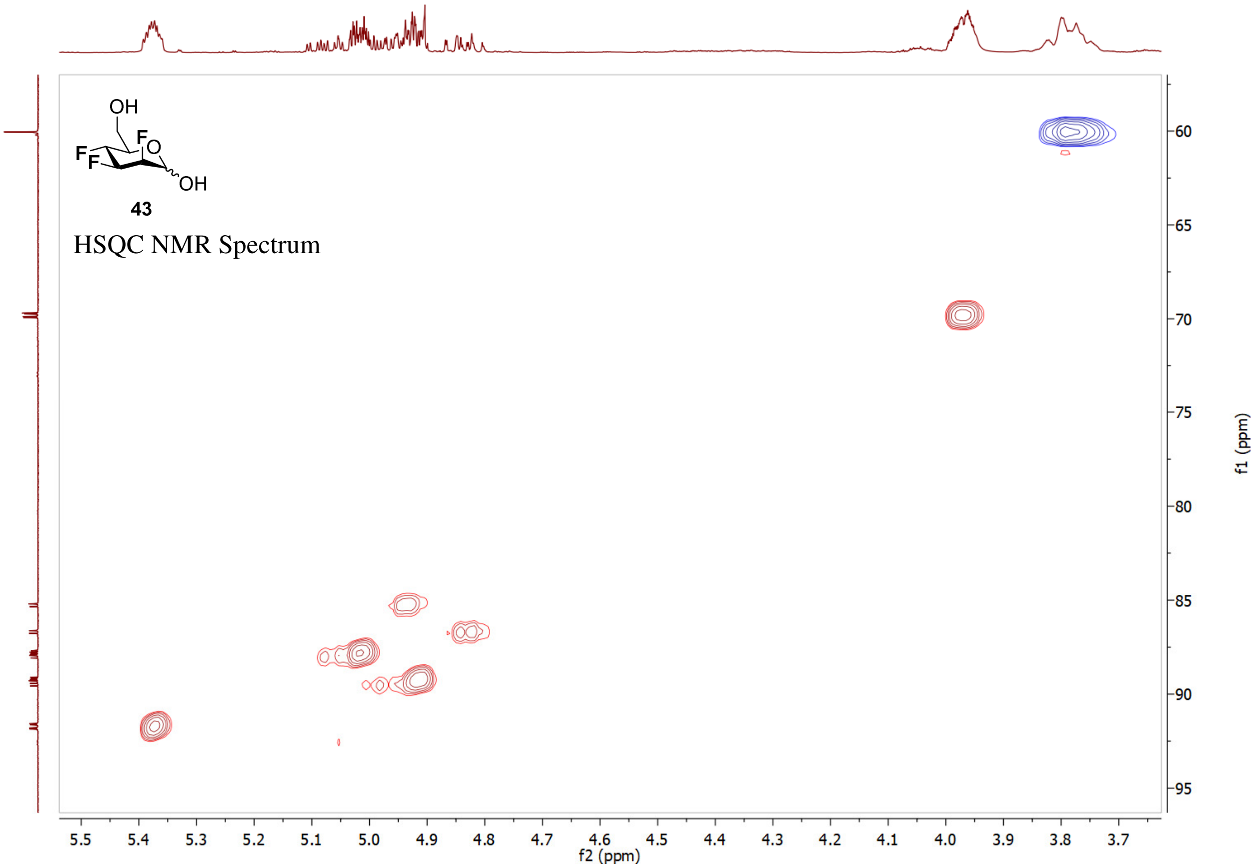

Supplementary Figure 115. HSQC NMR spectrum of compound **43**

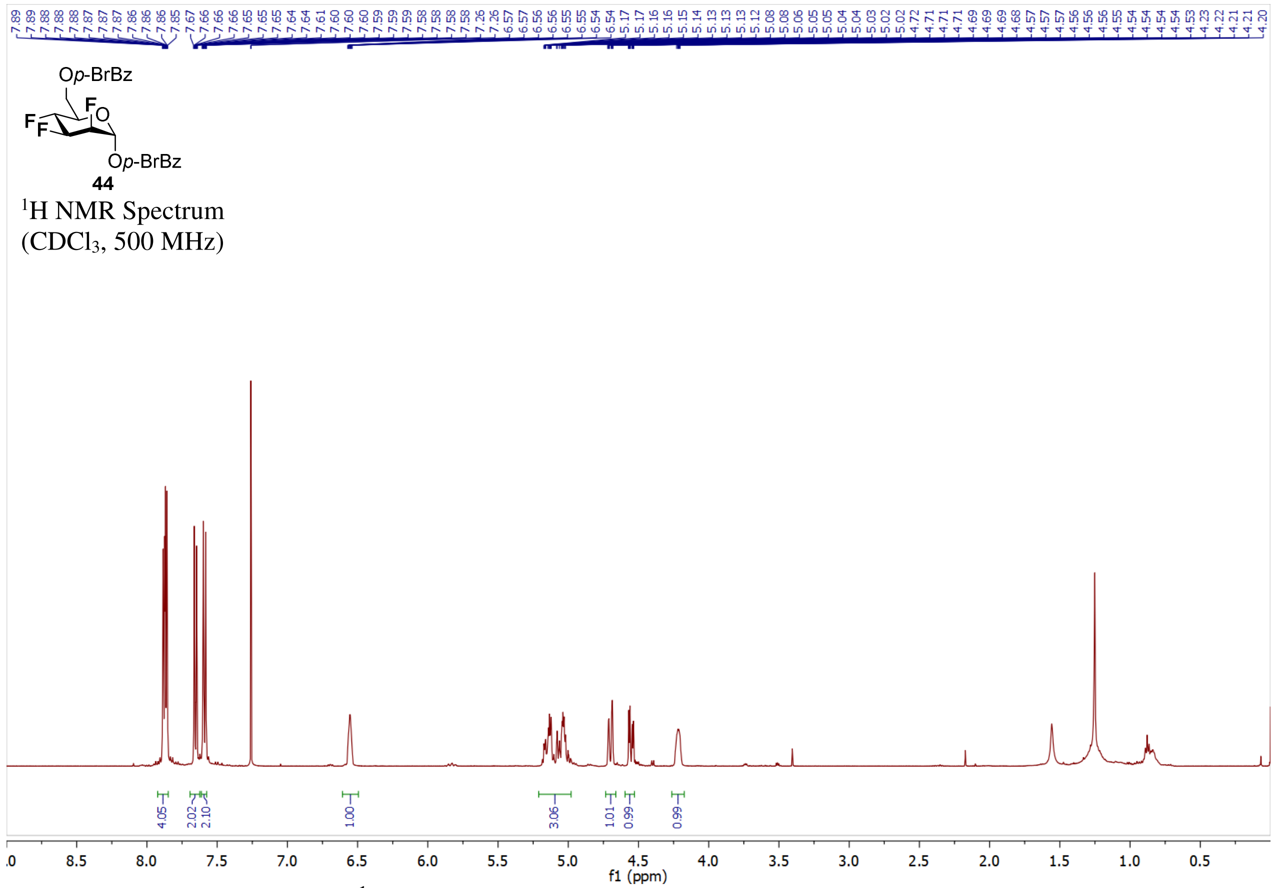

Supplementary Figure 116. ^{1}H NMR spectrum of compound **44**

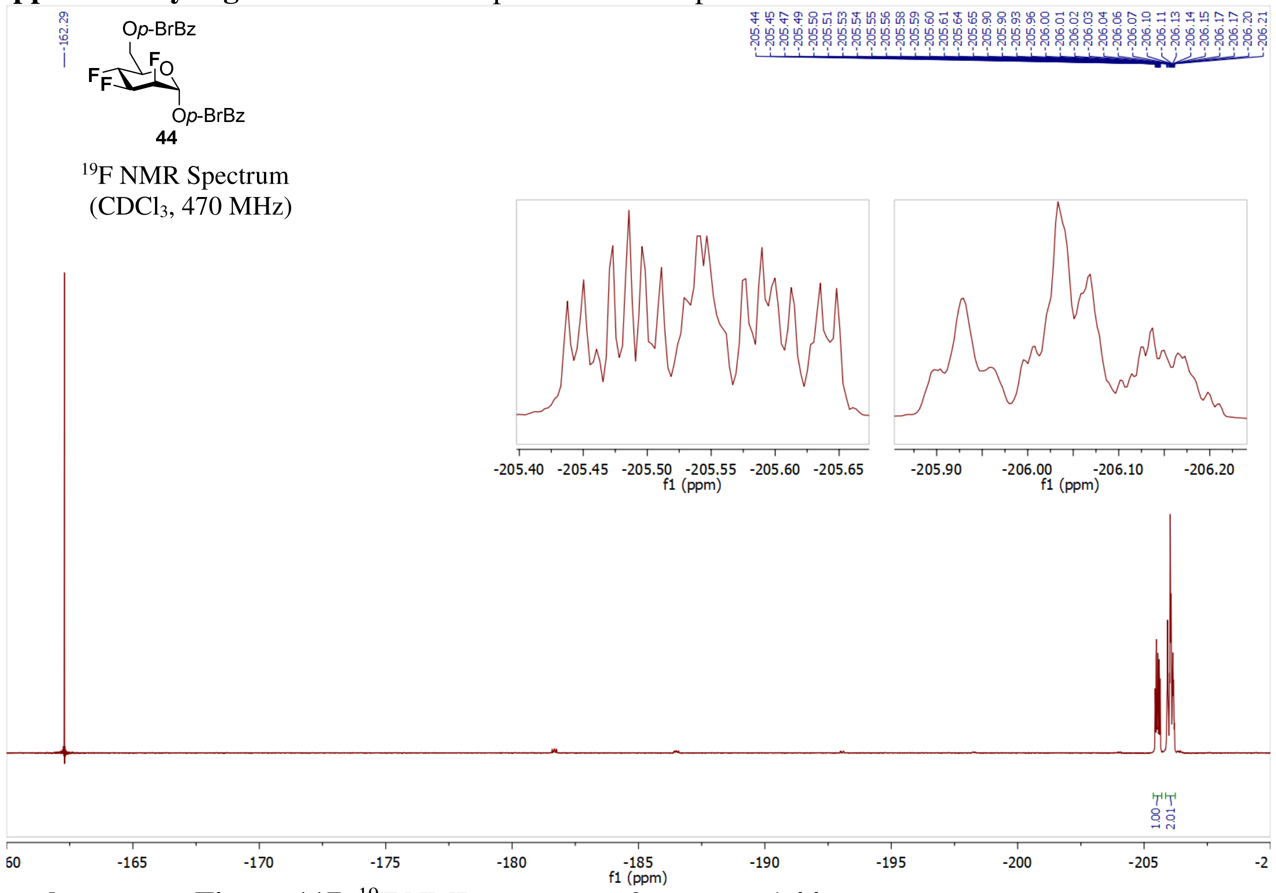

Supplementary Figure 117. ^{19}F NMR spectrum of compound **44**

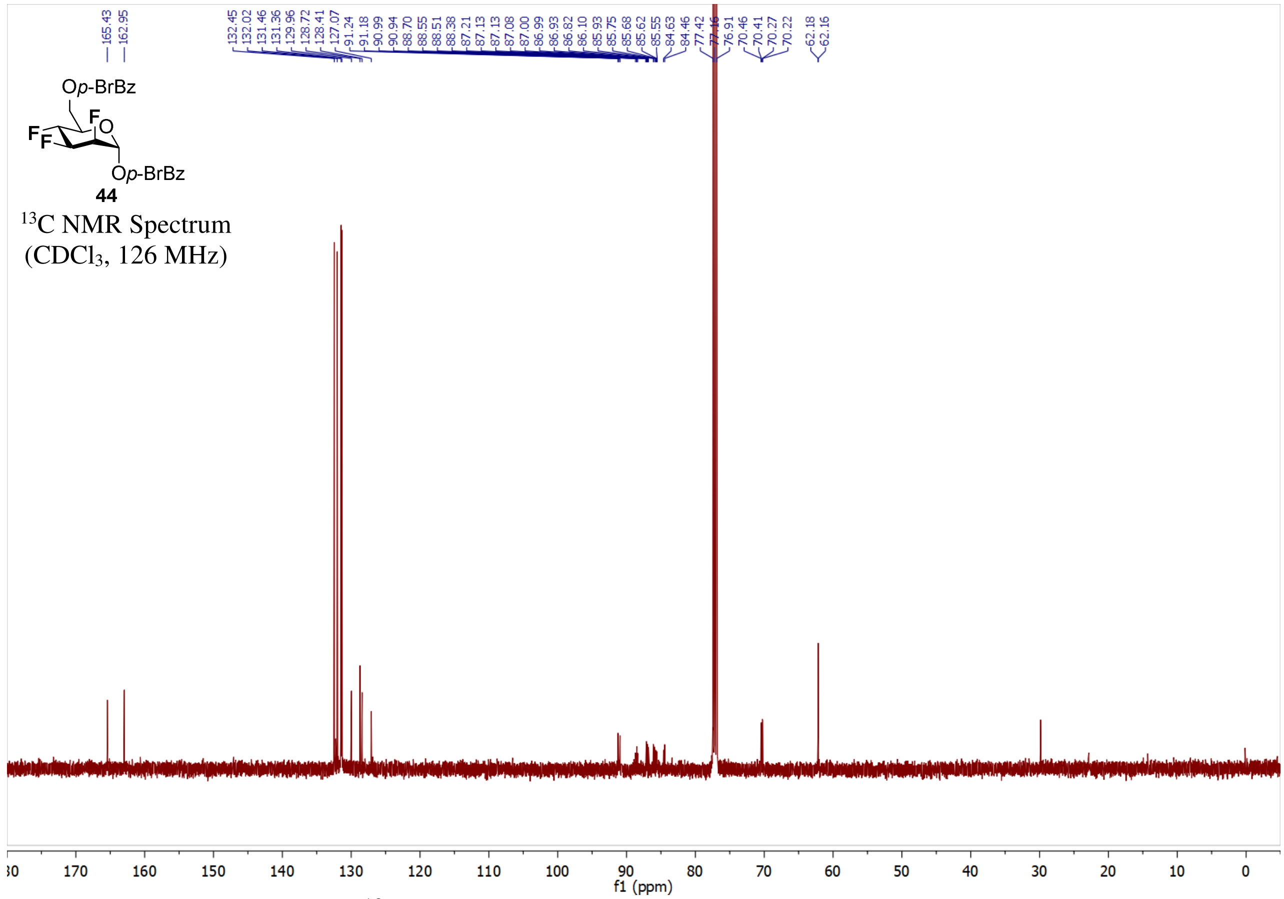

Supplementary Figure 118. ^{13}C NMR spectrum of compound **44**

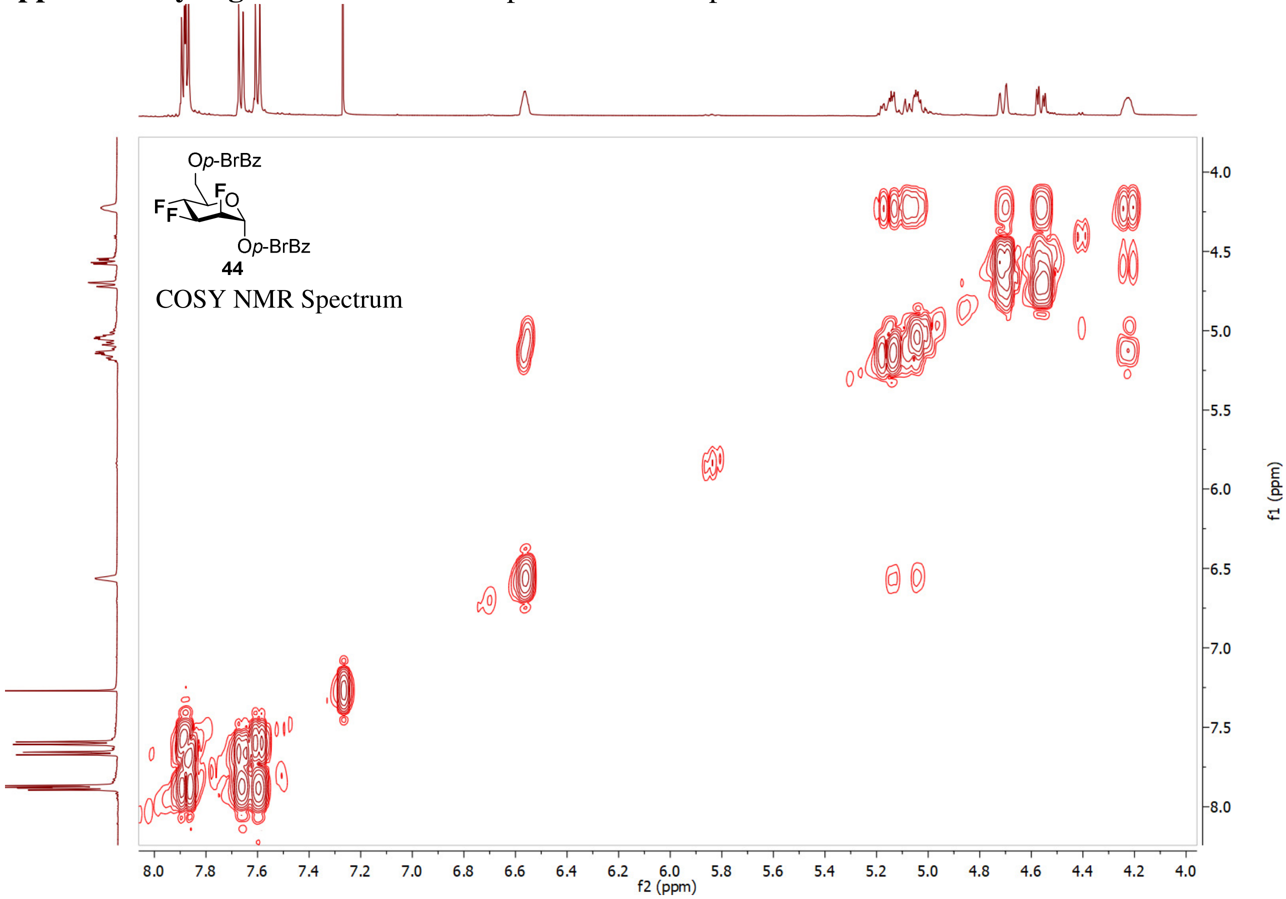

Supplementary Figure 119. COSY NMR spectrum of compound **44**

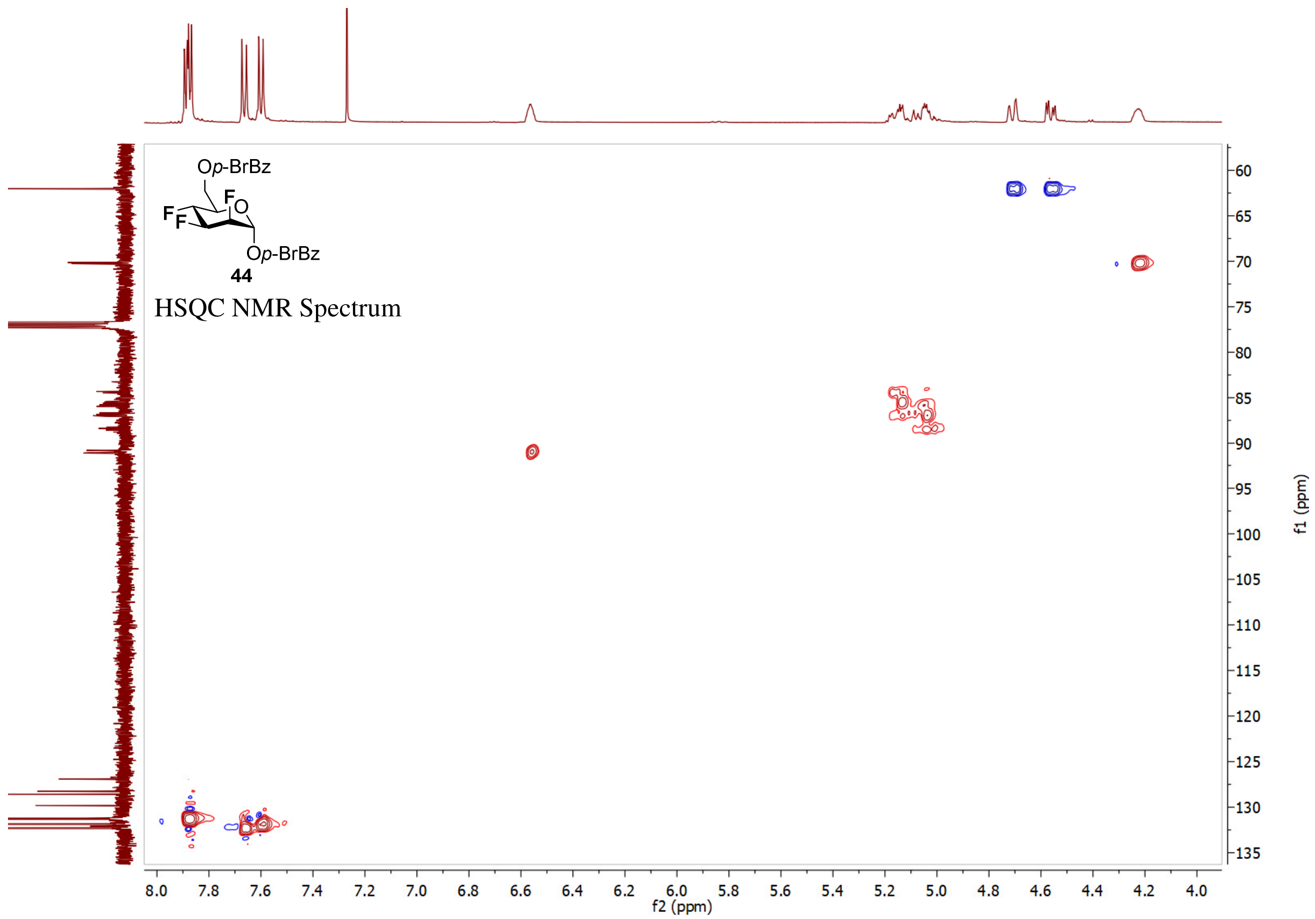

Supplementary Figure 120. HSQC NMR spectrum of compound **44**

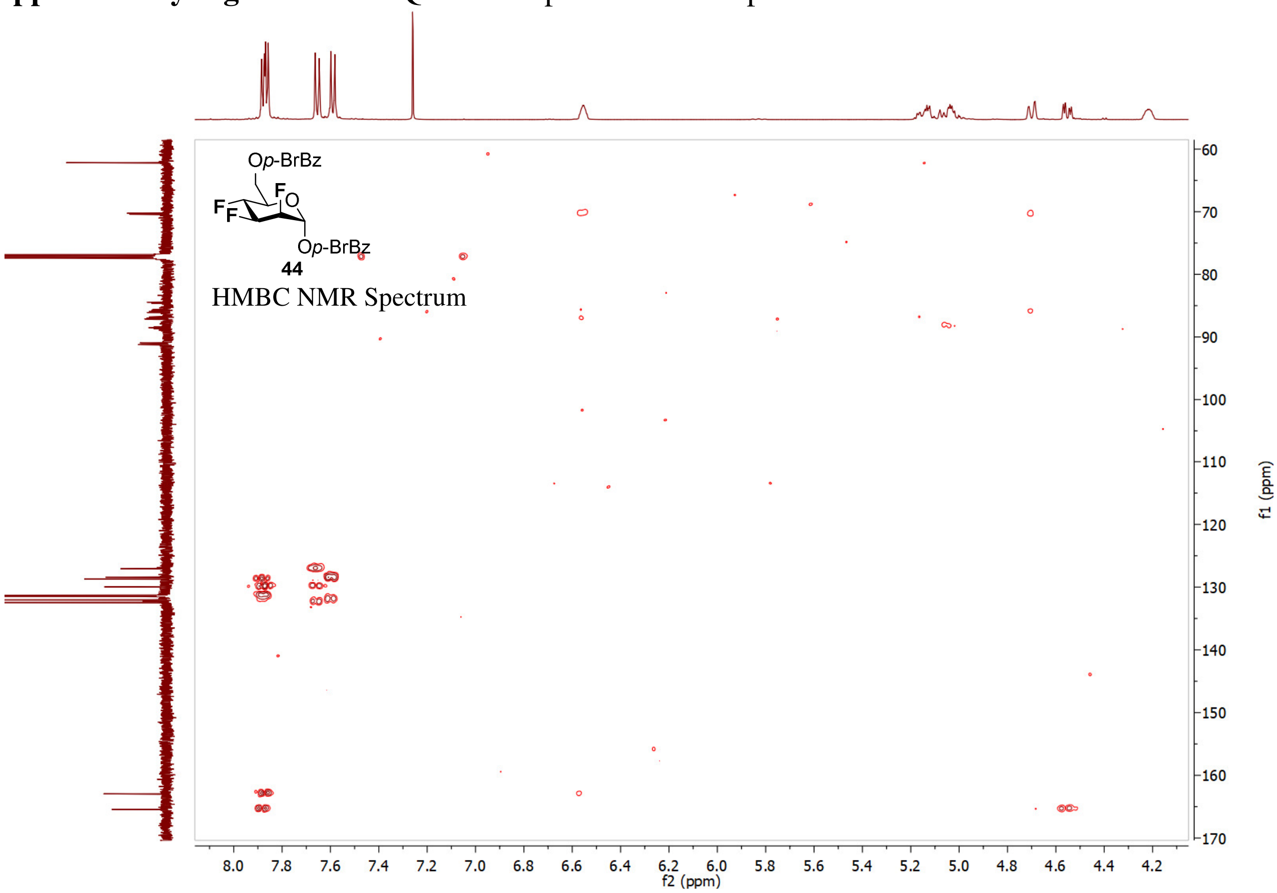

Supplementary Figure 121. HMBC NMR spectrum of compound **44**

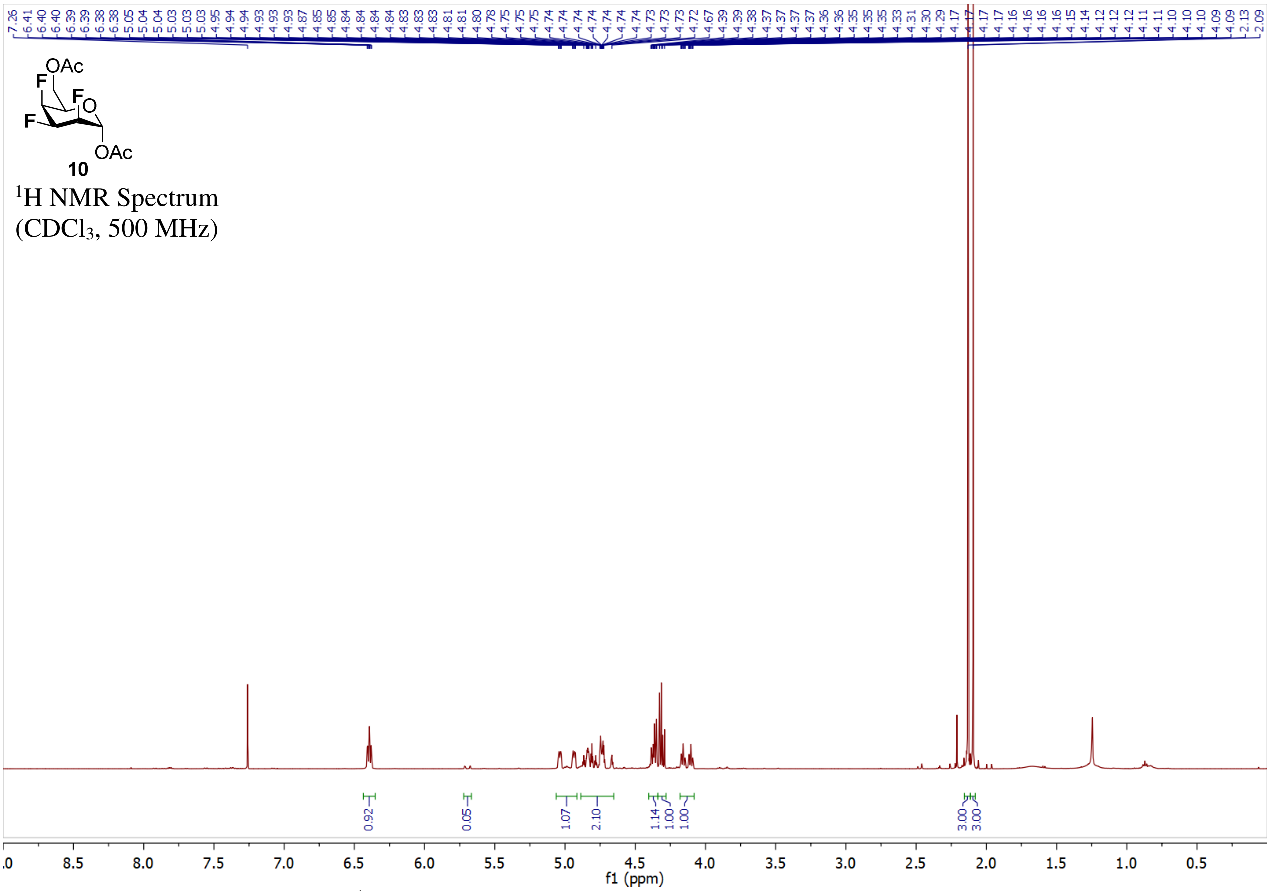

Supplementary Figure 122. ^{1}H NMR spectrum of compound **10**

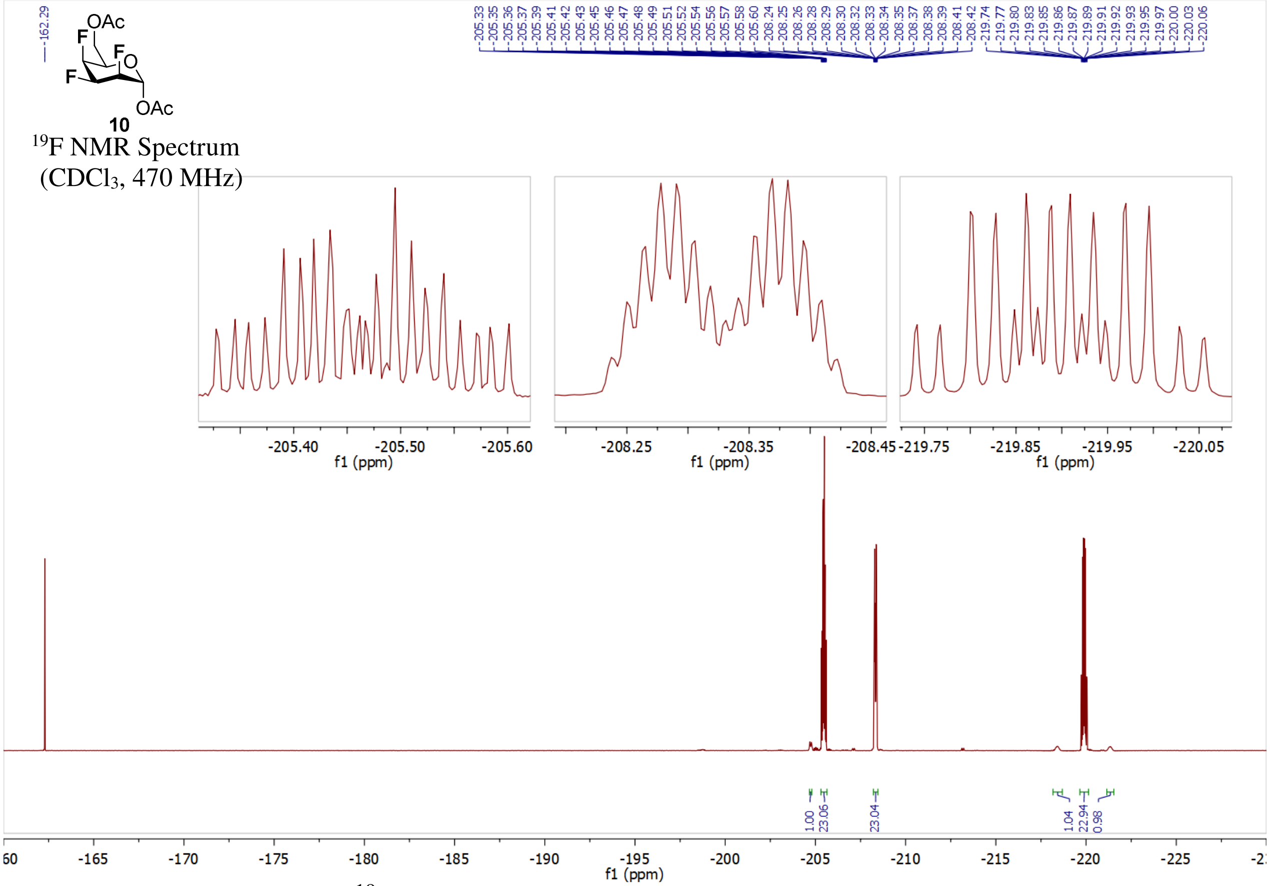

Supplementary Figure 123. ^{19}F NMR spectrum of compound **10**

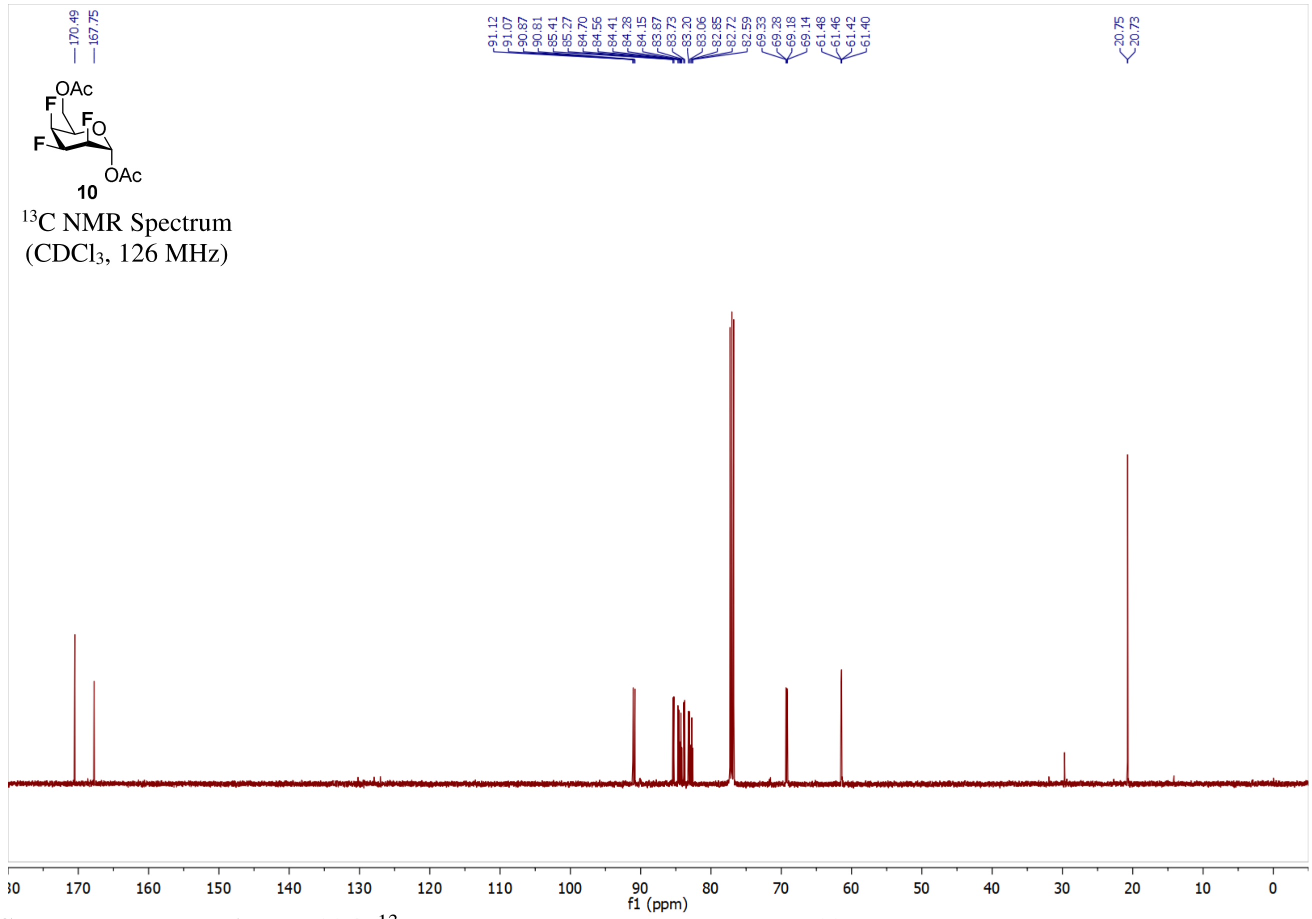

Supplementary Figure 124. ^{13}C NMR spectrum of compound **10**

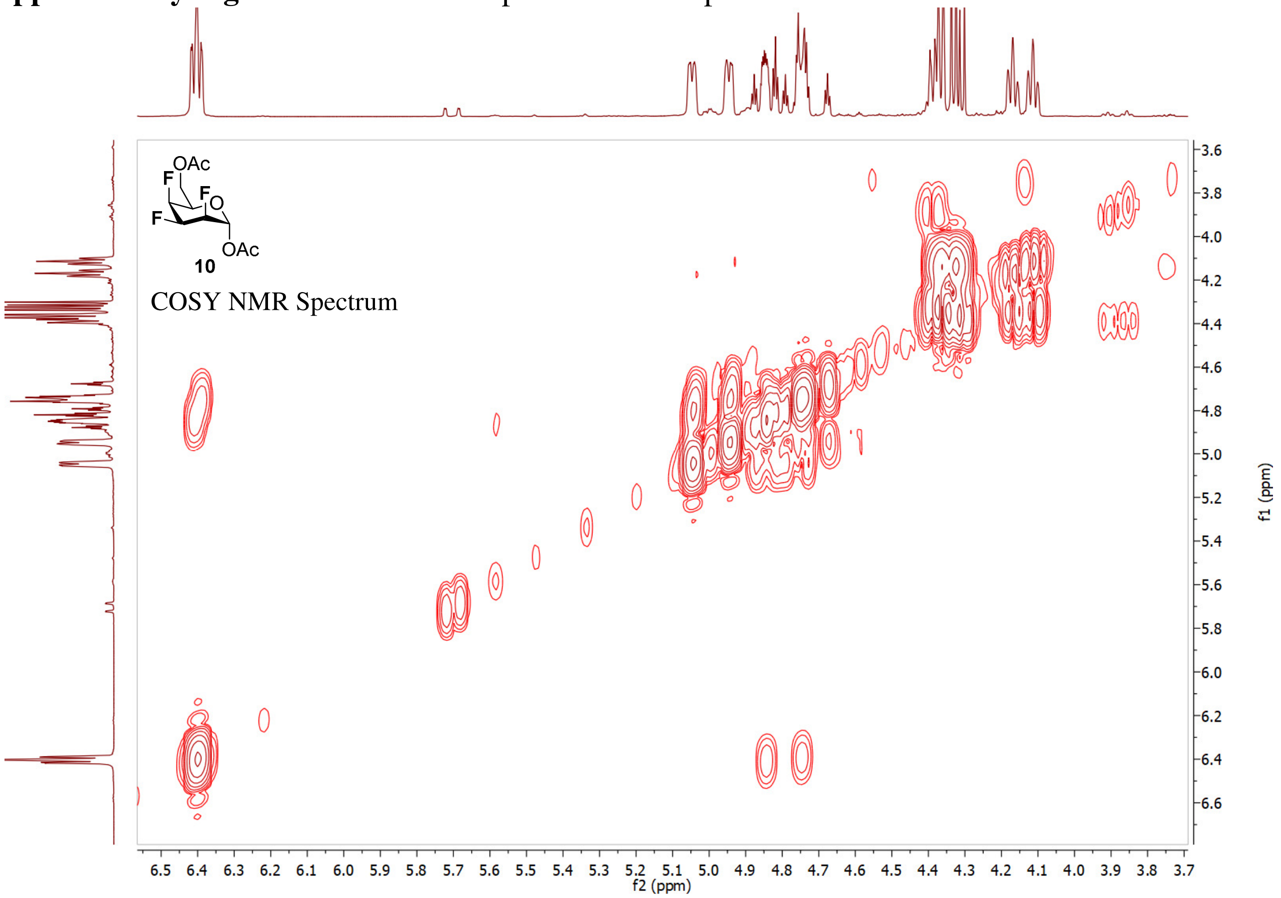

Supplementary Figure 125. COSY NMR spectrum of compound **10**

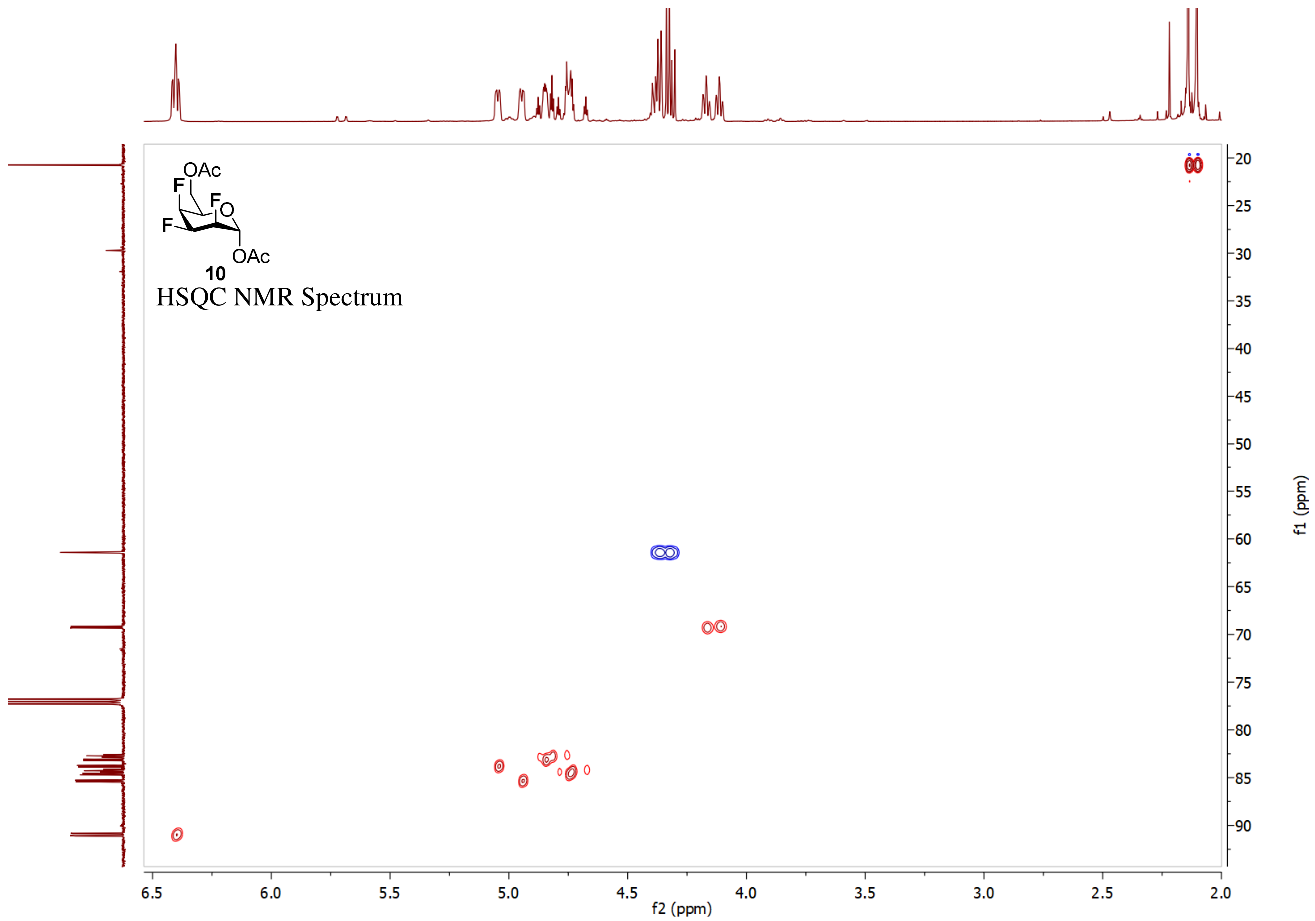

Supplementary Figure 126. HSQC NMR spectrum of compound **10**

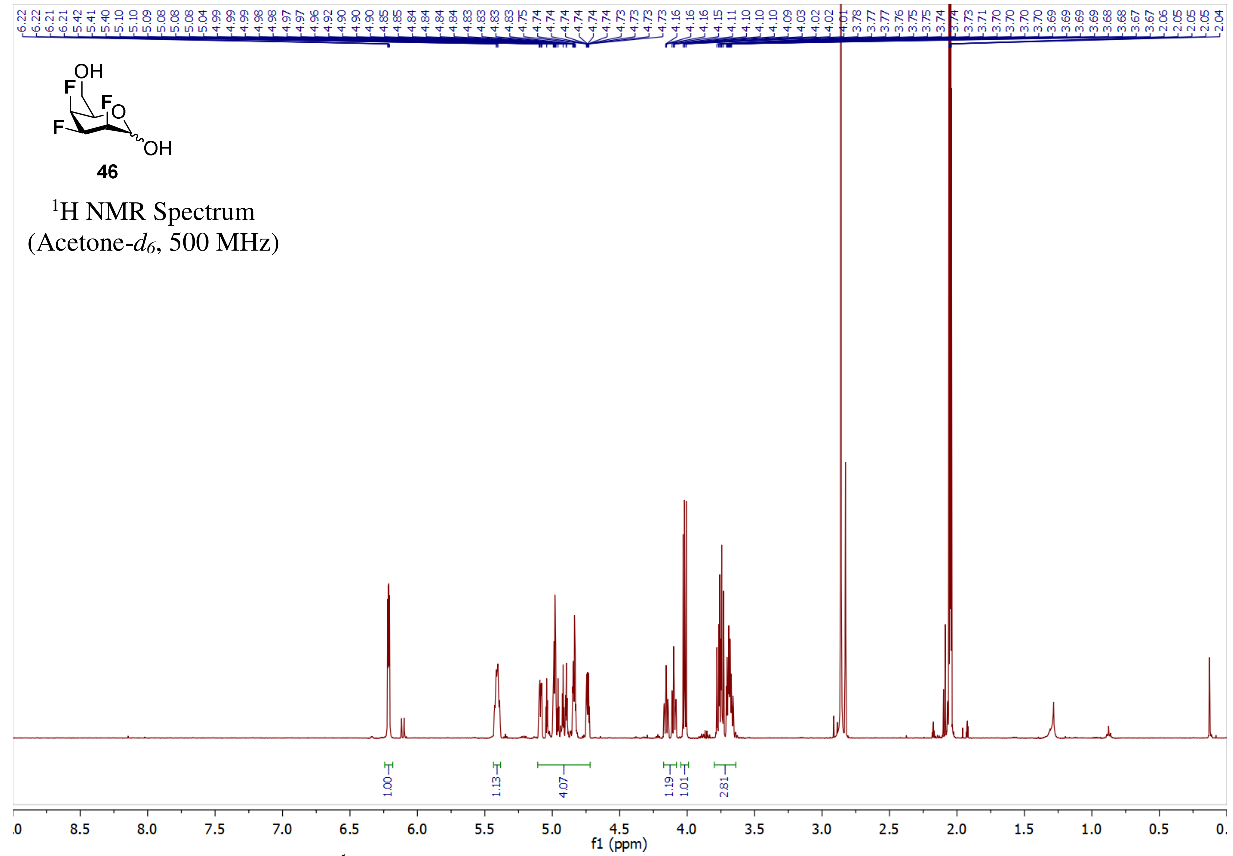

Supplementary Figure 127. ^{1}H NMR spectrum of compound **46**

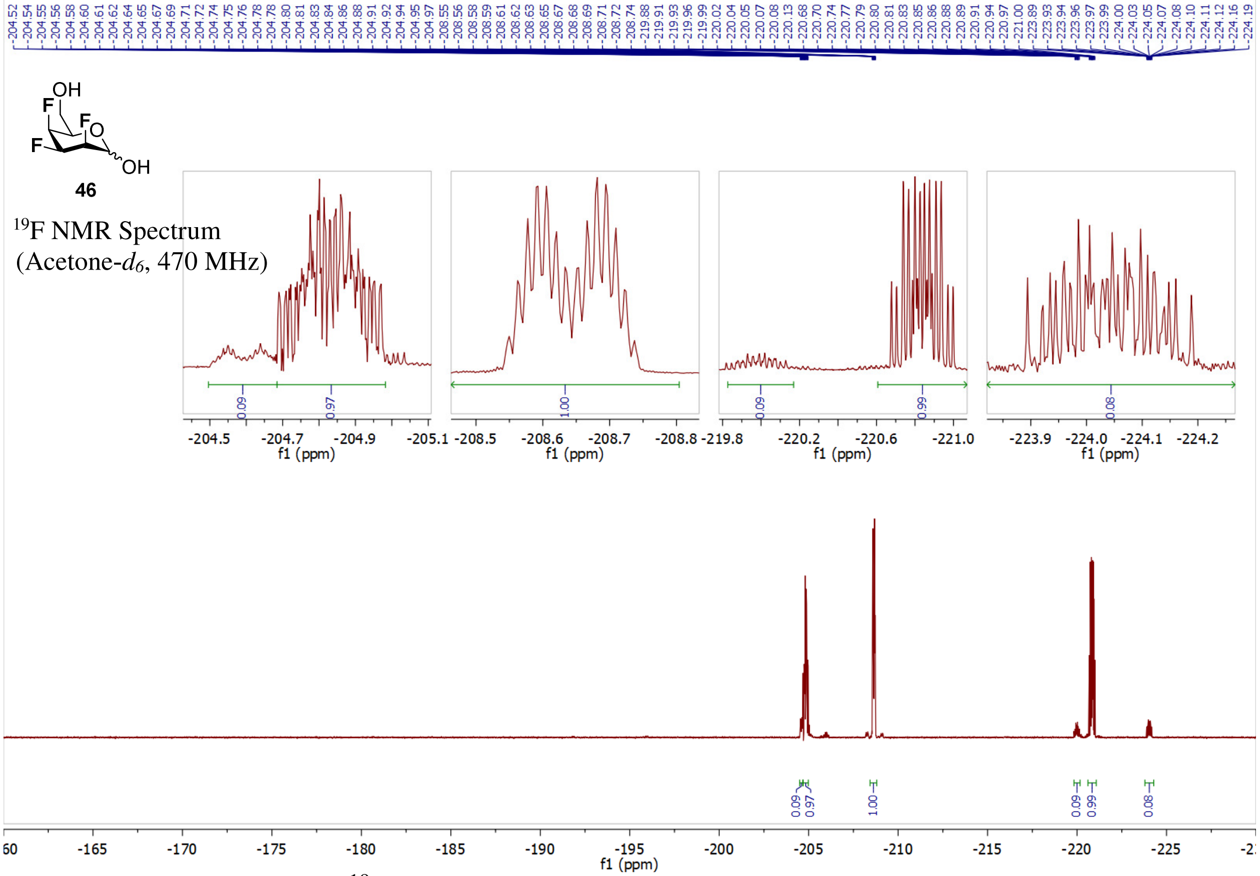

Supplementary Figure 128. ^{19}F NMR spectrum of compound **46**

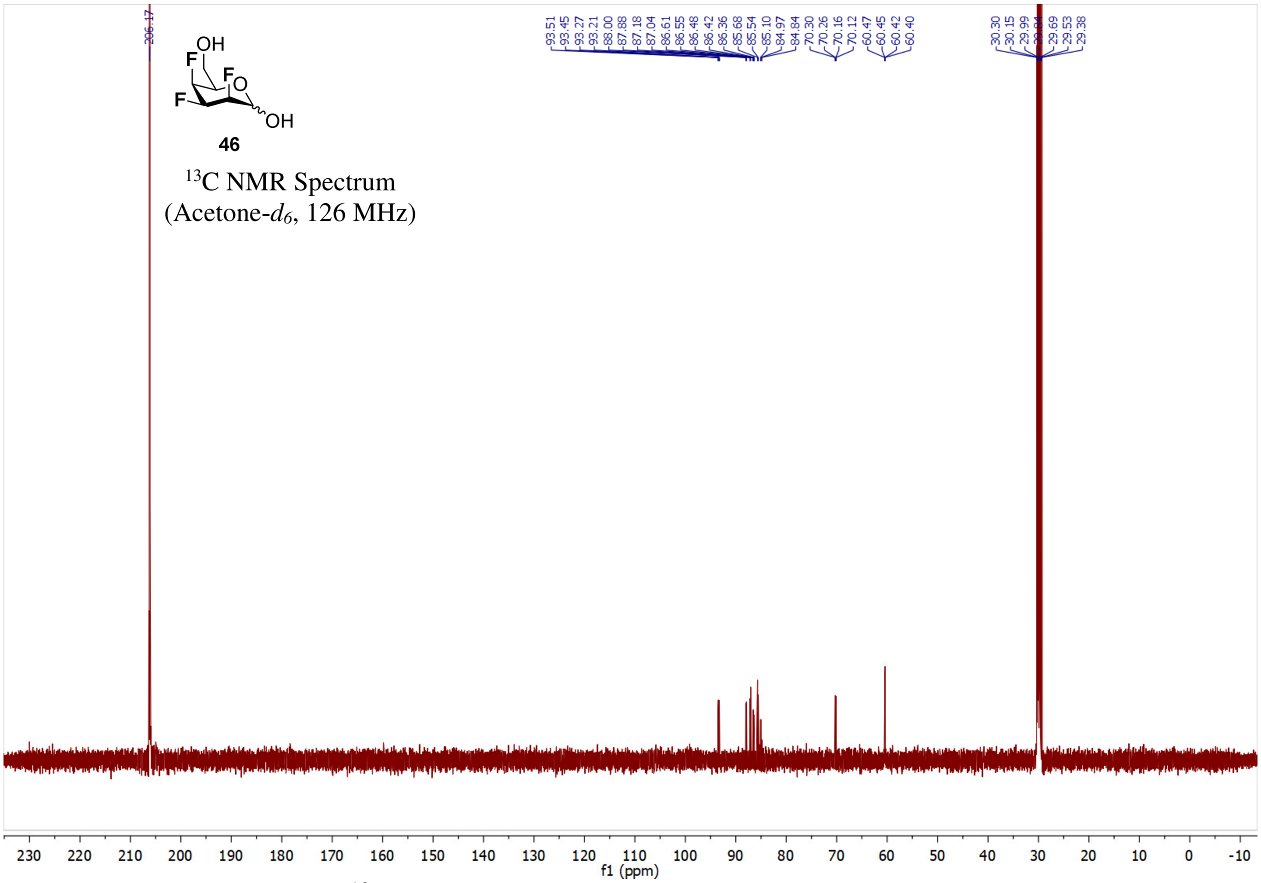

Supplementary Figure 129. ^{13}C NMR spectrum of compound **46**

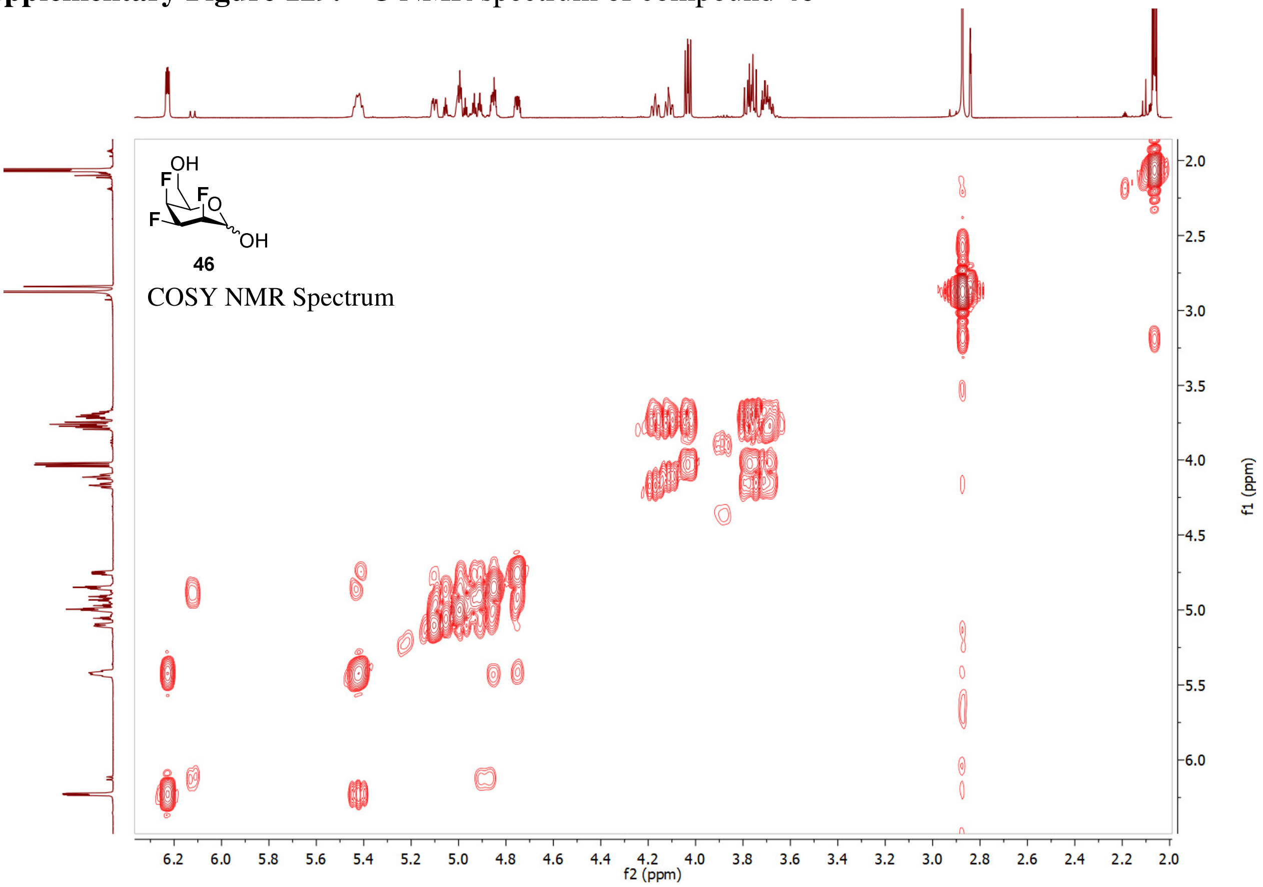

Supplementary Figure 130. COSY NMR spectrum of compound **46**

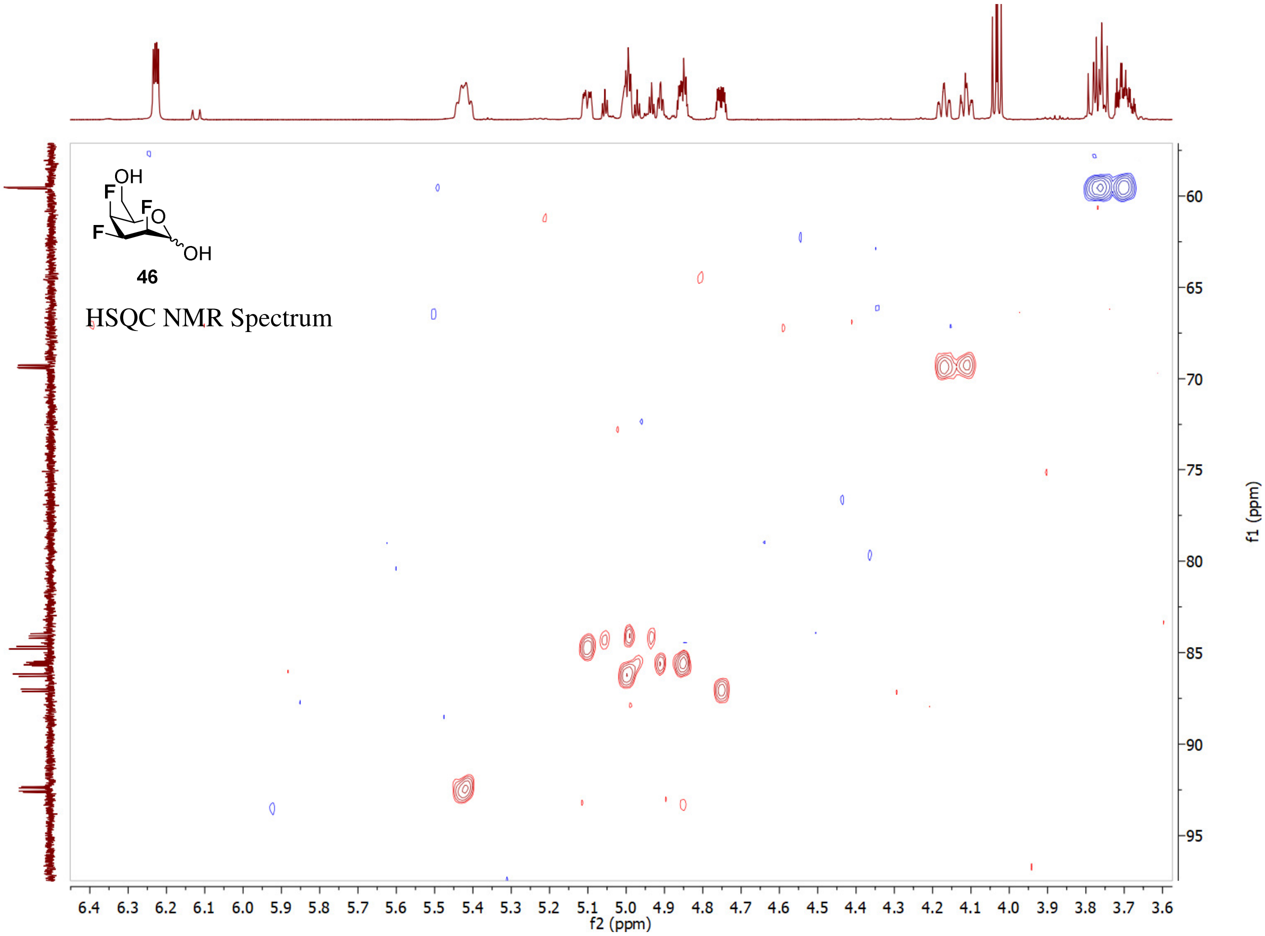

Supplementary Figure 131. HSQC NMR spectrum of compound **46**

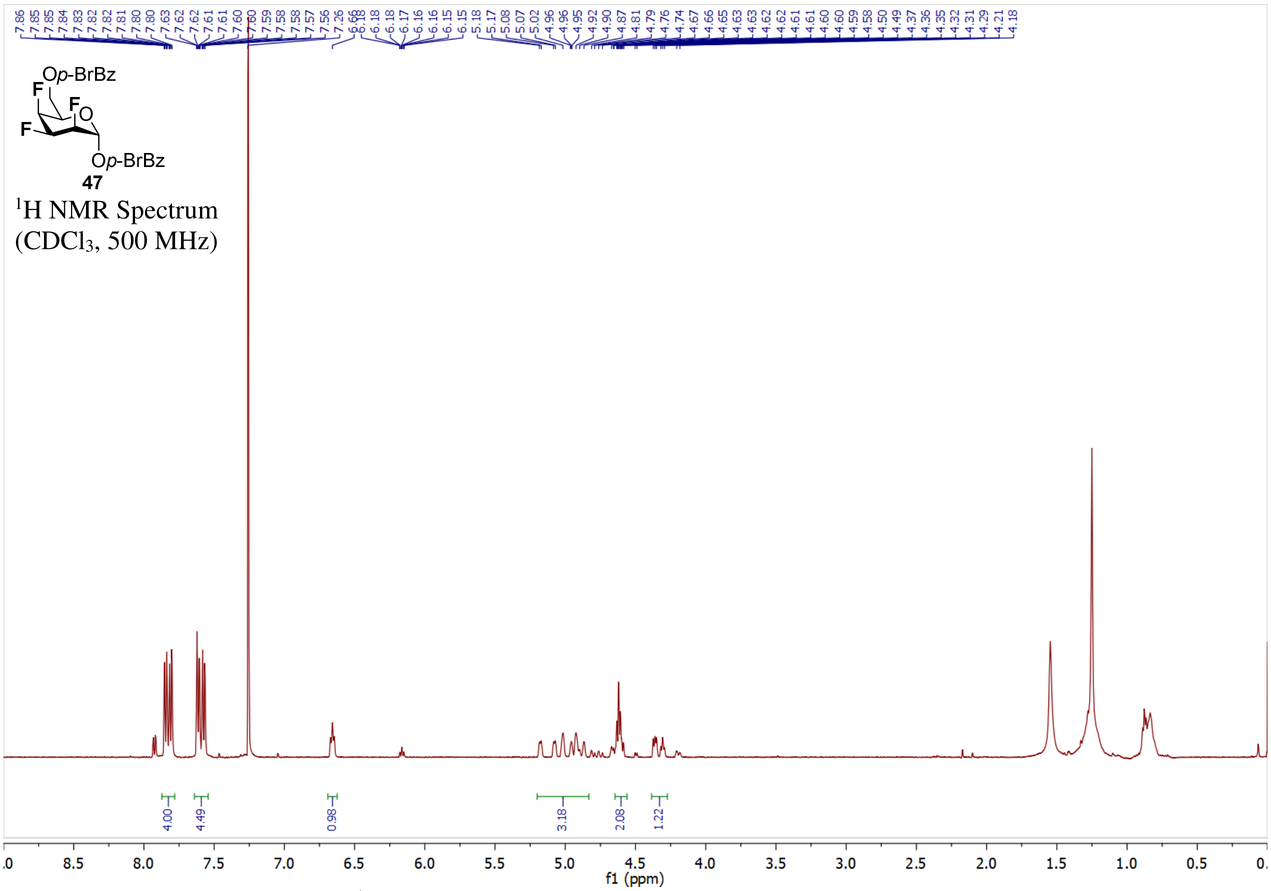

Supplementary Figure 132. ^{1}H NMR spectrum of compound **47**

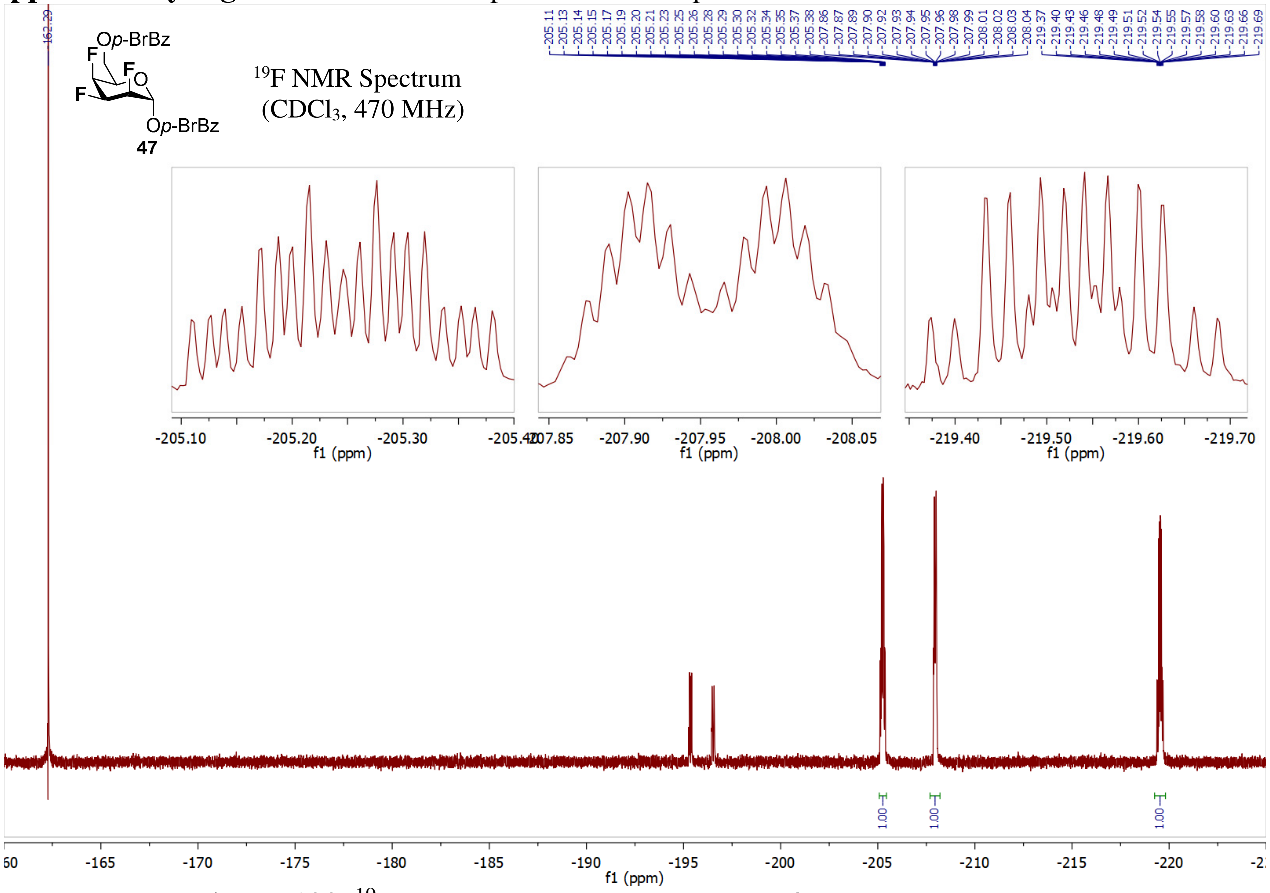

Supplementary Figure 133. ^{19}F NMR spectrum of compound **47**

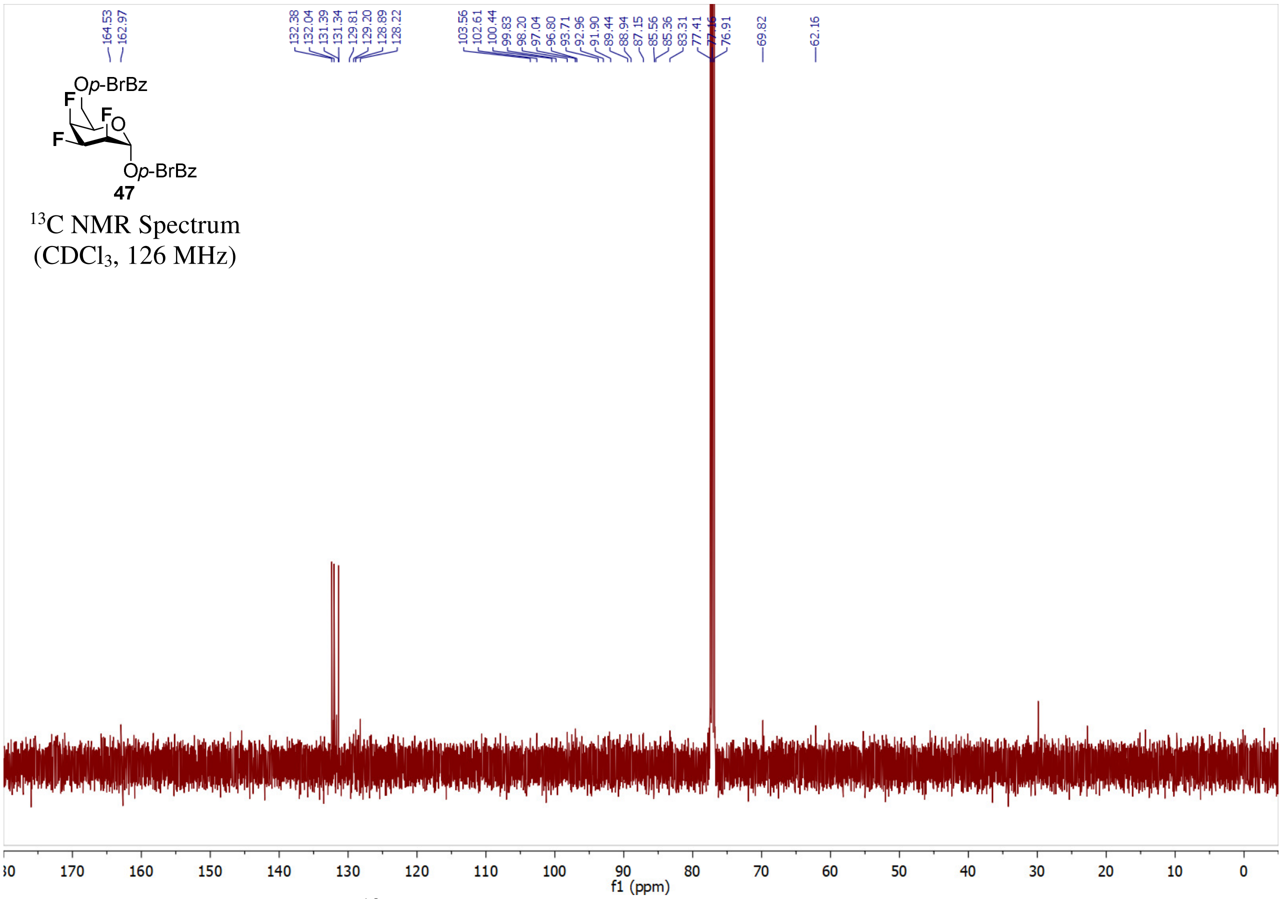

Supplementary Figure 134. ^{13}C NMR spectrum of compound **47**

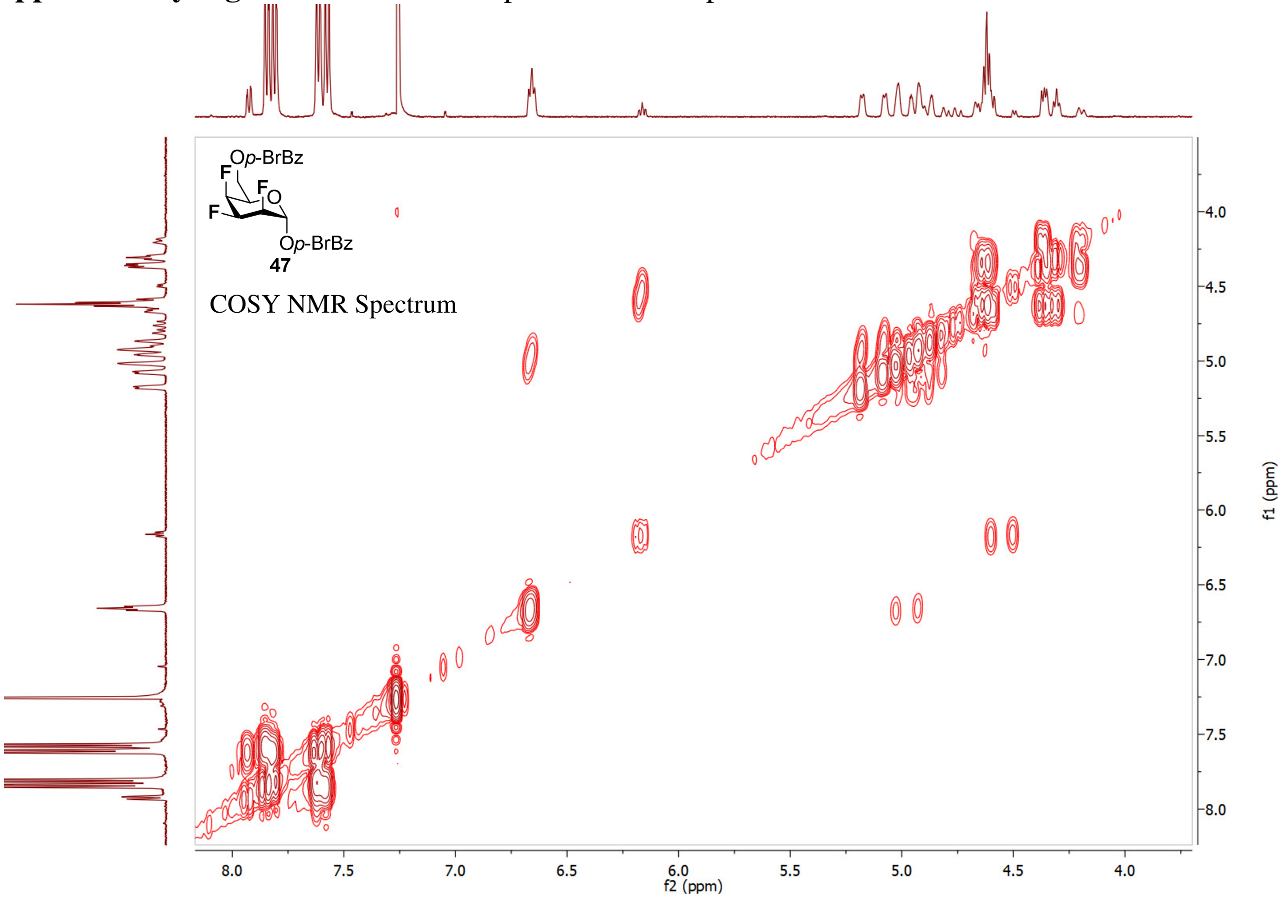

Supplementary Figure 135. COSY NMR spectrum of compound **47**

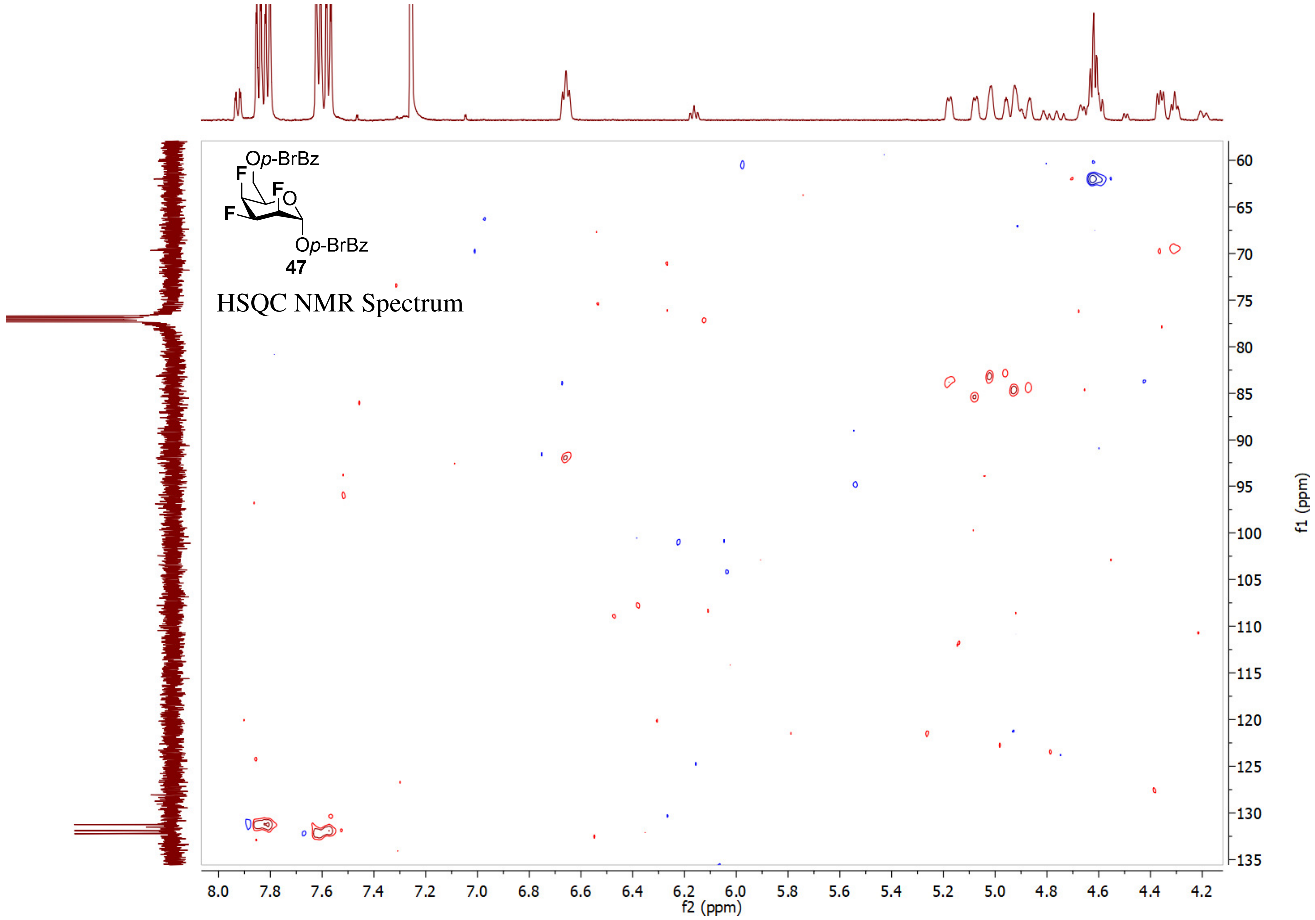

Supplementary Figure 136. HSQC NMR spectrum of compound **47**

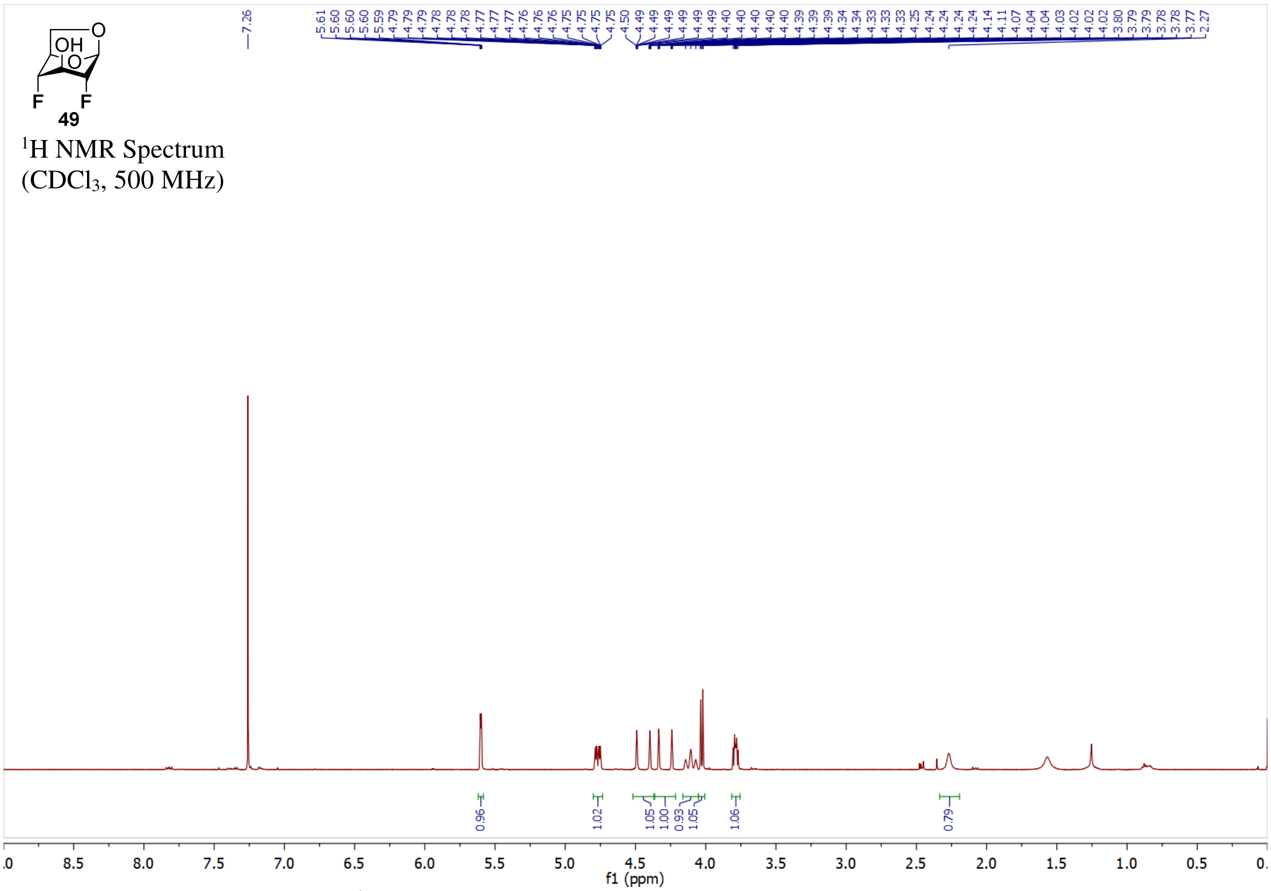

Supplementary Figure 137. ^{1}H NMR spectrum of compound **49**

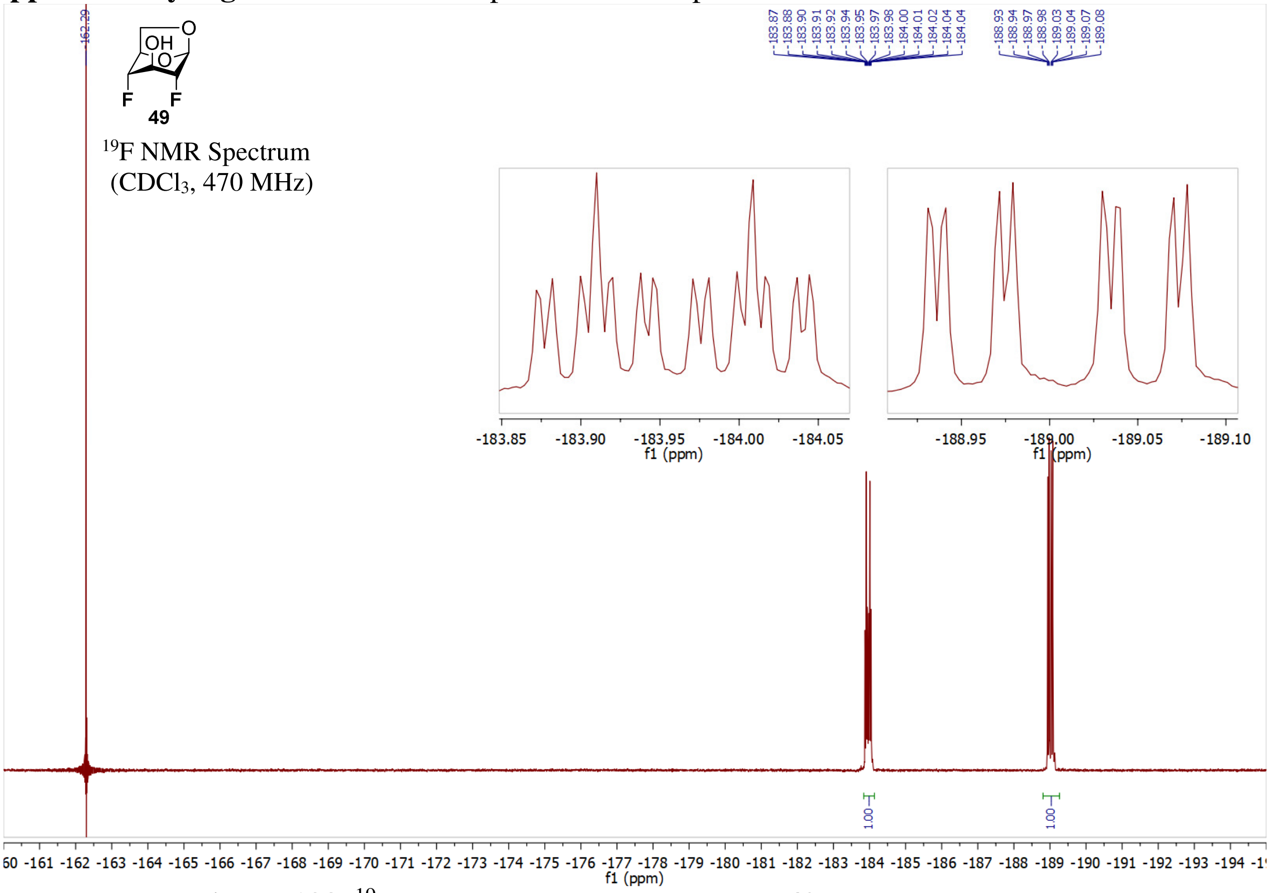

Supplementary Figure 138. ^{19}F NMR spectrum of compound **49**

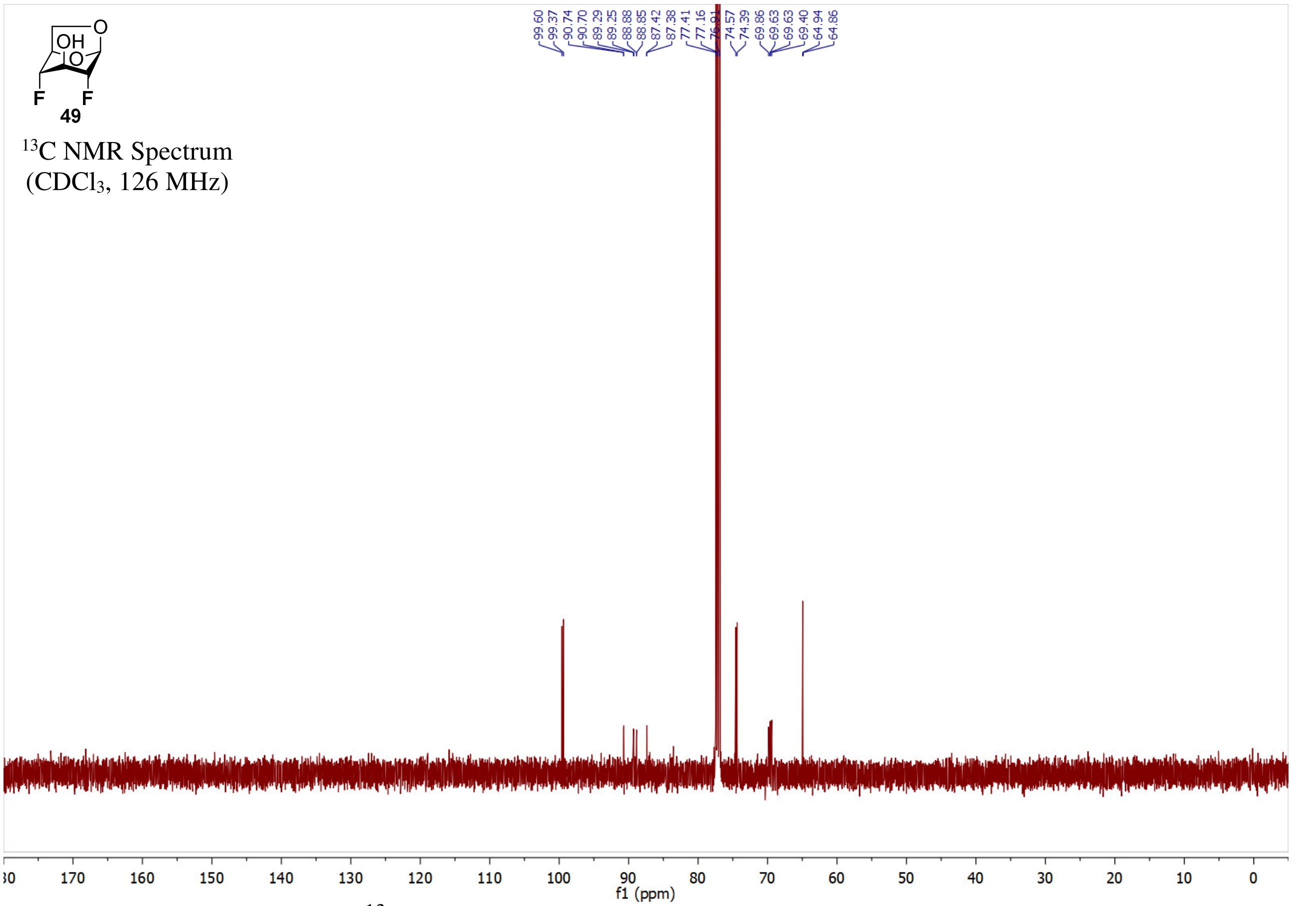

Supplementary Figure 139. ^{13}C NMR spectrum of compound **49**

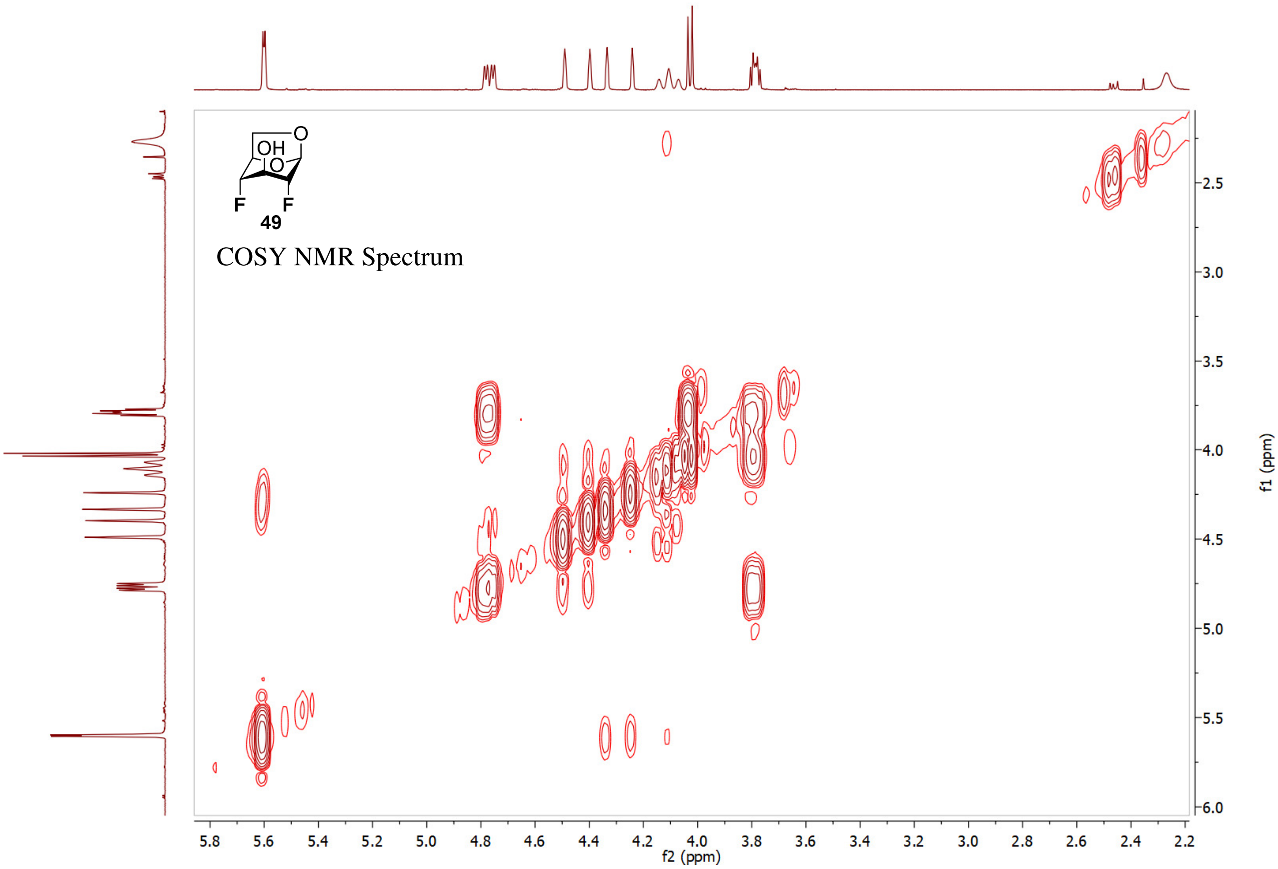

Supplementary Figure 140. COSY NMR spectrum of compound **49**

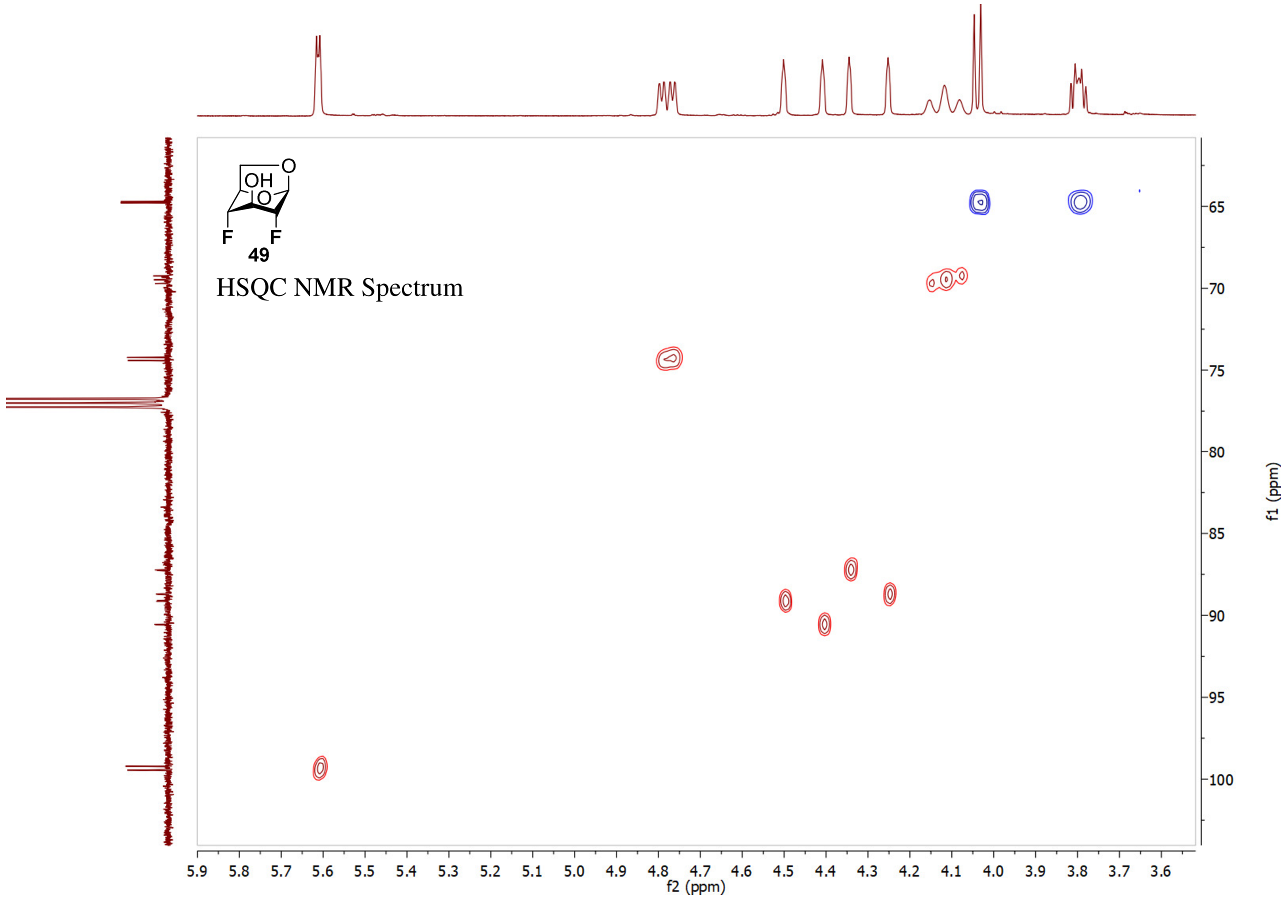

Supplementary Figure 141. HSQC NMR spectrum of compound **49**

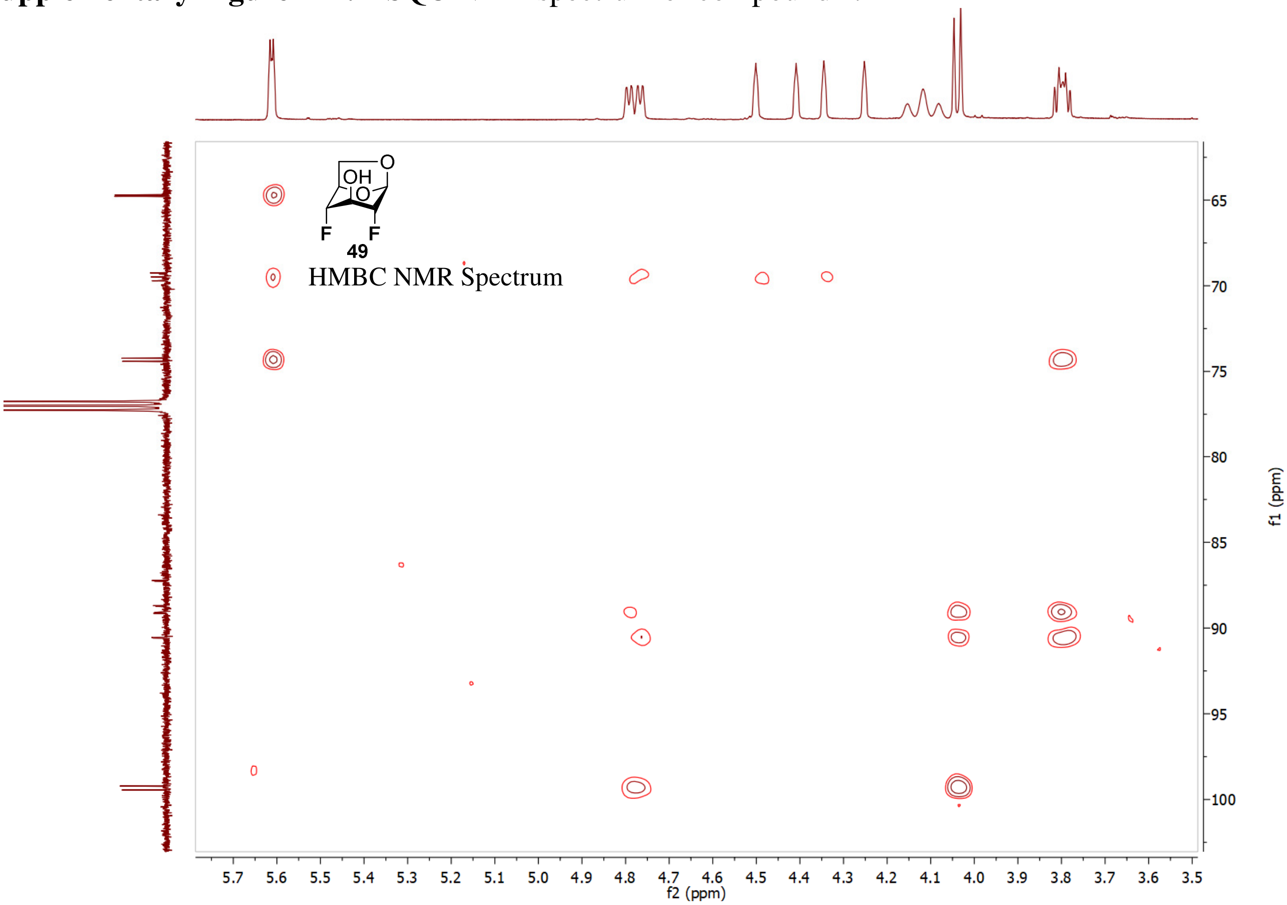

Supplementary Figure 142. HMBC NMR spectrum of compound **49**

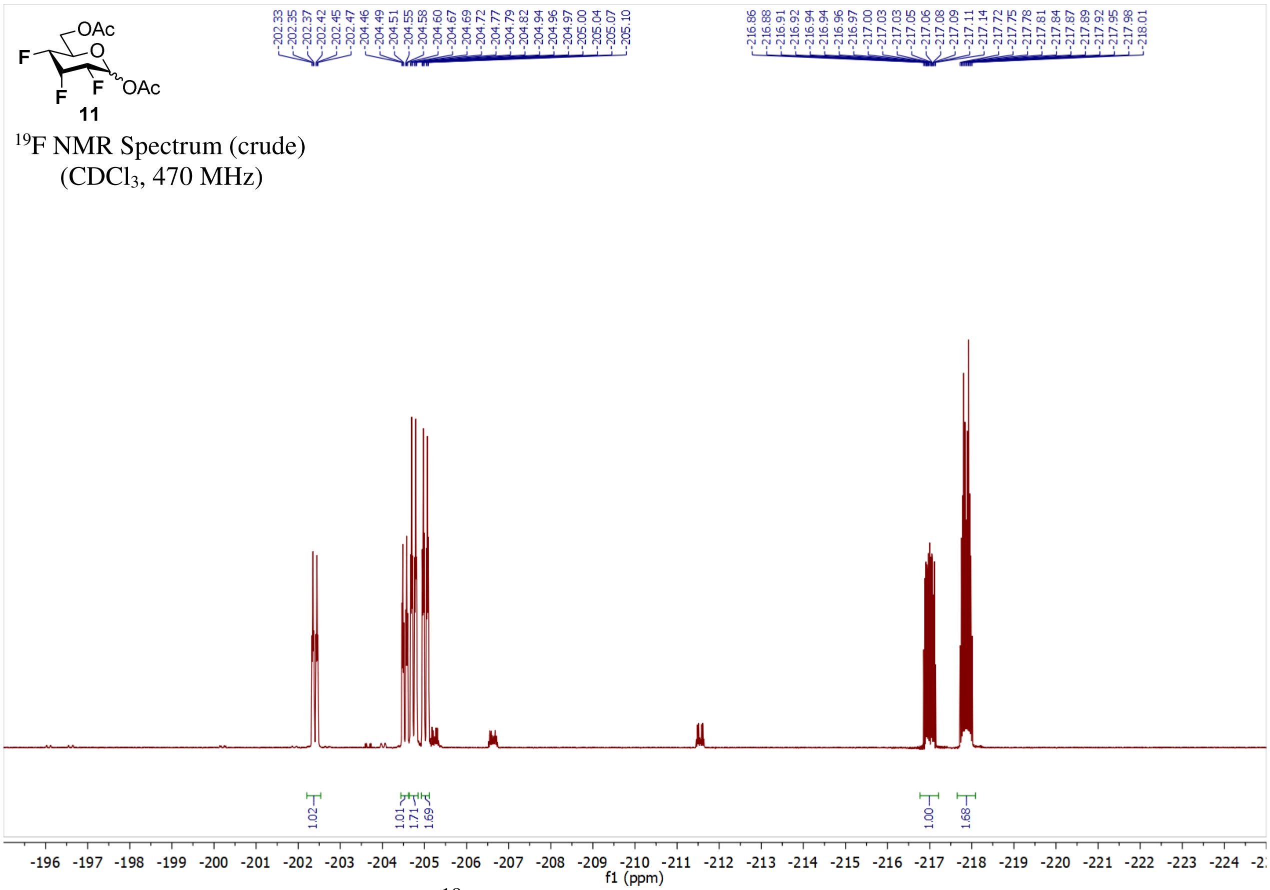

Supplementary Figure 143. Crude ^{19}F NMR spectrum of compound **11**

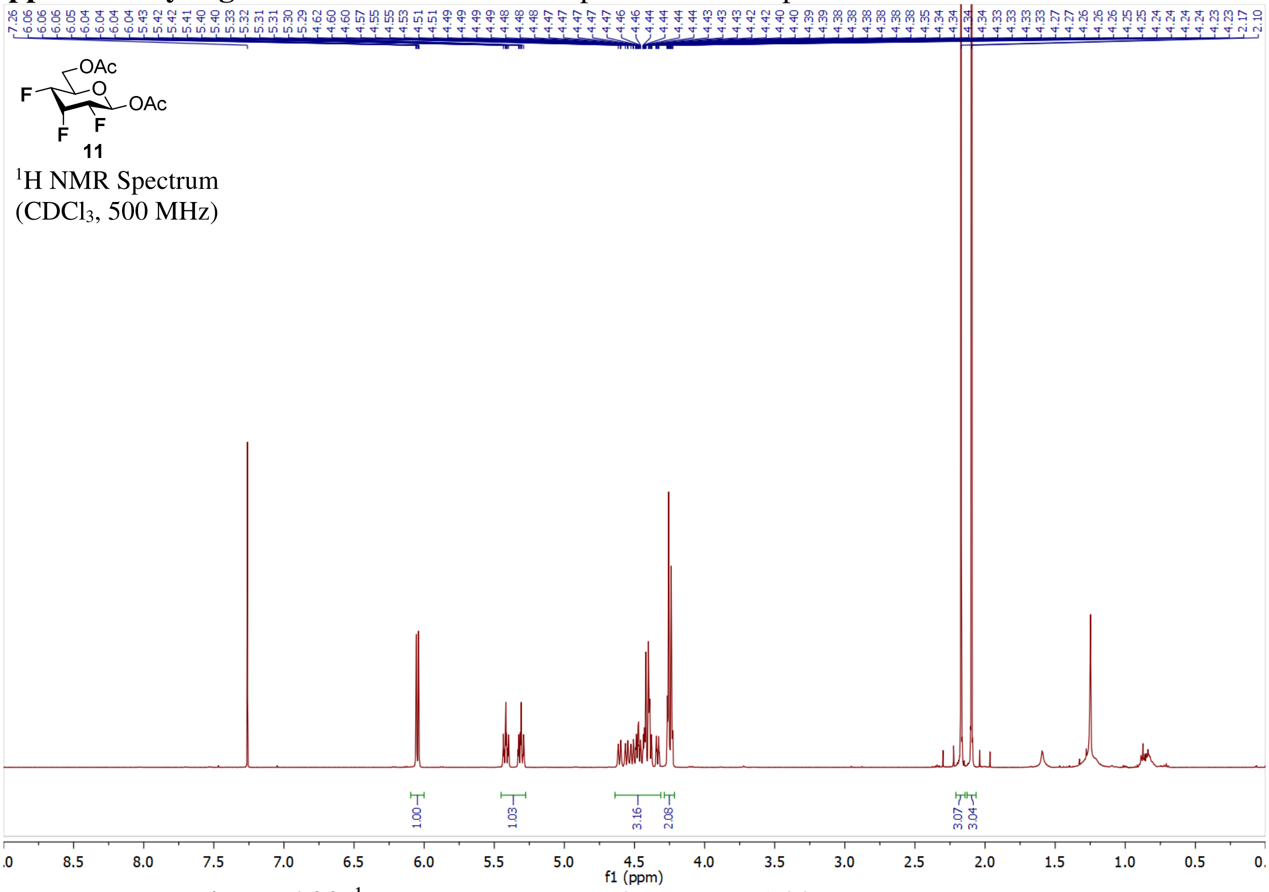

Supplementary Figure 144. ^{1}H NMR spectrum of compound **11**

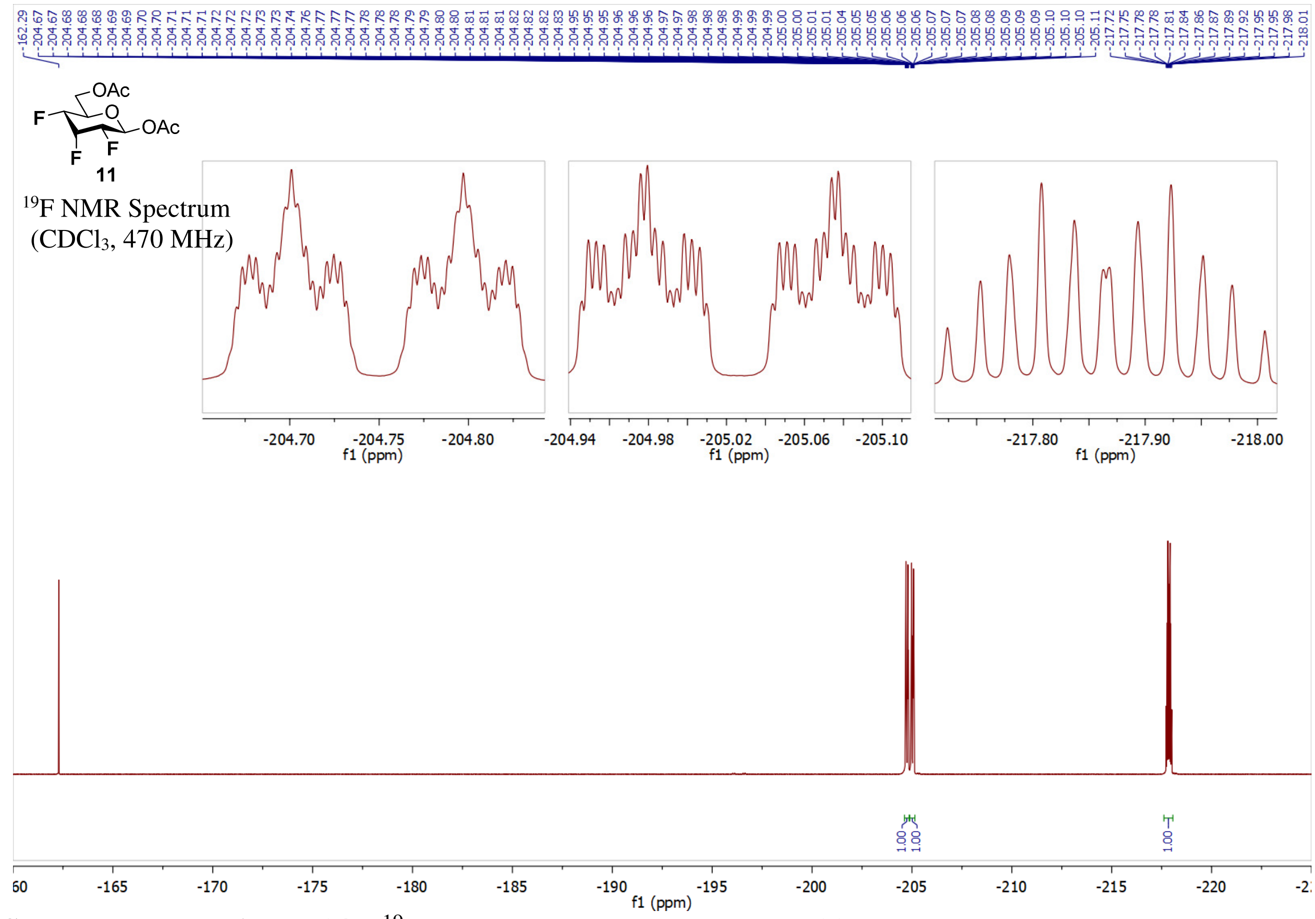

Supplementary Figure 145. ^{19}F NMR spectrum of compound **11**

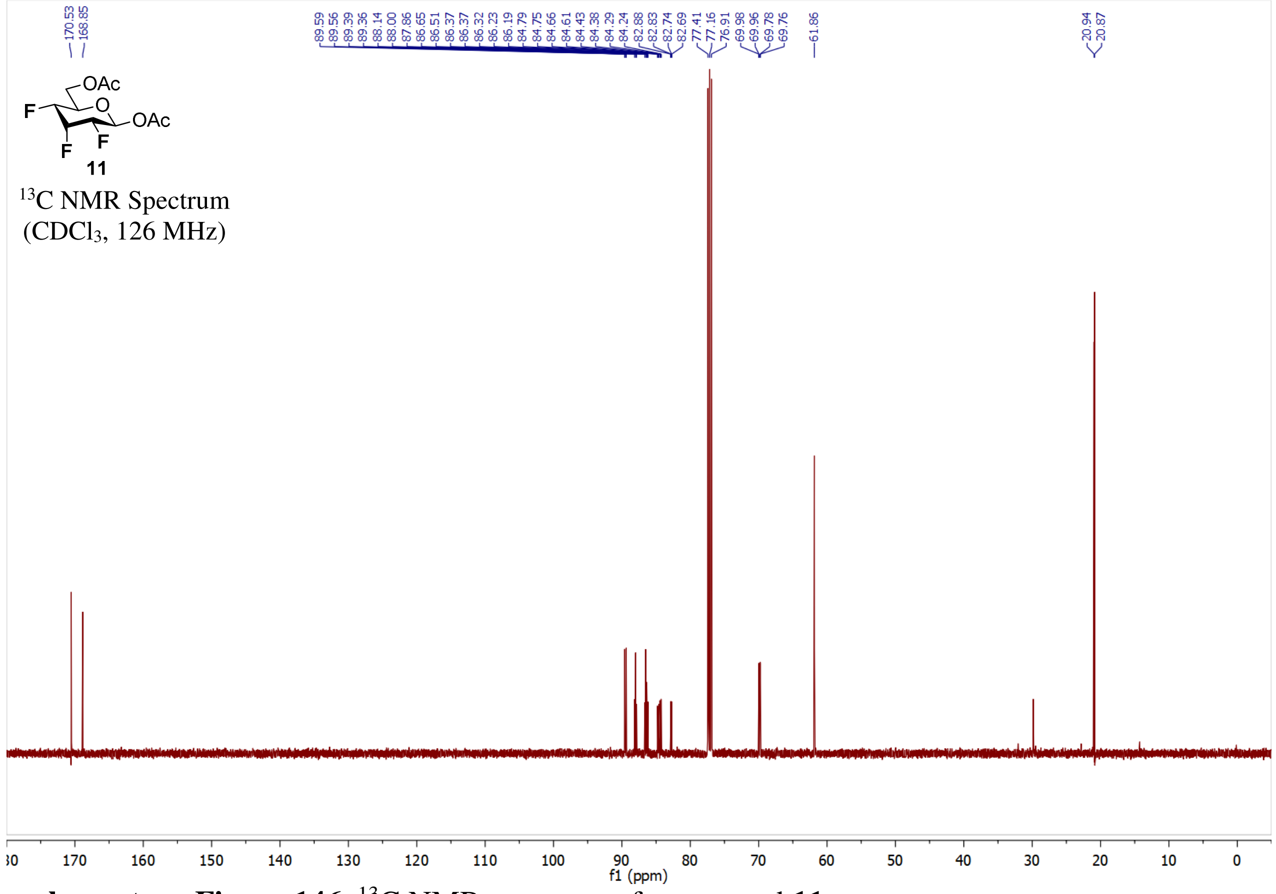

Supplementary Figure 146. ^{13}C NMR spectrum of compound **11**

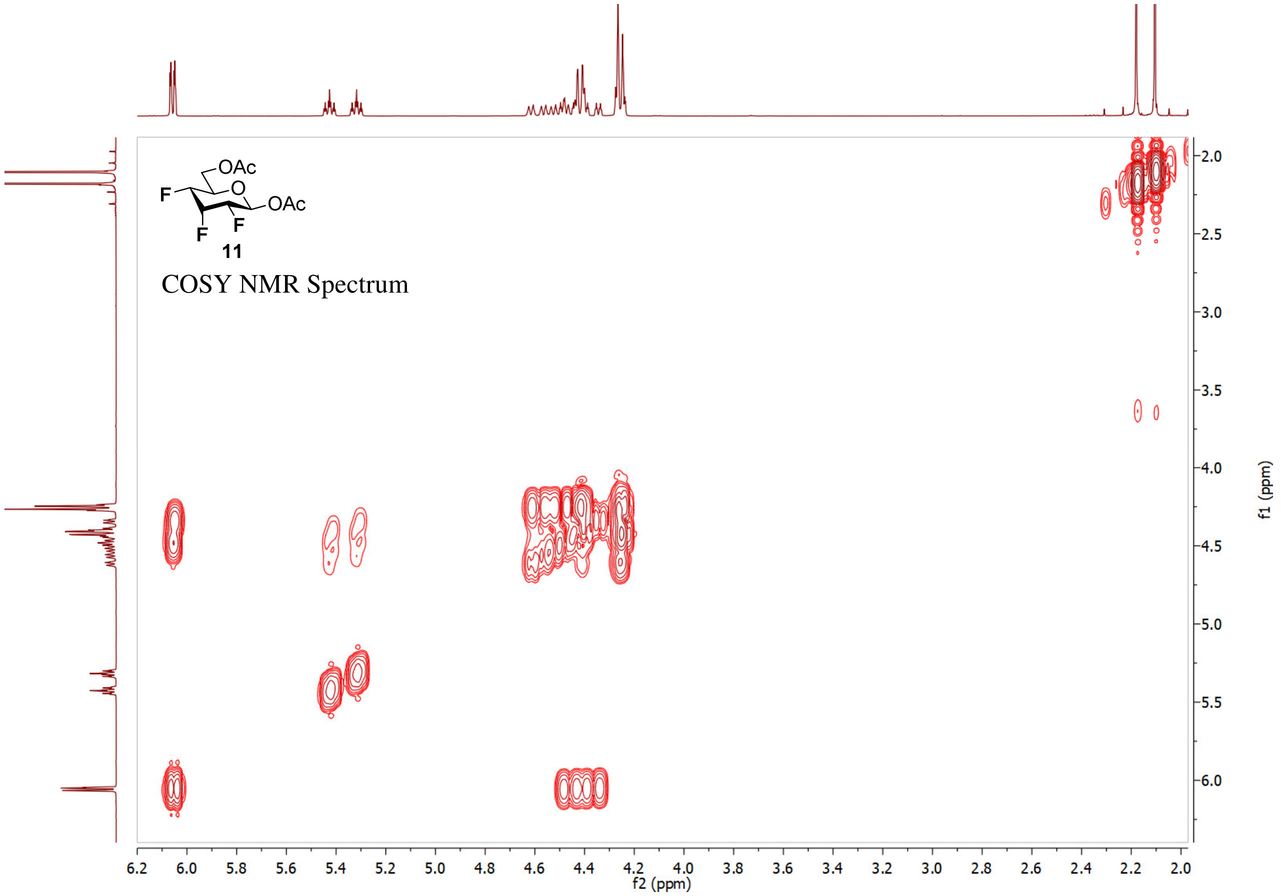

Supplementary Figure 147. COSY NMR spectrum of compound **11**

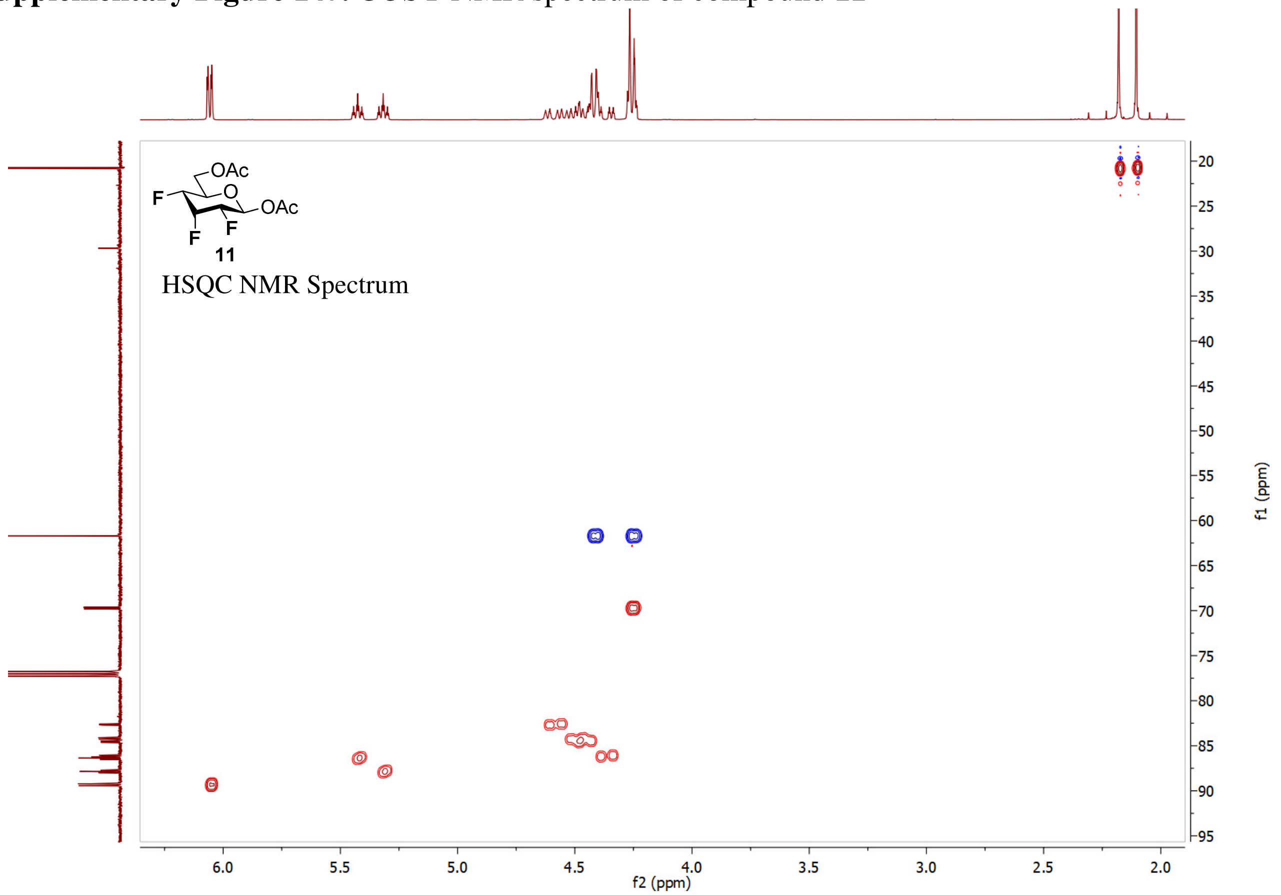

Supplementary Figure 148. HSQC NMR spectrum of compound **11**

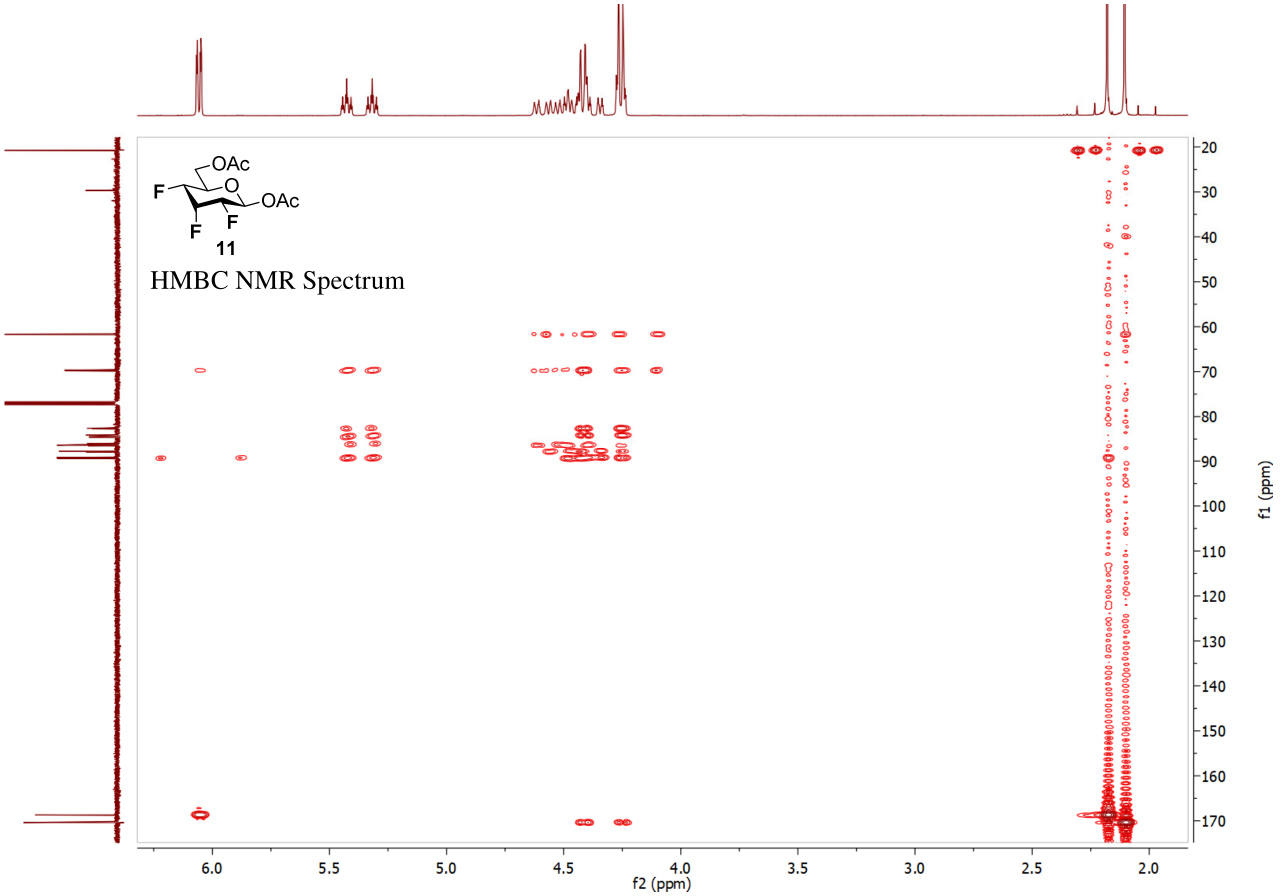

Supplementary Figure 149. HMBC NMR spectrum of compound **11**

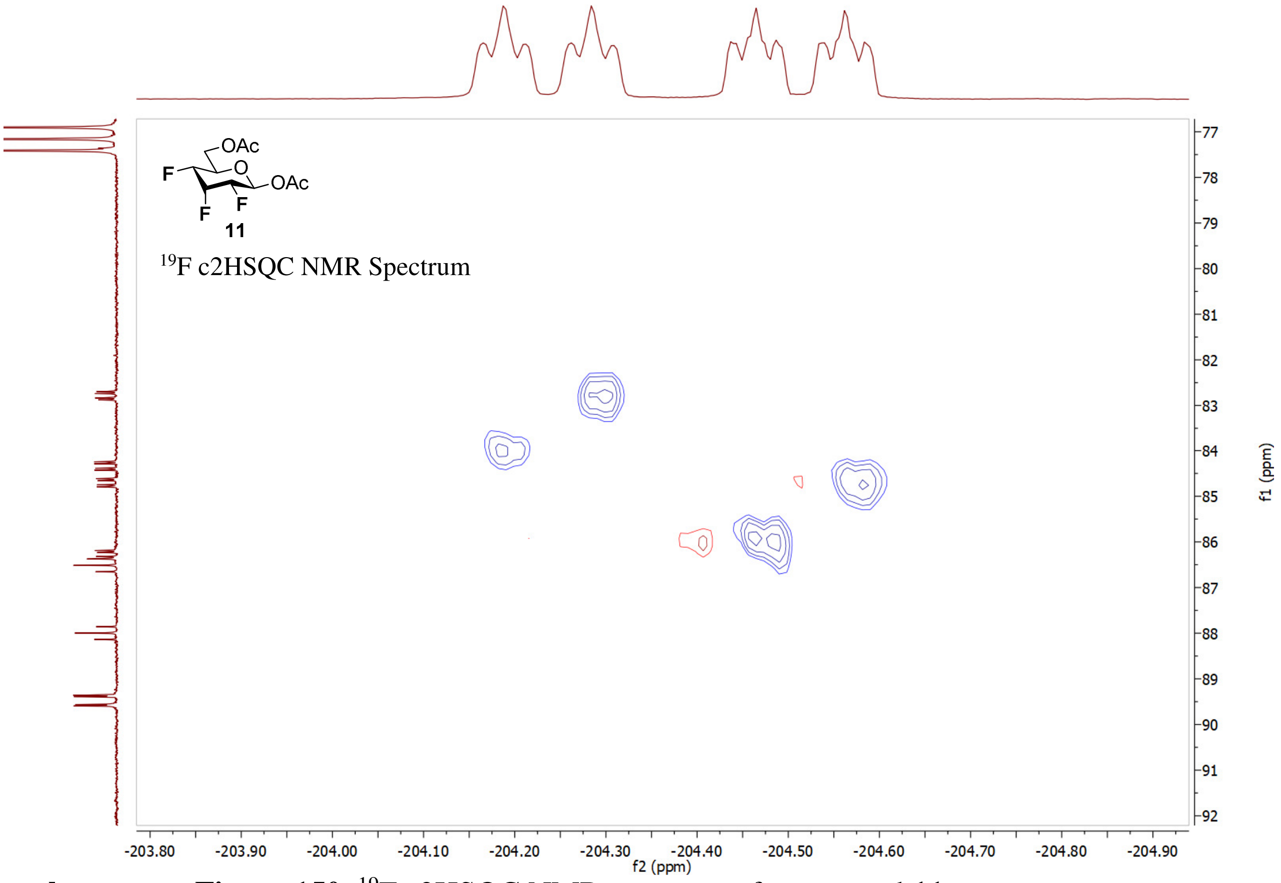

Supplementary Figure 150. ^{19}F c2HSQC NMR spectrum of compound **11**

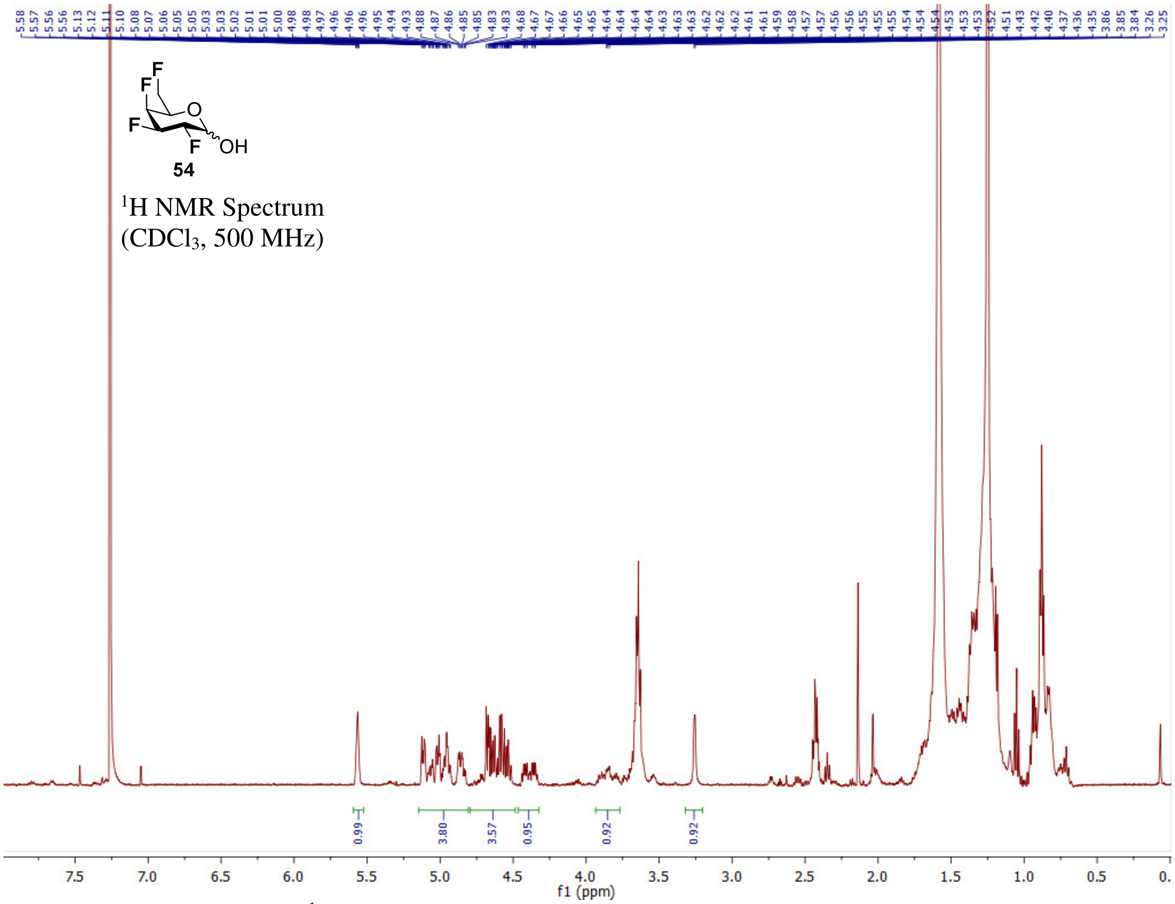

Supplementary Figure 151. ^{1}H NMR spectrum of compound **54**

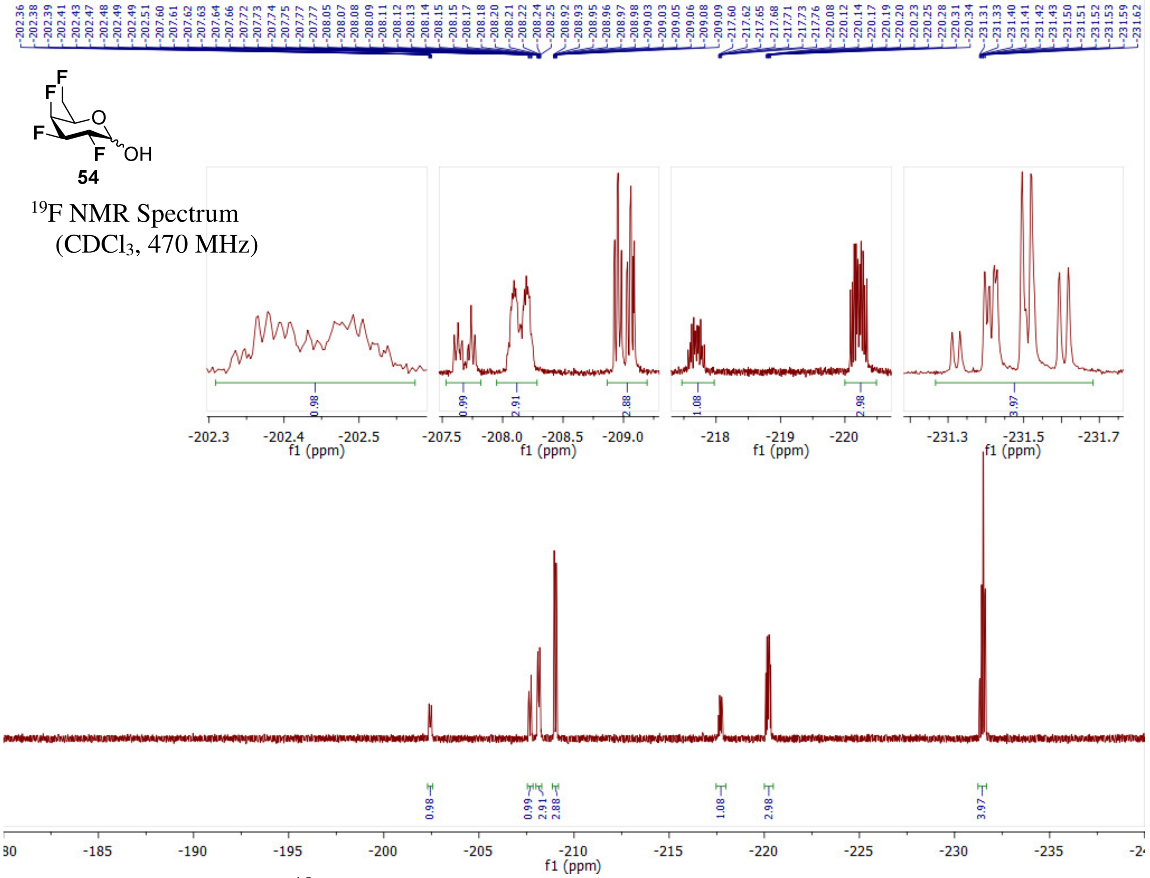

Supplementary Figure 152. ^{19}F NMR spectrum of compound **54**

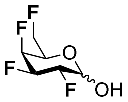

54

COSY NMR Spectrum

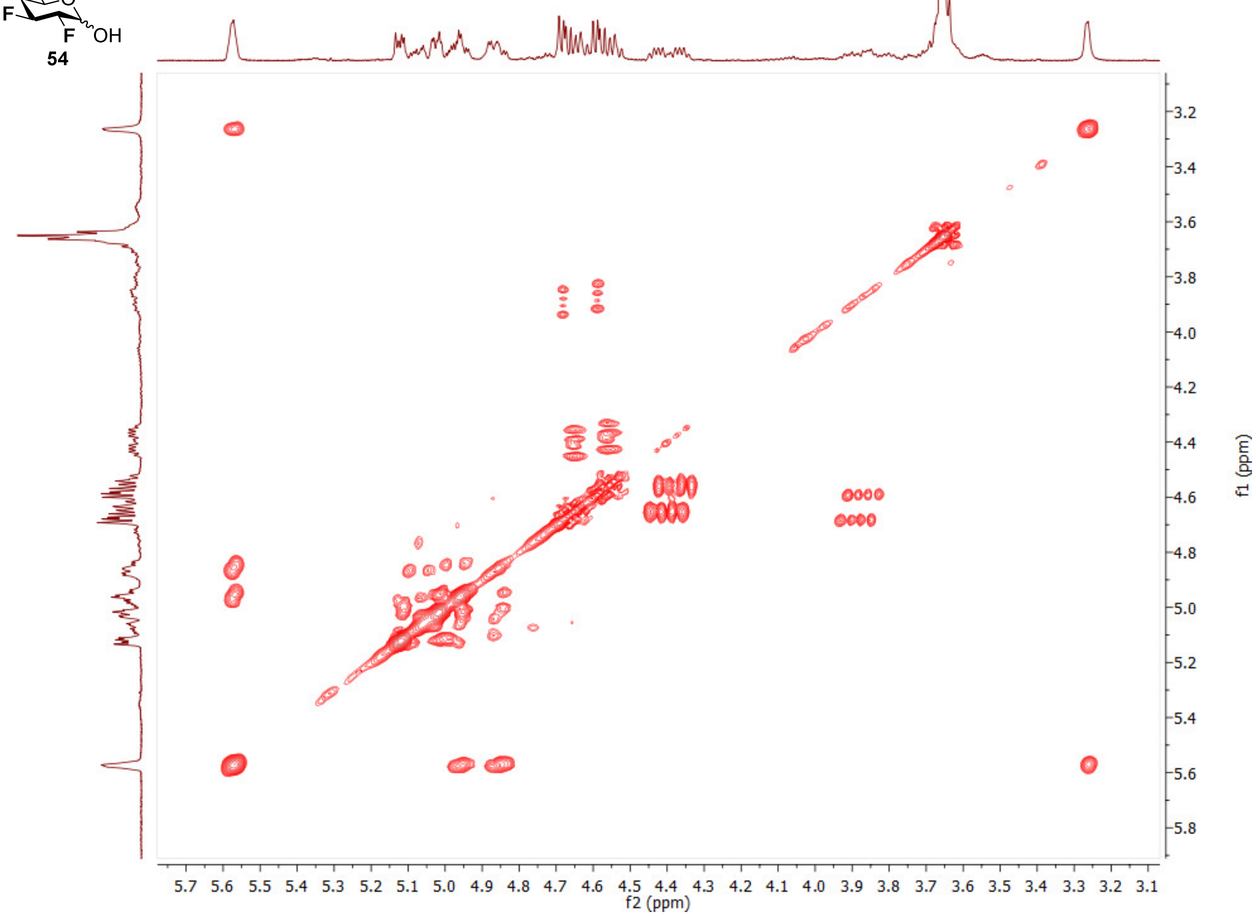

Supplementary Figure 153. COSY NMR spectrum of compound **54**

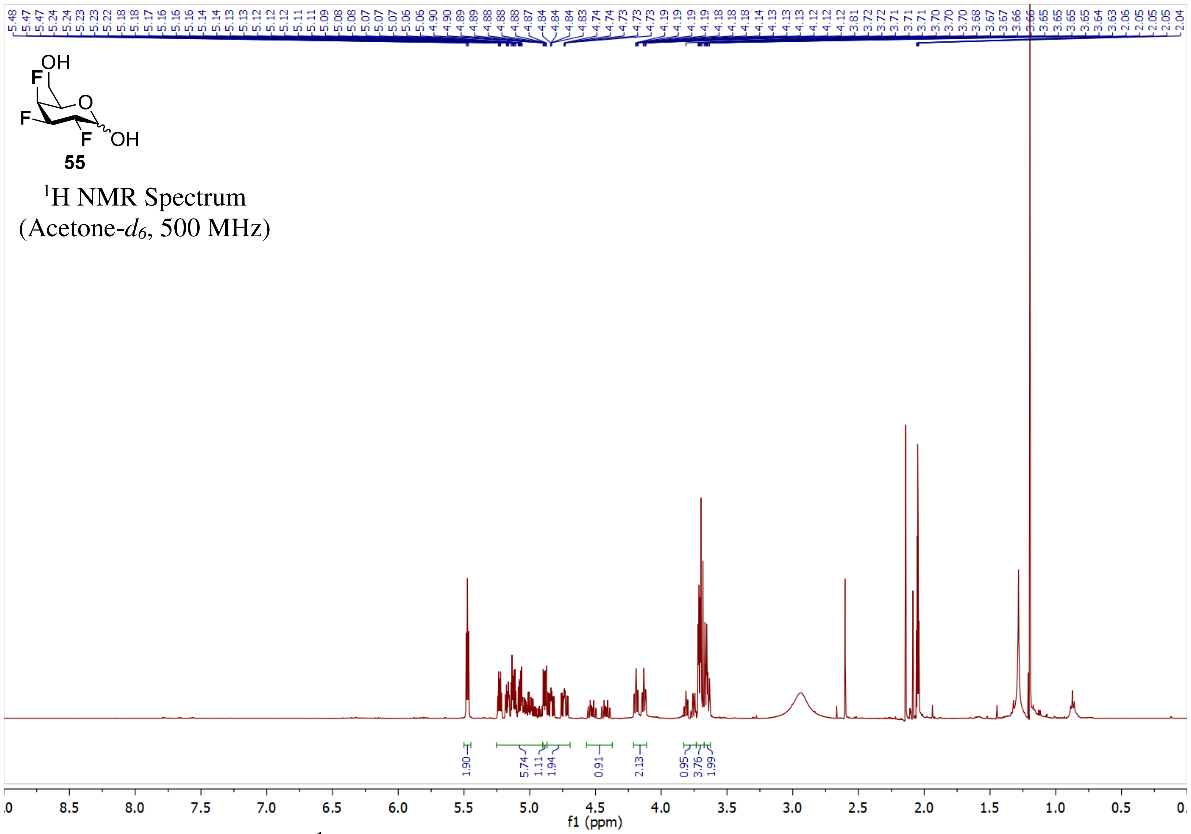

Supplementary Figure 154. ^{1}H NMR spectrum of compound **55**

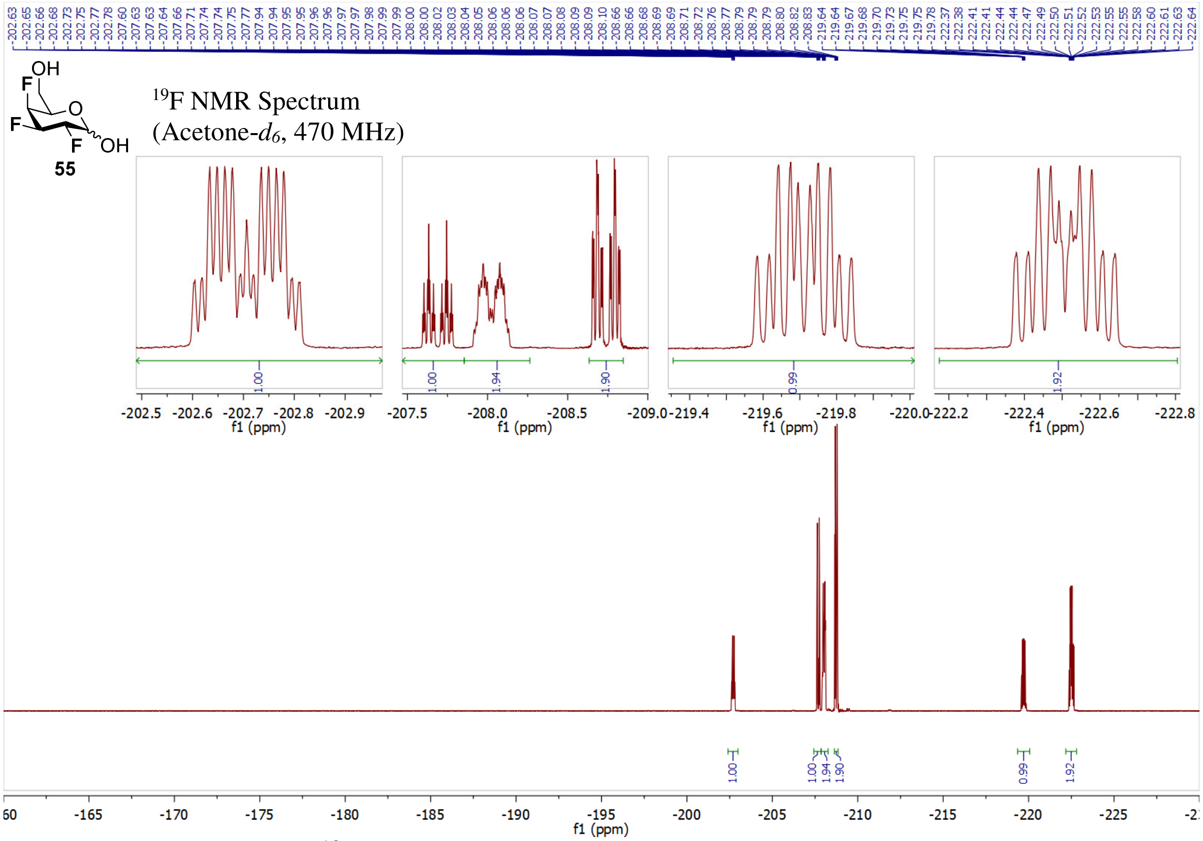

Supplementary Figure 155. ^{19}F NMR spectrum of compound **55**

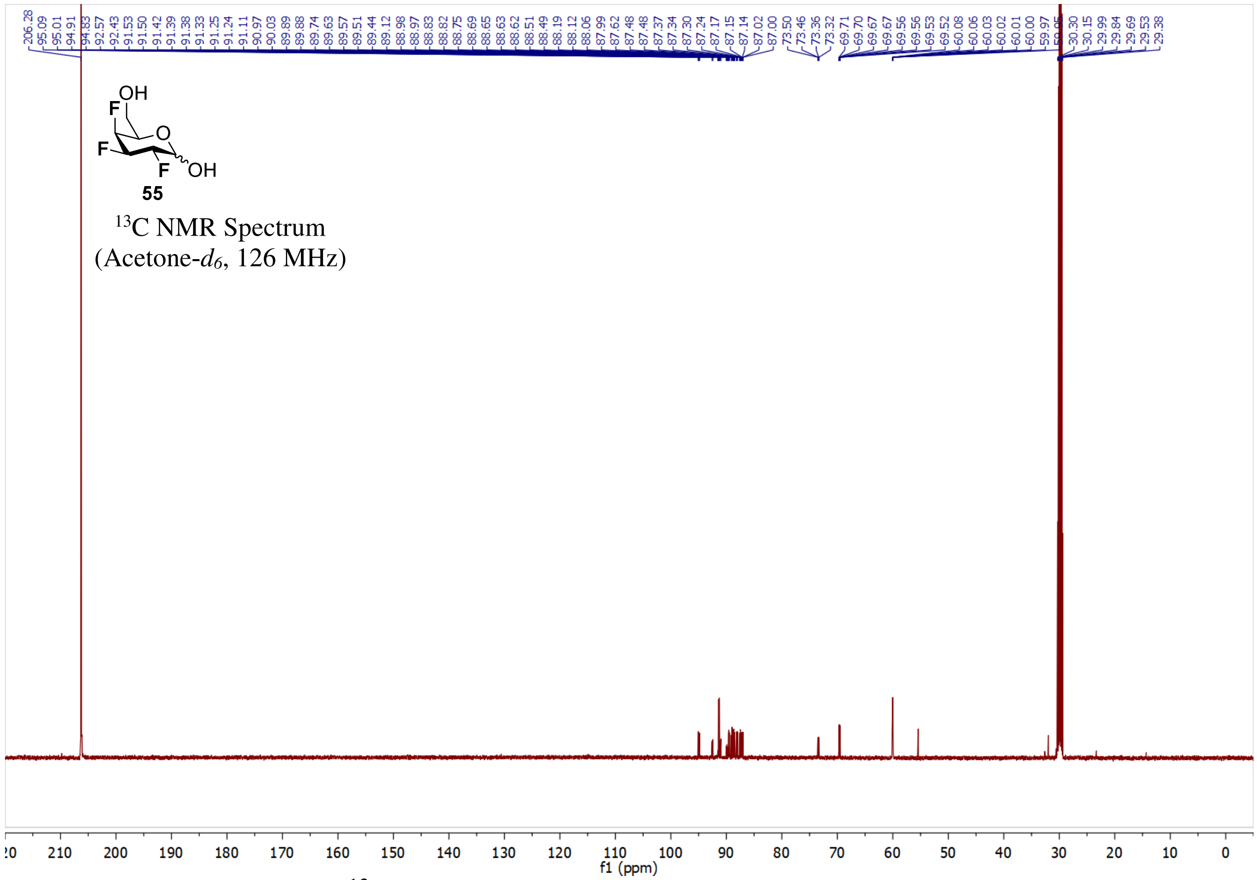

Supplementary Figure 156. ^{13}C NMR spectrum of compound **55**

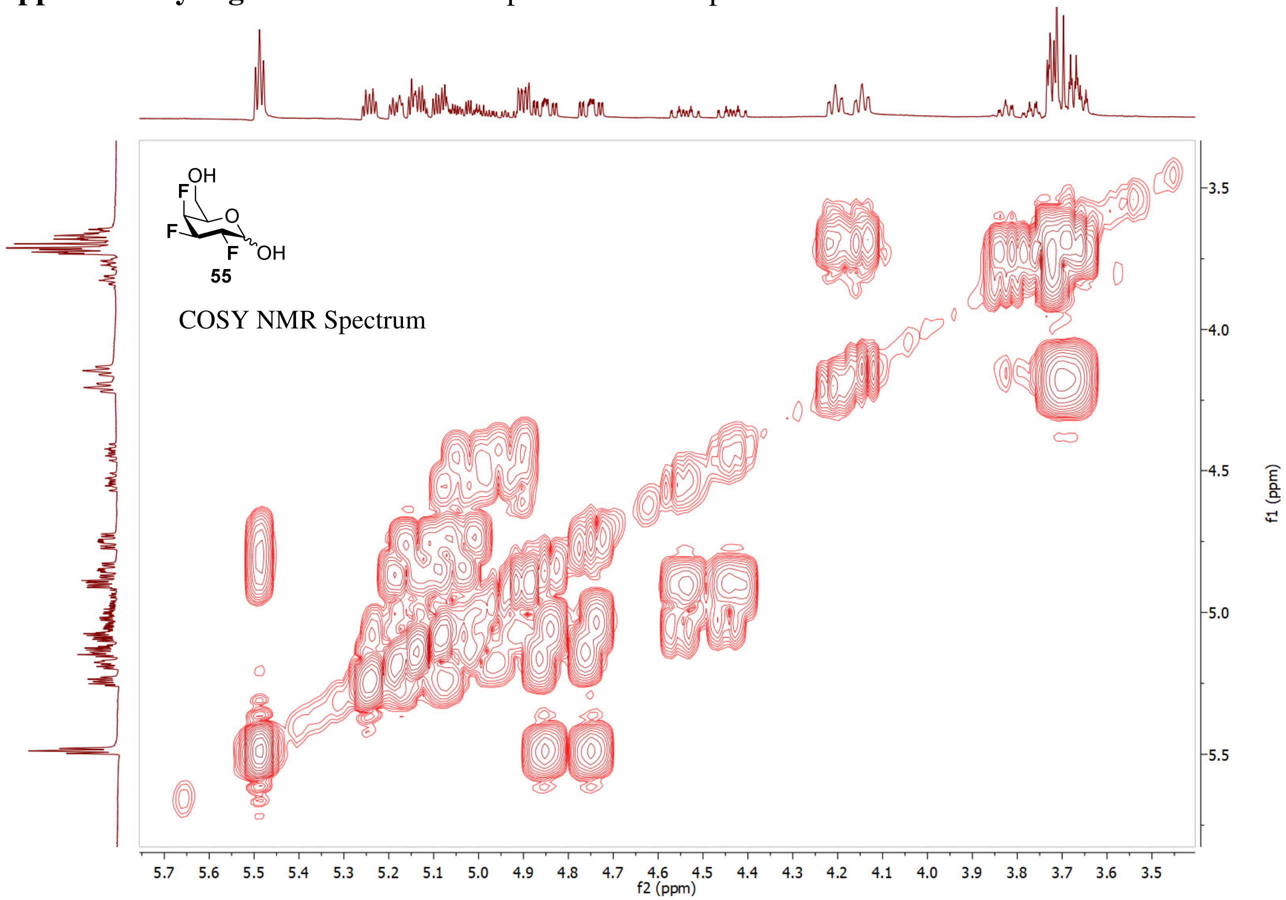

Supplementary Figure 157. COSY NMR spectrum of compound **55**

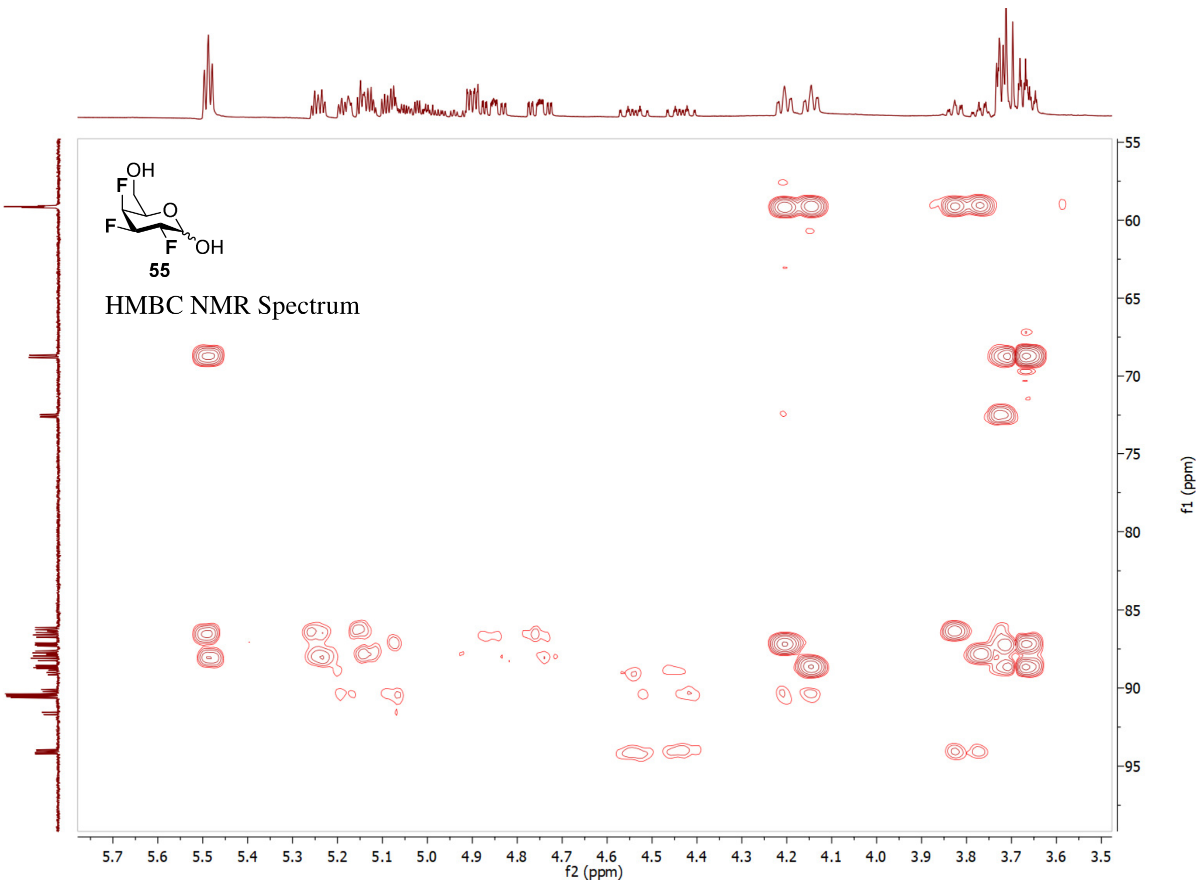

Supplementary Figure 158. HMBC NMR spectrum of compound **55**

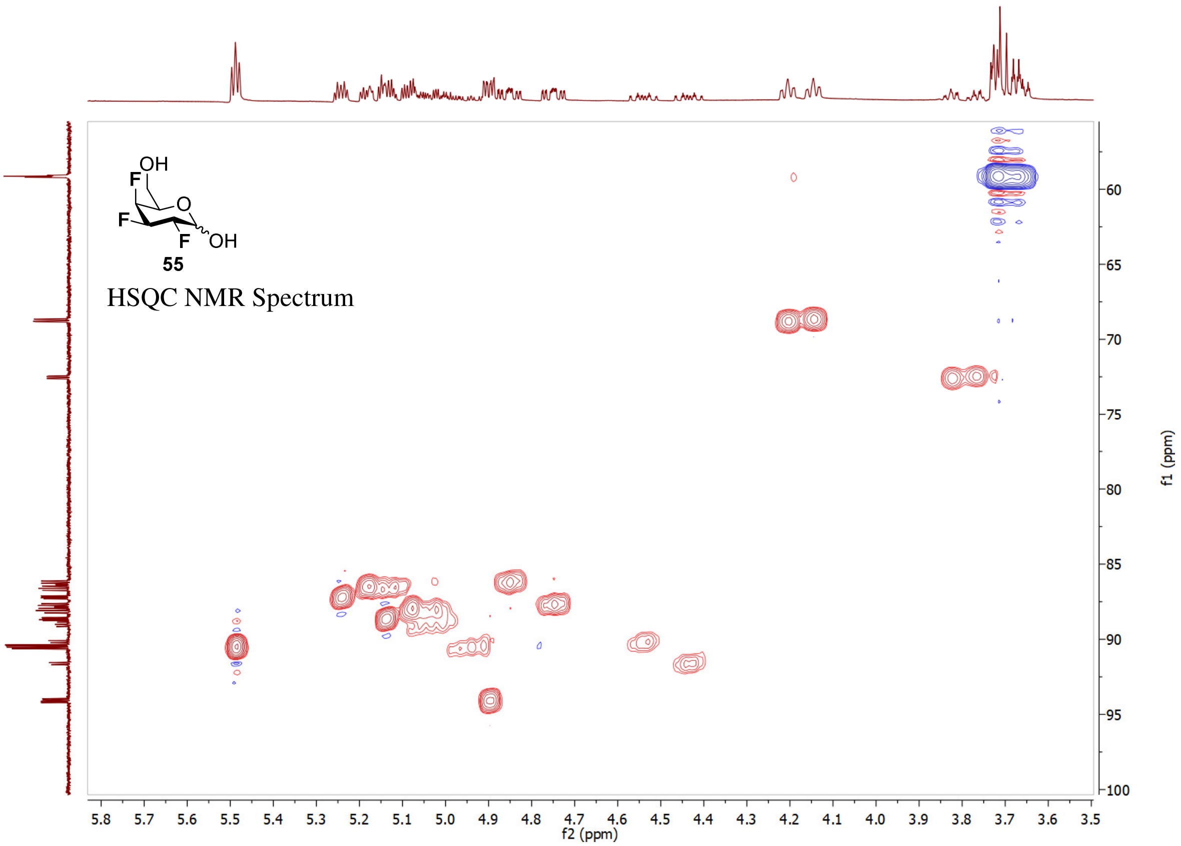

Supplementary Figure 159. HSQC NMR spectrum of compound **55**

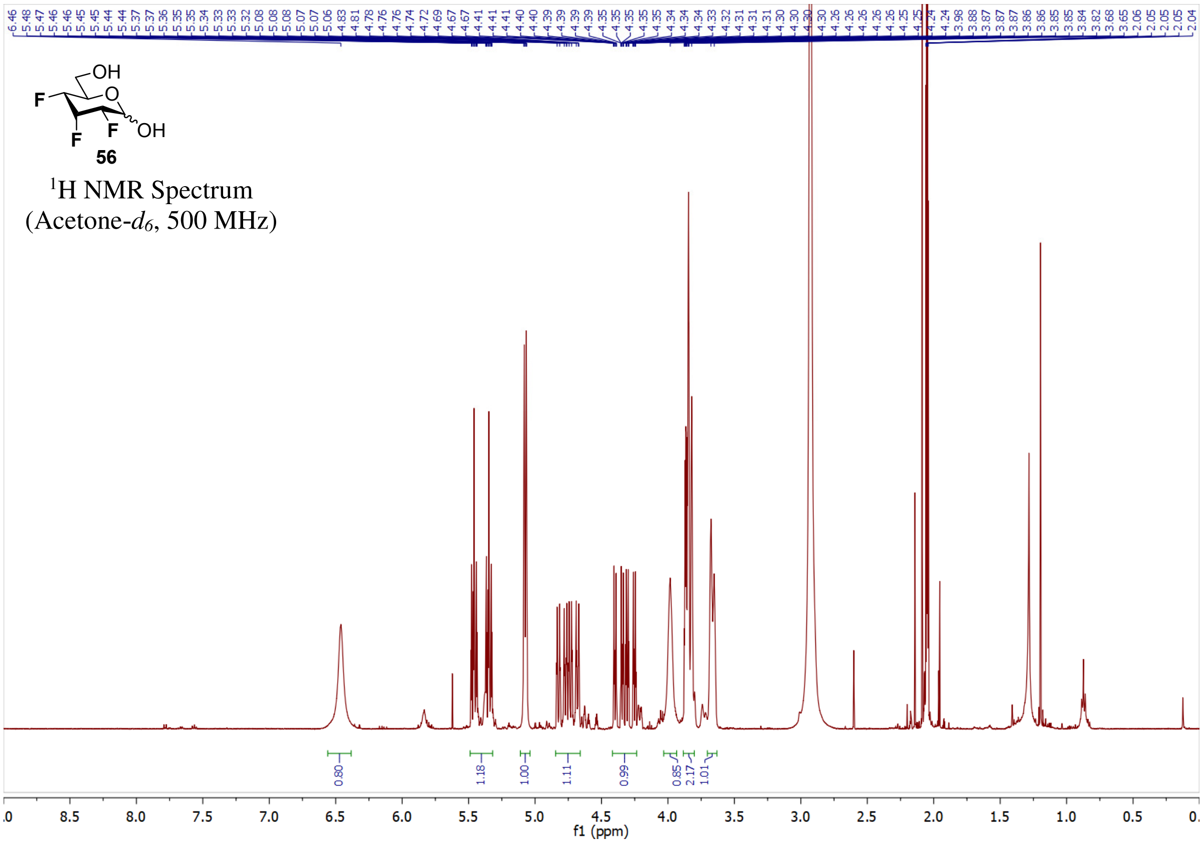

Supplementary Figure 160. ^{1}H NMR spectrum of compound **56**

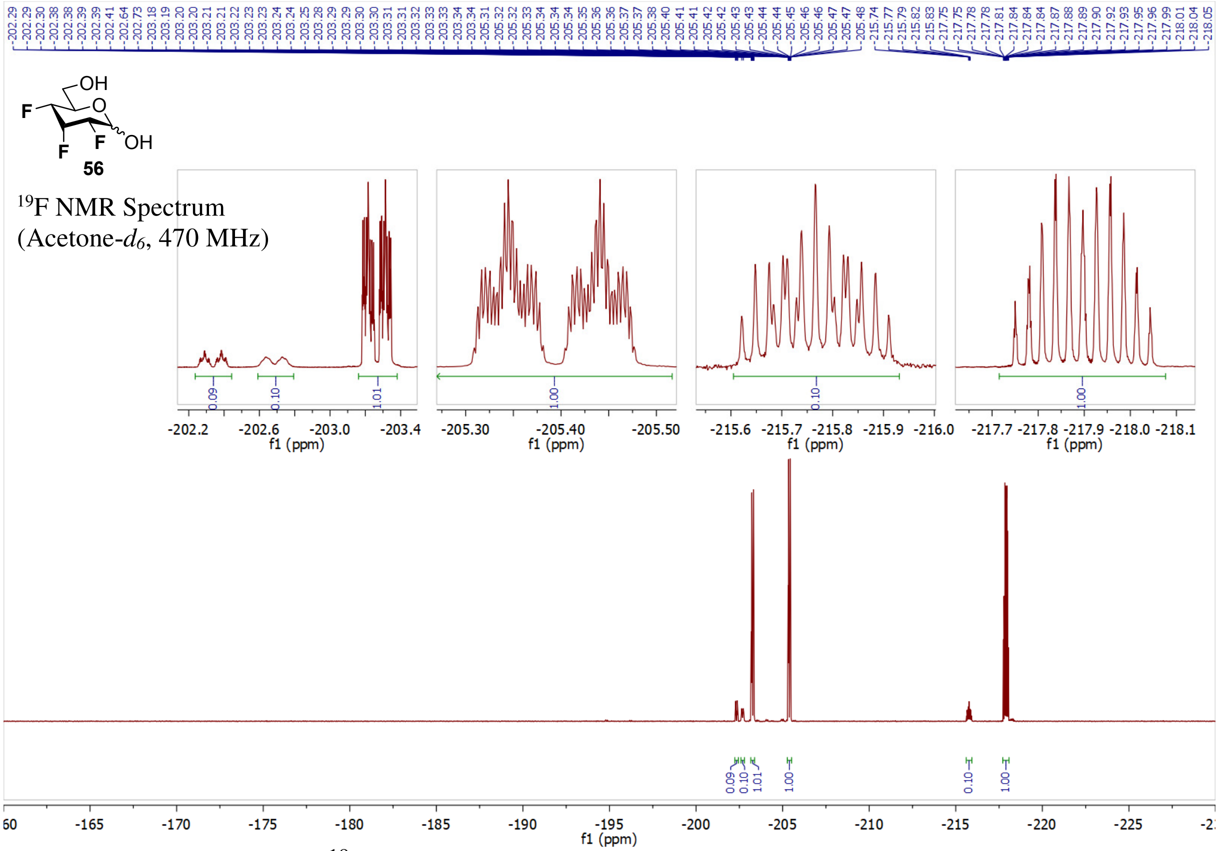

Supplementary Figure 161. ^{19}F NMR spectrum of compound **56**

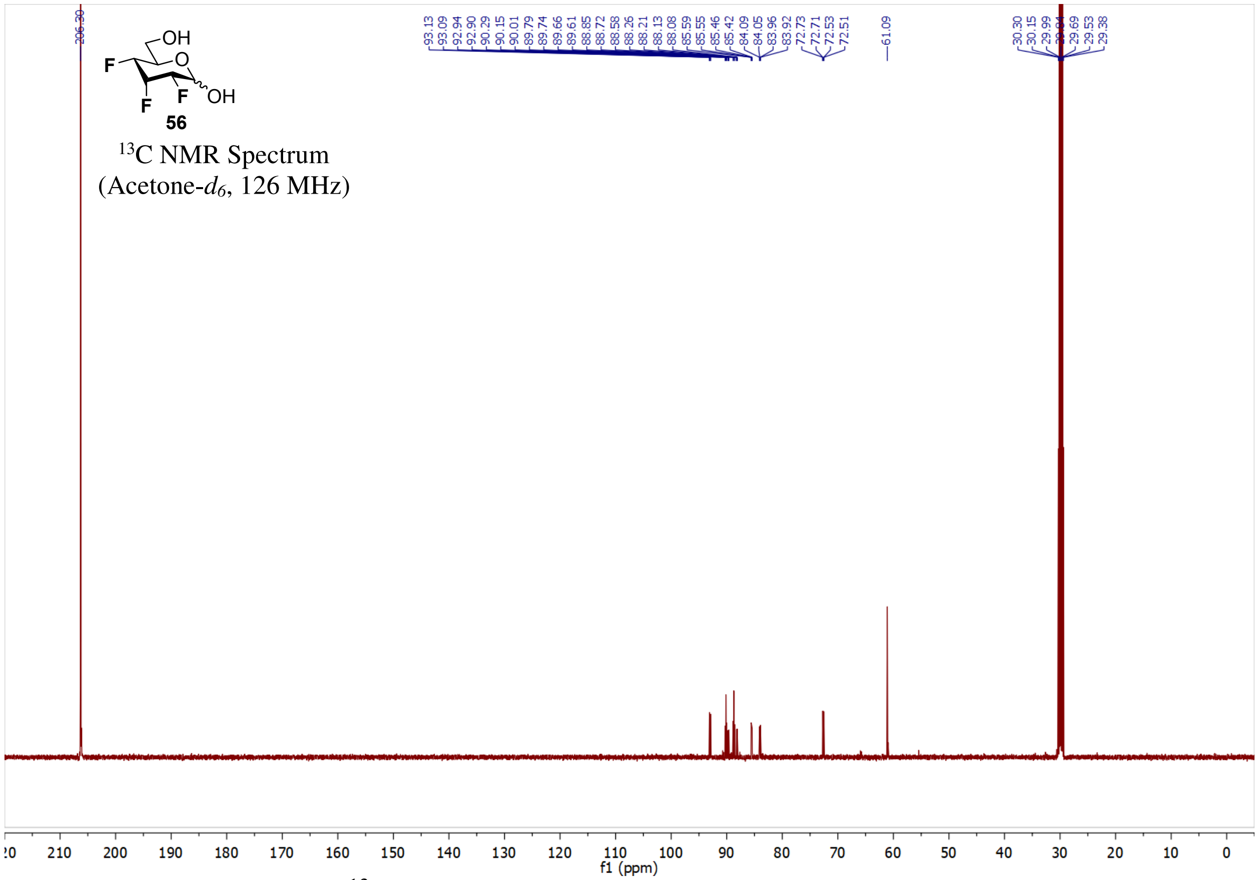

Supplementary Figure 162. ^{13}C NMR spectrum of compound **56**

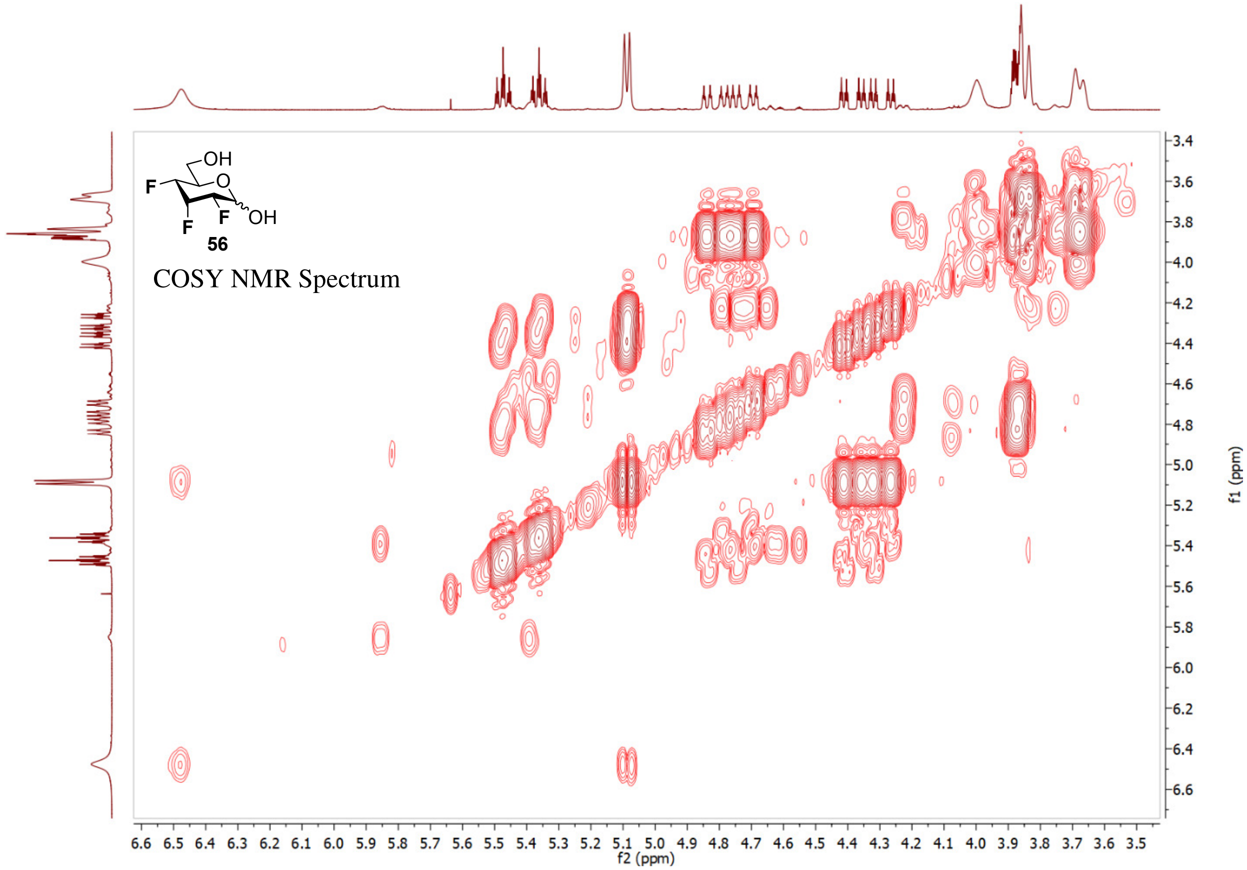

Supplementary Figure 163. COSY NMR spectrum of compound **56**

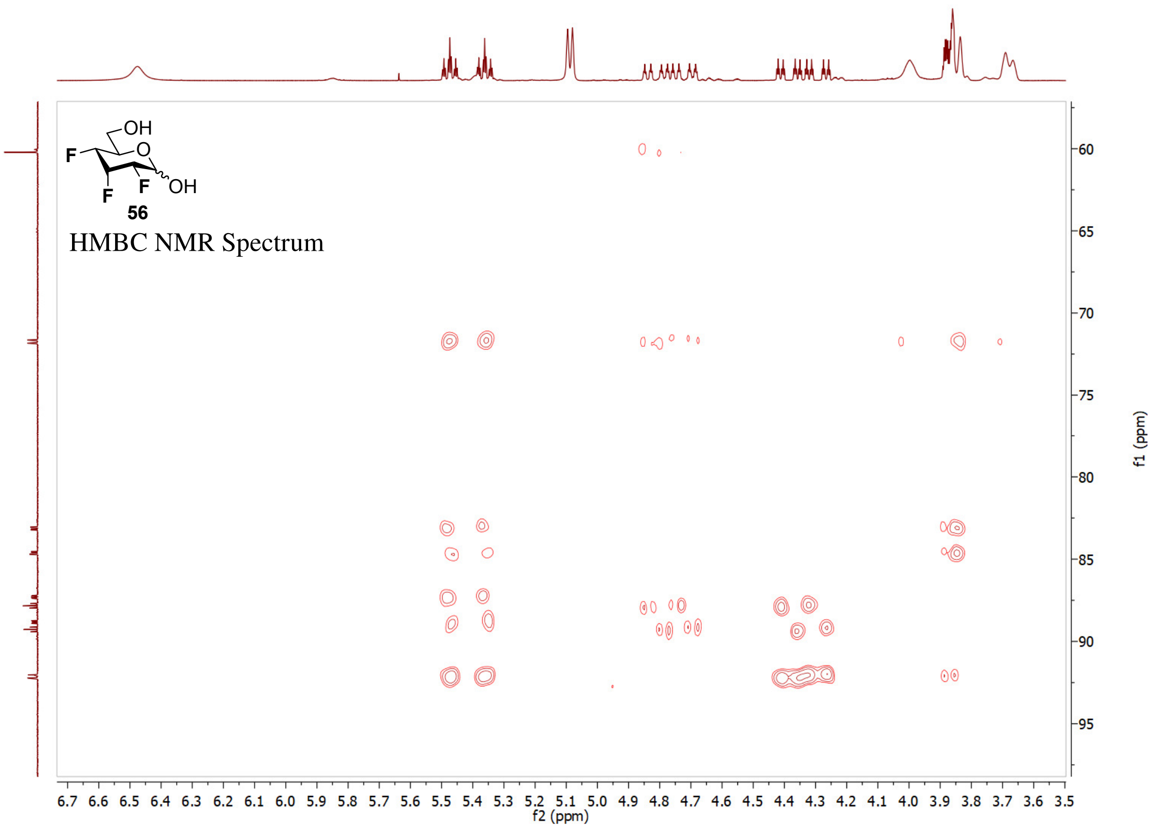

Supplementary Figure 164. HMBC NMR spectrum of compound **56**

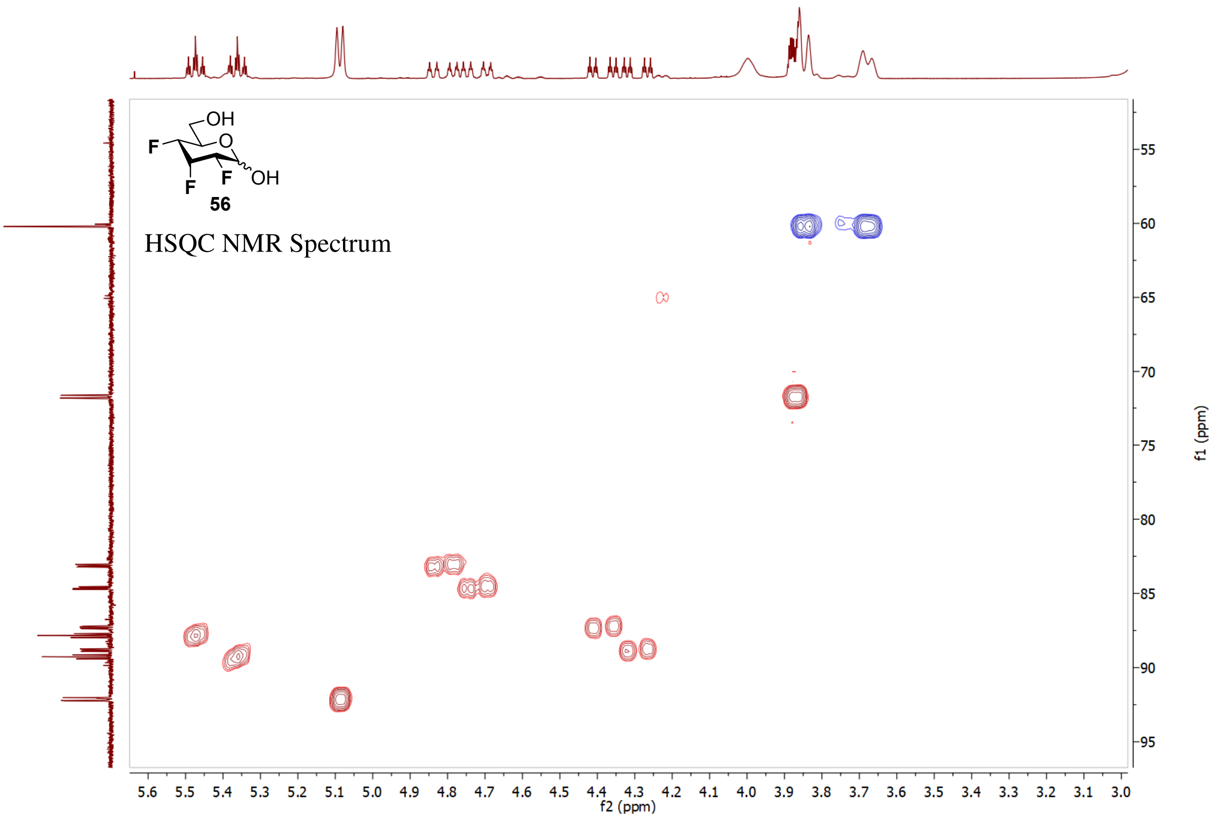

Supplementary Figure 165. HSQC NMR spectrum of compound **56**

Log*P* determination using the NMR

We used the log*P* determination method developed by the group of Linclau. For the NMR experiments, we used the same parameters as the one described in their original article.

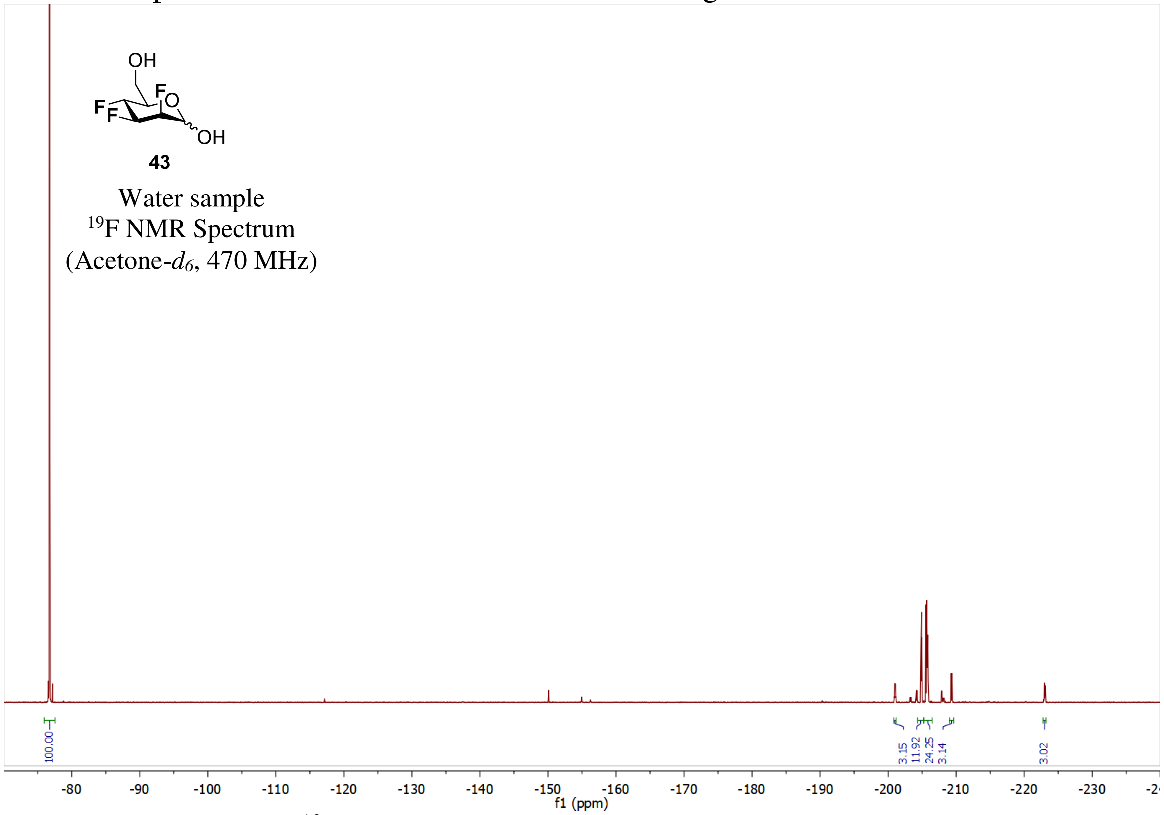

Supplementary Figure 166. ^{19}F NMR spectrum of the water sample of compound **43**

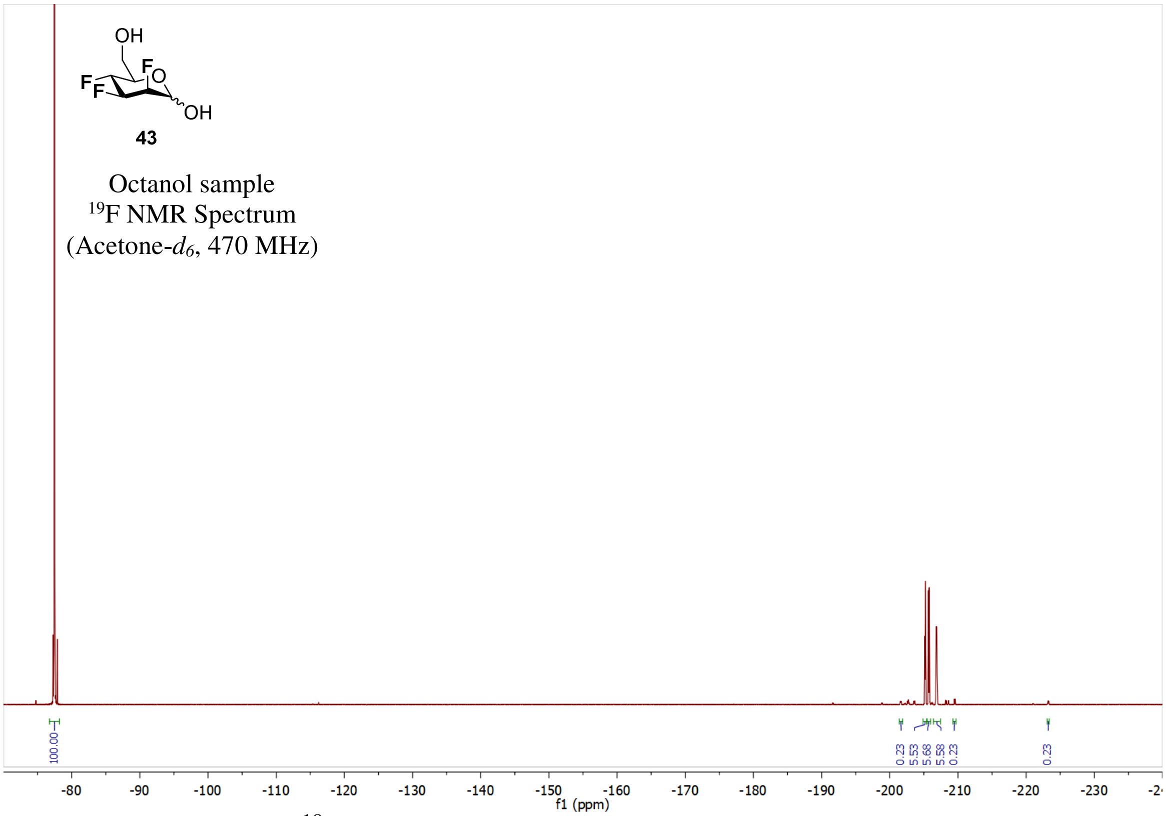

Supplementary Figure 167. ^{19}F NMR spectrum of the octanol sample of compound **43**

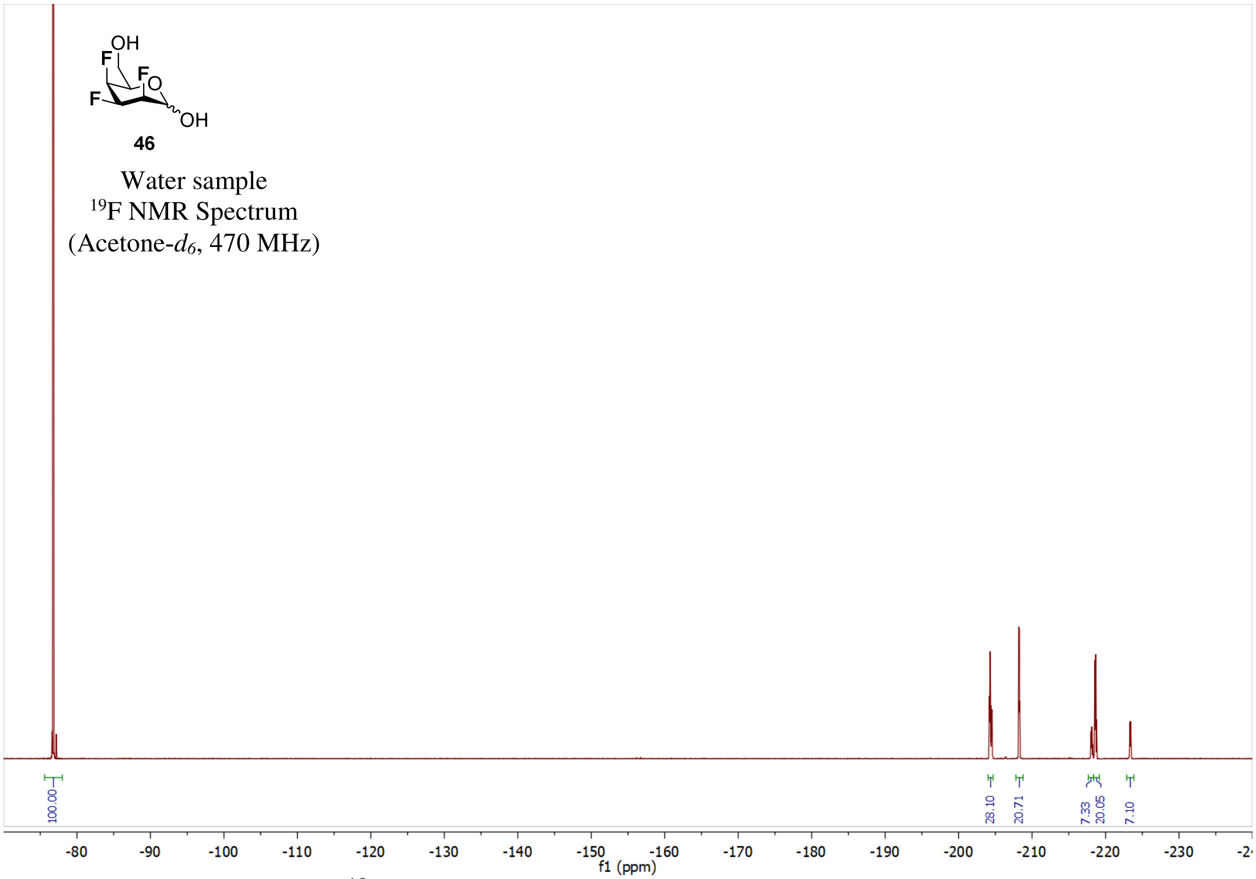

Supplementary Figure 168. ^{19}F NMR spectrum of the water sample of compound **46**

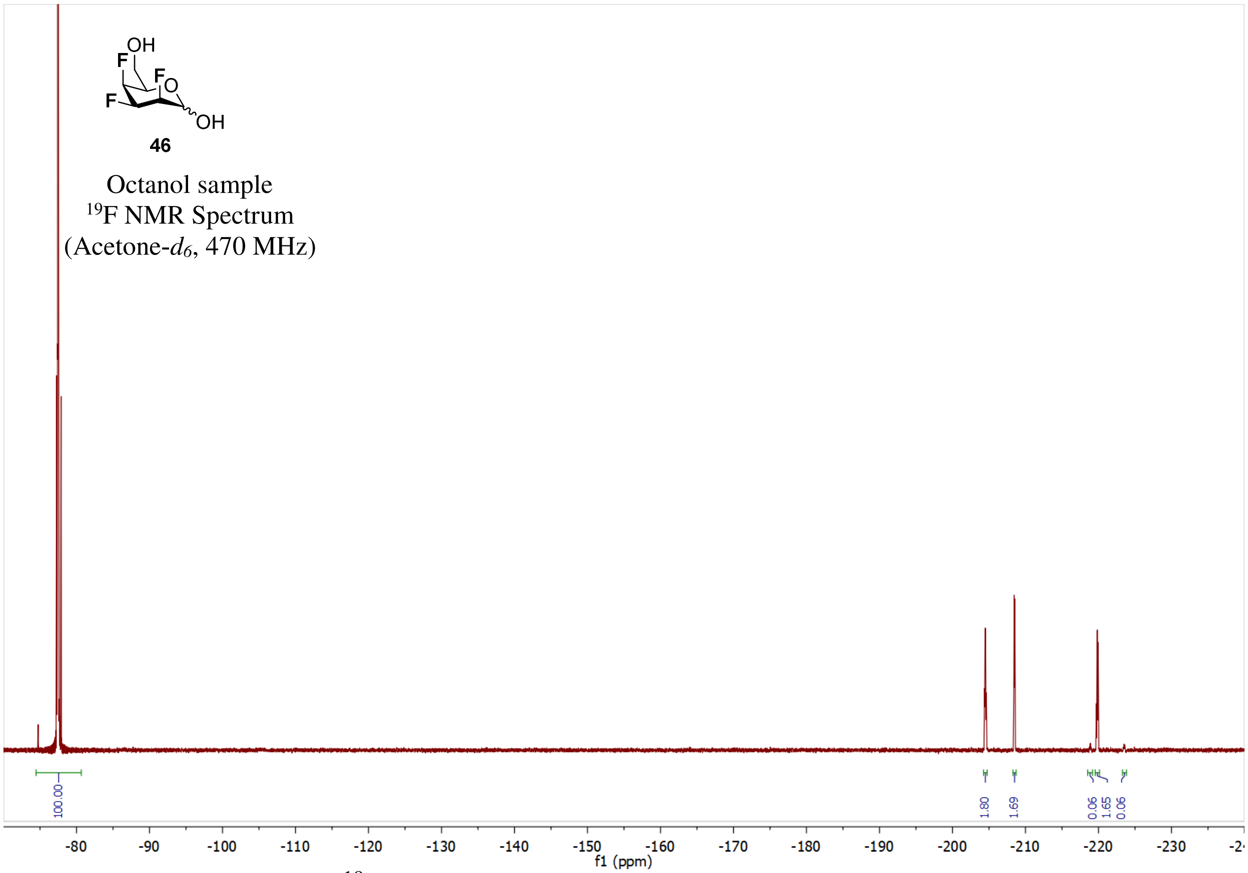

Supplementary Figure 169. ^{19}F NMR spectrum of the octanol sample of compound **46**

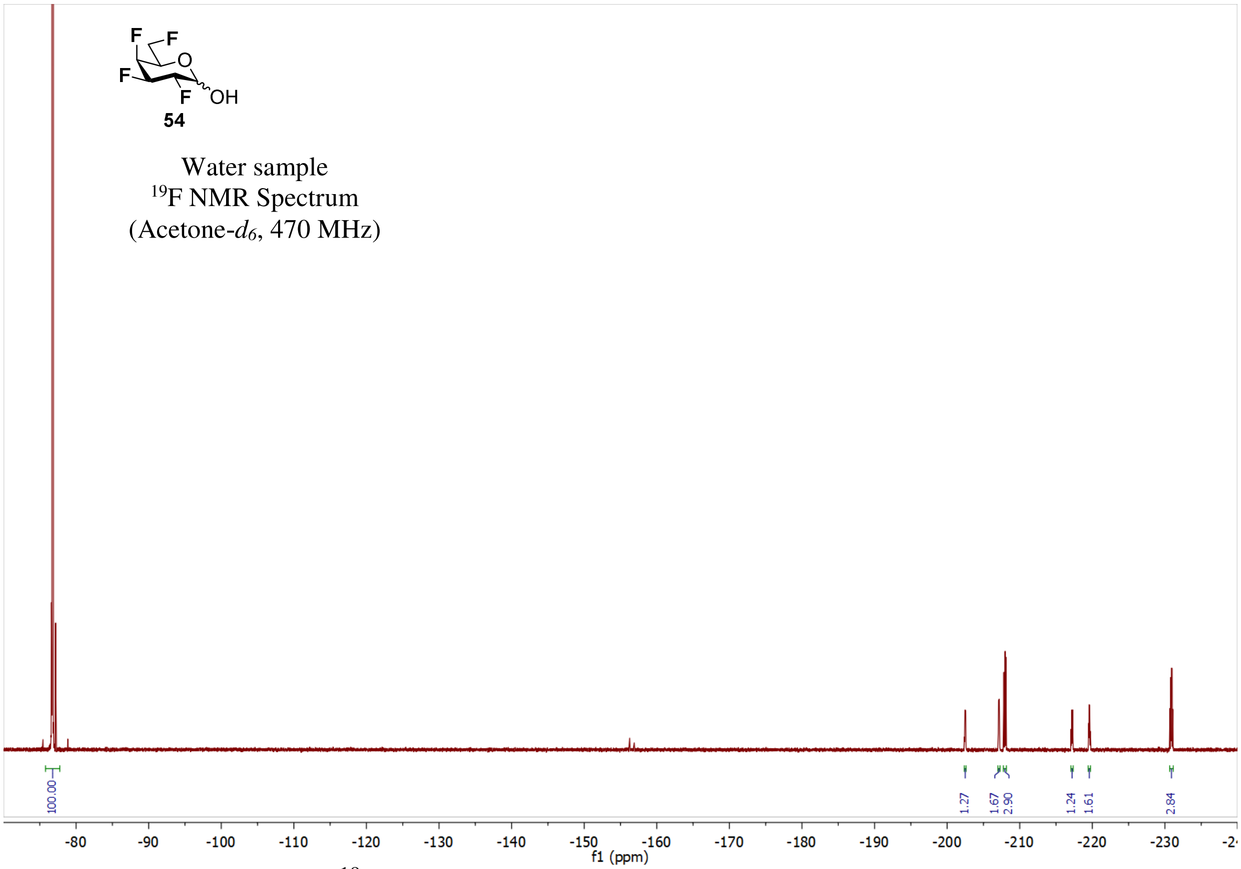

Supplementary Figure 170. ^{19}F NMR spectrum of the water sample of compound **54**

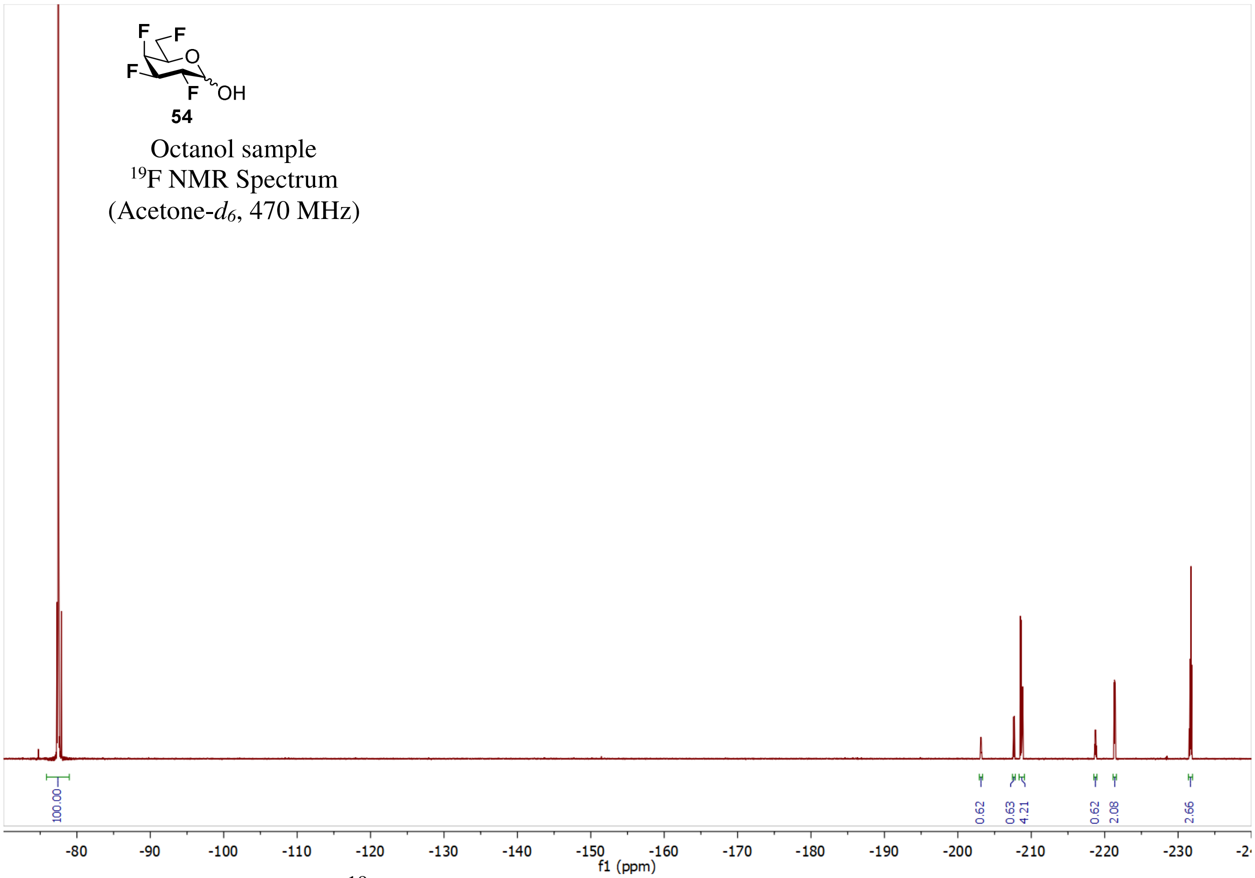

Supplementary Figure 171. ^{19}F NMR spectrum of the octanol sample of compound **54**

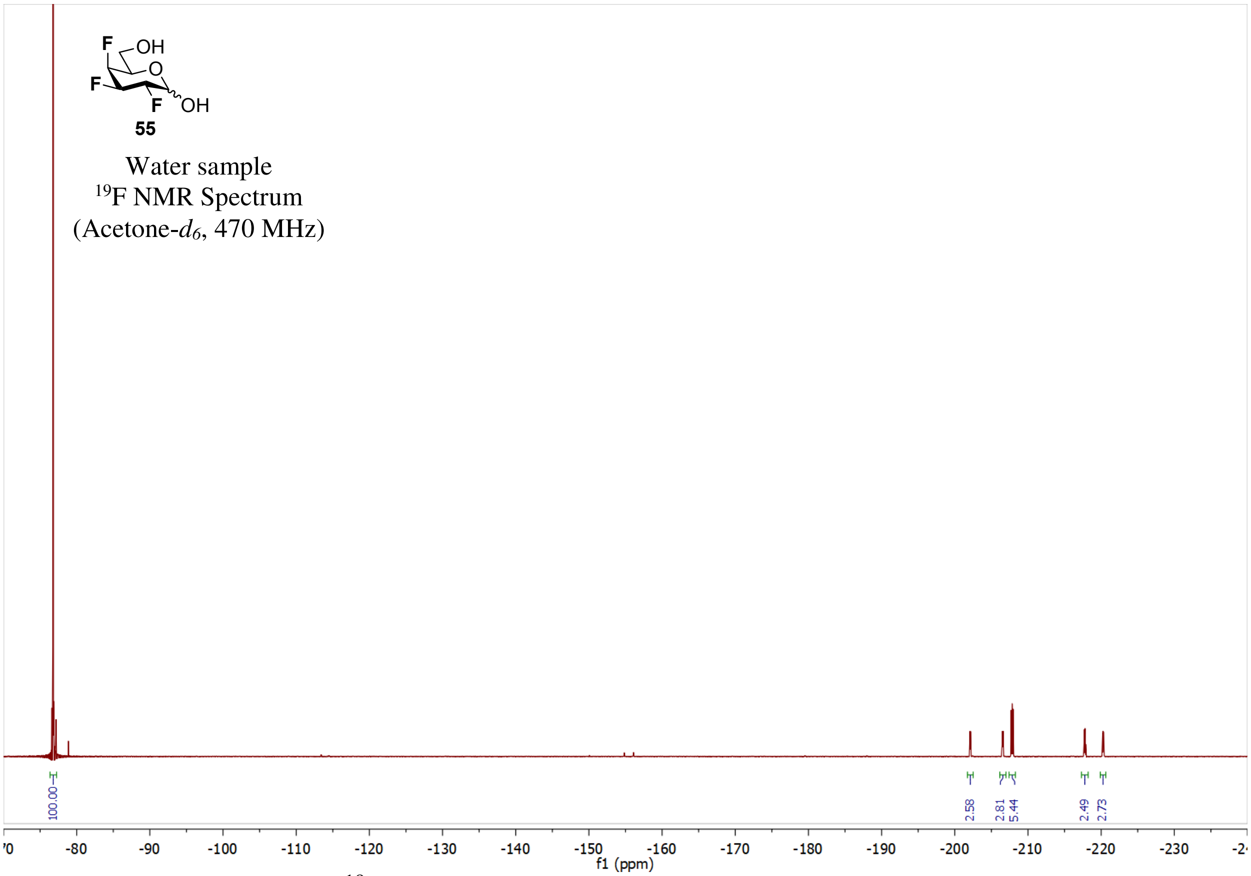

Supplementary Figure 172. ^{19}F NMR spectrum of the water sample of compound **55**

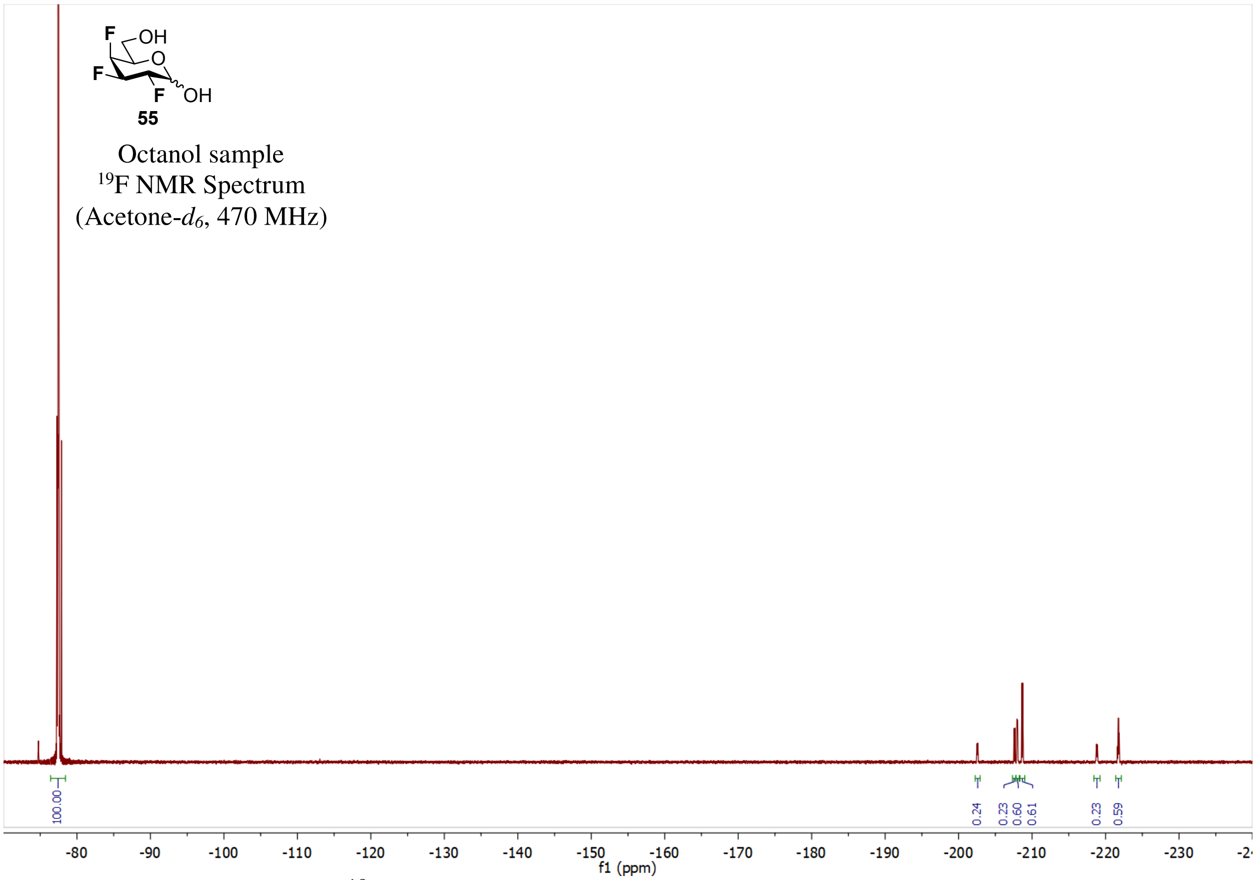

Supplementary Figure 173. ^{19}F NMR spectrum of the octanol sample of compound **55**

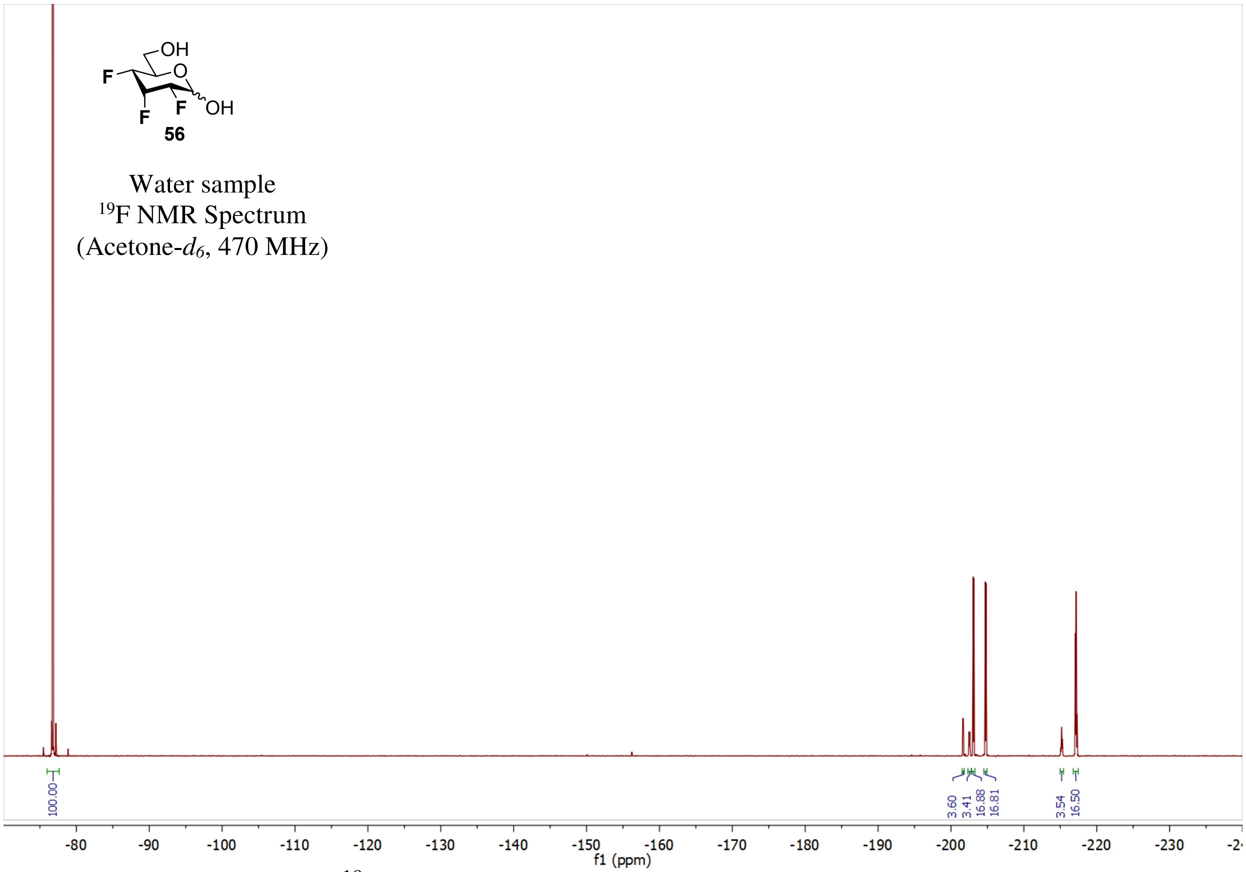

Supplementary Figure 174. ^{19}F NMR spectrum of the water sample of compound **56**

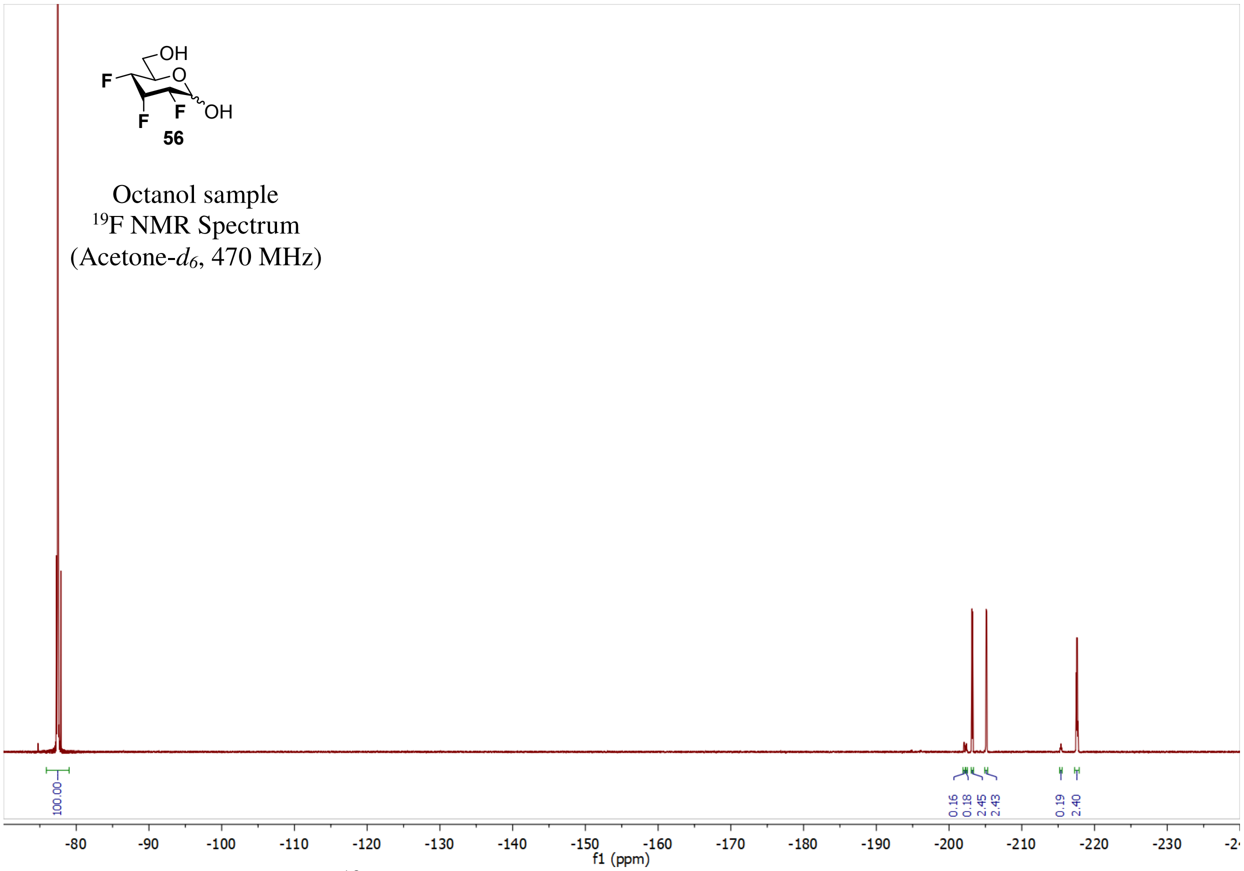

Supplementary Figure 175. ^{19}F NMR spectrum of the octanol sample of compound **56**

Supplementary References

[1] Pacak, J., Tocik, Z. & Cerny, M. Synthesis of 2-deoxy-2-fluoro-D-glucose. *J. Chem. Soc. Chem. Comm.* 77 (1969).

[2] Sosa, G. L., Peruchena, N. M., Contreras, R. H. & Castro, E. A. Topological and NBO analysis of hydrogen bonding interactions involving C–H···O bonds. *J. Mol. Struct.* **577**, 219–228 (2002).

[3] Sheldrick, B. The crystal structures of the α- and β-anomers of D-galactose. *Acta Cryst.* **B32**, 1016–1020 (1976).

[4] Longchambon, F., Avenel, D. & Neuman, A. Structure cristalline de l'α-D-mannopyrannose. *Acta Cryst.* **B32**, 1822–1826 (1976).

[5] Kroon-Batenburg, L. M. J., van der Sluis, P. & Kanters, J. A. Structure of β-D-allose, $C_6H_{12}O_6$. *Acta Cryst.* **C40**, 1863–1865 (1984).
